# PVGA: a precise viral genome assembler using an iterative alignment graph

**DOI:** 10.1093/gigascience/giaf063

**Published:** 2025-06-24

**Authors:** Zhi Song, Dehan Cai, Yanni Sun, Lusheng Wang

**Affiliations:** Department of Computer Science, City University of Hong Kong, Kowloon, Hong Kong SAR (HKG), China; Department of Electrical Engineering, City University of Hong Kong, Kowloon, Hong Kong SAR (HKG), China; Department of Electrical Engineering, City University of Hong Kong, Kowloon, Hong Kong SAR (HKG), China; Department of Computer Science, City University of Hong Kong, Kowloon, Hong Kong SAR (HKG), China; City University of Hong Kong Shenzhen Research Institute, Shenzhen, Guangdong Province, China

**Keywords:** genome assembler, virus genome, alignment graph, maximum total weight path, iterative method

## Abstract

**Background:**

Viral genome analysis is crucial for understanding virus evolution and mutation. Investigations into viral evolutionary dynamics and mutation patterns have garnered significant research attention since the outbreak of COVID-19. The basic structure of many virus genomes is highly conserved [1]. RNA viruses have high mutation rates, and single-nucleotide variations may induce substantial phenotypic alterations in terms of viral function and pathogenicity. Thus, special assembly methods are required for viral genome analysis.

**Result:**

PVGA starts with a reference genome and the sequencing reads. The first step in PVGA involves constructing an alignment graph based on a reference genome and the set of input sequencing reads. Then the optimal genomic path is determined through dynamic programming, maximizing the cumulative edge weights that reflect read support density across the alignment graph. The obtained path corresponds to a refined genome. Finally, we repeat the process by using the new reference genomes until no further improvement is possible. We evaluate PVGA’s performance across both assembly and polishing tasks using simulated and real datasets, including both long reads and short reads. The experiments demonstrate that PVGA always outperforms popular existing programs in terms of the quality of assembly results, while the running time of our method is compatible to others. In particular, simulated Nanopore datasets show that our method can correctly report the true genomes with 0 mismatches and 0 indels.

**Conclusions:**

PVGA is a novel viral genome assembler that seamlessly integrates assembly and polishing into a unified workflow. Its design prioritizes high accuracy, enabling the detection of subtle genomic variations that can impact viral function and pathogenicity. By addressing the unique challenges of viral genome assembly, PVGA provides a reliable and precise solution for advancing our understanding of viral evolution and behavior.

## Introduction

Viral genome analysis is crucial for understanding virus evolution and mutation. Investigations into viral evolutionary dynamics and mutation patterns have garnered significant research attention since the outbreak of COVID-19. The basic structure of many virus genomes is highly conserved [1]. RNA viruses have high mutation rates, and single-nucleotide variations may induce substantial phenotypic alterations in terms of viral function and pathogenicity. For example, genetic mutations in the coronavirus genome that alter the spike protein can affect its ability to interact with host cells, thereby affecting transmissibility and disease severity [2]. Thus, special assembly methods are required for viral genome analysis.

Sequencing of viral genomes primarily relies on next-generation sequencing (NGS) and third-generation sequencing (TGS) technologies. NGS is recognized for its short read lengths and high accuracy, with platforms such as Illumina. However, due to the short read lengths and the presence of repetitive regions, it is often challenging to assemble genomes accurately using NGS data. TGS technologies, such as Nanopore and PacBio, are effective for resolving complex genome structures and repetitive regions due to their longer read lengths. In comparison with NGS, TGS tends to have higher error rates. For instance, Nanopore sequencing relies on measuring electrical current changes as DNA passes through a nanopore, and factors such as pore condition, molecule speed, and signal noise can interfere with base-calling accuracy, making it difficult to distinguish adjacent bases, thereby reducing the quality of the reads [3]. PacBio sequencing, using its single-molecule real-time (SMRT) technology [4], is also widely applied for TGS. SMRT sequencing is well suited for detecting structural variations and resolving repetitive regions, but it frequently introduces insertion and deletion (indel) errors. To improve accuracy, PacBio introduced HiF i(high-fidelity) sequencing, which produces highly accurate long reads by repeatedly sequencing the same molecule [5]. However, this increased accuracy comes at a higher cost. Achieving high-quality viral genome assembly requires balancing sequencing accuracy with cost. Both the choice of sequencing technology and the assembly algorithms play critical roles in producing reliable genome assemblies.

Genome assembly techniques are broadly categorized into 2 types: *de novo* assembly and reference-guided assembly. *De novo* assembly tools include Velvet [6], ABySS [7], SPAdes [8], Flye [9], Canu [10], and Translig [11], which reconstruct the genome without relying on a reference genome. However, *de novo* methods often encounter challenges in highly repetitive regions, which may lead to misassemblies, redundant contigs, or gaps. Moreover, in regions of low coverage, *de novo* assembly may produce incomplete or missing sequences and further introduce gaps and errors. With the help of the reference genome sequence, one can obtain the locations of reads in the genome. Thus, reference-guided assembly methods can possibly fill gaps between reads and improve prediction accuracy in low-coverage regions. Famous reference-guided assembly methods include Novoalign [12], Maq [13], iVar [14], Accuvir [15], and bcftools [16].

There are some assemblers that are optimized for viruses. For instance, IVA was developed as a *de novo* assembler for RNA viruses, utilizing paired-end datasets to achieve more accurate assemblies [17]. Similarly, Accuvir [15] introduced a reference-based long-read assembler for viruses, primarily employing diverse beam search algorithms on alignment graphs to improve accuracy. In addition to assembly tools, genome polishing methods have become increasingly important for enhancing assembly accuracy by correcting errors using high-accuracy reads. Pilon [18] improves genome assemblies by analyzing read alignments, constructing a pileup structure to evaluate base-level evidence, and iteratively adjusting the assembly based on read quality and consistency. NextPolish [19] employs a combination of alignment-based error correction and iterative consensus polishing, utilizing short-read data to precisely correct mismatches and indels, further refining the final genome assembly. However, despite these advancements, current genome assembly tools still fail to achieve the requisite base-level accuracy for viral genome assembly.

In this article, we present PVGA, a novel viral genome assembler that can perform both assembly and polishing, effectively handling both long-read and short-read sequencing data. PVGA starts with a reference genome and utilizes the sequencing reads directly to reduce noise. The first step in PVGA involves constructing an alignment graph based on a reference genome and the set of input sequencing reads. Then the optimal genomic path is determined through dynamic programming, maximizing the cumulative edge weights that reflect read support density across the alignment graph. Finally, we repeat the process by using the new reference genomes until no further improvement is possible.

We evaluated PVGA’s performance across both assembly and polishing tasks using simulated and real datasets, including both long reads and short reads. The results demonstrate that PVGA consistently outperforms popular existing programs. In particular, simulated Nanopore datasets show that our method can correctly report the true genomes with 0 mismatches and 0 indels, except for some small errors at the 2 ends of the genomes.

## Methods

Our new method contains 3 steps. Step 1: We construct an alignment graph based on the set of input reads using an initial reference genome as the backbone. The initial reference genome should be from the same species. One can also generate an initial reference genome using an existing *de novo* assembler. Step 2: After constructing the alignment graph, we apply a dynamic programming algorithm to select a path supported by the largest number of read coverage and construct a new reference genome based on the path. Step 3: We then use the latest reference genome as the backbone to repeat steps 1–2. The process stops when the new reference genome is identical to the old one.

### Alignment graph construction

The graph construction method is inspired by the hierarchical genome assembly process (HGAP) proposed by Chin et al. [20]. The input contains 2 parts: read sequences and a backbone sequence. First, we construct the initial graph $G_b$ with *n* nodes $v_1, v_2, \ldots v_n$ and $n-1$ edges based on the backbone sequence $S=s_1s_2\ldots s_n$, where each node $v_i$ is labeled with the letter $s_i$ and there is an edge $(v_i, v_{i+1})$ connecting the 2 consecutive nodes. We then align each read $R=r_1r_2\ldots r_k$ with the reference sequence $G_b$.

**Figure 1:**
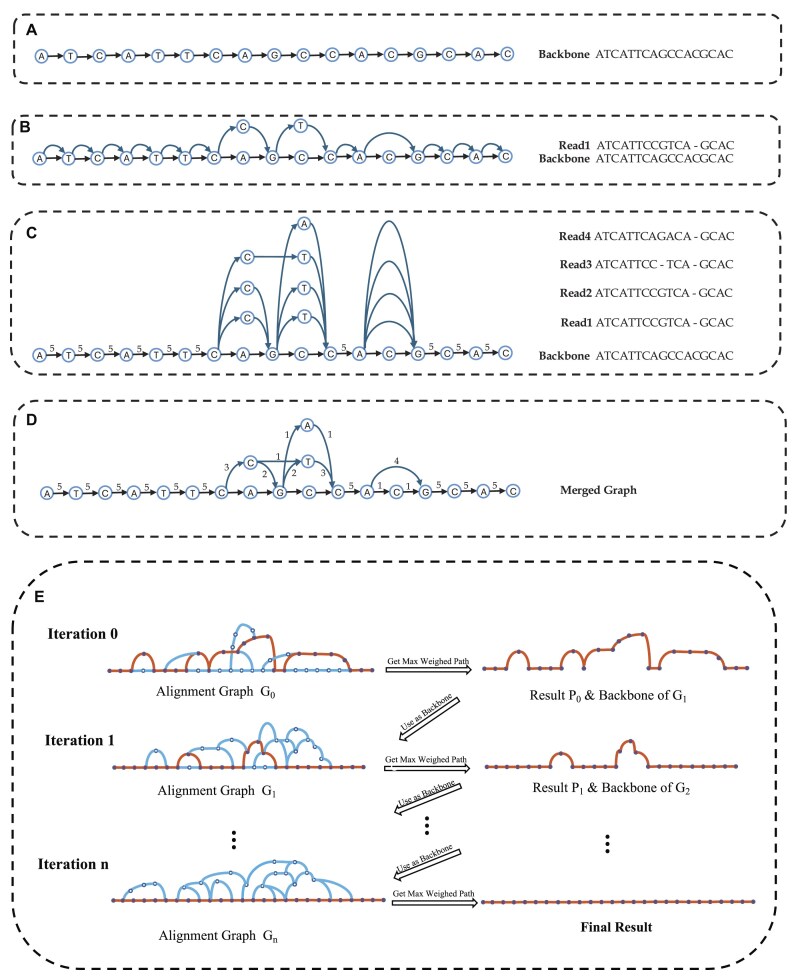
Flowchart of construction of alignment graph and iteration process. (A) PVGA takes a reference genome as backbone graph $G_B$. (B) PVGA aligns the first read Read1 to the backbone. (C) Four reads are aligned with the backbone, awaiting the subsequent merging process. (D) PVGA merges edges that point to the same node; the new edge’s weight is equal to the sum of the weights of the merged edges. This process can be performed either after aligning all reads or during the alignment process, with a final merge conducted after all reads have been aligned. (E) Iteratively construct the alignment graph using the result from the previous iteration as the backbone.

In the alignment, if $r_i$ is aligned to an identical letter $s_j$ in the backbone sequence, then $r_i$ corresponds to the existing node $v_j$ in the graph $G_b$. The weight of the edge $(v_{j-1}, v_j)$ will be incremented by 1, where $v_{j-1}$ is the predecessor node of $v_j$. If $r_i$ is aligned with a space or a letter $s_j$ not identical to $r_i$, we will create a new node $u_i$ labeled with $r_i$ and add an edge $(u_{i-1}, u_i)$ with weight 1, where $u_{i-1}$ is the node corresponding to the previous letter $r_{i-1}$. See Fig. 1B.

We will repeat the above process until all reads have been applied. The obtained intermediate graph is denoted as $G_I = (V, E)$. In the intermediate graph $G_I$, each edge is assigned a weight of 1, representing the number of supporting reads. To reduce the complexity of $G_I$, we merge nodes with the same label and the same parent repeatedly. When merging nodes into a new node $u^{\prime }$ with its predecessor *v*, the weight of the resulting edge $(v, u^{\prime })$ is updated to reflect the total number of supporting reads. Finally, if there are multiple edges between any 2 nodes *u* and *v* in $G_I$, we combine them into a single edge and update the weight accordingly. This process results in a simplified final alignment graph *G*.

### Finding a directed path in the alignment graph with maximum total weight

The weight on each edge is the number of supporting reads. In order to find a new reference sequence, we will try to find a path in *G* containing the maximum total weight. Such a path is the path supported by the largest number of reads.

We do a topological sorting on the set of nodes in *G* and obtain a linear order among the set of nodes in *G*. Let $\text{DP}[v]$ denote the maximum total weight of paths ending at node $v$. The value of $\text{DP}[v]$ can be computed as follows.


(1) \begin{eqnarray*}
DP[u] = \max _{v \in Pred(u)} \lbrace DP[v] + w(v, u)\rbrace ,
\end{eqnarray*}

where $w(v, u)$ is the weight of the edge from node $v$ to node $u$. We can compute all the $\text{DP}[v]$s according to the topological order. After that, we will find a node *v* with the largest $\text{DP}[v]$ value and use a standard backtracking process to get a path with maximum total weight on *G* ending at *v*.

**Running time:** To compute each $DP[u]$ in equation (1), we need $O( Deg(u) )$ time, where $Deg(u)$ is the in-degree of *u*. Therefore, the total running time complexity of this dynamic programming algorithm is $O(|E|)$, where $|E|$ represents the total number of edges in the graph.

### Updating the reference genome iteratively

The quality of the obtained maximum weight path heavily depends on the initial reference genome, as errors or biases in the initial reference can impact the alignment and subsequent path computation. To address this, we use the obtained maximum weight path as a new reference genome. A new alignment graph is then reconstructed using all the input reads and the updated reference genome. The maximum weight path is computed again based on this updated graph. This process is repeated iteratively to refine the assembly. With each iteration, the alignment graph and the resulting path become more accurate and consistent. Iteration stops when the genome obtained from the current iteration is identical to the genome from the previous iteration. This indicates that the backbone and the assembly result have reached a consistent state, and no further adjustment is possible using this method. This condition for algorithm termination seems to be very strong, and one may worry about the running time of this condition.

Recall that PVGA is for virus genome assembly, and the genome size is relatively small. Experimental results indicate that our method exhibits rapid convergence, often requiring no more than 3 or 4 iterations to reach a stable solution in practical applications. The running time comparison is in Fig. 5. Moreover, this iterative method can reach high-quality results, and experiments show that our iterative method always outperforms the state-of-the-art methods.

**Figure 2:**
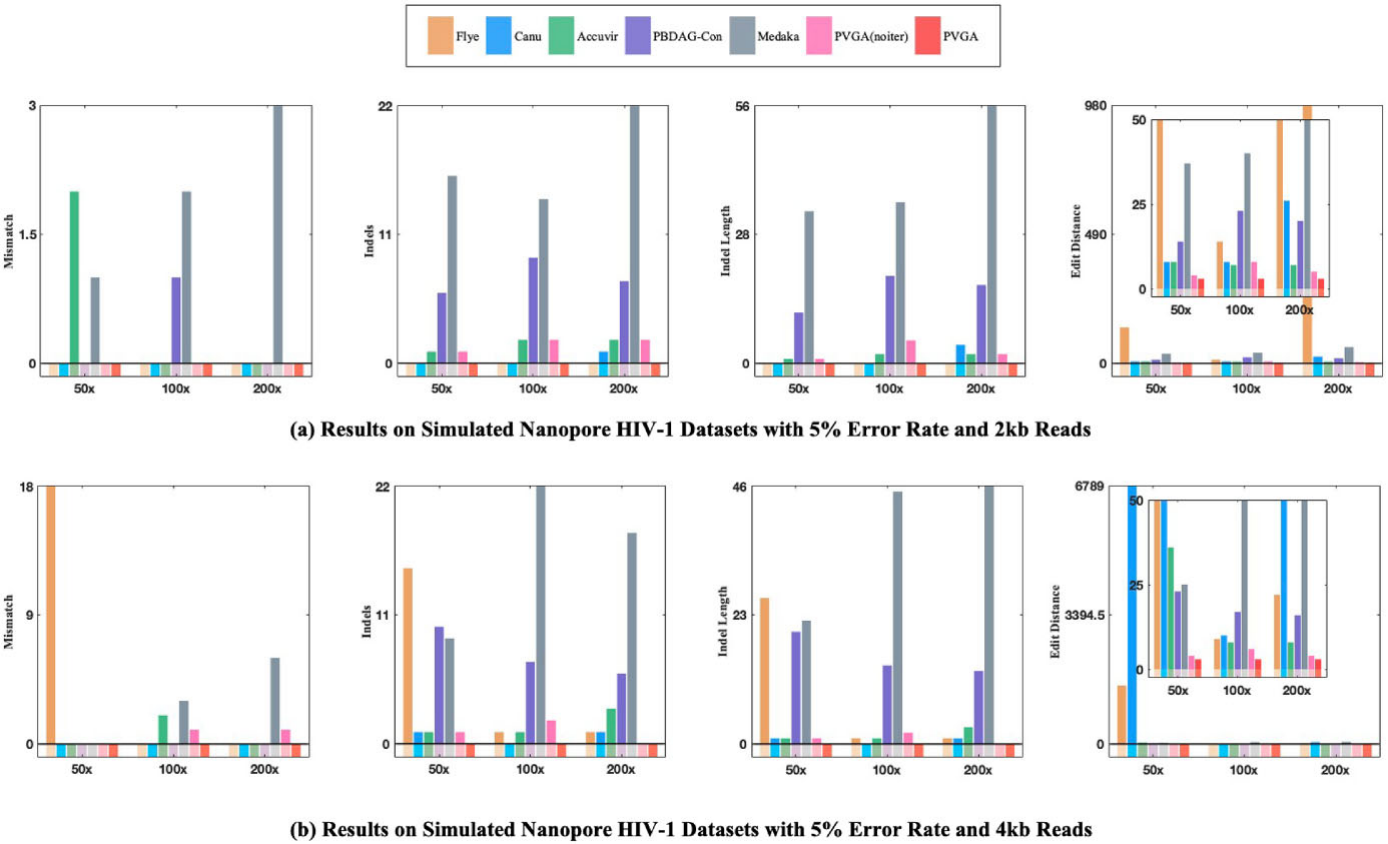
Results on simulated Nanopore HIV-1 datasets with an average read length of 2 kb and 4 kb, respectively. The 4 subfigures in each row represent mismatch, indels, indel length, and edit distance from left to right, respectively.

**Figure 3:**
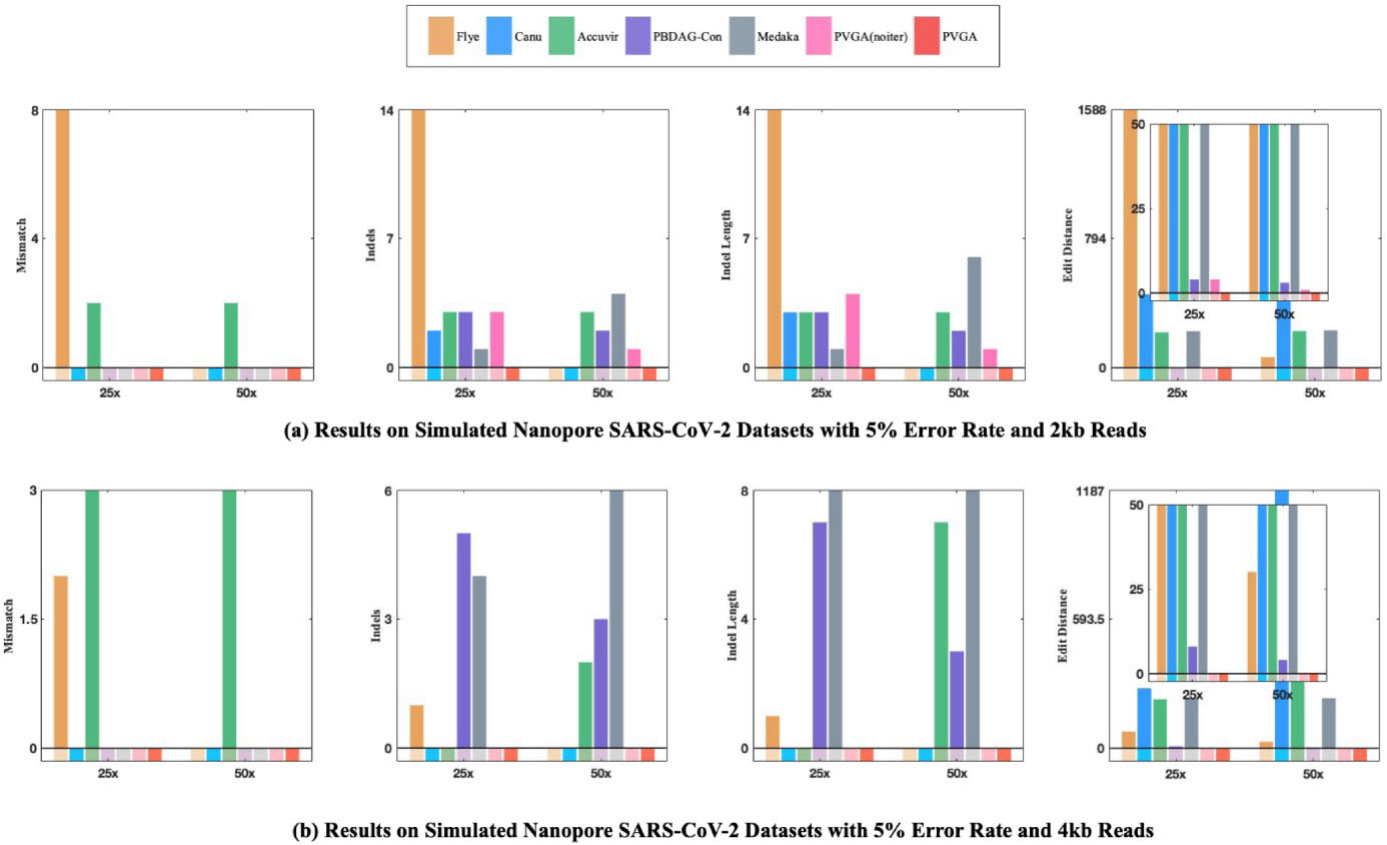
Results on simulated Nanopore SARS-CoV-2 datasets with an average read length of 2 kb and 4 kb, respectively. The 4 subfigures in each row represent mismatch, indels, indel length, and edit distance from left to right, respectively.

**Figure 4:**
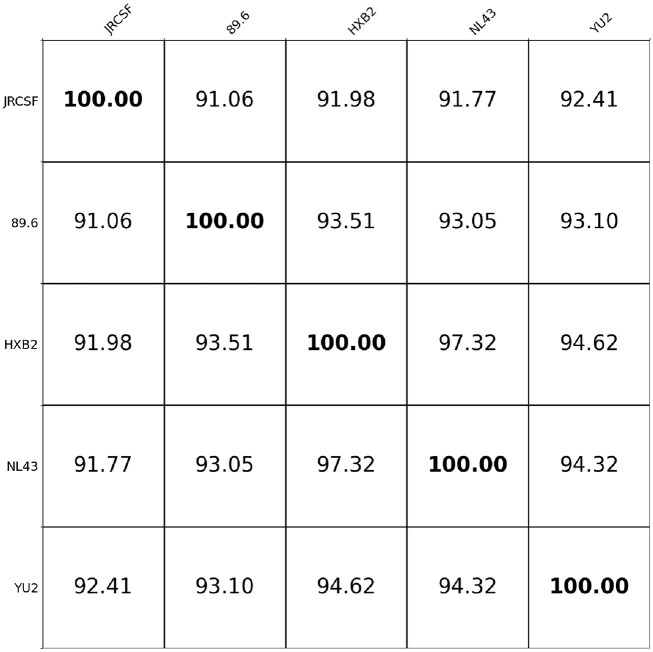
Pairwise similarity matrix of 5 HIV-1 strains.

**Figure 5:**
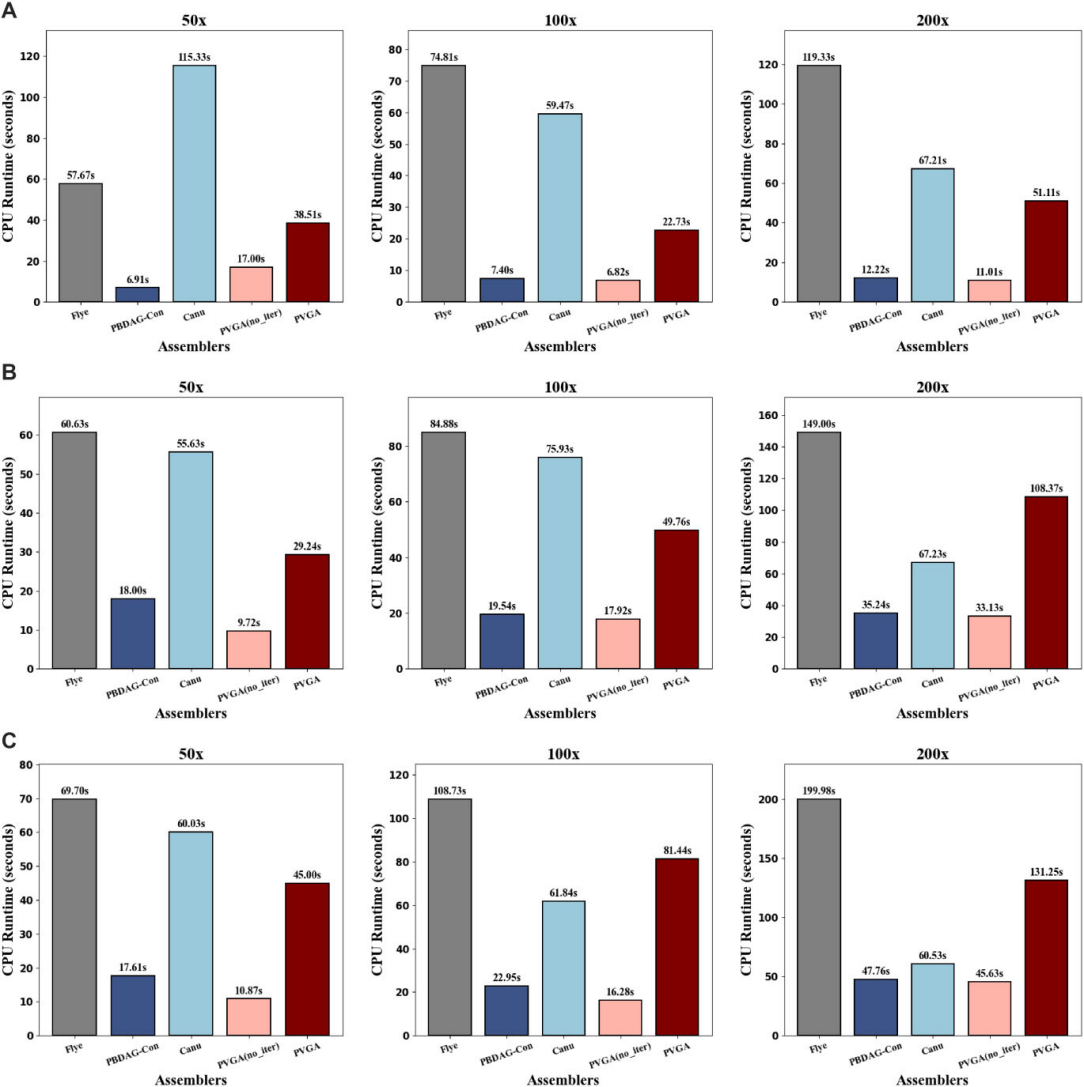
Comparison of CPU times for the 5 tools on the 3 datasets of 50×, 100×, and 200× coverage, respectively. (A) HIV-1 virus 89.6 strain. (B) Measles virus. (C) SARS-CoV-2.

## Results

### Datasets

We evaluate PVGA using both simulated and real viral sequencing data. To simulate a broad spectrum of sequencing conditions, we use Badread [21], which is designed for generating various kinds of simulated genomes. All real genome data and real sequencing reads used in this study are sourced from the National Center for Biotechnology Information (NCBI). These datasets include the following viral strains.

#### HIV-1

HIV-specific data provide a critical benchmark for evaluating assembly methods, given their widespread availability in public databases. We use the 89.6 strain (GenBank: U39362.2) as the target genome and generate simulated reads in bulk, with sizes detailed in the next subsection, to serve as the ground truth. The HXB2 strain (GenBank: K03455.1) is selected as the reference genome for constructing the initial alignment graph, which shares 93.58% similarity with the ground-truth 89.6 strain.

The haplotype benchmarking dataset [22] contains mixed PacBio reads from 5 strains (HXB2, 89.6, JR-CSF, NL4-3, YU-2). To obtain reads from a single strain, we first align the reads to the genomes of these strains using minimap2 [23]. We then extract the reads based on their closest aligned genome (identified by samtools [24]).

#### SARS-CoV-2

Severe acute respiratory syndrome coronavirus 2 (SARS-CoV-2) sequencing data are characterized by extensive genomic length and high sequence homology. We select SARS-CoV-2 isolate Wuhan-Hu-1 (NCBI Reference Sequence: NC_045512.2) as the reference genome and SARS-CoV-2 isolate (GenBank: OZ072292.1) as a target and to generate simulation reads.

#### Norovirus

Noroviruses are common pathogens that can cause acute gastroenteritis. We obtain third-generation Nanopore sequencing data of noroviruses (SRX10330013) from the National Food Virology Reference Centre at Health Canada [25]. This dataset consists of 5,741 spots, totaling 2.9 million bases, with the norovirus GII strain BMH19-097 serving as the ground-truth genome. We use the complete genome of Norovirus GII (NC_039477.1) as the reference for graph construction. We also employ actual Illumina sequencing data of the norovirus. We utilize SRR13951201 (35.8 M bases), SRR13951221 (60 M bases), and SRR13951199 (12.9 M bases) as input reads, with corresponding ground-truth data from Norovirus GII isolates BMH19-145, BMH13-039, and BMH14-056.

#### Ebola

We utilize 2 Ebola virus (EBOV) genomes. The first genome corresponds to the Ebola virus (EBOV-May) Mayinga strain, isolated in Zaire in 1976. This genome consists of 18,959 base pairs and is publicly available under the NCBI accession number AF086833.2. EBOV-May is a negative-sense, single-stranded RNA virus of the genus *Orthoebolavirus*, encoding 7 structural proteins such as nucleoprotein (NP), glycoprotein (GP), and RNA-dependent RNA polymerase (L) [26, 27]. The second Ebola virus strain is isolated from *Macaca fascicularis* and sequenced using IonTorrent technology. The genome, consisting of 18,871 base pairs, is publicly accessible under the NCBI accession number KY786027.1. It was assembled using the CLC Genomics Workbench v9.5.4 and serves as the ground-truth genome for generating synthetic reads in this study. The metadata associated with this genome are part of BioProject PRJNA379115 and BioSample SAMN06603499, with additional information submitted by Guedj et al. [28].

#### Measles

Measles virus (MV), a member of the genus *Morbillivirus* in the family Paramyxoviridae, is a highly contagious, negative-sense, single-stranded RNA virus. We utilize 2 MV strains. The first genome, a complete reference genome of measles *Morbillivirus*, was sourced from the NCBI RefSeq database (accession number NC_001498.1). This genome consists of 15,894 base pairs. The second genome is the MV genotype A transgenic strain vac2(GFP)H and serves as the ground-truth genome for generating synthetic reads. This genome, available under the GenBank accession number MH144178.1, spans 16,728 base pairs and includes a transgenic insertion of the green fluorescent protein (GFP) gene. It was sequenced using Sanger dideoxy sequencing and has been used in experimental studies for functional and structural analyses [29, 30].

### Evalution on simulation data

We evaluate the performance of several assemblers using simulated Nanopore and PacBio data. Tests are conducted on 2 viral strains, HIV-1, and SARS-CoV-2, under both standard and low-coverage conditions to assess their robustness.

We compare our method PVGA with the state-of-the-art methods, including *de novo* methods (Flye [9], Canu [10]) and reference-based methods (Accuvir [15], PBDAG-Con [20], and Medaka [31]). In addition, to illustrate the improvement achieved by the iterative step of PVGA, we also present the results of our method without the iterative step (referred to as “PVGA (no_iter)”).

We evaluate the assembly quality using several metrics, including genome fraction, mismatches, indels, indel length, and edit distance. Genome fraction represents the percentage of reference genome bases accurately matched by the assembled genome. Mismatches refer to the number of positions where the nucleotide in the assembly differs from the reference sequence. Indels refer to the number of insertions and deletions in the assembly relative to the reference. Indel length refers to the total length of the indels. Edit distance defines a minimum number of substitution and indel operations required to transform the assembled genome into the reference sequence. These metrics collectively measure the assembly’s accuracy and its deviation from the ground-truth genome.

#### Benchmarking on standard-length and depth HIV-1 simulated data

As the genome length increases, all methods tend to show higher error rates. However, PVGA consistently outperforms the compared assemblers. For our simulations, the average lengths of reads for Nanopore and PacBio were set at 2k, 4k, and 6k, with sequencing depths of 50×, 100×, and 200×.

To simulate sequencing data, we used the Badread tool with the error model parameter set to nanopore2023, which was trained on ONT R10.4.1 reads. The identity was set to (95, 99, 2.5), indicating a normal distribution with a mean of 95 and a standard deviation of 2.5. For PacBio sequencing, we set the error model in Badread to pacbio2021, trained on PacBio Sequel II HiFi reads. We use the same identity settings as (95, 99, 2.5). The results for Nanopore are presented in Fig. 2 and Table 1, while the results for PacBio are shown in the supplementary materials as Tables S1 to Table S3.

**Table 1.** Results on simulated Nanopore HIV-1 (genome length: 9,713 bp) datasets with 5% error rate and an average read length of 6 kb (“-” indicates that the assembler fails to produce a result).

Reads		Genome	Genome			Indel	Edit
depth	Tool	fraction	length	Mismatch	Indels	length	distance
50×	Flye	93.143	9,067	0	0	0	686
	Canu	100	9,748	0	0	0	35
	Accuvir	99.949	9,709	0	2	3	4
	PBDAG-Con	99.969	9,693	1	7	17	20
	medaka	99.969	9,724	2	12	30	35
	PVGA (no_iter)	99.969	9,706	0	2	4	7
	**PVGA**	**99.969**	**9,710**	**0**	**0**	**0**	**3**
100×	Flye	96.757	9,405	0	0	0	322
	Canu	−	−	−	−	−	−
	Accuvir	99.949	9,706	0	2	2	7
	PBDAG-Con	99.969	9,693	0	8	17	20
	medaka	99.969	9,728	1	22	46	50
	PVGA (no_iter)	99.969	9,709	2	1	1	6
	**PVGA**	**99.969**	**9,710**	**0**	**0**	**0**	**3**
200×	Flye	100	9,734	0	1	1	23
	Canu	−	−	−	−	−	−
	Accuvir	99.959	9,710	0	1	1	5
	PBDAG-Con	99.969	9,696	0	6	14	17
	medaka	99.969	9,732	7	26	68	77
	PVGA (no_iter)	99.969	9,705	1	5	5	9
	**PVGA**	**99.969**	**9,710**	**0**	**0**	**0**	**3**

Canu could not construct the assembly graph required for genome assembly, when the reads dataset lacks sufficient independent reads and effective overlaps.

From Table 1, we can see that PVGA achieves 0 mismatches and 0 indels with an edit distance of 3 across all Nanopore test cases. Figure 2, illustrates similar cases, where the average lengths of reads are 2 kb and 4 kb, respectively.

The PacBio results are illustrated in the supplementary material. As shown in Table 1 and Fig. 2, the iterative refinement process enhances assembly accuracy. For instance, with 6k read lengths and a depth of 200×, the process reduces indels from 5 to 0, mismatches from 1 to 0, and the edit distance from 9 to 3. The experiments show that increasing coverage improves performance for some assemblers. For PVGA, a coverage of 50× is sufficient to achieve 0 mismatches and 0 indels.

#### Benchmarking on low-coverage HIV-1 simulated data

Sequencing costs increase with coverage. An efficient assembler should perform well for high-coverage and lower-coverage conditions to support cost-effective sequencing. Thus, we further test the cases, where the coverage is 30×, 25×, and 20×, respectively, with an average read length of 2 kb. See Table 2.

**Table 2.** Results on simulated Nanopore HIV-1 (genome length: 9,713 bp) datasets with low coverage: 30×, 25×, and 20×

Reads		Genome	Genome			Indel	Edit
depth	Tool	fraction	length	Mismatch	Indels	length	distance
30×	Flye	70.751	6,917	0	1	1	2,888
	Canu	93.452	11,602	0	1	1	4,147
	Accuvir	99.938	9,707	1	3	4	11
	PBDAG-Con	99.969	9,695	0	7	15	18
	Medaka	99.969	9,714	1	9	18	22
	PVGA (no_iter)	99.969	9,706	0	1	4	7
	**PVGA**	**99.969**	**9,710**	**0**	**0**	**0**	**3**
25×	Flye	77.906	7,576	0	0	0	2,155
	Canu	93.452	11,156	0	1	1	7,325
	Accuvir	99.959	9,713	3	3	4	11
	PBDAG-Con	99.969	9,688	0	8	22	25
	Medaka	99.969	9,711	2	8	17	22
	PVGA (no_iter)	99.969	9,709	0	1	1	4
	**PVGA**	**99.969**	**9,710**	**0**	**0**	**0**	**3**
20×	Flye	96.788	9,440	0	1	1	353
	Canu	93.452	11,549	0	4	5	5,918
	Accuvir	99.949	9,708	2	2	2	9
	PBDAG-Con	99.969	9,688	0	13	24	27
	Medaka	99.969	9,714	0	10	20	23
	PVGA (no_iter)	99.969	9,710	0	0	0	3
	**PVGA**	**99.969**	**9,710**	**0**	**0**	**0**	**3**

At these lower-coverage levels, PVGA continues to show robust performance. As shown in Table 2, at coverage levels of 30×, 25×, and 20×, while some assemblers experience a noticeable drop in accuracy, PVGA consistently achieves the best performance with 0 mismatches and 0 indels. To figure out the threshold at which the accuracy of PVGA begins to decline, we test the case, where the coverage ranges from 19× down to 15×, with an average read length of 2 kb. The results are shown in Table 3.

**Table 3.** Results on simulated Nanopore HIV-1 (genome length: 9,713 bp) datasets with low coverage (<20×)

Reads		Genome	Genome			Indel	Edit
depth	Tool	fraction	length	Mismatch	Indels	length	distance
19×	Flye	88.963	8,673	0	0	0	1,104
	Canu	93.452	11,853	0	5	6	5,044
	Accuvir	99.866	9,709	0	1	1	8
	PBDAG-Con	99.969	9,682	1	11	28	32
	Medaka	99.969	9,707	3	11	17	23
	PVGA (no_iter)	99.969	9,707	0	1	3	6
	**PVGA**	**99.969**	**9,710**	**0**	**0**	**0**	**3**
18×	Flye	93.926	9,140	0	3	3	613
	Canu	99.969	9,708	0	2	2	5
	Accuvir	99.959	9,711	4	5	6	14
	PBDAG-Con	99.969	9,686	0	11	24	27
	Medaka	99.969	9,707	1	8	14	18
	PVGA (no_iter)	99.969	9,709	0	1	1	4
	**PVGA**	**99.969**	**9,710**	**0**	**0**	**0**	**3**
17×	Flye	72.614	7,085	0	2	2	2,694
	Canu	99.866	10,907	0	5	5	1,226
	Accuvir	99.753	9,713	1	1	2	12
	PBDAG-Con	99.969	9,685	0	8	25	28
	Medaka	99.969	9,711	0	7	23	26
	PVGA (no_iter)	99.969	9,710	0	0	0	3
	**PVGA**	**99.969**	**9,710**	**0**	**0**	**0**	**3**
16×	Flye	93.092	9,047	0	3	3	682
	Canu	93.452	11,805	0	3	3	4,126
	Accuvir	99.959	9,719	0	7	10	14
	PBDAG-Con	99.969	9,696	1	6	14	18
	Medaka	99.969	9,707	0	1	3	7
	PVGA (no_iter)	99.969	9,710	0	2	2	5
	**PVGA**	**99.969**	**9,711**	**0**	**1**	**1**	**4**
15×	Flye	96.87	9,402	0	7	7	315
	Canu	94.399	9,163	1	6	6	551
	Accuvir	99.856	9,693	0	7	8	22
	PBDAG-Con	99.969	9,687	2	12	27	32
	Medaka	99.969	9,703	3	6	13	19
	PVGA (no_iter)	99.969	9,706	0	4	4	7
	**PVGA**	**99.969**	**9,706**	**0**	**4**	**4**	**7**

As shown in Table 3, at coverages of 19×, 18×, and 17×, PVGA maintains zero indels. When the coverage drops to 16×, PVGA has its first indel with a length of 1, increasing to 4 at 15×. Despite this, PVGA continues to outperform all other assemblers, with the lowest indel count, indel length, mismatch rate, and edit distance. These results demonstrate PVGA’s robustness under low-coverage conditions, highlighting its potential to reduce sequencing costs while maintaining accuracy.

#### Benchmarking on simulated SARS-CoV-2 data

The SARS-CoV-2 virus is one of the RNA viruses with a long genome, approximately 29.9 kb. Although SARS-CoV-2 variants are highly similar, a few differences can lead to distinct biological properties such as pathogenicity, transmissibility, and immune response. Therefore, assembling an accurate SARS-CoV-2 genome is essential. In this section, we conduct experiments on simulated SARS-CoV-2 data at different depths (25× and 50×) and read lengths (2 kb, 4 kb, and 8 kb) for both Nanopore and PacBio datasets to evaluate the performance of tools on a highly similar virus with a long genome. Results for Nanopore are presented in Table 4 and Fig. 3, while PacBio results are included in the supplementary material as Tables S4 to Table S6.

**Table 4.** Results on simulated Nanopore SARS-CoV-2 (length: 29,646 bp) datasets with 5% error rate and an average read length of 8 kb

Reads		Genome	Genome			Indel	Edit
depth	Tool	fraction	length	Mismatch	Indels	length	distance
25×	Flye	100	29,656	0	0	0	10
	Canu	96.711	28,669	0	6	11	977
	Accuvir	100	29,872	0	3	3	226
	PBDAG-Con	100	29,646	0	14	1	2
	Medaka	100	29,878	0	2	8	232
	PVGA (no_iter)	100	29,644	0	4	4	4
	**PVGA**	**100**	**29,646**	**0**	**0**	**0**	**0**
50×	Flye	99.98	29,645	0	0	0	11
	Canu	97.834	28,999	0	2	5	647
	Accuvir	100	29,869	0	3	3	223
	PBDAG-Con	100	29,672	0	0	0	26
	Medaka	100	29,646	0	5	11	235
	PVGA (no_iter)	100	29,643	0	0	0	3
	**PVGA**	**100**	**29,646**	**0**	**0**	**0**	**0**

Table 4 shows that both PVGA and Flye achieve zero indels and mismatches. However, PVGA demonstrates superior performance, reconstructing the genome flawlessly, with no errors even at the 2 ends of the genome.

### Evaluation on poor sequencing conditions

With the advancement of sequencing technologies, there has been a significant leap in both the capabilities and quality of sequencing. For instance, PacBio sequencing technology can offer HiFi reads that provide an accuracy of 99.9%. However, some laboratories continue to rely on older sequencing equipment or encounter suboptimal results due to experimental limitations. In such cases, there is a need for an assembler capable of effectively handling data with relatively higher error rates. A study by the MinION Analysis and Reference Consortium reported that the median total error of all 2-dimensional (2D) reads was 12%, with 2D pass reads showing a slightly lower error rate of 10.5% [32]. Additionally, after basecalling, the global error rate of raw reads is typically around 10% [33].

To simulate poor sequencing conditions, we configured the following parameters for evaluating assembler performance under suboptimal data quality: we apply a truncated normal distribution of basecall identity (range: 85–95%, mean: 90%, SD: 5%), resulting in an average read error rate of 10% and an upper accuracy bound of 95%. We assign uniform sequencing depth (30$\times$) and average read length (4 kb) across all viral genomes (HIV-1, measles, Ebola) to standardize suboptimal quality conditions. The results are shown in Table 5.

**Table 5.** Results on simulated Nanopore HIV-1, measles, and Ebola virus datasets with a 10% error rate and an average read length of 4 kb with an average depth of 30×

		Genome	Genome			Indel	Edit
Virus	Tool	fraction (%)	length	Mismatch	Indels	length	distance
HIV-1	Flye	100	9,732	0	16	17	53
(Length: 9,713 bp)	Canu	99.053	16,468	0	36	43	6,993
	Accuvir	99.835	9,683	0	14	14	30
	PBDAG-Con	99.969	9,645	1	26	67	71
	PVGA (no_iter)	99.969	9,720	1	26	38	42
	**PVGA**	**99.969**	**9,717**	**0**	**7**	**11**	**14**
Measles	Flye	99.988	13,899	0	17	20	2,829
(Length: 16,728 bp)	Canu	99.815	16,674	2	21	23	56
	Accuvir	99.994	16,725	4	9	12	17
	PBDAG-Con	94.996	15,879	1	10	12	850
	PVGA (no_iter)	100	16,733	2	12	17	19
	**PVGA**	**100**	**16,734**	**1**	**5**	**8**	**9**
Ebola	Flye	99.989	18,851	1	18	18	21
(Length: 18,871 bp)	Canu	99.862	18,824	1	16	21	48
	Accuvir	100	18,979	9	26	28	123
	PBDAG-Con	100	18,858	0	13	13	13
	PVGA (no_iter)	100	18,874	0	12	13	13
	**PVGA**	**100**	**18,879**	**0**	**7**	**8**	**8**

For HIV-1, the 89.6 strain is used as the target to simulate reads, with the HXB2 strain serving as the backbone. For the measles virus, the NC_001498.1 sequence (15,894 bp) is used as the backbone, while the vac2(GFP)H sequence (length: 16,728 bp) served as the target. For the Ebola virus, the EBOV-May strain (18,959 bp) is used as the backbone, with the KY786027 strain serving as the ground truth for read simulation.

### Evaluation on real data

Although there are many available long-read sequencing datasets of viruses, most of them lack ground-truth genomes for validation. Thus, we use norovirus and HIV-1 to evaluate the tools’ performance, as they have ground-truth genomes from both long-read and short-read sequencing data.

For the HIV-1 real datasets, we collect PacBio sequencing data from a mock HIV-1 community [22]. To create the datasets for viral genome reconstruction, we separate this dataset into read sets from 5 HIV-1 strains by aligning them to the ground-truth genomes using the best hit. We test the 89.6, JR-CSF, and YU-2 subtypes, using HXB2 as the backbone to construct the alignment graph. Given that real data often contain gaps between reads, most *de novo* assemblers fail to achieve consensus or produce only very short contigs. As shown in Table 6, as for the 89.6 strain, Canu produces a contig of only 4,593 bp. Flye, on the other hand, encounters errors during real data processing, resulting in an unsuccessful assembly. Due to the lower quality of real reads, assemblers display higher mismatches, indels, and edit distances in the HIV-1 89.6 strain than observed in simulations. However, PVGA still outperforms all other assemblers. In the JR-CSF results, apart from Canu, which failed to assemble a complete genome, only PVGA and PBDAG-Con maintained single-digit mismatches, with PVGA showing lower indels, indel length, and edit distance.

**Table 6.** Results on real HIV-1 strain datasets (89.6, JR-CSF, YU-2)

		Genome	Genome			Indel	Edit
Strain	Tool	fraction	length	Mismatch	Indels	length	distance
89.6 strain	Canu	47.287	4,593	6	0	0	5,127
(Length: 9,713 bp)	Accuvir	99.990	9,710	33	4	4	30
	Medaka	99.856	9,733	25	6	22	74
	PBDAG-Con	100	9,711	24	2	2	40
	PVGA (no_iter)	100	9,709	25	2	2	36
	**PVGA**	**100**	**9,710**	**24**	**1**	**1**	**28**
JR-CSF	Canu	88.512	8,448	6	1	1	1,320
(Length: 9,535 bp)	Accuvir	99.727	9,736	36	3	3	241
	PBDAG-Con	99.99	9,720	5	8	20	221
	Medaka	97.735	9,535	33	8	42	316
	PVGA (no_iter)	99.99	9,610	3	1	1	159
	**PVGA**	**99.99**	**9,610**	**3**	**1**	**1**	**159**
YU-2	Canu	86.750	8,473	5	2	4	1,343
(Length: 9,706 bp)	Accuvir	99.727	9,713	5	5	9	16
	PBDAG-Con	100.000	9,705	4	7	19	23
	Medaka	100.000	9,698	28	9	52	80
	PVGA (no_iter)	100.000	9,615	3	9	13	16
	**PVGA**	**100.000**	**9,617**	**3**	**7**	**11**	**14**

We also test our method on real norovirus data (SRX10330013). As shown in Table 7, our method PVGA results in the fewest mismatches, indels, and the lowest edit distance among the 5 assemblers evaluated. This demonstrates that our PVGA method more effectively utilizes information from the alignment graph compared to PBDAG-Con, which focuses on assigning scores to nodes to maximize consensus, and Accuvir, which employs diverse beam search. While a diverse beam search approach increases the diversity of candidate paths, it often falls into local optima, failing to achieve the best results.

**Table 7.** Results on real Nanopore noroviruses (SRX10330013), with ground-truth genome as Norovirus GII isolate BMH19-097 (length: 7,618 bp)

	Genome	Genome			Indel	Edit
Tool	fraction	length	Mismatch	Indels	length	distance
Flye	92.964	7,097	0	11	13	577
Canu	99.593	7,632	0	12	14	104
Accuvir	99.396	7,564	1	8	10	57
PBDAG-Con	99.383	7,562	1	7	9	57
**PVGA**	**99.383**	**7,569**	**0**	**4**	**4**	**51**

To assess the effectiveness of the PVGA method with extensive short-read datasets, we employ actual Illumina sequencing data of the norovirus. The Norovirus GII complete genome (NC_039477.1) serves as the reference framework for graph construction. We utilize SRR13951201, SRR13951221, and SRR13951199 as input reads, with corresponding ground-truth data from Norovirus GII isolates BMH19-145, BMH13-039, and BMH14-056. The results are in Table 8. In all 3 Illumina norovirus datasets, PVGA achieves exceptional accuracy, with no mismatches, indels, or indel length errors except for some small misalignment at the 2 ends of the genomes. This verifies PVGA’s excellent performance in assembling short-read datasets as well.

**Table 8.** Results on real Illumina norovirus

	Genome	Genome			Indel	Edit
Reads	fraction	length	Mismatch	Indels	length	distance
SRR13951201	100	7,572	0	0	0	2
SRR13951221	100	7,567	0	0	0	17
SRR13951199	99.574	7,485	0	0	0	20

Ground-truth genome lengths: SRR13951201 (BMH19-145): 7,570 bp; SRR13951221 (BMH13-039): 7,550 bp; SRR13951199 (BMH14-056): 7,505 bp.

### Benchmarking the capability of polishing

The results from low-coverage HIV-1 sequencing data indicate that, at extremely low depths, some *de novo* assemblers, such as Flye and Canu, fail to obtain near-optimal solutions. Comparisons with the ground truth reveal that these assemblers do not achieve complete reconstruction in terms of both length and accuracy. For instance, at a coverage of 15×, Flye shows a high edit distance of 315, and Canu has a high edit distance of 551. These errors considerably compromise the accuracy of the assembly, adversely impacting subsequent tasks, such as protein structure prediction [34].

To address these inaccuracies, the next essential step is to polish the assembled sequences to enhance their accuracy. A common approach is to use hybrid methods that integrate high-quality short reads, such as those from Illumina, with flawed assemblies. Polishing tools such as Pilon [18], which utilizes a mapping-based method, align these short reads to the assembly and apply a Bayesian model to determine the most accurate sequence by considering base quality scores and error frequencies. Another tool, NextPolish [19], similarly aligns short reads and uses an iterative process to correct errors in small regions of the assembly.

To evaluate the genome polishing performance of PVGA against NextPolish and Pilon, we conduct experiments to polish the assembly results generated by the PVGA and *de novo* assembler Canu in previous experiments, respectively. The first involves a 9,706-bp HIV-1 genome assembled by PVGA from 15$\times$ coverage Nanopore reads (2-kb simulated read length; see Table 3), while the second consists of a 29,493-bp SARS-CoV-2 genome generated by Canu using 50$\times$ coverage long reads (2-kb average length; Fig. 3).

We simulate Illumina short reads via Badread by setting parameter qscores as ideal (read length range 200–300 bp), with coverage parameters specifically calibrated: 15$\times$ for HIV-1 and 25$\times$ for SARS-CoV-2. The results in Table 9 demonstrate that PVGA outperforms NextPolish and Pilon in terms of polishing.

**Table 9.** Polished results of 15× coverage HIV-1 simulation data and 25× coverage SARS-CoV-2 simulation data

		Genome	Genome			Indel	Edit
Virus	Tool	fraction	length	Mismatch	Indels	length	distance
HIV-1	NextPolish	99.969	9,722	2	12	12	17
(Length: 9,713 bp)	Pilon	99.969	9,707	0	3	3	6
	**PVGA**	**100**	**9,710**	**0**	**0**	**0**	**3**
SARS-CoV-2	NextPolish	99.906	29,636	2	26	28	58
(Length: 29,646 bp)	Pilon	99.906	29,555	0	63	63	91
	**PVGA**	**99.906**	**29,619**	**0**	**3**	**3**	**31**

### Convergence performance across different backbones

In this subsection, we investigate how the choice of backbone influences the resulting genome assembly. We simulate the HIV-1 JRCSF strain nanopore reads with an average read length of 4 kb and coverage of 30× for the assembly. The error rate of simulation JRCSF reads is 5%. To evaluate the impact of different backbone sequences, we use 4 well-characterized HIV-1 reference genomes, including strains 89.6, HXB2, NL43, and YU2 as backbones. In addition, we also apply the *de novo* assembly tool Flye [9] on our simulated reads, and the obtained genome sequence is also used as the backbone. The Flye-derived backbone has an edit distance of 20 from the ground-truth genome. The similarities between the JRCSF ground-truth genome and the various backbones are as follows: 89.6 (91.45%), HXB2 (92.78%), NL43 (92.55%), YU2 (92.83%), and Flye-derived assembly result (99.1%). Here, the similarity between the 2 sequences is 1 − edit distance over the length of the ground-truth genome. The similarities between the 5 different HIV-1 strain genome sequences are shown in Figure 4. The results are shown in Table 10.

**Table 10.** Results on simulated Nanopore HIV-1 JRCSF strain reads with a 5% error rate on different HIV-1 strain backbones

	Backbone	Genome	Genome			Indel	Edit
Virus	genome	fraction	length	Mismatch	Indels	length	distance
JRCSF	Flye-derived backbone	100	9,540	0	0	0	0
(Length: 9,540 bp)	89.6	99.99	9,539	0	0	0	1
	HXB2	99.99	9,539	0	0	0	1
	NL43	99.99	9,539	0	0	0	1
	YU2	99.99	9,539	0	0	0	1

The similarities between the JRCSF ground-truth genome and the 4 backbones are as follows: 89.6 (91.45%), HXB2 (92.78%), NL43 (92.55%), YU2 (92.83%), and the Flye assembly genome (99.1%).

From Table 10, we can see that for the backbones derived from 4 different HIV-1 strains, PVGA can obtain the same genome sequence with edit distance 1, and no indel or mismatch. In fact, there is just 1 missing base at the end of the obtained genome sequence. For the backbone generated by the *de novo* assembler Flye, PVGA eliminates the 20-edit-distance discrepancy introduced by the *de novo* approach, ultimately recovering the genome sequence identical to the ground-truth genome.

To further investigate how the degree of divergence between the backbone and the ground truth impacts assembly results, we modify the HIV-1 JRCSF genome and randomly replace 15%, 20%, 25%, and 30% of the bases in the HIV-1 JRCSF genome to create backbones with different divergence. For the simulation reads, we maintain a coverage of 30× and an average read length of 4 kb.

As shown in Table 11, with the increase of diversity between the backbone and the ground-truth genome, our method can still obtain good results in terms of mismatches and indels, while the edit distance increases and the obtained genome length decreases. Basically, the obtained genome missed some bases at the 2 ends but can still perfectly match the ground-truth genome in the middle. The reason is that very few reads completely cover the ends, and with the increase of backbone diversity, the few reads covering the ends have a higher possibility of being aligned to the wrong places when constructing the alignment graph.

**Table 11.** Results on simulated Nanopore HIV-1 JRCSF strain reads with different backbones

	Backbone	Genome	Genome			Indel	Edit
Virus	similarity	fraction	length	Mismatch	Indels	length	distance
JRCSF	85%	99.948	9,535	0	0	0	5
(Length: 9,540 bp)	80%	99.937	9,533	0	0	0	7
	75%	99.843	9,525	0	0	0	15
	70%	99.644	9,506	0	0	0	34

### Evaluation of computing resource usage

We evaluate the CPU runtime and memory usage of PVGA in comparison with several widely used assemblers. The datasets used in the experiments included HIV-1, measles, and SARS-CoV-2, with sequencing coverages of 50$\times$, 100$\times$, and 200$\times$, and respective genome lengths of approximately 10 kb, 20 kb, and 30 kb. Notably, Accuvir [15] exhibited a runtime exceeding 30 minutes and was therefore excluded from subsequent performance figures. As illustrated in Fig. 5, PVGA achieves runtime performance comparable to other mainstream assemblers, although it is not always the best in all scenarios.

Canu and Flye consistently demonstrate the longest runtimes. For Canu, this is primarily due to the computationally intensive processes of read error correction and graph simplification, while Flye’s substantial runtime stems from constructing and optimizing the De Bruijn assembly graph. As sequencing coverage increases, the runtime for nearly all assemblers grows proportionally. Interestingly, even with the iterative mechanism, PVGA exhibits a lower runtime compared to other assemblers like Canu and Flye. The efficiency gained through iteration primarily stems from the rapid convergence of the process. In the initial iteration, the generated result is already close to the ground truth, providing a more refined backbone for constructing the alignment graph in the next iteration. As the backbone becomes increasingly accurate, subsequent iterations achieve more precise alignments, and the dynamic programming algorithm further optimizes the assembly. This iterative refinement continues until the backbone and the assembled result become identical, ensuring that the assembly process reaches a stable and accurate configuration in a relatively short time.

**Figure 6:**
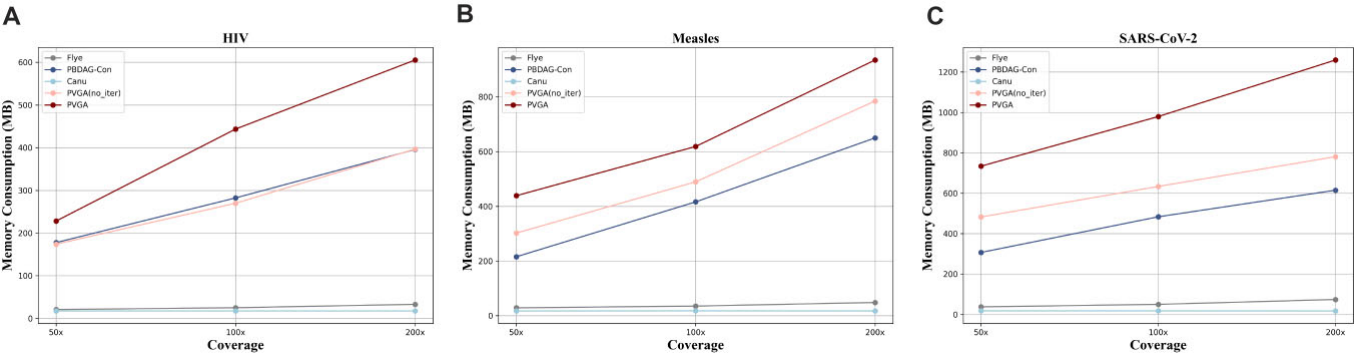
Comparison of maximum memory consumption during the runtime across 3 datasets with 50$\times$, 100$\times$, and 200$\times$ coverage: (A) HIV-1 89.6 strain, (B) measles virus, and (C) SARS-CoV-2.

In terms of memory consumption, we monitor the maximum memory usage during the assembly process across different assemblers. As shown in Figure 6, assemblers relying on alignment graph construction tend to require more memory such as PVGA and PBDAG-Con. This is because PVGA employs a more complex global graph-processing approach, which necessitates storing and manipulating a large amount of graph data, leading to higher memory consumption. In contrast, Canu and Flye typically break the input data into smaller chunks, which reduces memory usage and facilitates multithreaded optimization. However, given the relatively small size of viral genomes compared to those of other species, PVGA’s memory consumption remains within a reasonable range for viral genome assembly. All the computing resource experiments are conducted on an Apple M2 chip for evaluation.

## Conclusion

PVGA is a powerful virus-focused assembler that does both assembly and polishing. For virus genomes, small changes will lead to huge differences in terms of viral function and pathogenicity. Thus, for virus-focused assemblers, high-accuracy results are crucial. Our approach heavily depends on the input reads as evidence to produce the reported genome. It first adopts a reference genome to start with. We then align all the reads against the reference genome to get an alignment graph. After that, we use a dynamic programming algorithm to compute a path with the maximum weight of edges supported by reads. Most importantly, the obtained path is used as the new reference genome, and the process is repeated until no further improvement is possible.

The proposed framework demonstrates robust compatibility with diverse sequencing platforms, achieving nucleotide-level accuracy for both long-read (Nanopore/PacBio) and short-read (Illumina) data modalities. Experiments show that PVGA always outperforms popular existing programs in various cases. In particular, simulated Nanopore datasets show that our method can correctly report the true genomes with 0 mismatches and 0 indels.

## Availability of Source Code and Requirements

- Project name: PVGA
- Project homepage: https://github.com/SoSongzhi/PVGAWorkflow DOIs: 10.48546/workflowhub.workflow.1305.1 [35]biotoolsID: PVGA SciCrunch.org databases RRID: SCR_026410
- Operating system(s): Platform independent
- Programming language: Python
- Other requirements: numpy, pysam 0.22.0 or higher, pandas 1.5.2 or higher, Bio 1.7.1 or higher, biopython 1.83 or higher, consensus 1.0.5 or higher, networkx 3.1 or higher, pandas 1.5.2 or higher, python 3.10
- Biotools: QUAST 5.3.0, Badread 0.4.1
- License: MIT License

## Supplementary Material

giaf063_Supplemental_File

giaf063_Authors_Response_To_Reviewer_Comments_Original_Submission

giaf063_Authors_Response_To_Reviewer_Comments_Revision_1

giaf063_GIGA-D-25-00004_Original_Submission

giaf063_GIGA-D-25-00004_Revision_1

giaf063_GIGA-D-25-00004_Revision_2

giaf063_Reviewer_1_Report_Original_Submission Thomas Krannich -- 2/3/2025

giaf063_Reviewer_1_Report_Revision_1 Thomas Krannich -- 4/7/2025

giaf063_Reviewer_1_Report_Revision_2 Thomas Krannich -- 4/24/2025

giaf063_Reviewer_2_Report_Original_Submission Michael Roach -- 2/4/2025

giaf063_Reviewer_2_Report_Revision_1 Michael Roach -- 3/31/2025

## Data Availability

**HIV-1:** 89.6 strain complete genome is available in the NCBI database (GenBank: U39362.2). HXB2 strain complete genome is available in the NCBI database (GenBank: K03455.1). Haplotype benchmarking dataset contains mixed PacBio reads from 5 strains (HXB2, 89.6, JR-CSF, NL4-3, YU-2) [22]. **SARS-CoV-2:** SARS-CoV-2 isolate Wuhan-Hu-1 is available in the NCBI database (NCBI Reference Sequence: NC_045512.2). SARS-CoV-2 isolate INEI121916 is available in the NCBI database (GenBank: OZ072292.1). **Norovirus:** Nanopore sequencing reads are available in the NCBI database (Run: SRX10330013). Illumina sequencing reads are available in the NCBI database (Run: RR13951201, Run: SRR13951221, and Run: SRR1395119). The complete genome of Norovirus GII is available in the NCBI database (GenBank MW661264.1, MW661284.1, MW661248.1, and MW661250.1). **Ebola virus:** Ebola virus (EBOV-May) Mayinga strain complete genome is available in the NCBI database (GenBank: AF086833.2). Ebola virus/*M. fascicularis*-wt/GAB/2001/untreated-CCL053D9, complete genome is available in the NCBI database (GenBank: KY786027.1). **Measles virus:** Measles complete genome is available in the NCBI database (NCBI Reference Sequence: NC_001498.1). Measles virus genotype A transgenic strain vac2(GFP)H genome is available in the NCBI database (GenBank: MH144178.1).

## Additional Files

**Supplementary Table S1**. Results on simulated PacBio HIV-1 datasets with a 5% error rate and an average read length of 2 kb.

**Supplementary Table S2**. Results on simulated PacBio HIV-1 datasets with a 5% error rate and an average read length of 4 kb.

**Supplementary Table S3**. Results on simulated PacBio HIV-1 datasets with a 5% error rate and an average read length of 6 kb.

**Supplementary Table S4**. Results on simulated PacBio SARS-CoV-2 datasets with a 5% error rate and an average read length of 2 kb.

**Supplementary Table S5**. Results on simulated PacBio SARS-CoV-2 datasets with a 5% error rate and an average read length of 4 kb.

**Supplementary Table S6**. Results on simulated PacBio SARS-CoV-2 datasets with a 5% error rate and an average read length of 8 kb.

## Abbreviations

EBOV: Ebola virus; GP: glycoprotein; HGAP: hierarchical genome assembly process; NBCI: National Center for Biotechnology Information; NGS: next-generation sequencing; NP: nucleoprotein; SARS-CoV-2: severe acute respiratory syndrome coronavirus 2; SMRT: single molecule real time; TGS: third-generation sequencing.
