# Supplementary material for: PVGA: a precise viral genome assembler using an iterative alignment graph
Source: Gigascience. 2025 Jun 24;14:giaf063. doi: 10.1093/gigascience/giaf063 (PMC12206156; doi:10.1093/gigascience/giaf063)
Supplement: giaf063_Supplemental_File [file giaf063_supplemental_file.zip › supplementary.pdf]

SUPPLEMENTARY DOCUMENT

1 Benchmarking results on PacBio Datasets

Beyond testing on Nanopore data, we also test simulated PacBio data. Tables 1-3 present the results for HIV PacBio datasets, with average read lengths of 2kb, 4kb, and 6kb, respectively, while Tables 4-6 display the benchmarking results for SARS-CoV-2 at average read lengths of 2kb, 4kb, and 6kb, respectively. For both HIV and SARS-CoV-2 results, PVGA consistently demonstrates the lowest values across all conditions in terms of mismatches, indels, indel lengths, and edit distance, indicating that PVGA consistently outperforms other assemblers.

Although we set the same parameter "identity" in badread to simulate reads at a 5% error rate. the assembly result quality from nearly all assemblers decline when processing simulated PacBio reads compared to those from simulated Nanopore reads. To evaluate the simulated PacBio reads' accuracy, we align reads to the reference genome using minimap2 and calculate their average identity. The tested reads maintained the expected 95% identity (5% error rate). According to Badread's documentation, PacBio-like simulations inherently produce fewer perfect reads (0 mismatches/indels) than Nanopore-like simulations, though this does not alter the global identity parameter. This indicates that error-containing reads are more likely to occur, leading to increased alignment errors or misalignments during assembly.

Nevertheless, PVGA continues to outperform all other assemblers, consistently exhibiting the lowest numbers of indels, mismatches, and edit distances. For instance, as shown in Table 6, at coverage of 50x with an 8kb read length from SARS-CoV-2 datasets, other assemblers report indels, mismatches, and edit distances in double or even triple digits, whereas PVGA maintains single-digit values for each of these metrics.

Table 1: Results on simulated PacBio HIV-1 datasets with a 5% error rate and an average read length of 2kb

Reads depth	Tool	Genome fraction	Contig length	Mismatch	Indels	Indel length	Edit distance
50x	Flye	89.9	8669	0	63	63	1044
	Canu	93.452	12459	0	157	163	7848
	Accuvir	99.959	9678	1	45	49	54
	PBDAG-Con	99.969	9635	0	70	79	82
	Medaka	99.969	9698	9	30	48	60
	PVGA(noiter)	99.969	9703	0	9	9	12
	**PVGA**	**99.969**	**9703**	**0**	**9**	**9**	**12**
100x	Flye	99.938	9642	0	65	65	79
	Canu	99.732	9619	0	66	68	89
	Accuvir	99.959	9672	1	39	43	48
	PBDAG-Con	99.969	9644	0	60	72	75
	Medaka	99.969	9672	8	37	58	69
	PVGA(noiter)	99.969	9710	0	4	4	7
	**PVGA**	**99.969**	**9711**	**0**	**3**	**3**	**6**
200x	Flye	99.99	9635	0	77	77	78
	Canu	93.452	12966	0	131	138	6157
	Accuvir	99.959	9669	0	46	48	52
	PBDAG-Con	99.969	9633	0	69	81	84
	Medaka	99.969	9737	10	17	41	54
	PVGA(noiter)	99.969	9698	4	33	48	55
	**PVGA**	**99.969**	**9707**	**0**	**3**	**3**	**6**

Table 2: Results on simulated PacBio HIV-1 datasets with a 5% error rate and an average read length of 4kb

Reads depth	Tool	Genome fraction	Contig length	Mismatch	Indels	Indel length	Edit distance
50x	Flye	98.312	9490	0	59	59	1066
	Canu	99.979	24809	0	181	194	15224
	Accuvir	99.959	9675	0	43	44	48
	PBDAG-Con	99.969	9648	0	56	62	65
	Medaka	99.969	9687	8	26	48	59
	PVGA(noiter)	99.969	9704	0	10	10	13
	**PVGA**	**99.969**	**9704**	**0**	**10**	**10**	**13**
100x	Flye	100	9653	0	60	60	63
	Canu	100	18670	0	143	143	9093
	Accuvir	99.959	9679	1	34	34	39
	PBDAG-Con	99.969	9650	0	53	60	63
	Medaka	99.969	9705	0	8	8	8
	PVGA(noiter)	99.969	9731	7	23	47	57
	**PVGA**	**99.969**	**9710**	**0**	**4**	**4**	**7**
200x	Flye	99.99	9637	0	75	75	76
	Canu	99.99	9689	0	69	69	116
	Accuvir	99.959	9694	1	27	29	34
	PBDAG-Con	99.969	9637	0	64	73	76
	Medaka	99.969	9684	10	32	58	71
	PVGA(noiter)	99.969	9709	0	1	1	4
	**PVGA**	**99.969**	**9710**	**0**	**1**	**1**	**4**

Table 3: Results on simulated PacBio HIV-1 datasets with a 5% error rate and an average read length of 6kb

Reads depth	Tool	Genome fraction	Contig length	Mismatch	Indels	Indel length	Edit distance
50x	Flye	85.689	8266	0	57	57	1471
	Accuvir	99.959	9680	1	40	43	48
	PBDAG-Con	99.969	9657	0	49	59	62
	Medaka	99.969	9694	14	21	52	69
	PVGA(noiter)	99.969	9709	0	7	7	10
	**PVGA**	**99.969**	**9709**	**0**	**7**	**7**	**10**
100x	Flye	99.969	9650	0	63	63	69
	Canu	99.99	9691	0	61	61	102
	Accuvir	99.959	9675	0	52	60	64
	PBDAG-Con	99.969	9640	0	58	70	73
	Medaka	99.969	9690	19	28	61	83
	PVGA(noiter)	99.969	9710	0	2	2	5
	**PVGA**	**99.969**	**9710**	**0**	**2**	**2**	**5**
200x	Flye	99.568	10304	1	97	98	819
	Accuvir	99.959	9682	1	34	35	40
	PBDAG-Con	99.969	9646	0	60	64	67
	Medaka	99.969	9706	0	7	7	7
	PVGA(noiter)	99.969	9737	10	17	41	54
	**PVGA**	**99.969**	**9710**	**0**	**1**	**1**	**4**

Table 4: Results on simulated PacBio SARS-CoV-2 datasets with 5% error rate and an average read length of 2kb

Reads depth	Tool	Genome fraction	Contig length	Mismatch	Indels	Indel length	Edit distance
25x	Flye	97.46	28759	0	139	140	899
	Canu	98.644	29078	0	164	166	2548
	Accuvir	100	29784	1	97	101	236
	PBDAG-Con	100	29554	0	92	93	94
	Medaka	100	29850	0	22	30	254
	PVGA(noiter)	100	29652	0	20	20	20
	**PVGA**	**100**	**29652**	**0**	**20**	**20**	**20**
50x	Flye	99.997	29531	0	118	119	125
	Canu	99.906	29493	0	124	125	153
	Accuvir	100	29792	1	83	85	309
	PBDAG-Con	100	29593	0	78	79	105
	Medaka	100	29855	0	22	25	249
	PVGA(noiter)	100	29647	0	14	14	15
	**PVGA**	**100**	**29647**	**0**	**14**	**14**	**15**

Table 5: Results on simulated PacBio SARS-CoV-2 datasets with 5% error rate and an average read length of 4kb

Reads depth	Tool	Genome fraction	Contig length	Mismatch	Indels	Indel length	Edit distance
25x	Flye	99.993	29501	0	140	143	145
	Canu	99.811	29420	0	166	170	226
	Accuvir	100	29744	1	125	129	353
	PBDAG-Con	100	29552	0	92	95	96
	Medaka	100	29813	0	64	67	291
	PVGA(noiter)	100	29651	0	20	20	21
	**PVGA**	**100**	**29651**	**0**	**20**	**20**	**21**
50x	Flye	99.997	29556	0	93	94	100
	Canu	91.716	27098	0	90	92	2548
	Accuvir	100	29747	1	108	127	337
	PBDAG-Con	100	29579	0	67	68	69
	Medaka	100	29813	0	24	28	252
	PVGA(noiter)	100	29648	0	5	5	6
	**PVGA**	**100**	**29648**	**0**	**5**	**5**	**6**

Table 6: Results on simulated PacBio SARS-CoV-2 datasets with 5% error rate and an average read length of 8kb("-" indicates that the assembler fails to produce a result.)

Reads depth	Tool	Genome fraction	Contig length	Mismatch	Indels	Indel length	Edit distance
25x	Flye	99.997	29531	0	118	119	125
	Canu	99.906	29493	0	124	125	153
	Accuvir	100	29792	1	83	85	309
	Medaka	100	29609	0	43	45	270
	PBDAG-Con	100	29593	0	78	79	105
	PVGA(noiter)	100	29647	0	14	14	15
	**PVGA**	**100**	**29647**	**0**	**14**	**14**	**15**
50x	Flye	99.983	29572	0	92	93	114
	Canu	-	-	-	-	-	-
	Accuvir	100	29807	0	66	68	291
	Medaka	100	29609	0	23	25	270
	PBDAG-Con	100	29577	2	69	70	73
	PVGA(noiter)	100	29649	0	8	8	9
	**PVGA**	**100**	**29650**	**0**	**7**	**7**	**8**
