# Supplementary material for: PVGA: a precise viral genome assembler using an iterative alignment graph
Source: Gigascience. 2025 Jun 24;14:giaf063. doi: 10.1093/gigascience/giaf063 (PMC12206156; doi:10.1093/gigascience/giaf063)
Supplement: giaf063_GIGA-D-25-00004_Original_Submission [file giaf063_giga-d-25-00004_original_submission.pdf]

GigaScience

PVGA: A Precise Viral Genome Assembler Using Iterative Alignment Graph

--Manuscript Draft--

Manuscript Number:	GIGA-D-25-00004
Full Title:	PVGA: A Precise Viral Genome Assembler Using Iterative Alignment Graph
Article Type:	Research
Funding Information:	
Abstract:	Background: Viral genome analysis is crucial for understanding virus evolution and mutation. The studies of virus evaluation and mutation have attracted a lot of attention since the outbreak of COVID-19. As the basic structure of the virus genomes is highly conserved and RNA viruses have high mutation rates, small changes will lead to significant differences in terms of viral function and pathogenicity. Thus, special assembly methods are required for viral genome analysis.<br>Result: PVGA starts with a reference genome and utilizes the sequencing reads directly to reduce noise. The first step in PVGA involves constructing an alignment graph based on a reference genome and the set of input sequencing reads. We then use a dynamic programming algorithm to identify the path in the alignment graph supported by the highest number of reads. The obtained path corresponds to a refined genome. Finally, we repeat the process by using the new reference genomes until no further improvement is possible. We evaluate PVGA's performance across both assembly and polishing tasks using simulated and real datasets including both long reads and short reads. The experiments demonstrate that PVGA always outperforms the best existing programs in terms of the quality of assembly results, while the running time of our method is compatible to others. In particular, simulated Nanopore datasets show that our method can correctly report the true genomes with 0 mismatch and 0 indels. Conclusions: PVGA is a novel viral genome assembler that seamlessly integrates assembly and polishing into a unified workflow. Its design prioritizes high accuracy, enabling the detection of subtle genomic variations that can significantly impact viral function and pathogenicity. By addressing the unique challenges of viral genome assembly, PVGA provides a reliable and precise solution for advancing our understanding of viral evolution and behavior.
Corresponding Author:	LUSHENG WANG<br>City University of Hong Kong<br>Hong Kong, Kowloon HONG KONG
Corresponding Author Secondary Information:	
Corresponding Author's Institution:	City University of Hong Kong
Corresponding Author's Secondary Institution:	
First Author:	Zhi Song
First Author Secondary Information:	
Order of Authors:	Zhi Song
	Dehan Cai
	Yanni Sun
	LUSHENG WANG
Order of Authors Secondary Information:	
Additional Information:	
Question	Response
Are you submitting this manuscript to a special series or article collection?	No

Powered by Editorial Manager® and ProduXion Manager® from Aries Systems Corporation

**Experimental design and statistics**<br><br>Full details of the experimental design and statistical methods used should be given in the Methods section, as detailed in our Minimum Standards Reporting Checklist. Information essential to interpreting the data presented should be made available in the figure legends.<br><br>Have you included all the information requested in your manuscript?	Yes
**Resources**<br><br>A description of all resources used, including antibodies, cell lines, animals and software tools, with enough information to allow them to be uniquely identified, should be included in the Methods section. Authors are strongly encouraged to cite Research Resource Identifiers (RRIDs) for antibodies, model organisms and tools, where possible.<br><br>Have you included the information requested as detailed in our Minimum Standards Reporting Checklist?	Yes
**Availability of data and materials**<br><br>All datasets and code on which the conclusions of the paper rely must be either included in your submission or deposited in publicly available repositories (where available and ethically appropriate), referencing such data using a unique identifier in the references and in the “Availability of Data and Materials” section of your manuscript.<br><br>Have you have met the above requirement as detailed in our Minimum Standards Reporting Checklist?	Yes

Powered by Editorial Manager® and ProduXion Manager® from Aries Systems Corporation

GigaScience has policies and guidelines in place for the use of generative AI-writing tools such as ChatGPT. If you have used such writing tools to assist with writing the manuscript this must be declared and cited in the text. Authors should not list AI-writing tools and other AI-assisted technologies as an author or co-author and should acknowledge that they are fully responsible for text generated or refined by AI-writing tools.<p><br><br>A summary of use (particularly in the introduction or among methods) needs to be included at the end of the paper, and the outputs should also be included as a supplementary file hosted in GigaDB or other open repositories. Please read our guidelines for more information. <p><br><br>By submitting to GigaScience, you are aware of the journal's AI-writing tools policy, and if you have declared use of such tools below, you have acknowledged this where appropriate in your manuscript and have made a summary of use and outputs available. <p><br><b>AI-assisted writing tools have been used in the preparation of this manuscript?	No

main file
Click here to access/download;Manuscript;vmhkmgnjmmymkwsbywgpvstdd

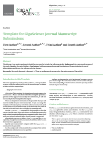

GigaScience, 2023, 1–11

doi: xx.xxxx/xxxx
Manuscript in Preparation
Paper

PAPER

PVGA: A Precise Viral Genome Assembler Using Iterative Alignment Graph

Zhi Song[1], Dehan Cai[2], Yanni Sun[2,*] and Lusheng Wang[1,*]

[1]Department of Computer Science, City University of Hong Kong and [2]Department of Electrical Engineering, City University of Hong Kong

*Correspondence address. Yanni Sun, Electrical Engineering, City University of Hong Kong, Kowloon, China. Email: yannisun@cityu.edu.hk; Lusheng Wang, Department of Computer Science, City University of Hong Kong, Kowloon, China. Email: cswangl@cityu.edu.hk

Abstract

Background: Viral genome analysis is crucial for understanding virus evolution and mutation. The studies of virus evaluation and mutation have attracted a lot of attention since the outbreak of COVID-19. As the basic structure of the virus genomes is highly conserved and RNA viruses have high mutation rates, small changes will lead to significant differences in terms of viral function and pathogenicity. Thus, special assembly methods are required for viral genome analysis.
Result: PVGA starts with a reference genome and utilizes the sequencing reads directly to reduce noise. The first step in PVGA involves constructing an alignment graph based on a reference genome and the set of input sequencing reads. We then use a dynamic programming algorithm to identify the path in the alignment graph supported by the highest number of reads. The obtained path corresponds to a refined genome. Finally, we repeat the process by using the new reference genomes until no further improvement is possible. We evaluate PVGA's performance across both assembly and polishing tasks using simulated and real datasets including both long reads and short reads. The experiments demonstrate that PVGA always outperforms the best existing programs in terms of the quality of assembly results, while the running time of our method is compatible to others. In particular, simulated Nanopore datasets show that our method can correctly report the true genomes with 0 mismatch and 0 indels.
Conclusions: PVGA is a novel viral genome assembler that seamlessly integrates assembly and polishing into a unified workflow. Its design prioritizes high accuracy, enabling the detection of subtle genomic variations that can significantly impact viral function and pathogenicity. By addressing the unique challenges of viral genome assembly, PVGA provides a reliable and precise solution for advancing our understanding of viral evolution and behavior.

Key words: Genome assembler; Virus genome; Alignment graph; Maximum total weight path; Iterative method

Introduction

Viral genome analysis is crucial for understanding virus evolution and mutation. The studies of virus evaluation and mutation have attracted lots of attention since the outbreak of COVID-19. The virus genomes have relatively small size. As the basic structure of the virus genomes is highly conserved and RNA viruses have high mutation rates, small changes in genomes will lead to significant differences in terms of viral function and pathogenicity. For example, several mutations in the spike protein gene of coronavirus can alter its ability to enter host cells, thereby affecting transmissibility and disease severity [1]. Thus, special assembly methods are required for viral genome analysis.

Sequencing of viral genomes primarily relies on Next-Generation Sequencing (NGS) and Third-Generation Sequencing (TGS) technologies. NGS is recognized for its short read lengths and high accuracy, with platforms such as Illumina. However, due to the short length of reads, it is well-known that NGS struggles with assembling repetitive regions in the genome.

Third-generation sequencing (TGS) technologies, such as Nanopore and PacBio, are effective for resolving complex genome structures and repetitive regions due to their longer read lengths. However, these longer reads tend to have higher error rates. For instance, Nanopore sequencing relies on measuring electrical current

Compiled on: January 5, 2025.
Draft manuscript prepared by the author.

changes as DNA passes through a nanopore, and factors such as pore condition, molecule speed, and signal noise can interfere with base-calling accuracy, making it difficult to distinguish adjacent bases, thereby reducing the quality of the reads [2]. PacBio sequencing, using its Single Molecule Real-Time (SMRT) technology [3], is also widely applied for TGS. SMRT sequencing is well-suited for detecting structural variations and resolving repetitive regions, but it frequently introduces insertion and deletion (indel) errors. To improve accuracy, PacBio introduced HiFi(high-fidelity) sequencing, which produces highly accurate long reads by repeatedly sequencing the same molecule [4]. However, this increased accuracy comes at a higher cost. Achieving high-quality viral genome assembly requires balancing sequencing accuracy with cost. Both the choice of sequencing technology and the assembly algorithms play critical roles in producing reliable genome assemblies.

Genome assembly techniques are broadly categorized into two types: De Novo assembly and reference-guided assembly. De Novo assembly tools include Velvet [5], ABySS [6], SPAdes [7], Flye [8], Canu [9]and Translig [10], which reconstructs the genome without relying on a reference genome. However, De Novo methods often encounter challenges in highly repetitive regions, which may lead to misassemblies, redundant contigs, or gaps. Moreover, in regions of low coverage, De Novo assembly may produce incomplete or missing sequences, and further introduce gaps and errors. With the help of the reference genome sequence, one can obtain the locations of reads in the genome. Thus, reference-guided assembly methods can possibly fill gaps between reads and improve prediction accuracy in low-coverage regions. Famous reference-guided assembly methods include BWA [11], Bowtie2 [12], GATK [13], Novoalign [14], and Maq [15].

There are some assemblers that are optimized for viruses. For instance, IVA was developed as a De Novo assembler for RNA viruses, utilizing paired-end datasets to achieve more accurate assemblies [16]. Similarly, Accuvir introduced a reference-based long-read assembler for viruses, primarily employing diverse beam search algorithms on alignment graphs to improve accuracy [17]. In addition to assembly tools, genome polishing methods have become increasingly important for enhancing assembly accuracy by correcting errors using high-accuracy reads. Pilon improves genome assemblies by analyzing read alignments, constructing a pileup structure to evaluate base-level evidence, and iteratively adjusting the assembly based on read quality and consistency [18]. NextPolish employs a combination of alignment-based error correction and iterative consensus polishing, utilizing short-read data to precisely correct mismatches and indels, further refining the final genome assembly [19].

In this paper, we present PVGA, a novel viral genome assembler that can perform both assembly and polishing, effectively handling both long-read and short-read sequencing data. PVGA starts with a reference genome and utilizes the sequencing reads directly to reduce noise. The first step in PVGA involves constructing an alignment graph based on a reference genome and the set of input sequencing reads. We then use a dynamic programming algorithm to identify the path in the alignment graph supported by the highest number of reads. The obtained path corresponds to a refined genome. Finally, we repeat the process by using the new reference genomes until no further improvement is possible.

We evaluated PVGA's performance across both assembly and polishing tasks using simulated and real datasets including both long reads and short reads. The results demonstrate that PVGA consistently outperforms the best existing programs. In particular, simulated Nanopore datasets show that our method can correctly report the true genomes with 0 mismatch and 0 indels, except for some small errors at the two ends of the genomes.

Methods

Our new method contains three steps. Step 1: We construct an alignment graph based on the set of input reads using an initial reference genome as the backbone. The initial reference genome should be from the same species. One can also generate an initial reference genome using an existing De Novo assembler. Step 2: After constructing the alignment graph, we apply a dynamic programming algorithm to select a path supported by the largest number of read coverage and construct a new reference genome based on the path. Step 3: We then use the latest reference genome as the backbone to repeat Steps 1-2. The process stops when the new reference genome is identical to the old one.

Alignment graph construction

The graph construction method is inspired by the hierarchical genome-assembly process (HGAP) proposed by Chin [20]. The input contains two parts: read sequences and a backbone sequence. First, we construct the initial graph G_b with n nodes $v_1, v_2, \ldots v_n$ and $n-1$ edges based on the backbone sequence $S = s_1 s_2 \ldots s_n$, where each node v_i is labeled with the letter s_i and there is an edge (v_i, v_{i+1}) connecting the two consecutive nodes. We then align each read $R = r_1 r_2 \ldots r_k$ with the reference sequence G_b. Such an alignment corresponds to a path with k nodes and $k-1$ edges, where each node is labeled with r_i.

If r_i is matched with an identical letter s_j in the alignment, then r_i corresponds to the node in v_j in G_b. Otherwise, we will create a new node with label r_i. Finally, we add an edge to connect the two nodes labeled with r_i and r_{i+1}. See Fig. 1(b). we will repeat the above process until all reads have been applied. The obtained intermediate graph is denoted as $G_I = (V, E)$. In the intermediate graph G_I, each edge is assigned a weight of 1, representing the number of supporting reads . To reduce the complexity of G_I, we merge nodes with the same label and the same parent repeatedly. The weight of the remaining edge (v, u') is updated to be the number of supporting reads. Finally, if there are multiple edges between any two nodes u and v in G_I, we combine them into a single edge and update the weight accordingly. This process results in a simplified final alignment graph G.

Finding a directed path in the alignment graph with maximum total weight

The weight on each edge is the number of supporting reads. In order to find a new reference sequence, we will try to find a path in G containing the maximum total weight. Such a path is the path supported by the largest number of reads.

We do a topological sorting on the set of nodes in G and obtain a linear order among the set of nodes in G. Let DP[v] denote the maximum total weight of paths ending at node v. The value of DP[v] can be computed as follows.

$$DP[u] = \max_{v \in Pred(u)} \{DP[v] + w(v, u)\}, \quad (1)$$

where $w(v, u)$ is the weight of the edge from node v to node u. We can compute all the DP[v]s according to the topological order. After that, we will find a node v with the largest DP[v] value and use a standard backtracking process to get a path with maximum total weight on G ending at v.

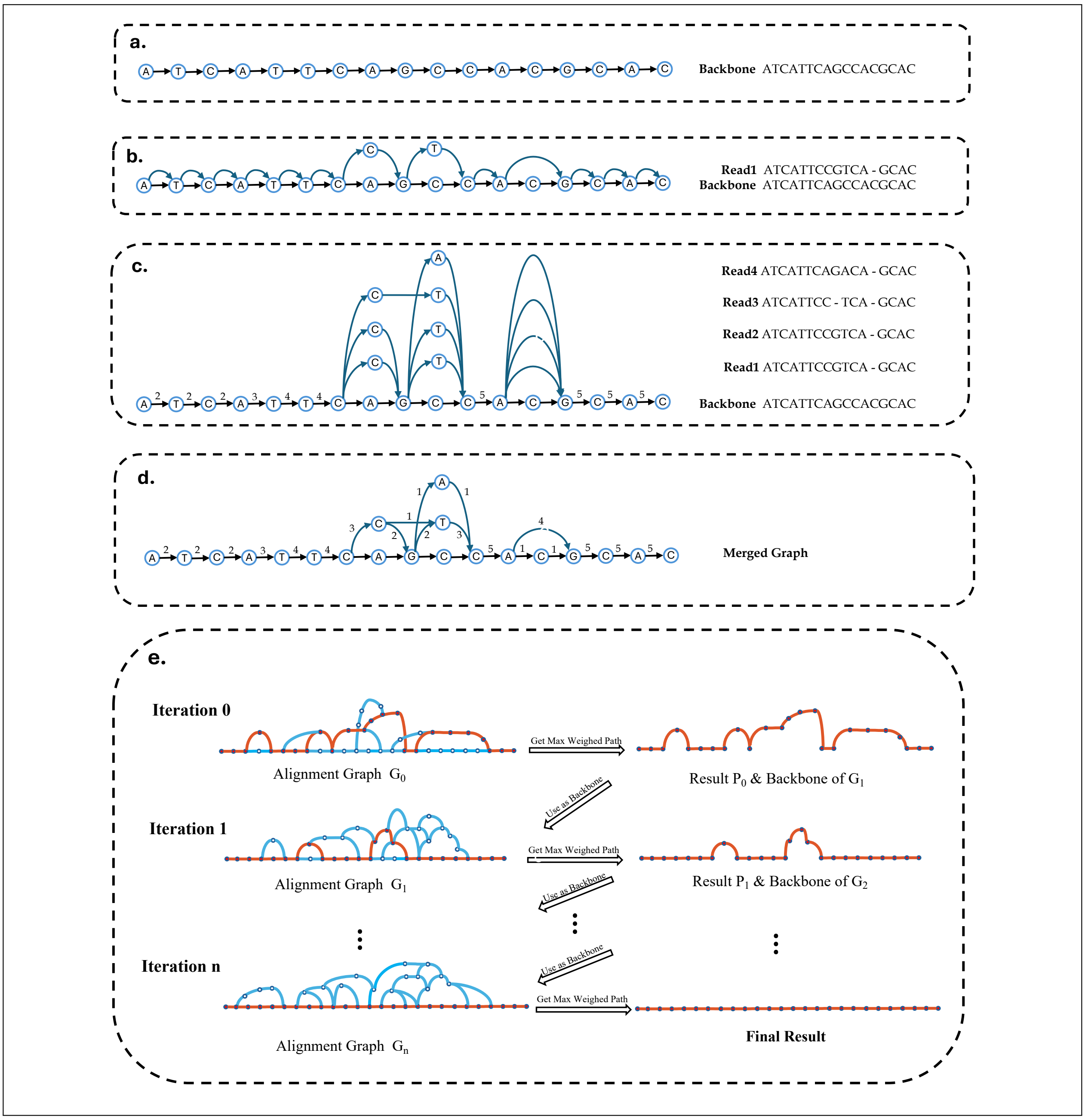

Figure 1. Flowchart of construction of alignment graph and iteration process. **a** PVGA takes a reference Genome as backbone graph G_B.
b PVGA aligns the first read Read1 to the backbone.
c Four reads are aligned with the backbone, awaiting the subsequent merging process.
d PVGA merges edges that point to the same node; the new edge's weight is equal to the sum of the weights of the merged edges. This process can be performed either after aligning all reads or during the alignment process, with a final merge conducted after all reads have been aligned.
e Iteratively construct the alignment graph using the result from the previous iteration as the backbone.

Running time: To compute each $DP[u]$ in equation (1), we need $O(Deg(u))$ time, where $Deg(u)$ is the in-degree of u. Therefore, the total running time complexity of this dynamic programming algorithm is $O(|E|)$, where $|E|$ represents the total number of edges in the graph.

Updating the reference genome iteratively

The quality of the obtained maximum weight path heavily depends on the initial reference genome, as errors or biases in the initial reference can impact the alignment and subsequent path computation. To address this, we use the obtained maximum weight path as a new reference genome. A new alignment graph is then reconstructed using all the input reads and the updated reference genome. The maximum weight path is computed again based on this updated graph. This process is repeated iteratively to refine the assembly. With each iteration, the alignment graph and the resulting path become more accurate and consistent. Iteration stops when the genome obtained from the current iteration is identical to the genome from the previous iteration. This indicates that the backbone and the assembly result have reached a consistent state and no further adjustment is possible using this method. This condition for algorithm termination seems to be very strong and one may worry about the running time of this condition.

Recall that PVGA is for virus genome assembly and the genome size is relatively small. Experimental results indicate that our method exhibits rapid convergence, often requiring no more than

3 or 4 iterations to reach a stable solution in practical applications. The running time comparison is in Figure 4. Moreover, this iterative method can reach high quality results and experiments show that our iterative method always outperforms the state-of-art methods.

Results

Datasets

We evaluate PVGA using both simulated and real viral sequencing data. To simulate a broad spectrum of sequencing conditions, we use Badread [21], which is designed for generating various kinds of simulated genomes. All real genome data and real sequencing reads used in this study are sourced from the National Center for Biotechnology Information (NCBI). These datasets include the following viral strains.

HIV:
HIV-specific data provides a critical benchmark for evaluating assembly methods, given its widespread availability in public databases. We use the 89.6 strain (GenBank: U39362.2) as the target genome, and generate simulated reads in bulk, with sizes detailed in the next subsection, to serve as the ground truth. The HXB2 strain (GenBank: K03455.1) is selected as the reference genome for constructing the initial alignment graph, which shares 93.88% similarity with the ground truth 89.6 strain.

The haplotype benchmarking dataset [22] contains mixed PacBio reads from five strains (HXB2, 89.6, JR-CSF, NL4-3, YU-2). To obtain reads from a single strain, we first align the reads to the genomes of these strains using minimap2 [23]. We then extract the reads based on their closest aligned genome (identified by samtools [24]).

SARS-CoV-2:
SARS-CoV-2 sequencing data is characterized by extensive genomic length and high sequence homology. We select SARS-Cov-2 isolate Wuhan-Hu-1 (NCBI Reference Sequence: NC_045512.2) as the reference genome and SARS-Cov-2 isolate (GenBank: OZ072292.1) as a target to generate the simulation reads.

Norovirus:
Noroviruses are common pathogens that can cause acute gastroenteritis. We obtain third-generation nanopore sequencing data of noroviruses(SRX10330013) from the National Food Virology Reference Centre at Health Canada [25]. This dataset consists of 5,741 spots, totaling 2.9 million bases, with the norovirus GII strain BMH19-097 serving as the ground truth genome. We use the complete genome of Norovirus GII (NC_039477.1) as the reference for graph construction. We also employ actual Illumina sequencing data of the norovirus. We utilize SRR13951201 (35.8M bases), SRR13951221 (60M bases), and SRR13951199(12.9M bases) as input reads, with corresponding ground truth data from Norovirus GII isolates BMH19-145, BMH13-039 and BMH14-056.

Ebola:
We utilize two Ebola virus (EBOV) genomes. The first genome corresponds to the Ebola virus (EBOV-May) Mayinga strain, isolated in Zarie in 1976. This genome consists of 18,959 base pairs and is publicly available under the NCBI accession number AF086833.2. EBOV-May is a negative-sense, single-stranded RNA virus of the genus Orthoebolavirus, encoding seven structural proteins such as nucleoprotein (NP), glycoprotein (GP), and RNA-dependent RNA polymerase (L) [26, 27]. The second Ebola virus strain was isolated from Macaca fascicularis and sequenced using IonTorrent technology. The genome, consisting of 18,871 base pairs, is publicly accessible under the NCBI accession number KY786027.1. It was assembled using the CLC Genomics Workbench v9.5.4 and serves as the ground truth genome for generating synthetic reads in this study. The metadata associated with this genome are part of BioProject PRJNA379115 and BioSample SAMN06603499, with additional information submitted by Guedj et al. [28]

Measles:
Measles virus (MV), a member of the genus Morbillivirus in the family Paramyxoviridae, is a highly contagious, negative-sense, single-stranded RNA virus. We utilize two Measles virus strain. The first genome, a complete reference genome of Measles morbillivirus, was sourced from the NCBI RefSeq database (accession number NC_001498.1). This genome consists of 15,894 base pairs. The second genome is the Measles virus genotype A transgenic strain vac2(GFP)H and serves as the ground truth genome for generating synthetic reads. This genome, available under the GenBank accession number MH144178.1, spans 16,728 base pairs and includes a transgenic insertion of the green fluorescent protein (GFP) gene. It was sequenced using Sanger dideoxy sequencing and has been used in experimental studies for functional and structural analyses [29, 30].

Evalution on simulation data

We evaluate the performance of several assemblers using simulated Nanopore and PacBio data. Tests are conducted on two viral strains, HIV and SARS-CoV-2, under both standard and low coverage conditions to assess their robustness.

We compare our method PVGA with the state-of-the-art methods, Flye [8], Canu [9], Accuvir [17], PBDAG-Con [20] and Medaka [31]. In addition, to illustrate the improvement achieved by the iterative step of PVGA, we also present the results of our method without the iterative step (referred to as PVGA (no_iter))

We evaluate the assembly quality using several metrics, including genome fraction, mismatches, indels, indel length, and edit distance. Genome fraction represents the percentage of reference genome bases accurately matched by the assembled genome. Mismatches refer to the number of positions where the nucleotide in the assembly differs from the reference sequence. Indels refer to the number of insertions and deletions in the assembly relative to the reference. Indel length refers to the total length of the indels. Edit distance indicates a minimum number of operations required to transform the assembled genome into the reference sequence. These metrics collectively measure the assembly's accuracy and its deviation from the ground truth genome.

Benchmarking on Standard-Length and Depth HIV Simulated Data
As the genome length increases, all methods tend to show higher error rates. However, PVGA consistently outperforms the compared assemblers. For our simulations, the average lengths of reads for Nanopore and PacBio were set at 2k, 4k, and 6k, with sequencing depths of 50x, 100x, and 200x.

To simulate real sequencing data, we used the Badread tool with the error model parameter set to nanopore2023, which was trained on ONT R10.4.1 reads. The identity was set to (95, 99, 2.5), indicating a normal distribution with a mean of 95 and a standard deviation of 2.5. For PacBio sequencing, we set the error model in Badread to pacbio2021, trained on PacBio Sequel II HiFi reads. We use the same identity settings as (95, 99, 2.5). The results for Nanopore are presented in Figure 2 and Table 1, while the results for PacBio are shown in the supplementary materials.

From Table 1, we can see that PVGA achieves 0 mismatches and 0 indels with an edit distance of 3 across all Nanopore test cases. Figure 2, illustrates similar cases, where the average lengths of reads are 2kb and 4kb, respectively.

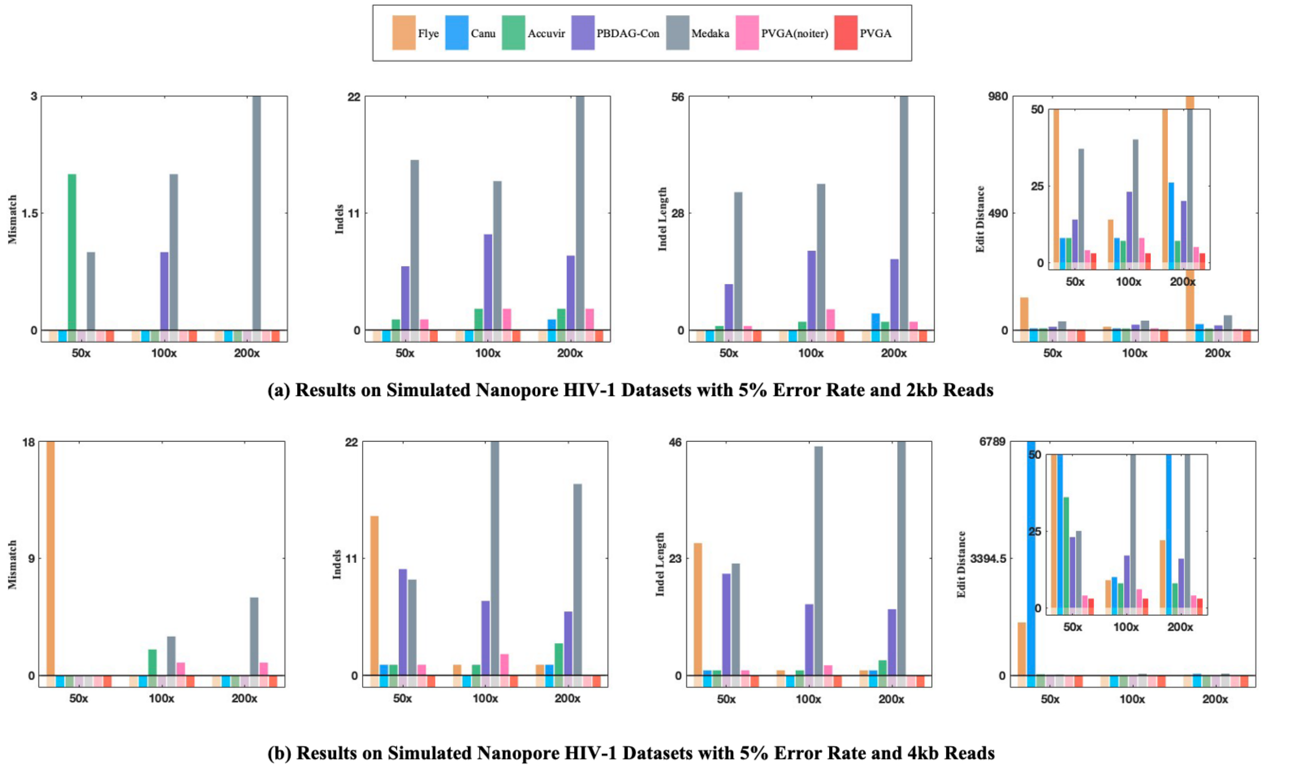

Figure 2. Results on simulated Nanopore HIV-1 datasets with an average read length of 2kb and 4kb, respectively. The 4 sub-figures in each row represent mismatch, indels, indel length, and edit distance from left to right, respectively.

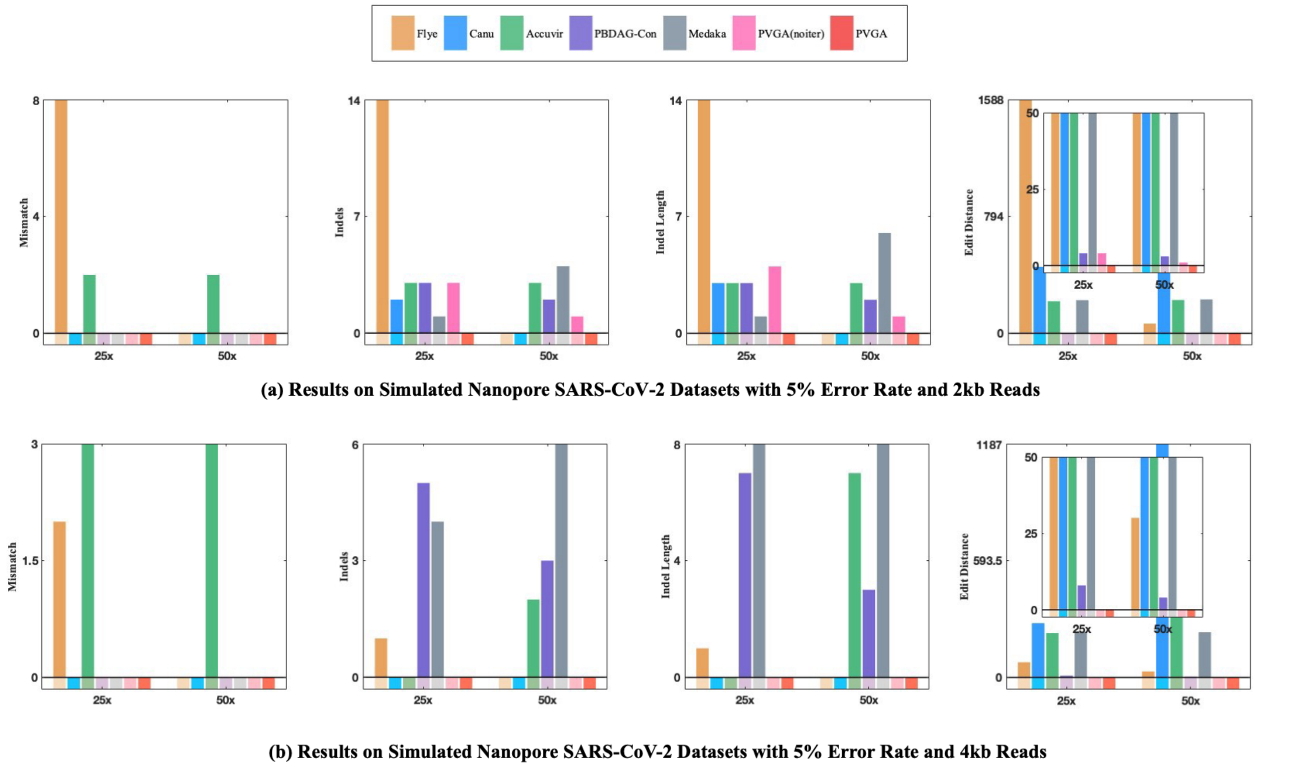

Figure 3. Results on simulated Nanopore SARS-CoV-2 datasets with an average read length of 2kb and 4kb, respectively. The 4 sub-figures in each row represent mismatch, indels, indel length, and edit distance from left to right, respectively.

Table 1. Results on Simulated Nanopore HIV-1 (length: 9713bp) Datasets with 5% Error Rate and an average read length of 6kb ("-" indicates that the assembler fails to produce a result.)

Reads depth	Tool	Genome fraction	Genome length	Mismatch	Indels	Indel length	Edit distance
50x	Flye	93.143	9067	0	0	0	686
	Canu	100	9748	0	0	0	35
	Accuvir	99.949	9709	0	2	3	4
	PBDAG-Con	99.969	9693	1	7	17	20
	medaka	99.969	9724	2	12	30	35
	PVGA (no_iter)	99.969	9706	0	2	4	7
	**PVGA**	**99.969**	**9710**	**0**	**0**	**0**	**3**
100x	Flye	96.757	9405	0	0	0	322
	Canu	-	-	-	-	-	-
	Accuvir	99.949	9706	0	2	2	7
	PBDAG-Con	99.969	9693	0	8	17	20
	medaka	99.969	9728	1	22	46	50
	PVGA (no_iter)	99.969	9709	2	1	1	6
	**PVGA**	**99.969**	**9710**	**0**	**0**	**0**	**3**
200x	Flye	100	9734	0	1	1	23
	Canu	-	-	-	-	-	-
	Accuvir	99.959	9710	0	1	1	5
	PBDAG-Con	99.969	9696	0	6	14	17
	medaka	99.969	9732	7	26	68	77
	PVGA (no_iter)	99.969	9705	1	5	5	9
	**PVGA**	**99.969**	**9710**	**0**	**0**	**0**	**3**

Canu could not construct the assembly graph required for genome assembly, when the reads dataset lacks sufficient independent reads and effective overlaps.

The PacBio results are illustrated in the supplementary document Section. As shown in Tables 1 as well as Figures 2, the iterative refinement process significantly enhances assembly accuracy. For instance, with 6k read lengths and a depth of 200x, the process reduces indels from 5 to 0, mismatches from 1 to 0, and the edit distance from 9 to 3. The experiments show that increasing coverage improves performance for some assemblers. For PVGA, a coverage of 50x is sufficient to achieve 0 mismatches and 0 indels.

Benchmarking on Low Coverage HIV Simulated Data

Sequencing costs increase with coverage. An efficient assembler should perform well not only for high coverage but also under lower coverage conditions to support cost-effective sequencing. Thus, we further test the cases, where the coverage are 30x, 25x, and 20x, respectively, with an average read length of 2kb. See Table 2.

Table 2. Results on simulated Nanopore HIV-1 (length: 9713bp) datasets with low coverage: 30x, 25x and 20x

Reads depth	Tool	Genome fraction	Genome length	Mismatch	Indels	Indel length	Edit distance
30x	Flye	70.751	6917	0	1	1	2888
	Canu	93.452	11602	0	1	1	4147
	Accuvir	99.938	9707	1	3	4	11
	PBDAG-Con	99.969	9695	0	7	15	18
	Medaka	99.969	9714	1	9	18	22
	PVGA (no_iter)	99.969	9706	0	1	4	7
	**PVGA**	**99.969**	**9710**	**0**	**0**	**0**	**3**
25x	Flye	77.906	7576	0	0	0	2155
	Canu	93.452	11156	0	1	1	7325
	Accuvir	99.959	9713	3	3	4	11
	PBDAG-Con	99.969	9688	0	8	22	25
	Medaka	99.969	9711	2	8	17	22
	PVGA (no_iter)	99.969	9709	0	1	1	4
	**PVGA**	**99.969**	**9710**	**0**	**0**	**0**	**3**
20x	Flye	96.788	9440	0	1	1	353
	Canu	93.452	11549	0	4	5	5918
	Accuvir	99.949	9708	2	2	2	9
	PBDAG-Con	99.969	9688	0	13	24	27
	Medaka	99.969	9714	0	10	20	23
	PVGA (no_iter)	99.969	9710	0	0	0	3
	**PVGA**	**99.969**	**9710**	**0**	**0**	**0**	**3**

At these lower coverage levels, PVGA continues to show robust performance. As shown in Tables 2, at coverage levels of 30x, 25x, and 20x, while some assemblers experience a noticeable drop in accuracy, PVGA consistently achieves the best performance with 0 mismatches and 0 indels. To figure out the threshold at which the accuracy of PVGA begins to decline, we test the case, where the coverage ranges from 19x down to 15x with an average read length of 2kb. The results are shown in Table 3.

As shown in Table 3, at coverages of 19x, 18x, and 17x, PVGA maintains zero indels. When the coverage drops to 16x, PVGA has its first indel with a length of 1, increasing to 4 at 15x. Despite this, PVGA continues to outperform all other assemblers, with the lowest indel count, indel length, mismatch rate, and edit distance. These results demonstrate PVGA's robustness under low coverage conditions, highlighting its potential to significantly reduce sequencing costs while maintaining accuracy.

Table 3. Results on simulated Nanopore HIV-1 (length: 9713bp) datasets with low coverage (<20x)

Reads depth	Tool	Genome fraction	Genome length	Mismatch	Indels	Indel length	Edit distance
19x	Flye	88.963	8673	0	0	0	1104
	Canu	93.452	11853	0	5	6	5044
	Accuvir	99.866	9709	0	1	1	8
	PBDAG-Con	99.969	9682	1	11	28	32
	Medaka	99.969	9707	3	11	17	23
	PVGA (no_iter)	99.969	9707	0	1	3	6
	**PVGA**	**99.969**	**9710**	**0**	**0**	**0**	**3**
18x	Flye	93.926	9140	0	3	3	613
	Canu	99.969	9708	0	2	2	5
	Accuvir	99.959	9711	4	5	6	14
	PBDAG-Con	99.969	9686	0	11	24	27
	Medaka	99.969	9707	1	8	14	18
	PVGA (no_iter)	99.969	9709	0	1	1	4
	**PVGA**	**99.969**	**9710**	**0**	**0**	**0**	**3**
17x	Flye	72.614	7085	0	2	2	2694
	Canu	99.866	10907	0	5	5	1226
	Accuvir	99.753	9713	1	1	2	12
	PBDAG-Con	99.969	9685	0	8	25	28
	Medaka	99.969	9711	0	7	23	26
	PVGA (no_iter)	99.969	9710	0	0	0	3
	**PVGA**	**99.969**	**9710**	**0**	**0**	**0**	**3**
16x	Flye	93.092	9047	0	3	3	682
	Canu	93.452	11805	0	3	3	4126
	Accuvir	99.959	9719	0	7	10	14
	PBDAG-Con	99.969	9696	1	6	14	18
	Medaka	99.969	9707	0	1	3	7
	PVGA (no_iter)	99.969	9710	0	2	2	5
	**PVGA**	**99.969**	**9711**	**0**	**1**	**1**	**4**
15x	Flye	96.87	9402	0	7	7	315
	Canu	94.399	9163	1	6	6	551
	Accuvir	99.856	9693	0	7	8	22
	PBDAG-Con	99.969	9687	2	12	27	32
	Medaka	99.969	9703	3	6	13	19
	PVGA (no_iter)	99.969	9706	0	4	4	7
	**PVGA**	**99.969**	**9706**	**0**	**4**	**4**	**7**

Table 4. Results on simulated Nanopore SARS-CoV-2 (length: 29646bp) datasets with 5% error rate and an average read length of 8kb

Reads depth	Tool	Genome fraction	Genome length	Mismatch	Indels	Indel length	Edit distance
25x	Flye	100	29656	0	0	0	10
	Canu	96.711	28669	0	6	11	977
	Accuvir	100	29872	0	3	3	226
	PBDAG-Con	100	29646	0	14	1	2
	Medaka	100	29878	0	2	8	232
	PVGA (no_iter)	100	29644	0	4	4	4
	**PVGA**	**100**	**29646**	**0**	**0**	**0**	**0**
50x	Flye	99.98	29645	0	0	0	11
	Canu	97.834	28999	0	2	5	647
	Accuvir	100	29869	0	3	3	223
	PBDAG-Con	100	29672	0	0	0	26
	Medaka	100	29646	0	5	11	235
	PVGA (no_iter)	100	29643	0	0	0	3
	**PVGA**	**100**	**29646**	**0**	**0**	**0**	**0**

Benchmarking on Simulated SARS-CoV-2 Data

The SARS-CoV-2 virus is one of the RNA viruses with a long genome, approximately 29.9 kb. Although SARS-CoV-2 variants are highly similar, a few differences can lead to distinct biological properties such as pathogenicity, transmissibility, and immune response. Therefore, assembling an accurate SARS-CoV-2 genome is essential. In this section, we conduct experiments on simulated SARS-CoV-2

data at different depths (25x and 50x) and read lengths (2kb, 4kb, and 8kb) for both Nanopore and PacBio datasets to evaluate the performance of tools on a highly similar virus with a long genome. Results for Nanopore are presented in Table 4 and Figure 3, while PacBio results are included in the supplementary document.

Table 4 shows that both PVGA and Flye achieve zero indels and mismatches. However, PVGA demonstrates superior performance, reconstructing the genome flawlessly, with no errors even at the two ends of the genome.

Evaluation on poor sequencing conditions

A study by the MinION Analysis and Reference Consortium reported that the median total error of all 2D reads was 12%, with 2D pass reads showing a slightly lower error rate of 10.5% [32]. Additionally, after basecalling, the global error rate of raw reads is typically around 10% [33].

To reflect poor sequencing conditions, we set the reads' average error rate to 10% and use a sequencing depth of 30x and an average read length of 4kb on HIV, Measles and Ebola virus respectively, to evaluate the assembler's performance under suboptimal data quality.

Table 5. Results on simulated Nanopore HIV-1, Measles, Ebola virus datasets with 10% error rate and an average read length of 4kb with an average depth of 30x

Virus	Tool	Genome fraction (%)	Genome length	Mismatch	Indels	Indel length	Edit distance
HIV (Length: 9713bp)	Flye	100	9732	0	16	17	53
	Canu	99.053	16468	0	36	43	6993
	Accuvir	99.835	9683	0	14	14	30
	PBDAG-Con	99.969	9645	1	26	67	71
	PVGA (no_iter)	99.969	9720	1	26	38	42
	**PVGA**	**99.969**	**9717**	**0**	**7**	**11**	**14**
Measles (Length: 16728bp)	Flye	99.988	13899	0	17	20	2829
	Canu	99.815	16674	2	21	23	56
	Accuvir	99.994	16725	4	9	12	17
	PBDAG-Con	94.996	15879	1	10	12	850
	PVGA (no_iter)	100	16733	2	12	17	19
	**PVGA**	**100**	**16734**	**1**	**5**	**8**	**9**
Ebola (Length: 18871bp)	Flye	99.989	18851	1	18	18	21
	Canu	99.862	18824	1	16	21	48
	Accuvir	100	18979	9	26	28	123
	PBDAG-Con	100	18858	0	13	13	13
	PVGA (no_iter)	100	18874	0	12	13	13
	**PVGA**	**100**	**18879**	**0**	**7**	**8**	**8**

For HIV, the 89.6 strain is used as the target to simulate reads, with the HXB2 strain serving as the backbone. For measles virus, the NC_001498.1 sequence (15,894 bp) is used as the backbone, while the vac2(GFP)H sequence (Length: 16,728 bp) served as the target. For Ebola virus, the EBOV-May strain (18,959 bp) is used as the backbone, with the KY786027 strain serving as the ground truth for read simulation.

Evaluation on real data

Although there are many available long-read sequencing datasets of viruses, most of them lack ground-truth genomes for validation. Thus, we use Norovirus and HIV to evaluate the tools' performance, as they have ground-truth genomes from both long-read and short-read sequencing data.

For the HIV real datasets, we collect PacBio sequencing data from a mock HIV-1 community. To create the datasets for viral genome reconstruction, we separate this dataset into read sets from five HIV strains, by aligning them to the ground-truth genomes using the best hit. We test the 89.6, JR-CSF, and YU-2 subtypes, using HXB2 as the backbone to construct the alignment graph. Given that real data often contains gaps between reads, most De Novo assemblers fail to achieve consensus or produce only very short contigs. As shown in Table 5, as for the 89.6 Strain, Canu produces a contig of only 4,593 base pairs. Flye, on the other hand, encounters errors during real data processing, resulting in an unsuccessful assembly. Due to the lower quality of real reads, assemblers display higher mismatches, indels, and edit distances in the HIV 89.6 strain than observed in simulations. However, PVGA still outperforms all other assemblers. In the JR-CSF results, apart from Canu, which failed to assemble a complete genome, only PVGA and PBDAG-Con maintained single-digit mismatches, with PVGA showing lower indels, indel length, and edit distance.

Table 6. Results on real HIV strain datasets(89.6, JR-CSF, YU-2)

Strain	Tool	Genome fraction	Genome length	Mismatch	Indels	Indel length	Edit distance
89.6 Strain (Length: 9713bp)	Canu	47.287	4593	6	0	0	5127
	Accuvir	99.990	9710	33	4	4	30
	Medaka	99.856	9733	25	6	22	74
	PBDAG-Con	100	9711	24	2	2	40
	PVGA (no_iter)	100	9709	25	2	2	36
	**PVGA**	**100**	**9710**	**24**	**1**	**1**	**28**
JR-CSF (Length: 9535bp)	Canu	88.512	8448	6	1	1	1320
	Accuvir	99.727	9736	36	3	3	241
	PBDAG-Con	99.99	9720	5	8	20	221
	Medaka	97.735	9535	33	8	42	316
	PVGA (no_iter)	99.99	9610	3	1	1	159
	**PVGA**	**99.99**	**9610**	**3**	**1**	**1**	**159**
YU-2 (Length: 9706bp)	Canu	86.750	8473	5	2	4	1343
	Accuvir	99.727	9713	5	5	9	16
	PBDAG-Con	100.000	9705	4	7	19	23
	Medaka	100.000	9698	28	9	52	80
	PVGA (no_iter)	100.000	9615	3	9	13	16
	**PVGA**	**100.000**	**9617**	**3**	**7**	**11**	**14**

We also test our method on real norovirus data(SRX10330013). As shown in Table 7, our method PVGA results in the fewest mismatches, indels, and the lowest edit distance among the five assemblers evaluated. This demonstrates that our PVGA method more effectively utilizes information from the alignment graph compared to PBDAG-Con, which focuses on assigning scores to nodes to maximize consensus, and Accuvir, which employs diverse beam search. While diverse beam search approach increases the diversity of candidate paths, it often falls into local optima, failing to achieve the best results.

Table 7. Results on real Nanopore noroviruses (SRX10330013), with ground truth genome as Norovirus GII isolate BMH19-097 (Length: 7618 bp)

Tool	Genome fraction	Genome length	Mismatch	Indels	Indel length	Edit distance
Flye	92.964	7097	0	11	13	577
Canu	99.593	7632	0	12	14	104
Accuvir	99.396	7564	1	8	10	57
PBDAG-Con	99.383	7562	1	7	9	57
**PVGA**	**99.383**	**7569**	**0**	**4**	**4**	**51**

To assess the effectiveness of the PVGA method with extensive short-read datasets, we employ actual Illumina sequencing data of the norovirus. The Norovirus GII complete genome (NC_039477.1) serves as the reference framework for graph construction. We utilize SRR13951201, SRR13951221, and SRR13951199 as input reads, with corresponding ground truth data from Norovirus GII isolates BMH19-145 (Length: 7570 bp), BMH13-039 (Length: 7550 bp), and BMH14-056(Length: 7505bp). The results are in Table 7. Our method PVGA exhibits exceptional accuracy, as evidenced by the absence of insertions, deletions, and mismatches.

Table 8. Results on real Illumina norovirus

Reads	Genome fraction	Genome length	Mismatch	Indels	Indel length	Edit distance
SRR13951201	100	7572	0	0	0	2
SRR13951221	99.574	7485	0	0	0	20
SRR13951199	100	7567	0	0	0	17

In all three Illumina norovirus datasets, PVGA achieves exceptional accuracy, with no mismatches, indels, or indel length errors except for some small misalignment at the two ends of the genomes.

This verifies PVGA's excellent performance in assembling short-read datasets as well.

Benchmarking the capability of polishing

The results from low-coverage HIV sequencing data indicate that, at extremely low depths, some De Novo assemblers, such as Flye and Canu, fail to obtain near-optimal solutions. Comparisons with the ground truth reveal that these assemblers do not achieve complete reconstruction in terms of both length and accuracy. For instance, at a coverage of 15x, Flye shows an edit distance of 315, while Canu has an edit distance of 551. These errors considerably compromise the accuracy of the assembly, adversely impacting subsequent tasks, such as protein structure prediction [34], etc.

To address these inaccuracies, the next essential step is polishing the assembled sequences to enhance their accuracy. A common approach is to use hybrid methods that integrate high-quality short reads, such as those from Illumina, with flawed assemblies. Polishing tools such as Pilon, which utilizes a mapping-based method, align these short reads to the assembly and apply a Bayesian model to determine the most accurate sequence by considering base quality scores and error frequencies. Another tool, NextPolish, similarly aligns short reads and uses an iterative process to correct errors in small regions of the assembly.

To simulate Illumina short reads, we set the qscores parameter to ideal in the Badread tool. We simulate HIV and SARS-CoV-2 reads with lengths of 200–300 base pairs and coverages of 15x and 25x, respectively, for polishing. This setting illustrates the scenario of low coverage, where De Novo assemblers reach performance bottlenecks. We then compare the performance of PVGA with NextPolish and Pilon to refine the results from previous experiments. Specifically, we use the PVGA assembly result with length of 9706 bp from the 15x coverage, 2k read length simulated Nanopore HIV-1 data, as shown in Table 3, and the Canu assembly SARS-CoV-2 result with length of 29493 bp from the 50x coverage, 2k average lengths of reads, as shown in Figure 3. The results in Table 9 demonstrate that PVGA outperforms the best existing methods in terms of polishing

Table 9. Polished result of 15x coverage HIV simulation data and 25x coverage SARS-CoV-2 simulation data

Virus	Tool	Genome fraction	Genome length	Mismatch	Indels	Indel length	Edit distance
HIV (Length: 9713bp)	NextPolish	99.969	9722	2	12	12	17
	Pilon	99.969	9707	0	3	3	6
	**PVGA**	**100**	**9710**	**0**	**0**	**0**	**3**
SARS-CoV-2 (Length: 29646bp)	NextPolish	99.906	29636	2	26	28	58
	Pilon	99.906	29555	0	63	63	91
	**PVGA**	**99.906**	**29619**	**0**	**3**	**3**	**31**

Evaluation of computing resource usage

We evaluate the CPU runtime and memory usage of PVGA in comparison with several widely used assemblers. The datasets used in the experiments included HIV, Measles, and SARS-CoV-2, with sequencing coverages of 50×, 100×, and 200×, and respective genome lengths of approximately 10 kb, 20 kb, and 30 kb. Notably, Accuvir [17] exhibited a runtime exceeding 30 minutes and was therefore excluded from subsequent performance figures. As illustrated in Figure 4, PVGA achieves runtime performance comparable to other mainstream assemblers, though it is not always the best in all scenarios.

Canu and Flye consistently demonstrate the longest runtimes. For Canu, this is primarily due to the computationally intensive processes of read error correction and graph simplification, while Flye's substantial runtime stems from constructing and optimizing the de Bruijn assembly graph. As sequencing coverage increases, the runtime for nearly all assemblers grows proportionally. Interestingly, even with the iterative mechanism, PVGA exhibits significantly lower runtime compared to other assemblers like Canu and Flye. The efficiency gained through iteration primarily stems from the rapid convergence of the process. In the initial iteration, the generated result is already close to the ground truth, providing a more refined backbone for constructing the alignment graph in the next iteration. As the backbone becomes increasingly accurate, subsequent iterations achieve more precise alignments, and the dynamic programming algorithm further optimizes the assembly. This iterative refinement continues until the backbone and the assembled result become identical, ensuring that the assembly process reaches a stable and accurate configuration in a relatively short time.

In terms of memory consumption, we monitor the maximum memory usage during the assembly process across different assemblers. As shown in Figure 4, assemblers relying on alignment graph construction tend to require more memory such as PVGA and PBDAG-Con. This is because PVGA employs a more complex global graph processing approach, which necessitates storing and manipulating a large amount of graph data, leading to higher memory consumption. In contrast, Canu and Flye typically break the input data into smaller chunks, which reduces memory usage and facilitates multi-threaded optimization. However, given the relatively small size of viral genomes compared to those of other organisms, PVGA's memory consumption remains within a reasonable range for viral genome assembly. All the computing resource experiments are conducted on an Apple M2 chip for evaluation.

Conclusion

PVGA is a powerful virus-focused assembler that does both assembly and polishing. For virus genomes, small changes will lead to significant differences in terms of viral function and pathogenicity. Thus, for virus-focused assemblers, high-accuracy results are crucial. Our approach heavily depends on the input reads as evidence to produce the reported genome. It first adopts a reference genome to start with. We then align all the reads against the reference genome to get an alignment graph. After that, we use a dynamic programming algorithm to compute a path with the maximum weight of edges supported by reads. Most importantly, the obtained path is used as the new reference genome and the process is repeated until no further improvement is possible.

Our method works for both long and short reads. Experiments show that PVGA always outperforms the best existing methods in various cases. In particular, simulated Nanopore datasets show that our method can correctly report the true genomes with 0 mismatch and 0 indels.

Availability of source code and requirements

- Project name: PVGA
- Project home page: e.g. `https://github.com/SoSongzhi/PVGA`
- Operating system(s): Platform independent
- Programming language: Python
- Other requirements: numpy, pysam 0.22.0 or higher, pandas 1.5.2 or higher, Bio 1.7.1 or higher, biopython 1.83 or higher, consensus 1.0.5 or higher, networkx 3.1 or higher, pandas 1.5.2 or higher, python 3.10
- Biotools: QUAST 5.3.0, Badread 0.4.1
- License: MIT License

This needs to be under an Open Source Initiative approved license where practicable compiled running software is made available. If the code is not hosted in a repository the *GigaScience* GitHub repository is also available for this purpose.

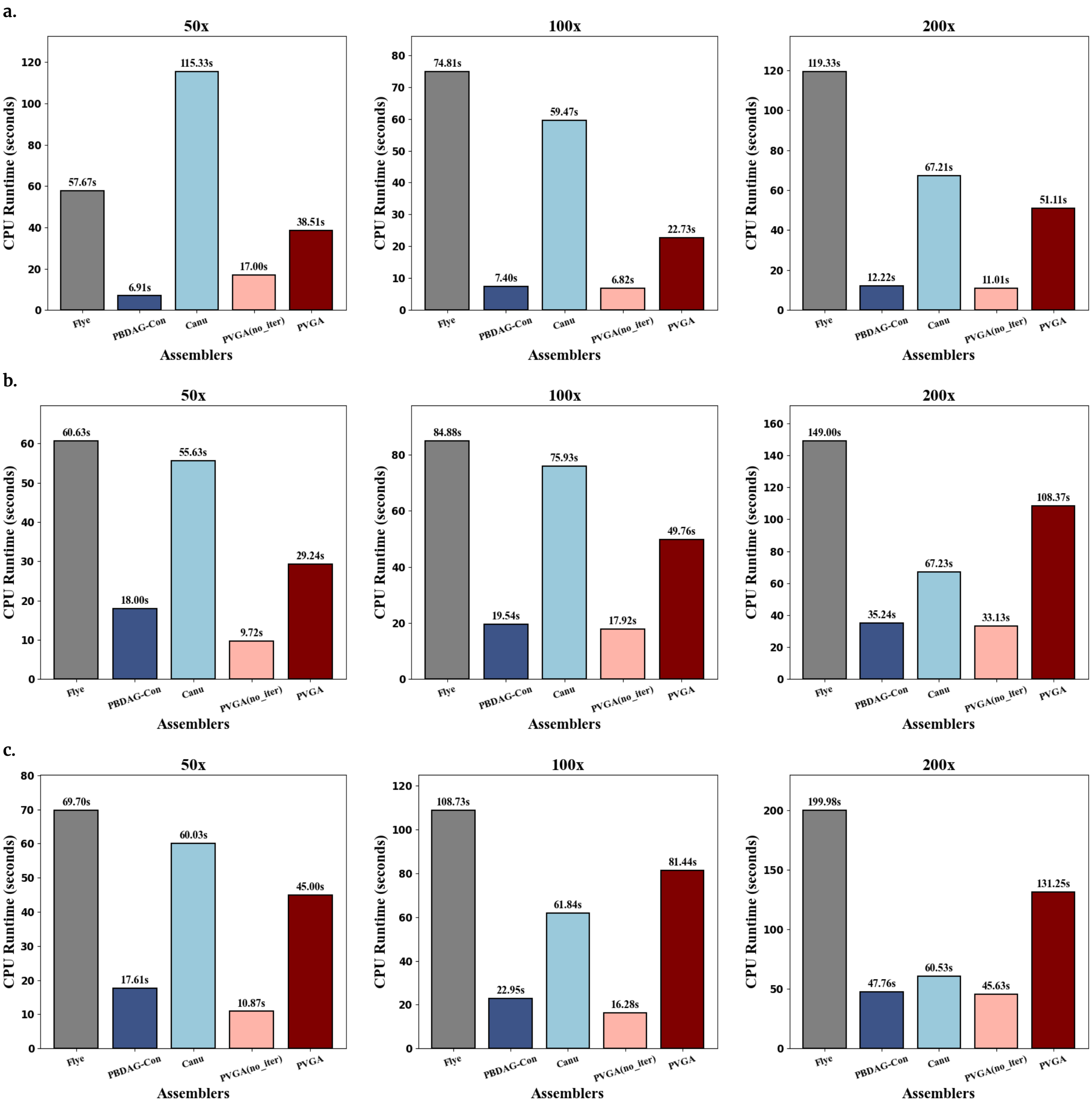

Figure 4. Comparison of CPU times for the five tools on the three datasets of 50x, 100x, 200x coverage respectively, **a.** HIV virus 89.6 Strain, **b.** Measles Virus, **c.** SARS-CoV-2

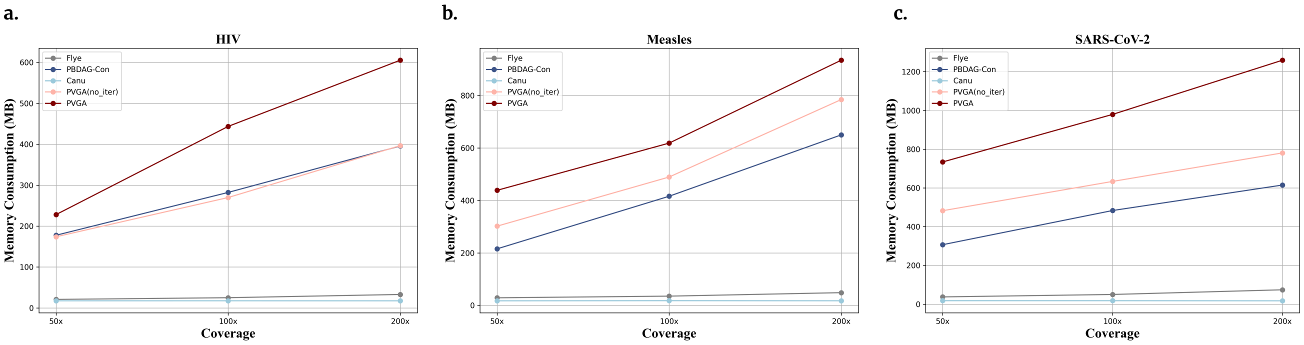

Figure 5. Comparison of maximum memory consumption during the runtime across three datasets with 50×, 100×, and 200× coverage: **(a)** HIV-1 89.6 strain, **(b)** Measles virus, **(c)** SARS-CoV-2.

Additional Files

Supplementary Table 1. Results on simulated PacBio HIV-1 datasets with a 5% error rate and an average read length of 2kb.
Supplementary Table 2. Results on simulated PacBio HIV-1 datasets with a 5% error rate and an average read length of 4kb.
Supplementary Table 3. Results on simulated PacBio HIV-1 datasets with a 5% error rate and an average read length of 6kb.
Supplementary Table 4. Results on simulated PacBio SARS-CoV-2 datasets with 5% error rate and an average read length of 2kb.
Supplementary Table 5. Results on simulated PacBio SARS-CoV-2 datasets with 5% error rate and an average read length of 4kb.
Supplementary Table 6. Results on simulated PacBio SARS-CoV-2 datasets with 5% error rate and an average read length of 8kb.

Data availability

HIV:

- 89.6 Strain complete genome is available in the NCBI database (GenBank: U39362.2)
- HXB2 Strain complete genome is available in the NCBI database (GenBank: K03455.1)
- haplotype benchmarking dataset which cantain mixed PacBio reads from five strains (HXB2, 89.6, JR-CSF, NL4-3, YU-2) [22] is availble in https://github.com/cbg-ethz/5-virus-mix

SARS-CoV-2:

- SARS-CoV-2 isolate Wuhan-Hu-1 is available in the NCBI database (NCBI Reference Sequence: NC_045512.2)
- SARS-CoV-2 isolate INEI121916 is available int the NCBI database (GenBank: OZ072292.1)

Norovirus:

- Nanopore sequencing reads are available in the NCBI database (Run: SRX10330013)
- Illumina sequencing reads are available in the NCBI database (Run: RR13951201, Run: SRR13951221 and Run: SRR1395119)
- The complete genome of Norovirus GII is available in the NCBI database (GenBank: GenBank: MW661264.1, MW661284.1, GenBank: MW661248.1 and GenBank: MW661250.1)

Ebola virus:

- Ebola virus (EBOV-May) Mayinga strain complete genome is available in the NCBI database (GenBank: AF086833.2)
- Ebola virus/M.fascicularis-wt/GAB/2001/untreated-CCL053D9, complete genome is available in the NCBI database (GenBank: KY786027.1)

Measles virus:

- Measles complete genome is available in the NCBI database (NCBI Reference Sequence: NC_001498.1)
- Measles virus genotype A transgenic strain vac2(GFP)H genome is available in the NCBI database (GenBank: MH144178.1)

Declarations

List of abbreviations

- EBOV: Ebola virus
- GP: glycoprotein
- HGAP: Hierarchical Genome-assembly Process
- HIV: Human Immunodeficiency Virus
- NBCI: National Center for Biotechnology Information (NCBI).
- NP: nucleoprotein
- NGS: Next-Generation Sequencing
- SARS-CoV-2: Severe Acute Respiratory Syndrome Coronavirus 2
- SMRT: Single Molecule Real-Time
- TGS: Third-Generation Sequencing

Consent for publication

Not applicable.

Competing Interests

No competing interests.

Funding

This work is fully supported by funds from the National Science Foundation (NSF: 61972329) and GRF grants for Hong Kong Special Administrative Region, P. R. China (CityU 11210119, and CityU 11206120).

Author's Contributions

L.W. conceived the original idea and supervised the entire work. Z.S. continuously expanded the work, conceptualized the iterative process, developed the entire PVGA algorithm, and was the primary contributor to the manuscript. D.C. provided suggestions during the experimental phase and assisted in locating real data for testing. Y.S. contributed valuable insights for the experimental design. All authors participated in reviewing and improving the manuscript.

Acknowledgements

This work is fully supported by funds from the National Science Foundation (NSF: 61972329) and GRF grants for Hong Kong Special Administrative Region, P. R. China (CityU 11210119, and CityU 11206120).

Authors' information

- **Zhi Song** is a Ph.D. candidate in the Department of Computer Science at City University of Hong Kong. His research interests include algorithms, machine learning, computational biology, and bioinformatics.
- **Dehan Cai** is a Ph.D. candidate in the Department of Electrical Engineering at City University of Hong Kong. His main research interests include sequence analysis in bioinformatics and computational biology, focusing on developing tools for microbial analysis using machine learning and statistical methods.
- **Yanni Sun** is a professor in the Department of Electrical Engineering at City University of Hong Kong. Before relocating to Hong Kong, she was an Associate Professor in the Department of Computer Science and Engineering at Michigan State University, USA. She received both her BS and MS degrees from Xi'an Jiao Tong University (China) in Computer Science and her PhD in Computer Science and Engineering from Washington University in Saint Louis, USA. Her research interests include bioinformatics and computational biology, particularly sequence analysis, machine learning, data mining for next-generation sequencing data, metagenomics, protein domain annotation, and noncoding RNA annotation. She received the NSF CAREER Award in 2010.
- **Lusheng Wang** obtained his Ph.D. in Computer Engineering

from McMaster University in 1995. He is currently a professor in the Department of Computer Science at City University of Hong Kong. His research interests include algorithms, computational biology, and bioinformatics.

References

1. Harvey WT, Carabelli AM, Jackson B, Gupta RK, Thomson EC, Harrison EM, et al. SARS-CoV-2 variants, spike mutations and immune escape. Nature Reviews Microbiology 2021;19(7):409–424.
2. Jain M, Koren S, Miga KH, Quick J, Rand AC, Sasani TA, et al. Nanopore sequencing and assembly of a human genome with ultra-long reads. Nature biotechnology 2018;36(4):338–345.
3. Eid J, Fehr A, Gray J, Luong K, Lyle J, Otto G, et al. Real-time DNA sequencing from single polymerase molecules. Science 2009;323(5910):133–138.
4. Wenger AM, Peluso P, Rowell WJ, Chang PC, Hall RJ, Concepcion GT, et al. Accurate circular consensus long-read sequencing improves variant detection and assembly of a human genome. Nature biotechnology 2019;37(10):1155–1162.
5. Zerbino DR, Birney E. Velvet: algorithms for de novo short read assembly using de Bruijn graphs. Genome research 2008;18(5):821–829.
6. Simpson JT, Wong K, Jackman SD, Schein JE, Jones SJ, Birol I. ABySS: a parallel assembler for short read sequence data. Genome research 2009;19(6):1117–1123.
7. Bankevich A, Nurk S, Antipov D, Gurevich AA, Dvorkin M, Kulikov AS, et al. SPAdes: a new genome assembly algorithm and its applications to single-cell sequencing. Journal of computational biology 2012;19(5):455–477.
8. Kolmogorov M, Yuan J, Lin Y, Pevzner PA. Assembly of long, error-prone reads using repeat graphs. Nature biotechnology 2019;37(5):540–546.
9. Koren S, Walenz BP, Berlin K, Miller JR, Bergman NH, Phillippy AM. Canu: scalable and accurate long-read assembly via adaptive k-mer weighting and repeat separation. Genome research 2017;27(5):722–736.
10. Liu J, Yu T, Mu Z, Li G. TransLiG: a de novo transcriptome assembler that uses line graph iteration. Genome biology 2019;20:1–9.
11. Li H, Durbin R. Fast and accurate short read alignment with Burrows–Wheeler transform. bioinformatics 2009;25(14):1754–1760.
12. Langmead B, Salzberg SL. Fast gapped-read alignment with Bowtie 2. Nature methods 2012;9(4):357–359.
13. McKenna A, Hanna M, Banks E, Sivachenko A, Cibulskis K, Kernytsky A, et al. The Genome Analysis Toolkit: a MapReduce framework for analyzing next-generation DNA sequencing data. Genome research 2010;20(9):1297–1303.
14. Mu JC, Jiang H, Kiani A, Mohiyuddin M, Bani Asadi N, Wong WH. Fast and accurate read alignment for resequencing. Bioinformatics 2012;28(18):2366–2373.
15. Li H, Ruan J, Durbin R. Maq: Mapping and assembly with qualities. Version 06 2008;3:508.
16. Hunt M, Gall A, Ong SH, Brener J, Ferns B, Goulder P, et al. IVA: accurate de novo assembly of RNA virus genomes. Bioinformatics 2015;31(14):2374–2376.
17. Yu R, Cai D, Sun Y. AccuVIR: an ACCUrate VIRal genome assembly tool for third-generation sequencing data. Bioinformatics 2023;39(1):btac827.
18. Walker BJ, Abeel T, Shea T, Priest M, Abouelliel A, Sakthikumar S, et al. Pilon: an integrated tool for comprehensive microbial variant detection and genome assembly improvement. PloS one 2014;9(11):e112963.
19. Hu J, Fan J, Sun Z, Liu S. NextPolish: a fast and efficient genome polishing tool for long-read assembly. Bioinformatics 2020;36(7):2253–2255.
20. Chin CS, Alexander DH, Marks P, Klammer AA, Drake J, Heiner C, et al. Nonhybrid, finished microbial genome assemblies from long-read SMRT sequencing data. Nature methods 2013;10(6):563–569.
21. Wick RR. Badread: simulation of error-prone long reads. Journal of Open Source Software 2019;4(36):1316.
22. Giallonardo FD, Töpfer A, Rey M, Prabhakaran S, Duport Y, Leemann C, et al. Full-length haplotype reconstruction to infer the structure of heterogeneous virus populations. Nucleic acids research 2014;42(14):e115–e115.
23. Li H. Minimap2: pairwise alignment for nucleotide sequences. Bioinformatics 2018;34(18):3094–3100.
24. Danecek P, Bonfield JK, Liddle J, Marshall J, Ohan V, Pollard MO, et al. Twelve years of SAMtools and BCFtools. Gigascience 2021;10(2):giab008.
25. Flint A, Reaume S, Harlow J, Hoover E, Weedmark K, Nasheri N. Genomic analysis of human noroviruses using combined Illumina–Nanopore data. Virus Evolution 2021;7(2):veab079.
26. Bukreyev A, Volchkov V, Blinov V, Netesov S. The VP35 and VP40 proteins of filoviruses: homology between Marburg and Ebola viruses. FEBS letters 1993;322(1):41–46.
27. Volchkov VE, Volchkova VA, Slenczka W, Klenk HD, Feldmann H. Release of viral glycoproteins during Ebola virus infection. Virology 1998;245(1):110–119.
28. Guedj J, Piorkowski G, Jacquot F, Madelain V, Nguyen THT, Rodallec A, et al. Antiviral efficacy of favipiravir against Ebola virus: A translational study in cynomolgus macaques. PLoS medicine 2018;15(3):e1002535.
29. Del Valle JR, Devaux P, Hodge G, Wegner NJ, McChesney MB, Cattaneo R. A vectored measles virus induces hepatitis B surface antigen antibodies while protecting macaques against measles virus challenge. Journal of virology 2007;81(19):10597–10605.
30. Pfaller CK, Mastorakos GM, Matchett WE, Ma X, Samuel CE, Cattaneo R. Measles virus defective interfering RNAs are generated frequently and early in the absence of C protein and can be destabilized by adenosine deaminase acting on RNA-1-like hypermutations. Journal of virology 2015;89(15):7735–7747.
31. Lee JY, Kong M, Oh J, Lim J, Chung SH, Kim JM, et al. Comparative evaluation of Nanopore polishing tools for microbial genome assembly and polishing strategies for downstream analysis. Scientific Reports 2021;11(1):20740.
32. Ip CL, Loose M, Tyson JR, de Cesare M, Brown BL, Jain M, et al. MinION Analysis and Reference Consortium: Phase 1 data release and analysis. F1000Research 2015;4.
33. Delahaye C, Nicolas J. Sequencing DNA with nanopores: Troubles and biases. PloS one 2021;16(10):e0257521.
34. Watson M, Warr A. Errors in long-read assemblies can critically affect protein prediction. Nature biotechnology 2019;37(2):124–126.

Manuscript Click here to access/download;Manuscript;supplementary.pdf

SUPPLEMENTARY DOCUMENT

1 Benchmarking results on Pacbio Datasets

Beyond testing on Nanopore data, we also test simulated PacBio data. Tables 1-3 present the results for HIV PacBio datasets, with average read lengths of 2kb, 4kb, and 6kb, respectively, while Tables 4-6 display the benchmarking results for SARS-CoV-2 at average read lengths of 2kb, 4kb, and 6kb, respectively. For both HIV and SARS-CoV-2 results, PVGA consistently demonstrates the lowest values across all conditions in terms of mismatches, indels, indel lengths, and edit distance, indicating that PVGA consistently outperforms other assemblers.

When using Badread to simulate PacBio-format reads, the quality of the reads is lower compared to those generated by Nanopore. This decline adversely affects the performance of nearly all assemblers in comparison to the Nanopore results. Nevertheless, PVGA continues to outperform all other assemblers, consistently exhibiting the lowest numbers of indels, mismatches, and edit distances. For instance, as shown in Table 6, at coverage of 50x with an 8kb read length from SARS-CoV-2 datasets, other assemblers report indels, mismatches, and edit distances in double or even triple digits, whereas PVGA maintains single-digit values for each of these metrics.

Table 1: Results on simulated PacBio HIV-1 datasets with a 5% error rate and an average read length of 2kb

Reads depth	Tool	Genome fraction	Contig length	Mismatch	Indels	Indel length	Edit distance
50x	Flye	89.9	8669	0	63	63	1044
	Canu	93.452	12459	0	157	163	7848
	Accuvir	99.959	9678	1	45	49	54
	PBDAG-Con	99.969	9635	0	70	79	82
	Medaka	99.969	9698	9	30	48	60
	PVGA(noiter)	99.969	9703	0	9	9	12
	**PVGA**	**99.969**	**9703**	**0**	**9**	**9**	**12**
100x	Flye	99.938	9642	0	65	65	79
	Canu	99.732	9619	0	66	68	89
	Accuvir	99.959	9672	1	39	43	48
	PBDAG-Con	99.969	9644	0	60	72	75
	Medaka	99.969	9672	8	37	58	69
	PVGA(noiter)	99.969	9710	0	4	4	7
	**PVGA**	**99.969**	**9711**	**0**	**3**	**3**	**6**
200x	Flye	99.99	9635	0	77	77	78
	Canu	93.452	12966	0	131	138	6157
	Accuvir	99.959	9669	0	46	48	52
	PBDAG-Con	99.969	9633	0	69	81	84
	Medaka	99.969	9737	10	17	41	54
	PVGA(noiter)	99.969	9698	4	33	48	55
	**PVGA**	**99.969**	**9707**	**0**	**3**	**3**	**6**

Table 2: Results on simulated PacBio HIV-1 datasets with a 5% error rate and an average read length of 4kb

Reads depth	Tool	Genome fraction	Contig length	Mismatch	Indels	Indel length	Edit distance
50x	Flye	98.312	9490	0	59	59	1066
	Canu	99.979	24809	0	181	194	15224
	Accuvir	99.959	9675	0	43	44	48
	PBDAG-Con	99.969	9648	0	56	62	65
	Medaka	99.969	9687	8	26	48	59
	PVGA(noiter)	99.969	9704	0	10	10	13
	**PVGA**	**99.969**	**9704**	**0**	**10**	**10**	**13**
100x	Flye	100	9653	0	60	60	63
	Canu	100	18670	0	143	143	9093
	Accuvir	99.959	9679	1	34	34	39
	PBDAG-Con	99.969	9650	0	53	60	63
	Medaka	99.969	9705	0	8	8	8
	PVGA(noiter)	99.969	9731	7	23	47	57
	**PVGA**	**99.969**	**9710**	**0**	**4**	**4**	**7**
200x	Flye	99.99	9637	0	75	75	76
	Canu	99.99	9689	0	69	69	116
	Accuvir	99.959	9694	1	27	29	34
	PBDAG-Con	99.969	9637	0	64	73	76
	Medaka	99.969	9684	10	32	58	71
	PVGA(noiter)	99.969	9709	0	1	1	4
	**PVGA**	**99.969**	**9710**	**0**	**1**	**1**	**4**

Table 3: Results on simulated PacBio HIV-1 datasets with a 5% error rate and an average read length of 6kb

Reads depth	Tool	Genome fraction	Contig length	Mismatch	Indels	Indel length	Edit distance
50x	Flye	85.689	8266	0	57	57	1471
	Accuvir	99.959	9680	1	40	43	48
	PBDAG-Con	99.969	9657	0	49	59	62
	Medaka	99.969	9694	14	21	52	69
	PVGA(noiter)	99.969	9709	0	7	7	10
	**PVGA**	**99.969**	**9709**	**0**	**7**	**7**	**10**
100x	Flye	99.969	9650	0	63	63	69
	Canu	99.99	9691	0	61	61	102
	Accuvir	99.959	9675	0	52	60	64
	PBDAG-Con	99.969	9640	0	58	70	73
	Medaka	99.969	9690	19	28	61	83
	PVGA(noiter)	99.969	9710	0	2	2	5
	**PVGA**	**99.969**	**9710**	**0**	**2**	**2**	**5**
200x	Flye	99.568	10304	1	97	98	819
	Accuvir	99.959	9682	1	34	35	40
	PBDAG-Con	99.969	9646	0	60	64	67
	Medaka	99.969	9706	0	7	7	7
	PVGA(noiter)	99.969	9737	10	17	41	54
	**PVGA**	**99.969**	**9710**	**0**	**1**	**1**	**4**

Table 4: Results on simulated PacBio SARS-CoV-2 datasets with 5% error rate and an average read length of 2kb

Reads depth	Tool	Genome fraction	Contig length	Mismatch	Indels	Indel length	Edit distance
25x	Flye	97.46	28759	0	139	140	899
	Canu	98.644	29078	0	164	166	2548
	Accuvir	100	29784	1	97	101	236
	PBDAG-Con	100	29554	0	92	93	94
	Medaka	100	29850	0	22	30	254
	PVGA(noiter)	100	29652	0	20	20	20
	**PVGA**	**100**	**29652**	**0**	**20**	**20**	**20**
50x	Flye	99.997	29531	0	118	119	125
	Canu	99.906	29493	0	124	125	153
	Accuvir	100	29792	1	83	85	309
	PBDAG-Con	100	29593	0	78	79	105
	Medaka	100	29855	0	22	25	249
	PVGA(noiter)	100	29647	0	14	14	15
	**PVGA**	**100**	**29647**	**0**	**14**	**14**	**15**

Table 5: Results on simulated PacBio SARS-CoV-2 datasets with 5% error rate and an average read length of 4kb

Reads depth	Tool	Genome fraction	Contig length	Mismatch	Indels	Indel length	Edit distance
25x	Flye	99.993	29501	0	140	143	145
	Canu	99.811	29420	0	166	170	226
	Accuvir	100	29744	1	125	129	353
	PBDAG-Con	100	29552	0	92	95	96
	Medaka	100	29813	0	64	67	291
	PVGA(noiter)	100	29651	0	20	20	21
	**PVGA**	**100**	**29651**	**0**	**20**	**20**	**21**
50x	Flye	99.997	29556	0	93	94	100
	Canu	91.716	27098	0	90	92	2548
	Accuvir	100	29747	1	108	127	337
	PBDAG-Con	100	29579	0	67	68	69
	Medaka	100	29813	0	24	28	252
	PVGA(noiter)	100	29648	0	5	5	6
	**PVGA**	**100**	**29648**	**0**	**5**	**5**	**6**

Table 6: Results on simulated PacBio SARS-CoV-2 datasets with 5% error rate and an average read length of 8kb("-" indicates that the assembler fails to produce a result.)

Reads depth	Tool	Genome fraction	Contig length	Mismatch	Indels	Indel length	Edit distance
25x	Flye	99.997	29531	0	118	119	125
	Canu	99.906	29493	0	124	125	153
	Accuvir	100	29792	1	83	85	309
	Medaka	100	29609	0	43	45	270
	PBDAG-Con	100	29593	0	78	79	105
	PVGA(noiter)	100	29647	0	14	14	15
	**PVGA**	**100**	**29647**	**0**	**14**	**14**	**15**
50x	Flye	99.983	29572	0	92	93	114
	Canu	-	-	-	-	-	-
	Accuvir	100	29807	0	66	68	291
	Medaka	100	29609	0	23	25	270
	PBDAG-Con	100	29577	2	69	70	73
	PVGA(noiter)	100	29649	0	8	8	9
	**PVGA**	**100**	**29650**	**0**	**7**	**7**	**8**
