# Supplementary material for: PVGA: a precise viral genome assembler using an iterative alignment graph
Source: Gigascience. 2025 Jun 24;14:giaf063. doi: 10.1093/gigascience/giaf063 (PMC12206156; doi:10.1093/gigascience/giaf063)
Supplement: giaf063_GIGA-D-25-00004_Revision_1 [file giaf063_giga-d-25-00004_revision_1.pdf]

// GigaScience

GigaScience

PVGA: A Precise Viral Genome Assembler Using Iterative Alignment Graph

--Manuscript Draft--

**Manuscript Number:**	GIGA-D-25-00004R1
**Full Title:**	PVGA: A Precise Viral Genome Assembler Using Iterative Alignment Graph
**Article Type:**	Technical Note
**Funding Information:**	
**Abstract:**	Abstract<br>Background: Viral genome analysis is crucial for understanding viruses evolution and mutation. Investigations into viral evolutionary dynamics and mutation patterns have garnered significant research attention since the outbreak of COVID-19. As the basic structure of many virus genomes is highly conserved [1]. RNA viruses have high mutation rates, and single-nucleotide variations may induce substantial phenotypic alterations in terms of viral function and pathogenicity. Thus, special assembly methods are required for viral genome analysis.<br>Result: PVGA starts with a reference genome and utilizes the sequencing reads directly to reduce noise. The first step in PVGA involves constructing an alignment graph based on a reference genome and the set of input sequencing reads. Then the optimal genomic path is determined through dynamic programming, maximizing the cumulative edge weights that reflect read support density across the alignment graph. The obtained path corresponds to a refined genome. Finally, we repeat the process by using the new reference genomes until no further improvement is possible. We evaluate PVGA's performance across both assembly and polishing tasks using simulated and real datasets including both long reads and short reads. The experiments demonstrate that PVGA always outperforms popular existing programs in terms of the quality of assembly results, while the running time of our method is compatible to others. In particular, simulated Nanopore datasets show that our method can correctly report the true genomes with 0 mismatch and 0 indels.<br>Conclusions: PVGA is a novel viral genome assembler that seamlessly integrates assembly and polishing into a unified workflow. Its design prioritizes high accuracy, enabling the detection of subtle genomic variations that can impact viral function and pathogenicity. By addressing the unique challenges of viral genome assembly, PVGA provides a reliable and precise solution for advancing our understanding of viral evolution and behavior.<br>Key words: Genome assembler; Virus genome; Alignment graph; Maximum total weight path; Iterative method.
**Corresponding Author:**	Lusheng Wang<br>City University of Hong Kong<br>Hong Kong, Kowloon HONG KONG
**Corresponding Author Secondary Information:**	
**Corresponding Author's Institution:**	City University of Hong Kong
**Corresponding Author's Secondary Institution:**	
**First Author:**	Zhi Song
**First Author Secondary Information:**	
**Order of Authors:**	Zhi Song
	Dehan Cai
	Yanni Sun
	LUSHENG WANG
**Order of Authors Secondary Information:**	
**Response to Reviewers:**	Dear Editor and Reviewers,

Powered by Editorial Manager® and ProduXion Manager® from Aries Systems Corporation

We would like to thank you for your careful reading of our paper and the valuable comments. We have addressed the comments of all reviewers, with their comments in black and our responses in blue. The revised figures and descriptions were added to the main manuscript and the supplementary file.

Before we provide the detailed responses, we would like to summarize the major improvements.

1.The tool is registered in bio.tools (RRID:SCR_026410) and its computational workflows are permanently archived at WorkflowHub (DOI:10.48546/workflowhub.workflow.1305.1). To ensure broad accessibility, PVGA supports both PyPI installation (pip install pvga) and Bioconda deployment (conda install -c bioconda pvga).
2.We have added our detail description of our parameters in PVGA at our Github repository https://github.com/SoSongzhi/PVGA.
3.We have expanded our experiments to test how backbone choice affects assembly accuracy. We use real HIV strains (89.6, HXB2, NL43, YU2) as backbones for assembling the JRCSF genome, we also created computer-modified JRCSF backbones with increasing differences (15%, 20%, 25%, 30%) through controlled random mutations. Results in Tables 10-11 show how these backbone variations impact assembly quality.
See subsection “Convergence performance across different backbones”

We hope that the following point-to-point responses will be clear enough to convey our ideas and address the comments of the reviewers.

Response to Reviewer 1’s comments

Major review points:
(1) Please add one proof of concept analysis for PVGA’s algorithmic convergence starting from
different backbone genomes. A concern of mine is that the algorithm might converges towards
certain alleles or strains when different backbone genomes are used. This can be tested with
just three – or more – different strains of the same species. E.g. from three distinct strains
{sample= X, backbones= {Y, Z}} you take reads from X and assemble them with Y and Z as
backbones. Then check whether both approaches still assemble X accurately. Ideally and to be
convincing, the genomes Y and Z in the set of backbones should be reasonably distant in
sequence similarity and no closely related recombinants.

This is one of the major improvements, which we have responded as item 3 in major improvements. Basically, we add subsection “Convergence performance across different backbones” to address the issue. See Page. 8.

(2) I tried using the software and run it on some very simple SARS-CoV-2 sequence data. I noticed several issues that made it difficult to install and impractical to use the software yet. Please address the following issues and consider suggestions regarding the software:

a. [Issue]
The installation instructions do not work without further ado.
conda create pvga --name pvga python==3.10
This line has pvga as a positional argument. This does not work in conda. Further, if the user makes it until
pip install -r requirements.txt
then the pip dependency “python==3.10” collides and breaks because of the python instance previously installed via conda.

Powered by Editorial Manager® and ProduXion Manager® from Aries Systems Corporation

We sincerely appreciate this critical technical feedback. Users now can download PVGA with follow commands:

conda create --name pvga python=3.10
conda activate pvga
conda install -c bioconda pvga

b. [Issue]
Once I got through the installation section I couldn't get PVGA to run. I provided a backbone genome (-b) and a set of ONT reads (-r). Now, the software informs me that a number if iterations (-n) is required as well as a ground truth sequence (-gt). The paper in contrast says that the iterative process simply runs until no further improvement is made in an iteration. Hence, I expect this parameter not to be required. If even specified, I'd understand this parameter as an upper bound. Also, in practical applications there is no known ground truth. Why is this parameter required?

We appreciate this critical technical feedback. The -n parameter was implemented to evaluate the influence of iterations on assembly results. For instance, setting -n 1 allows us to obtain results without iteration, as demonstrated in the benchmarking figure in the paper. Additionally, the -gt parameter introduced in the previous version was to input ground truth for alignment tool QUAST to evaluate the performance of the assembly. In the user-facing version, based on your recommendation, we have removed the -gt parameter and made the iteration number -n optional, with a default value as the upper bound. Users can still specify the iteration number as needed.

c. [Issue]
Hypothetically, if I could make PVGA run I wouldn't know how to provide paired-end short-read sequencing data. Since there is only one read parameter (-r) do I need to provide short-read paired-end data as interleaved fastq file? Please improve documentation.

Thank you for this valuable advice. While PVGA takes single reads as input, it can also process paired-end reads. Users with paired-end data can merge their FASTQ files using BBMap while preserving paired-end information. The merged FASTQ file, where overlapping read pairs are combined into longer single reads, can then be used as input for PVGA assembly. By combining these longer single reads with the unmerged reads from both FASTQ files, PVGA can effectively handle paired-end data. This approach maintains compatibility with PVGA's single-read input design while ensuring efficient processing of paired-end datasets. We have included this explanation along with the BBMap command lines in our README.

d. [Improvement suggestion]
For such a simple installation instruction you utilized two package management systems, conda and pip. Everything necessary to run PVGA can be installed via one concise conda environment. I suggest such an approach would be greatly appreciated by the users.
e. [Improvement suggestion]
Since every dependency can be installed via conda it would be of great help and simplicity for the user if PVGA itself could be installed via (bio)conda - https://bioconda.github.io/. This is a user-friendly way to distribute your bioinformatics software.

Thanks for the valuable suggestions regarding package management. PVGA is now officially available on both Bioconda (conda install -c bioconda pvga) and PyPI (pip install pvga), providing users with flexible installation options to suit their preferred workflows.

f.[Improvement suggestion]
It is good software development practice that the software complies with semantic versioning - https://semver.org/. Please display the version on the help page (--help). The shield on your Github README can then automatically use this implemented version.

Powered by Editorial Manager® and ProduXion Manager® from Aries Systems Corporation

	Thanks for this valuable improvement suggestion. We have updated PVGA to comply with semantic versioning as per semver.org. The version number is now prominently displayed in the help page (via --help) and integrated into the GitHub README using dynamic shields from shields.io. We will maintain strict adherence to semantic versioning in all future releases.<br>g. [Improvement suggestion]<br>Short parameter of two letters is rather confusing and unnecessary for PVGA's short list of parameters. You might like to have a look at Heng Li's CLI best practice - https://lh3.github.io/2021/07/04/designing-command-line-interfaces.<br><br>Thanks for this valuable improvement suggestion. we have revised PVGA's command-line interface to use single-letter short parameters exclusively (e.g., -r for reads, -b for backbone, -o for output directory), accompanied by clear and detailed descriptions in the help page (--help).<br><br>(1) Please improve some minor language errors:<br>a. [Intro] (Article) "The virus genomes have relatively small size."<br><br>We totally agree with your suggestion. We have removed this sentense.<br><br>b. [Intro] (Article) "For example, several mutations in the spike protein gene of coronavirus can […]"<br><br>We totally agree with your suggestion. "For example, several mutations in the spike protein gene of coronavirus can […]" has been modified to "For example, genetic mutations in the coronavirus genome that alter the spike protein can affect its ability to interact with host cells, thereby affecting transmissibility and disease severity." See the bottom of the left column on Page 1.<br><br>c. [Intro] (Wording) "[…] it is well-known that NGS struggles with assembling repetitive regions in the genome" → NGS itself is not performing assembly; better something like "difficult to assemble repetitive regions using NGS data"<br><br>We totally agree with your suggestion. "[…] it is well-known that NGS struggles with assembling repetitive regions in the genome" has been modified to "However, due to the short read lengths and the presence of repetitive regions, it is often challenging to assemble genomes accurately using NGS data." See the second paragraph of the Section "Introduction".<br><br>d. [Datasets] (Wording) "[…] as a target to generate the simulation reads." → "as a target and to generate simulated reads" or "as a target and to generate synthetic reads"<br><br>Thanks for your suggestion. "[…] as a target to generate the simulation reads." has been modified to "as a target and to generate simulation reads.". See the subsubsection "SAR-CoV-2" in the subsection "Datasets" See the bottom of the right column on Page 1.<br><br>e. [Datasets] (Typo) "We utilize two Measles virus strain." → plural "strains"<br><br>Thanks for your suggestion. "We utilize two Measles virus strain." has been modified to "We utilize two Measles virus strains." See the middle of the right column on Page 4.<br><br><br>f. [Evaluation on simulated data] (Wording) "[…] refinement process significantly enhances assembly accuracy." → I am against "significant" here since, in the scientific context, this usually means it's statistically tested which is rather difficult for these small numbers.<br><br>Thanks for your suggestion. The word "significant" has been removed.<br><br>g. I stop explicitly listing here but there are more errors, e.g. with the plural and relative clauses. Please spellcheck in general.

Powered by Editorial Manager® and ProduXion Manager® from Aries Systems Corporation

	(2) [Intro]<br>When you introduce reference-based assembly you cite tools like BWA, Bowtie and GATK. To<br>the best of my knowledge, these tools are primarily read mapper and variant caller. Both read<br>mapping and variant calling serve as a prior for reference-guided assembly but it is technically<br>not the assembly process they are performing. Assembly, both de novo and reference-guided,<br>should yield a consensus sequence or contigs as an output. Software like bcftools (https://samtools.github.io/bcftools/bcftools.html), iVar (https://github.com/andersen-lab/ivar) or AccuVir perform reference-guided assembly. Please improve the wording here or<br>correct accordingly.<br><br>Thanks for pointing out the error. We have removed the description of these tools from the section on reference-based assembly. Instead, we now focus on tools specifically designed for reference-guided assembly. The comparison before and after modification is as follows:<br><br>Origin: “Famous reference-guided assembly methods include BWA, Bowtie2, GATK, Novoalign, and Maq.”<br><br>Revised: “Famous reference-guided assembly methods include Novoalign, Maq, iVar, Accuvir and bcftools.” See the middle of the left column of Page 2.<br><br>(3) [Intro]<br>Please comment on the shortcoming of previous approaches that led to the motivation to<br>develop another reference-guided assembler.<br><br>Thank you for your valuable suggestion. We have addressed the shortcoming of previous approaches in the introduction by stating: “However, despite these advancements, current genome assembly tools still fail to achieve the requisite base-level accuracy for viral genome assembly.” See the middle of the left column of Page 2.<br><br>(4) [Methods – Alignment graph construction]<br>The process is mostly well defined and nicely visualized with Figure 1. However, I have several<br>detail questions here that need to be addressed in the manuscript:<br>a.What is ’? I assume from the context that it is the new node after a merging : , → ’ where and had the same label and parent.<br><br>Yes, you are correct. ’ is indeed the new node created after merging nodes and , which share the same label and parent. To clarify this process, we have added an illustration showing how ’ is derived, along with its predecessor node as follows:<br><br>Origin: “The weight of the remaining edge (v, u') is updated to be the number of supporting reads.”<br>Revised: “When merging nodes into a new node u' with its predecessor v, the weight of the resulting edge (v, u') is updated to reflect the total number of supporting reads.” See the subsection “Alignment graph construction”<br><br>b.A very crucial detail is hidden behind the wording “If is matched with an identical letter in the alignment, then corresponds to the node in in .” This matching is a complex problem itself. From your Figure 1c I can only hypothesize that some sort of sequence-to-graph alignment is involved. Please describe the matching in more detail.<br><br>Thanks for your advise. Our alignment graph methodology is primarily based on the paper "Nonhybrid, finished microbial genome assemblies from long-read SMRT sequencing data", which provides a detailed methodology for constructing the alignment graph. Specifically, we first align the reads to the backbone genome using

BLASR software, then construct the graph. We focus more explicitly on the algorithmic steps for building the alignment graph following read-to-backbone genome alignment, and we have supplemented these details in the manuscript as follows：

"In the alignment, if ri is aligned to an identical letter sj in the backbone sequence, then ri corresponds to the existing node vj in the graph Gb. The weight of the edge (vj–1, vj) will be incremented by one, where vj–1 is the predecessor node of vj. If ri is aligned with a space or a letter sj not identical to ri, we will create a new node ui labeled with ri and add an edge (ui–1, ui) with weight 1, where ui–1 is the node corresponding to the previous letter ri–1. See Fig. 1(b)."
See the subsection "Alignment graph construction"

c.What happens in the backtracking if there is a numeric tie between the weights (alleles)? Can alleles accidentally swap in and out per iteration?

Thanks for your question. Based on our empirical observations, we have not encountered instances of numeric ties between allele weights. From an algorithmic perspective, if such a tie were to occur, our method would resolve it by selecting one of the alleles as the final result This selection process ensures deterministic output while maintaining assembly consistency.

(5) [Methods – Evaluation on simulated data]
Please state whether the competing methods (Canu, Flye, AccuVir, PBDAG-Con, Medaka) are
de novo or reference-based methods.
Also, PEPPER-Margin is a quite popular pipeline for phased haplotype inference. To avoid
adding more analyses here you can argue that you improve upon PEPPER-Margin via implication through AccuVir.

Thank you for your suggestion. Canu and Flye are De Novo methods, while Accuvir, PBDAG-Con, and Medaka are reference-based methods. We have added this description in the revised manuscript. In Accuvir, PEPPER and Margin were tested extensively on simulated data experiments, and the results showed that Accuvir and Medaka outperformed PEPPER-Margin in most cases. Since we used a similar approach to generate simulated data and given the superior performance of Accuvir and Medaka, we benchmarked PVGA only against Medaka and Accuvir, excluding the others from our analysis.

(6) [Methods – Evaluation on simulated data]
Have you left out an evaluation on simulated short-read data? If so, why?

Yes, we evaluated PVGA on real-world Illumina short-read datasets (Table 8), focusing on norovirus sequencing data (SRR13951201, SRR13951221, SRR1395119 with read lengths 301/201). This aligns with our methodology stated in the text: "To assess the effectiveness of the PVGA method with extensive short-read datasets, we employ actual Illumina sequencing data of the norovirus. […]" See the bottom of the right column of Page 2.

(7) [Methods – Evaluation on simulated data]
"Edit distance indicates a minimum number of operations required to transform the assembled genome into the reference sequence." → "Edit distance defines a minimum number of substitution and indel operations required to transform the assembled genome into the reference sequence."

Thank you for this insightful suggestion. We have revised the definition of edit distance in the Results section as recommended. See the middel part of the right column on the Page 4.

(8) [Methods – Evaluation on simulated data]
"To simulate real sequencing data, we used the Badread […]" → "To simulate sequencing data,

	we used the Badread […]”<br><br>Thank you for this insightful suggestion. We have deleted the word “real”.<br><br>(9) [Methods – Evaluation on simulated data]<br>You applied different error models to Badread to simulate reads of different sequencing technologies but used the same identity settings in both cases, i.e. for PacBio and ONT. Have<br>you investigated whether this leads to a noticeable difference in sequence similarity between<br>the simulated datasets? I.e. if the identity settings are the same for PacBio and ONT, what<br>difference can you observe across the simulated datasets? Please add a brief comparison/number. One quick and easy solution would be to analyze the alignments of the<br>simulated reads with the perl one-liners by Heng Li (https://lh3.github.io/2018/11/25/on-the-<br>definition-of-sequence-identity).<br><br>Thanks for your question. We analyzed the simulated PacBio and Nanopore datasets using Heng Li's alignment-based identity metrics. While the overall read identities were comparable between the two datasets (both approximating the preset identity thresholds in Badread), we observed distinct error profile distributions as documented in Badread's error models (https://github.com/rrwick/Badread/wiki/Error-models). Specifically, PacBio-like simulated reads produced fewer completely correct reads compared to Nanopore-like simulated reads. (see " PacBio2016 model" section in documentation). This indicates that error-containing reads are more likely to occur, leading to increased alignment errors or misalignments during assembly. We have added relavite illustration in the supplemantary file.<br><br>(10) [Table 1,2,3]<br>“length: 9713bp” → “genome length: 9713bp” Maybe rephrase the description. The first half<br>of the sentence suggests that HIV-1 would be a simulation.<br><br>Thank you for this insightful suggestion. We have revised the description as recommended.<br><br>(11) [Evaluation on poor sequencing conditions]<br>I believe this section provides an important and relevant analysis of a more error-prone sequencing experiment. However, please consider adding the following adjustments:<br>a. Please state clearly how you increased the error-rate from an average of 5%(?) to now<br>10%. The former is a little bit hidden in the “identity settings” of the simulation setup.<br><br>Thank you for the constructive feedback. We have explicitly clarified the error rate modification protocol as follows:<br><br>“To simulate poor sequencing conditions, we configured the following parameters for evaluating assembler performance under suboptimal data quality: We apply a truncated normal distribution of basecall identity (range: 85–95%, mean: 90%, SD: 5%), resulting in an average read error rate of 10\% and an upper accuracy bound of 95%. And we assign uniform sequencing depth (30×) and average read length (4 kb) across all viral genomes (HIV, Measles, Ebola) to standardize suboptimal quality conditions. The results are shown as Table5.” See the subsection “Evaluation on poor sequencing conditions”.<br><br>b. You use [32] to reason the high error-rate. While historically true, this paper and its data are a decade old and, in my humble experience, Oxford Nanopore Technologies has done a massive leap in sequencing quality since. Consider adding that the poor sequencing conditions setup reflects particularly poor sequencing runs, usage of outdated flowcells, or assembly of elder sequencing data. All those are common and

perfectly realistic scenarios.

We sincerely thank the reviewer for their valuable insight. Based on your suggestion, we have revised the manuscript to add the rationale for evaluating the assembly of high error-rate reads as follows:
"With the advancement of sequencing technologies, there has been a significant leap in both the capabilities and quality of sequencing. For instance, PacBio sequencing technology can offer HiFi reads that provide an accuracy of 99.9%. However, some laboratories continue to rely on older sequencing equipment or encounter suboptimal results due to experimental limitations. In such cases, there is a need for an assembler capable of effectively handling data with relatively higher error rates." See the subsection "Evaluation on poor sequencing conditions".

(12) [Evaluation on real data]
The data situation is not entirely clear to me. As I understand, the mock community has sequencing data from five distinct HIV genomes. Then, you test the genome reconstruction
with three different subtypes as target. Please address the following questions to better understand the experiment:
a.Why not five targets, one for each genome?

We selected three HIV strains (89.6, JR-CSF, and YU-2) for the assembly analysis because they met our quality criteria. All reads were first aligned to the HXB2 reference genome to standardize the analysis. These three strains have sufficient sequencing depth making them suitable for assembly. In contrast, the NL43 strain are low abundance reads because its reads were of very low quality and had insufficient coverage.

b. I can only assume from the paragraph that 89.6, JR-CSF and YU-2 are members of the
five genomes from the mock community. Is that right?
c. Is the backbone genome HXB2 one member of the five genomes in the mock community?

These two questions were deleted by the reviewers.

d.Please add a reference of the mock community data here, or a hint to the Data availability section.

Thanks for your advise, We have added the citation for the mock HIV-1 community dataset in the subsection "Evaluation on real data", which was sourced from the paper "Giallonardo,F.D. et al. (2014) Full-length haplotype reconstruction to infer the structure of heterogeneous virus populations. Nucleic Acids Res., 42, e115." See the bottom of the left column on the page 7.

(13) [Evaluation on real data]
"As shown in Table 5, as for the 89.6 Strain, Canu produces a contig of only 4,593 base pairs"
→ I think that is supposed to be Table 6

Thank you for catching this inconsistency. We have corrected this reference in the revised manuscript.

(14) [Evaluation on real data]Do you have a reason/hypothesis what is causing the remaining mismatches and indels that remain after iterative PVGA? (e.g. in Table 6)

We attribute the residual mismatches and indels after iterative PVGA primarily to inherent limitations of real-world short-read data quality. This hypothesis aligns with the suboptimal performance of other assemblers (e.g., Accuvir, Medaka and PBDAG-Con) in Table 6), which exhibit higher mismatches compared with their performance under simulations reads. Additionally, the lack of validated ground truth for most real datasets complicates definitive error correction. In our manuscript, we illustrated "Due to the lower quality of real reads, assemblers dis-play higher mismatches" in the top of the left

column on Page 7.

(15) [Evaluation on real data]
“The results are in Table 7. Our method PVGA exhibits exceptional accuracy […]” → I think that
is supposed to be Table 8

Thank you for catching this inconsistency. We have corrected this reference in the revised manuscript.

(16) [Table 8]
Until here I assumed that if the number of mismatches and indels are both zero, an edit distance >0 can only be explained with missing sequence at the flanks of the genome. However, that’s not the case according to the first and third row of Table 8. How is the edit
distance >0 while the genome fraction is 100 and the number of mismatches and indels is zero?
Please comment on what’s the difference here between reconstructed strain and ground
truth. I suppose that is what is meant with “[…] misalignment at the two ends of the genomes.”
Is the reconstructed strain slightly longer than the ground truth?

It was a good question. The Genome fraction metric, as defined by QUAST, measures the proportion of the reference genome covered by aligned contigs, calculated by dividing the total aligned reference bases by the genome size.

Shown as the QUAST instructions: “Genome fraction (%): The total number of aligned bases in the reference, divided by the genome size. A base in the reference genome is counted as aligned if at least one contig has at least one alignment to this base. Contigs from repeat regions may map to multiple places, and thus may be counted multiple times in this quantity.”

This metric prioritizes coverage completeness over sequence accuracy. In Table 8, PVGA’s contigs are slightly longer than the ground truth due to non-reference homologous sequences at the termini. While these terminal extensions do not affect the Genome fraction as the entire ground truth is still covered by the aligned regions. they should be counted in edit distance calculations. We have added the ground truth genome lengths in the Table8’s notes.

(17) [Evaluation of computing resource usage]
“Notably, Accuvir[17] exhibited a runtime exceeding 30 minutes and was therefore excluded
from subsequent performance figures.” → I couldn’t find running time analyses in the AccuVir
publication but they ran analyses on 200x PacBio data. Can you double-check that your
code/command was running successfully in general? The running time difference between 1-
2 minutes of other tools and AccuVir requiring >30min is surprising.

Thank you for your careful review and valuable suggestion. We have reviewed our code for running Accuvir and confirmed that it takes longer on some datasets due to the numerous search operations. Accuvir employs a local search strategy (beam search) to generate multiple candidate paths and selects the highest-scoring one as the output. To ensure that the candidate paths include a high-quality option, Accuvir uses strategies involving multiple beam search operations, which increases the runtime. While we attempted to adjust parameters to reduce the search space, doing so resulted in faster execution but produced lower-quality output paths compared to the default settings. Therefore, we chose not to reduce the search space for Accuvir, accepting the longer running time.

(18) [Evaluation of computing resource usage]

Powered by Editorial Manager® and ProduXion Manager® from Aries Systems Corporation

"As shown in Figure 4, assemblers relying on alignment graph construction tend to require
more memory" → Again, I think this is supposed to be Figure 5

Thank you for catching this inconsistency. We have corrected this reference in the revised manuscript.

Response to Reviewer 2's comments

The authors describe a new reference-guided assembly tool designed for viral genomes. The authors validate their assembly approach and compare with similar tools. The paper is generally well written, and the tool would be valuable to the community.

Thank you for your summary. We really appreciate your time in reviewing our manuscript. We have revised the manuscript accordingly. Our point-by-point responses are detailed below.

The tool is presented as an alternative to de novo assemblers. However, I am interested to know if PVGA was able to correct errors that were present in assemblies that were generated de novo e.g. from Flye and Canu. It seems like PVGA would serve equally well as either a reference-guided assembly tool or as an assembly polishing tool.

Thank you for your question. In the originally submitted manuscript, under the section "Benchmarking the capability of polishing" , We have already evaluated PVGA's polishing ability to correct errors introduced by De Novo assemblers like Canu. We first simulated the SARS-CoV-2 Illumina reads with lengths of 200-300 base pairs and coverages of 25x for polishing the Canu's assembly result. Also, we have expanded in the revised manuscript (Section "Convergence performance across different backbones") by assembling the HIV-1 JRCSF strain using Flye-assembled results as the backbone. When reapplying PVGA with the same reads used in Flye, PVGA perfectly eliminate the edit distance of 20 introduced by Flye, reconstructing the ground-truth genome.

My main concern is a lack of information on the minimum similarity requirements of the reference genome. The quality of the final assembly is entirely dependent on the quality of the original assembly. Incomplete coverage or low similarity will impact the final assembly and it would be useful for users to know at what point they should use an alternative approach (such as my suggestion above: de novo assembly followed by PVGA for polishing).

Thanks for your suggestion. We have conducted additional experiments to evaluate the impact of backbone selection on PVGA's algorithmic convergence. Specifically, we tested four distinct HIV-1 strains (89.6, HXB2, NL43, and YU2) as backbones for assembling reads derived from the JRCSF strain in both real and simulated data. We first compared the sequence similarity between these backbone genomes and the ground truth JRCSF genome. To further assess how backbone divergence affects assembly accuracy, we introduced manual random errors (15%, 20%, 25%, and 30%) into the JRCSF genome and used these modified sequences as backbones to assemble simulated reads with 5% error rates. The results (Table 11) demonstrate that even with a 30% error rate in the backbone, PVGA maintained robust assembly accuracy. This is attributed to the backbone contributing only a weight of 1 for base connectivity decisions, while read-derived evidence (proportional to coverage) dominated the alignment graph construction. However, higher divergence between the backbone and the ground truth led to shorter assembly lengths, specifically manifesting as fragment loss at the genome ends, while the central regions remained error-free as shown in Table 11. This occurs because regions at the ends of the backbone with higher divergence showed reduced alignment efficiency: reads spanning these

divergent regions failed to align to the backbone and thus could not contribute to the graph construction.

I would strongly recommend adding the tool to bioconda. Can you use the bioconda version of blasr? I believe all the dependencies in requirements should be available on conda. You should also be to publish as a python package.

Thanks for your valuable suggestion, we have already contributed PVGA to bioconda. Now users can install and run the tool seamlessly without manually configuring their environments. The download command is provided below:
conda create --name pvga python=3.10
conda activate pvga
conda install -c bioconda pvga

Misc comments:
"The virus genomes have relatively small size."
All? Some?

Thanks for your suggestion. We have removed this sentence.

"As the basic structure of the virus genomes is highly conserved …"
Do you have a citation for this? As this is a reference-guided assembly tool, the quality of the final assembly is dependent on having adequate structural and sequence similarity to the reference used.

Thank you for your feedback. We have revised the statement to: “As the basic structure of many virus genomes is highly conserved…” The citation supporting this claim is derived from “Conserved RNA secondary structures in viral genomes: a survey” which illustrates that “The genomes of RNA viruses often carry conserved RNA structures that perform vital functions during the life cycle of the virus. See the begging part of Section “Introduction”.

"However, these longer reads tend to have higher error rates."
I don't believe this is nearly as relevant as it would have been 5 years ago. PacBio HiFi reads are very accurate and nanopore is not far behind.

We have revised the statement to “In comparison with NGS，TGS tends to have higher error rates.” In addition, we added discussion on this part in this paper to deepen the necessity of using assembler for high error rate reads’ assembly in “Evaluation on poor sequencing conditions” section as follows：
“For instance, PacBio sequencing technology can offer HiFi reads that provide an accuracy of 99.9\%. However, some laboratories continue to rely on older sequencing equipment or encounter suboptimal results due to experimental limitations. In such cases, there is a need for an assembler capable of effectively handling data with relatively higher error rates.”
See the begging part of the subsection “Evaluation on poor-sequencing conditions”

"Famous reference-guided assembly methods include BWA [11], Bowtie2 [12], GATK [13], Novoalign [14]..."
These are all read alignment programs if I'm not mistaken.

Thanks for your valuable suggestion. We have carefully reviewed your comments and agree that the tools mentioned (BWA, Bowtie, and GATK) are primarily designed for read mapping and variant calling rather than reference-guided assembly. While these tools are often used as part of the preprocessing steps for reference-guided assembly, they do not directly produce consensus sequences or contigs, which are the key outputs of an assembly process. We have removed the description of these tools from the section on reference-based assembly. Instead, we now focus on tools specifically designed for reference-guided assembly. The comparison before and after modification is as follows:

Origin: “Famous reference-guided assembly methods include BWA, Bowtie2, GATK, Novoalign, and Maq.”

Powered by Editorial Manager® and ProduXion Manager® from Aries Systems Corporation

	Revised: “Famous reference-guided assembly methods include Novoalign, Maq, iVar, Accuvir and bcftools.”<br><br>"For instance, IVA was developed as a De Novo assembler for RNA viruses, utilizing paired-end datasets to achieve more accurate assemblies [16]. Similarly, Accuvir introduced a reference-based long-read assembler for viruses, primarily employing diverse beam search algorithms on alignment graphs to improve accuracy [17]."<br>You could expand on how the PVGA approach compares with these other established tools.<br><br>Thanks for your comment, we have added the motivation of designing PVGA as follows:<br>“However, despite these advancements, current viral genome assembly tools still fail to achieve the requisite base-level accuracy.” And we had a comprehensive comparison in Results section.<br><br>"PVGA starts with a reference genome and utilizes the sequencing reads directly to reduce noise."<br>Reduce what noise? How?<br><br>The term "noise" in this context refers to the sequence divergence between the backbone genome and the ground truth. PVGA mitigates such noise through an iterative graph refinement process as shown in the section “Results” and the section “Method”.<br><br>"The results demonstrate that PVGA consistently outperforms the best existing programs"<br>I would reword 'the best programs' to something like 'popular existing programs'<br><br>Thanks for your suggestion. We have revised it.<br><br>Figure 1: do the reads get remapped each iteration?<br><br>Yes, as described in the subsection "Updating the Reference Genome Iteratively", the alignment graph is dynamically reconstructed during each iteration by realigning all input reads to the updated backbone genome.<br><br>Is there a hard cap on the number of iterations?<br><br>The upper bound of iteration number is PVGA’s optional parameter. Users can specify an upper limit for the number of iterations via the -n parameter (default: 10).
**Additional Information:**	
**Question**	**Response**
Are you submitting this manuscript to a special series or article collection?	No
**Experimental design and statistics**<br><br>Full details of the experimental design and statistical methods used should be given in the Methods section, as detailed in our Minimum Standards Reporting Checklist. Information essential to interpreting the data presented should be made available in the figure legends.	Yes

Have you included all the information requested in your manuscript?	
**Resources**<br><br>A description of all resources used, including antibodies, cell lines, animals and software tools, with enough information to allow them to be uniquely identified, should be included in the Methods section. Authors are strongly encouraged to cite Research Resource Identifiers (RRIDs) for antibodies, model organisms and tools, where possible.<br><br>Have you included the information requested as detailed in our Minimum Standards Reporting Checklist?	Yes
**Availability of data and materials**<br><br>All datasets and code on which the conclusions of the paper rely must be either included in your submission or deposited in publicly available repositories (where available and ethically appropriate), referencing such data using a unique identifier in the references and in the “Availability of Data and Materials” section of your manuscript.<br><br>Have you have met the above requirement as detailed in our Minimum Standards Reporting Checklist?	Yes
GigaScience has policies and guidelines in place for the use of generative AI-writing tools such as ChatGPT. If you have used such writing tools to assist with writing the manuscript this must be declared and cited in the text. Authors should not list AI-writing tools and other AI-assisted technologies as an author or co-author and should acknowledge that they are fully responsible for text generated or refined by AI-writing tools.<p><br><br>A summary of use (particularly in the introduction or among methods) needs to	No

be included at the end of the paper, and the outputs should also be included as a supplementary file hosted in GigaDB or other open repositories. Please read our guidelines for more information. <p><br><br>By submitting to GigaScience, you are aware of the journal's AI-writing tools policy, and if you have declared use of such tools below, you have acknowledged this where appropriate in your manuscript and have made a summary of use and outputs available. <p><br><b>AI-assisted writing tools have been used in the preparation of this manuscript?	

Powered by Editorial Manager® and ProduXion Manager® from Aries Systems Corporation

main file

Click here to access/download;Manuscript;mainNew.pdf

GigaScience, 2023, 1–12

doi: xx.xxxx/xxxx
Manuscript in Preparation
Paper

PAPER

PVGA: A Precise Viral Genome Assembler Using Iterative Alignment Graph

Zhi Song[1], Dehan Cai[2], Yanni Sun[2,*] and Lusheng Wang[1,*]

[1]Department of Computer Science, City University of Hong Kong and [2]Department of Electrical Engineering, City University of Hong Kong

*Correspondence address. Yanni Sun, Electrical Engineering, City University of Hong Kong, Kowloon, China. Email: yannisun@cityu.edu.hk; Lusheng Wang, Department of Computer Science, City University of Hong Kong, Kowloon, China. Email: cswangl@cityu.edu.hk

Abstract

Background: Viral genome analysis is crucial for understanding viruses evolution and mutation. Investigations into viral evolutionary dynamics and mutation patterns have garnered significant research attention since the outbreak of COVID-19. As the basic structure of many virus genomes is highly conserved [1]. RNA viruses have high mutation rates, and single-nucleotide variations may induce substantial phenotypic alterations in terms of viral function and pathogenicity. Thus, special assembly methods are required for viral genome analysis.
Result: PVGA starts with a reference genome and utilizes the sequencing reads directly to reduce noise. The first step in PVGA involves constructing an alignment graph based on a reference genome and the set of input sequencing reads. Then the optimal genomic path is determined through dynamic programming, maximizing the cumulative edge weights that reflect read support density across the alignment graph. The obtained path corresponds to a refined genome. Finally, we repeat the process by using the new reference genomes until no further improvement is possible. We evaluate PVGA's performance across both assembly and polishing tasks using simulated and real datasets including both long reads and short reads. The experiments demonstrate that PVGA always outperforms popular existing programs in terms of the quality of assembly results, while the running time of our method is compatible to others. In particular, simulated Nanopore datasets show that our method can correctly report the true genomes with 0 mismatch and 0 indels.
Conclusions: PVGA is a novel viral genome assembler that seamlessly integrates assembly and polishing into a unified workflow. Its design prioritizes high accuracy, enabling the detection of subtle genomic variations that can impact viral function and pathogenicity. By addressing the unique challenges of viral genome assembly, PVGA provides a reliable and precise solution for advancing our understanding of viral evolution and behavior.

Key words: Genome assembler; Virus genome; Alignment graph; Maximum total weight path; Iterative method

Introduction

Viral genome analysis is crucial for understanding viruses evolution and mutation. Investigations into viral evolutionary dynamics and mutation patterns have garnered significant research attention since the outbreak of COVID-19. As the basic structure of many virus genomes is highly conserved [1]. RNA viruses have high mutation rates, and single-nucleotide variations may induce substantial phenotypic alterations in terms of viral function and pathogenicity. For example, genetic mutations in the coronavirus genome that alter the spike protein can affect its ability to interact with host cells, thereby affecting transmissibility and disease severity [2]. Thus, special assembly methods are required for viral genome analysis.

Sequencing of viral genomes primarily relies on Next-Generation Sequencing (NGS) and Third-Generation Sequencing (TGS) technologies. NGS is recognized for its short read lengths and high accuracy, with platforms such as Illumina. However, due to the short read lengths and the presence of repetitive regions, it is often challenging to assemble genomes accurately using NGS data. Third-generation sequencing (TGS) technologies,

Compiled on: March 15, 2025.
Draft manuscript prepared by the author.

such as Nanopore and PacBio, are effective for resolving complex genome structures and repetitive regions due to their longer read lengths. In comparison with NGS, TGS tends to have higher error rates. For instance, Nanopore sequencing relies on measuring electrical current changes as DNA passes through a nanopore, and factors such as pore condition, molecule speed, and signal noise can interfere with base-calling accuracy, making it difficult to distinguish adjacent bases, thereby reducing the quality of the reads [3]. PacBio sequencing, using its Single Molecule Real-Time (SMRT) technology [4], is also widely applied for TGS. SMRT sequencing is well-suited for detecting structural variations and resolving repetitive regions, but it frequently introduces insertion and deletion (indel) errors. To improve accuracy, PacBio introduced HiFi(high-fidelity) sequencing, which produces highly accurate long reads by repeatedly sequencing the same molecule [5]. However, this increased accuracy comes at a higher cost. Achieving high-quality viral genome assembly requires balancing sequencing accuracy with cost. Both the choice of sequencing technology and the assembly algorithms play critical roles in producing reliable genome assemblies.

Genome assembly techniques are broadly categorized into two types: De Novo assembly and reference-guided assembly. De Novo assembly tools include Velvet [6], ABySS [7], SPAdes [8], Flye [9], Canu [10] and Translig [11], which reconstructs the genome without relying on a reference genome. However, De Novo methods often encounter challenges in highly repetitive regions, which may lead to misassemblies, redundant contigs, or gaps. Moreover, in regions of low coverage, De Novo assembly may produce incomplete or missing sequences, and further introduce gaps and errors. With the help of the reference genome sequence, one can obtain the locations of reads in the genome. Thus, reference-guided assembly methods can possibly fill gaps between reads and improve prediction accuracy in low-coverage regions. Famous reference-guided assembly methods include Novoalign [12], Maq [13], iVar [14], Accuvir [15] and bcftools [16].

There are some assemblers that are optimized for viruses. For instance, IVA was developed as a De Novo assembler for RNA viruses, utilizing paired-end datasets to achieve more accurate assemblies [17]. Similarly, Accuvir [15] introduced a reference-based long-read assembler for viruses, primarily employing diverse beam search algorithms on alignment graphs to improve accuracy. In addition to assembly tools, genome polishing methods have become increasingly important for enhancing assembly accuracy by correcting errors using high-accuracy reads. Pilon [18] improves genome assemblies by analyzing read alignments, constructing a pileup structure to evaluate base-level evidence, and iteratively adjusting the assembly based on read quality and consistency. NextPolish [19] employs a combination of alignment-based error correction and iterative consensus polishing, utilizing short-read data to precisely correct mismatches and indels, further refining the final genome assembly. However, despite these advancements, current genome assembly tools still fail to achieve the requisite base-level accuracy for viral genome assembly.

In this paper, we present PVGA, a novel viral genome assembler that can perform both assembly and polishing, effectively handling both long-read and short-read sequencing data. PVGA starts with a reference genome and utilizes the sequencing reads directly to reduce noise. The first step in PVGA involves constructing an alignment graph based on a reference genome and the set of input sequencing reads. Then the optimal genomic path is determined through dynamic programming, maximizing the cumulative edge weights that reflect read support density across the alignment graph. Finally, we repeat the process by using the new reference genomes until no further improvement is possible.

We evaluated PVGA's performance across both assembly and polishing tasks using simulated and real datasets including both long reads and short reads. The results demonstrate that PVGA consistently outperforms popular existing programs. In particular, simulated Nanopore datasets show that our method can correctly report the true genomes with 0 mismatch and 0 indels, except for some small errors at the two ends of the genomes.

Methods

Our new method contains three steps. Step 1: We construct an alignment graph based on the set of input reads using an initial reference genome as the backbone. The initial reference genome should be from the same species. One can also generate an initial reference genome using an existing De Novo assembler. Step 2: After constructing the alignment graph, we apply a dynamic programming algorithm to select a path supported by the largest number of read coverage and construct a new reference genome based on the path. Step 3: We then use the latest reference genome as the backbone to repeat Steps 1-2. The process stops when the new reference genome is identical to the old one.

Alignment graph construction

The graph construction method is inspired by the hierarchical genome-assembly process (HGAP) proposed by Chin [20]. The input contains two parts: read sequences and a backbone sequence. First, we construct the initial graph G_b with n nodes $v_1, v_2, \ldots v_n$ and $n-1$ edges based on the backbone sequence $S = s_1 s_2 \ldots s_n$, where each node v_i is labeled with the letter s_i and there is an edge (v_i, v_{i+1}) connecting the two consecutive nodes. We then align each read $R = r_1 r_2 \ldots r_k$ with the reference sequence G_b.

In the alignment, if r_i is aligned to an identical letter s_j in the backbone sequence, then r_i corresponds to the existing node v_j in the graph G_b. The weight of the edge (v_{j-1}, v_j) will be incremented by one, where v_{j-1} is the predecessor node of v_j. If r_i is aligned with a space or a letter s_j not identical to r_i, we will create a new node u_i labeled with r_i and add an edge (u_{i-1}, u_i) with weight 1, where u_{i-1} is the node corresponding to the previous letter r_{i-1}. See Fig. 1(b).

We will repeat the above process until all reads have been applied. The obtained intermediate graph is denoted as $G_I = (V, E)$. In the intermediate graph G_I, each edge is assigned a weight of 1, representing the number of supporting reads. To reduce the complexity of G_I, we merge nodes with the same label and the same parent repeatedly. When merging nodes into a new node u' with its predecessor v, the weight of the resulting edge (v, u') is updated to reflect the total number of supporting reads. Finally, if there are multiple edges between any two nodes u and v in G_I, we combine them into a single edge and update the weight accordingly. This process results in a simplified final alignment graph G.

Finding a directed path in the alignment graph with maximum total weight

The weight on each edge is the number of supporting reads. In order to find a new reference sequence, we will try to find a path in G containing the maximum total weight. Such a path is the path supported by the largest number of reads.

We do a topological sorting on the set of nodes in G and obtain a linear order among the set of nodes in G. Let DP[v] denote the maximum total weight of paths ending at node v. The value of DP[v] can be computed as follows.

$$DP[u] = \max_{v \in Pred(u)} \{DP[v] + w(v, u)\}, \quad (1)$$

where $w(v, u)$ is the weight of the edge from node v to node u. We can compute all the DP[v]s according to the topological order. After that, we will find a node v with the largest DP[v] value and use a standard backtracking process to get a path with maximum total weight on G ending at v.

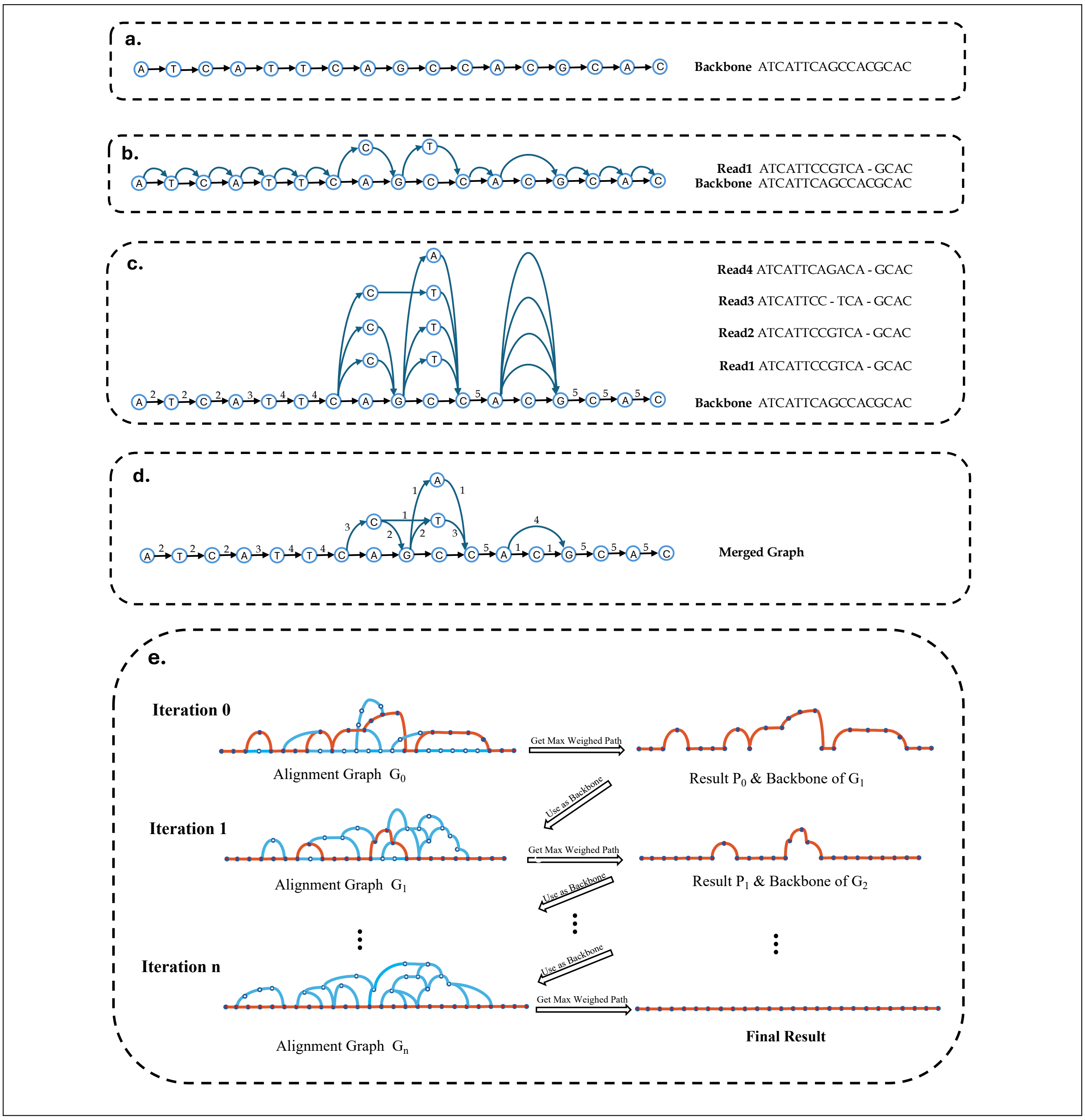

Figure 1. Flowchart of construction of alignment graph and iteration process. **a** PVGA takes a reference Genome as backbone graph G_B.
b PVGA aligns the first read Read1 to the backbone.
c Four reads are aligned with the backbone, awaiting the subsequent merging process.
d PVGA merges edges that point to the same node; the new edge's weight is equal to the sum of the weights of the merged edges. This process can be performed either after aligning all reads or during the alignment process, with a final merge conducted after all reads have been aligned.
e Iteratively construct the alignment graph using the result from the previous iteration as the backbone.

Running time: To compute each $DP[u]$ in equation (1), we need $O(Deg(u))$ time, where $Deg(u)$ is the in-degree of u. Therefore, the total running time complexity of this dynamic programming algorithm is $O(|E|)$, where $|E|$ represents the total number of edges in the graph.

Updating the reference genome iteratively

The quality of the obtained maximum weight path heavily depends on the initial reference genome, as errors or biases in the initial reference can impact the alignment and subsequent path computation. To address this, we use the obtained maximum weight path as a new reference genome. A new alignment graph is then reconstructed using all the input reads and the updated reference genome. The maximum weight path is computed again based on this updated graph. This process is repeated iteratively to refine the assembly. With each iteration, the alignment graph and the resulting path become more accurate and consistent. Iteration stops when the genome obtained from the current iteration is identical to the genome from the previous iteration. This indicates that the backbone and the assembly result have reached a consistent state and no further adjustment is possible using this method. This condition for algorithm termination seems to be very strong and one may worry about the running time of this condition.

Recall that PVGA is for virus genome assembly and the genome size is relatively small. Experimental results indicate that our method exhibits rapid convergence, often requiring no more than

3 or 4 iterations to reach a stable solution in practical applications. The running time comparison is in Figure 5. Moreover, this iterative method can reach high-quality results and experiments show that our iterative method always outperforms the state-of-art methods.

Results

Datasets

We evaluate PVGA using both simulated and real viral sequencing data. To simulate a broad spectrum of sequencing conditions, we use Badread [21], which is designed for generating various kinds of simulated genomes. All real genome data and real sequencing reads used in this study are sourced from the National Center for Biotechnology Information (NCBI). These datasets include the following viral strains.

HIV-1:
HIV-specific data provides a critical benchmark for evaluating assembly methods, given its widespread availability in public databases. We use the 89.6 strain (GenBank: U39362.2) as the target genome, and generate simulated reads in bulk, with sizes detailed in the next subsection, to serve as the ground truth. The HXB2 strain (GenBank: K03455.1) is selected as the reference genome for constructing the initial alignment graph, which shares 93.58% similarity with the ground truth 89.6 strain.

The haplotype benchmarking dataset [22] contains mixed PacBio reads from five strains (HXB2, 89.6, JR-CSF, NL4-3, YU-2). To obtain reads from a single strain, we first align the reads to the genomes of these strains using minimap2 [23]. We then extract the reads based on their closest aligned genome (identified by samtools [24]).

SARS-CoV-2:
SARS-CoV-2 sequencing data is characterized by extensive genomic length and high sequence homology. We select SARS-Cov-2 isolate Wuhan-Hu-1 (NCBI Reference Sequence: NC_045512.2) as the reference genome and SARS-Cov-2 isolate (GenBank: OZ072292.1) as a target and to generate simulation reads.

Norovirus:
Noroviruses are common pathogens that can cause acute gastroenteritis. We obtain third-generation nanopore sequencing data of noroviruses(SRX10330013) from the National Food Virology Reference Centre at Health Canada [25]. This dataset consists of 5,741 spots, totaling 2.9 million bases, with the norovirus GII strain BMH19-097 serving as the ground truth genome. We use the complete genome of Norovirus GII (NC_039477.1) as the reference for graph construction. We also employ actual Illumina sequencing data of the norovirus. We utilize SRR13951201 (35.8M bases), SRR13951221 (60M bases), and SRR13951199(12.9M bases) as input reads, with corresponding ground truth data from Norovirus GII isolates BMH19-145, BMH13-039 and BMH14-056.

Ebola:
We utilize two Ebola virus (EBOV) genomes. The first genome corresponds to the Ebola virus (EBOV-May) Mayinga strain, isolated in Zarie in 1976. This genome consists of 18,959 base pairs and is publicly available under the NCBI accession number AF086833.2. EBOV-May is a negative-sense, single-stranded RNA virus of the genus Orthoebolavirus, encoding seven structural proteins such as nucleoprotein (NP), glycoprotein (GP), and RNA-dependent RNA polymerase (L) [26, 27]. The second Ebola virus strain is isolated from Macaca fascicularis and sequenced using IonTorrent technology. The genome, consisting of 18,871 base pairs, is publicly accessible under the NCBI accession number KY786027.1. It was assembled using the CLC Genomics Workbench v9.5.4 and serves as the ground truth genome for generating synthetic reads in this study. The metadata associated with this genome are part of BioProject PRJNA379115 and BioSample SAMN06603499, with additional information submitted by Guedj et al. [28]

Measles:
Measles virus (MV), a member of the genus Morbillivirus in the family Paramyxoviridae, is a highly contagious, negative-sense, single-stranded RNA virus. We utilize two Measles virus strains. The first genome, a complete reference genome of Measles morbillivirus, was sourced from the NCBI RefSeq database (accession number NC_001498.1). This genome consists of 15,894 base pairs. The second genome is the Measles virus genotype A transgenic strain vac2(GFP)H and serves as the ground truth genome for generating synthetic reads. This genome, available under the GenBank accession number MH144178.1, spans 16,728 base pairs and includes a transgenic insertion of the green fluorescent protein (GFP) gene. It was sequenced using Sanger dideoxy sequencing and has been used in experimental studies for functional and structural analyses [29, 30].

Evalution on simulation data

We evaluate the performance of several assemblers using simulated Nanopore and PacBio data. Tests are conducted on two viral strains, HIV-1, and SARS-CoV-2, under both standard and low coverage conditions to assess their robustness.

We compare our method PVGA with the state-of-the-art methods, including De Novo methods: Flye [9], Canu [10] and reference-based methods: Accuvir [15], PBDAG-Con [20] and Medaka [31]. In addition, to illustrate the improvement achieved by the iterative step of PVGA, we also present the results of our method without the iterative step (referred to as "PVGA (no_iter)")

We evaluate the assembly quality using several metrics, including genome fraction, mismatches, indels, indel length, and edit distance. Genome fraction represents the percentage of reference genome bases accurately matched by the assembled genome. Mismatches refer to the number of positions where the nucleotide in the assembly differs from the reference sequence. Indels refer to the number of insertions and deletions in the assembly relative to the reference. Indel length refers to the total length of the indels. Edit distance defines a minimum number of substitution and indel operations required to transform the assembled genome into the reference sequence. These metrics collectively measure the assembly's accuracy and its deviation from the ground truth genome.

Benchmarking on Standard-Length and Depth HIV-1 Simulated Data
As the genome length increases, all methods tend to show higher error rates. However, PVGA consistently outperforms the compared assemblers. For our simulations, the average lengths of reads for Nanopore and PacBio were set at 2k, 4k, and 6k, with sequencing depths of 50x, 100x, and 200x.

To simulate sequencing data, we used the Badread tool with the error model parameter set to nanopore2023, which was trained on ONT R10.4.1 reads. The identity was set to (95, 99, 2.5), indicating a normal distribution with a mean of 95 and a standard deviation of 2.5. For PacBio sequencing, we set the error model in Badread to pacbio2021, trained on PacBio Sequel II HiFi reads. We use the same identity settings as (95, 99, 2.5). The results for Nanopore are presented in Figure 2 and Table 1, while the results for PacBio are shown in the supplementary materials.

From Table 1, we can see that PVGA achieves 0 mismatches and 0 indels with an edit distance of 3 across all Nanopore test cases. Figure 2, illustrates similar cases, where the average lengths of reads are 2kb and 4kb, respectively.

The PacBio results are illustrated in the supplementary document Section. As shown in Table 1 as well as Figure 2, the iterative

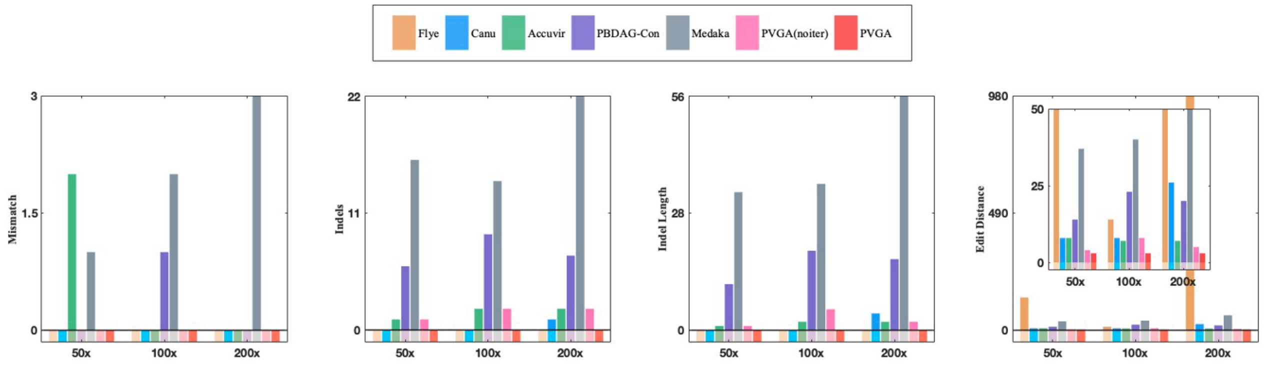

(a) Results on Simulated Nanopore HIV-1 Datasets with 5% Error Rate and 2kb Reads

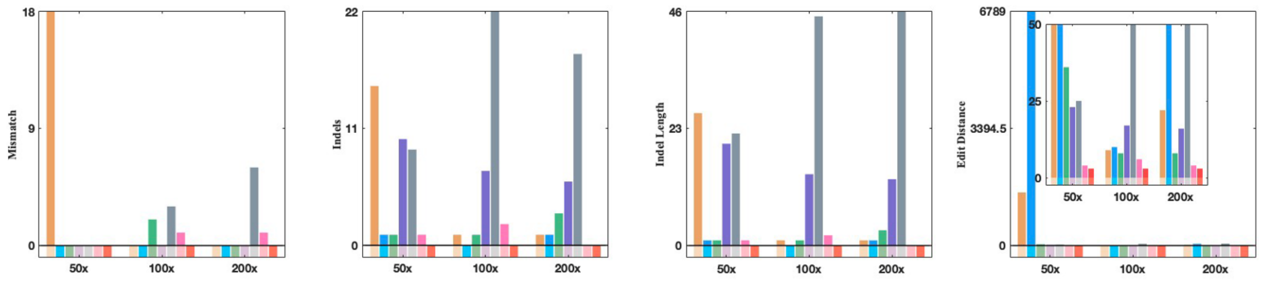

(b) Results on Simulated Nanopore HIV-1 Datasets with 5% Error Rate and 4kb Reads

Figure 2. Results on simulated Nanopore HIV-1 datasets with an average read length of 2kb and 4kb, respectively. The 4 sub-figures in each row represent mismatch, indels, indel length, and edit distance from left to right, respectively.

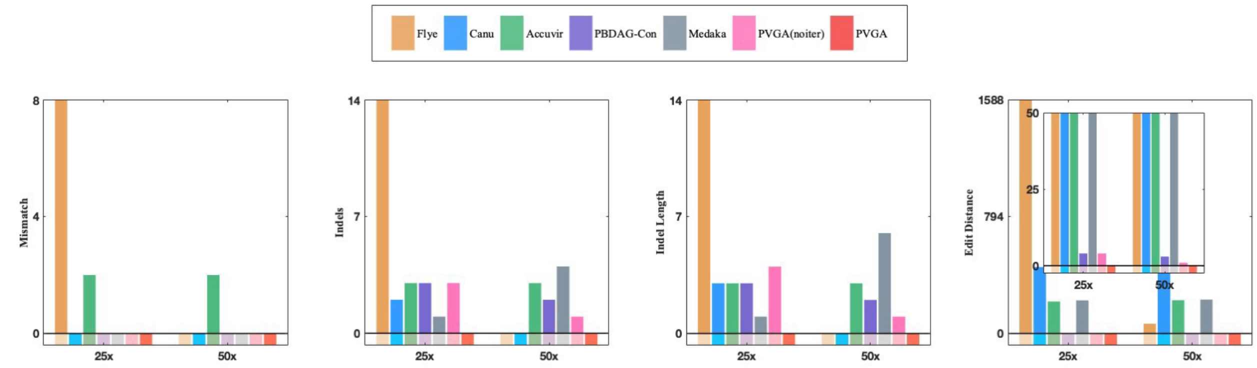

(a) Results on Simulated Nanopore SARS-CoV-2 Datasets with 5% Error Rate and 2kb Reads

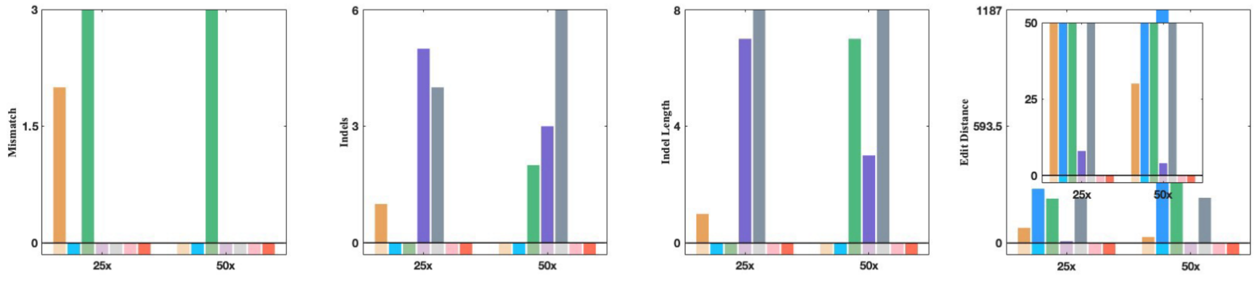

(b) Results on Simulated Nanopore SARS-CoV-2 Datasets with 5% Error Rate and 4kb Reads

Figure 3. Results on simulated Nanopore SARS-CoV-2 datasets with an average read length of 2kb and 4kb, respectively. The 4 sub-figures in each row represent mismatch, indels, indel length, and edit distance from left to right, respectively.

Table 1. Results on Simulated Nanopore HIV-1 (genome length: 9713bp) Datasets with 5% Error Rate and an average read length of 6kb ("-" indicates that the assembler fails to produce a result.)

Reads depth	Tool	Genome fraction	Genome length	Mismatch	Indels	Indel length	Edit distance
50x	Flye	93.143	9067	0	0	0	686
	Canu	100	9748	0	0	0	35
	Accuvir	99.949	9709	0	2	3	4
	PBDAG-Con	99.969	9693	1	7	17	20
	medaka	99.969	9724	2	12	30	35
	PVGA (no_iter)	99.969	9706	0	2	4	7
	**PVGA**	**99.969**	**9710**	**0**	**0**	**0**	**3**
100x	Flye	96.757	9405	0	0	0	322
	Canu	-	-	-	-	-	-
	Accuvir	99.949	9706	0	2	2	7
	PBDAG-Con	99.969	9693	0	8	17	20
	medaka	99.969	9728	1	22	46	50
	PVGA (no_iter)	99.969	9709	2	1	1	6
	**PVGA**	**99.969**	**9710**	**0**	**0**	**0**	**3**
200x	Flye	100	9734	0	1	1	23
	Canu	-	-	-	-	-	-
	Accuvir	99.959	9710	0	1	1	5
	PBDAG-Con	99.969	9696	0	6	14	17
	medaka	99.969	9732	7	26	68	77
	PVGA (no_iter)	99.969	9705	1	5	5	9
	**PVGA**	**99.969**	**9710**	**0**	**0**	**0**	**3**

Canu could not construct the assembly graph required for genome assembly, when the reads dataset lacks sufficient independent reads and effective overlaps.

refinement process enhances assembly accuracy. For instance, with 6k read lengths and a depth of 200x, the process reduces indels from 5 to 0, mismatches from 1 to 0, and the edit distance from 9 to 3. The experiments show that increasing coverage improves performance for some assemblers. For PVGA, a coverage of 50x is sufficient to achieve 0 mismatches and 0 indels.

Benchmarking on Low Coverage HIV-1 Simulated Data

Sequencing costs increase with coverage. An efficient assembler should perform well not only for high coverage but also under lower coverage conditions to support cost-effective sequencing. Thus, we further test the cases, where the coverage are 30x, 25x, and 20x, respectively, with an average read length of 2kb. See Table 2.

Table 2. Results on simulated Nanopore HIV-1 (genome length: 9713bp) datasets with low coverage: 30x, 25x and 20x

Reads depth	Tool	Genome fraction	Genome length	Mismatch	Indels	Indel length	Edit distance
30x	Flye	70.751	6917	0	1	1	2888
	Canu	93.452	11602	0	1	1	4147
	Accuvir	99.938	9707	1	3	4	11
	PBDAG-Con	99.969	9695	0	7	15	18
	Medaka	99.969	9714	1	9	18	22
	PVGA (no_iter)	99.969	9706	0	1	4	7
	**PVGA**	**99.969**	**9710**	**0**	**0**	**0**	**3**
25x	Flye	77.906	7576	0	0	0	2155
	Canu	93.452	11156	0	1	1	7325
	Accuvir	99.959	9713	3	3	4	11
	PBDAG-Con	99.969	9688	0	8	22	25
	Medaka	99.969	9711	2	8	17	22
	PVGA (no_iter)	99.969	9709	0	1	1	4
	**PVGA**	**99.969**	**9710**	**0**	**0**	**0**	**3**
20x	Flye	96.788	9440	0	1	1	353
	Canu	93.452	11549	0	4	5	5918
	Accuvir	99.949	9708	2	2	2	9
	PBDAG-Con	99.969	9688	0	13	24	27
	Medaka	99.969	9714	0	10	20	23
	PVGA (no_iter)	99.969	9710	0	0	0	3
	**PVGA**	**99.969**	**9710**	**0**	**0**	**0**	**3**

At these lower coverage levels, PVGA continues to show robust performance. As shown in Tables 2, at coverage levels of 30x, 25x, and 20x, while some assemblers experience a noticeable drop in accuracy, PVGA consistently achieves the best performance with 0 mismatches and 0 indels. To figure out the threshold at which the accuracy of PVGA begins to decline, we test the case, where the coverage ranges from 19x down to 15x with an average read length of 2kb. The results are shown in Table 3.

As shown in Table 3, at coverages of 19x, 18x, and 17x, PVGA maintains zero indels. When the coverage drops to 16x, PVGA has its first indel with a length of 1, increasing to 4 at 15x. Despite this, PVGA continues to outperform all other assemblers, with the lowest indel count, indel length, mismatch rate, and edit distance. These results demonstrate PVGA's robustness under low coverage conditions, highlighting its potential to reduce sequencing costs while maintaining accuracy.

Table 3. Results on simulated Nanopore HIV-1 (genome length: 9713bp) datasets with low coverage (<20x)

Reads depth	Tool	Genome fraction	Genome length	Mismatch	Indels	Indel length	Edit distance
19x	Flye	88.963	8673	0	0	0	1104
	Canu	93.452	11853	0	5	6	5044
	Accuvir	99.866	9709	0	1	1	8
	PBDAG-Con	99.969	9682	1	11	28	32
	Medaka	99.969	9707	3	11	17	23
	PVGA (no_iter)	99.969	9707	0	1	3	6
	**PVGA**	**99.969**	**9710**	**0**	**0**	**0**	**3**
18x	Flye	93.926	9140	0	3	3	613
	Canu	99.969	9708	0	2	2	5
	Accuvir	99.959	9711	4	5	6	14
	PBDAG-Con	99.969	9686	0	11	24	27
	Medaka	99.969	9707	1	8	14	18
	PVGA (no_iter)	99.969	9709	0	1	1	4
	**PVGA**	**99.969**	**9710**	**0**	**0**	**0**	**3**
17x	Flye	72.614	7085	0	2	2	2694
	Canu	99.866	10907	0	5	5	1226
	Accuvir	99.753	9713	1	1	2	12
	PBDAG-Con	99.969	9685	0	8	25	28
	Medaka	99.969	9711	0	7	23	26
	PVGA (no_iter)	99.969	9710	0	0	0	3
	**PVGA**	**99.969**	**9710**	**0**	**0**	**0**	**3**
16x	Flye	93.092	9047	0	3	3	682
	Canu	93.452	11805	0	3	3	4126
	Accuvir	99.959	9719	0	7	10	14
	PBDAG-Con	99.969	9696	1	6	14	18
	Medaka	99.969	9707	0	1	3	7
	PVGA (no_iter)	99.969	9710	0	2	2	5
	**PVGA**	**99.969**	**9711**	**0**	**1**	**1**	**4**
15x	Flye	96.87	9402	0	7	7	315
	Canu	94.399	9163	1	6	6	551
	Accuvir	99.856	9693	0	7	8	22
	PBDAG-Con	99.969	9687	2	12	27	32
	Medaka	99.969	9703	3	6	13	19
	PVGA (no_iter)	99.969	9706	0	4	4	7
	**PVGA**	**99.969**	**9706**	**0**	**4**	**4**	**7**

Benchmarking on Simulated SARS-CoV-2 Data

The SARS-CoV-2 virus is one of the RNA viruses with a long genome, approximately 29.9 kb. Although SARS-CoV-2 variants are highly similar, a few differences can lead to distinct biological properties such as pathogenicity, transmissibility, and immune response. Therefore, assembling an accurate SARS-CoV-2 genome is essential. In this section, we conduct experiments on simulated SARS-CoV-2 data at different depths (25x and 50x) and read lengths (2kb, 4kb, and 8kb) for both Nanopore and PacBio datasets to evaluate the performance of tools on a highly similar virus with a long genome. Results for Nanopore are presented in Table 4 and Figure 3, while PacBio results are included in the supplementary document.

Table 4. Results on simulated Nanopore SARS-CoV-2 (length: 29646bp) datasets with 5% error rate and an average read length of 8kb

Reads depth	Tool	Genome fraction	Genome length	Mismatch	Indels	Indel length	Edit distance
25x	Flye	100	29656	0	0	0	10
	Canu	96.711	28669	0	6	11	977
	Accuvir	100	29872	0	3	3	226
	PBDAG-Con	100	29646	0	14	1	2
	Medaka	100	29878	0	2	8	232
	PVGA (no_iter)	100	29644	0	4	4	4
	**PVGA**	**100**	**29646**	**0**	**0**	**0**	**0**
50x	Flye	99.98	29645	0	0	0	11
	Canu	97.834	28999	0	2	5	647
	Accuvir	100	29869	0	3	3	223
	PBDAG-Con	100	29672	0	0	0	26
	Medaka	100	29646	0	5	11	235
	PVGA (no_iter)	100	29643	0	0	0	3
	**PVGA**	**100**	**29646**	**0**	**0**	**0**	**0**

Table 4 shows that both PVGA and Flye achieve zero indels and mismatches. However, PVGA demonstrates superior performance, reconstructing the genome flawlessly, with no errors even at the two ends of the genome.

Evaluation on poor sequencing conditions

With the advancement of sequencing technologies, there has been a significant leap in both the capabilities and quality of sequencing. For instance, PacBio sequencing technology can offer HiFi reads that provide an accuracy of 99.9%. However, some laboratories continue to rely on older sequencing equipment or encounter suboptimal results due to experimental limitations. In such cases, there is a need for an assembler capable of effectively handling data with relatively higher error rates. A study by the MinION Analysis and Reference Consortium reported that the median total error of all 2D reads was 12%, with 2D pass reads showing a slightly lower error rate of 10.5% [32]. Additionally, after basecalling, the global error rate of raw reads is typically around 10% [33].

To simulate poor sequencing conditions, we configured the following parameters for evaluating assembler performance under suboptimal data quality: We apply a truncated normal distribution of basecall identity (range: 85–95%, mean: 90%, SD: 5%), resulting in an average read error rate of 10% and an upper accuracy bound of 95%. We assign uniform sequencing depth (30×) and average read length (4 kb) across all viral genomes (HIV-1, Measles, Ebola) to standardize suboptimal quality conditions. The results are shown in Table 5.

Table 5. Results on simulated Nanopore HIV-1, Measles, Ebola virus datasets with 10% error rate and an average read length of 4kb with an average depth of 30x

Virus	Tool	Genome fraction (%)	Genome length	Mismatch	Indels	Indel length	Edit distance
HIV-1 (Length: 9713bp)	Flye	100	9732	0	16	17	53
	Canu	99.053	16468	0	36	43	6993
	Accuvir	99.835	9683	0	14	14	30
	PBDAG-Con	99.969	9645	1	26	67	71
	PVGA (no_iter)	99.969	9720	1	26	38	42
	**PVGA**	**99.969**	**9717**	**0**	**7**	**11**	**14**
Measles (Length: 16728bp)	Flye	99.988	13899	0	17	20	2829
	Canu	99.815	16674	2	21	23	56
	Accuvir	99.994	16725	4	9	12	17
	PBDAG-Con	94.996	15879	1	10	12	850
	PVGA (no_iter)	100	16733	2	12	17	19
	**PVGA**	**100**	**16734**	**1**	**5**	**8**	**9**
Ebola (Length: 18871bp)	Flye	99.989	18851	1	18	18	21
	Canu	99.862	18824	1	16	21	48
	Accuvir	100	18979	9	26	28	123
	PBDAG-Con	100	18858	0	13	13	13
	PVGA (no_iter)	100	18874	0	12	13	13
	**PVGA**	**100**	**18879**	**0**	**7**	**8**	**8**

For HIV-1, the 89.6 strain is used as the target to simulate reads, with the HXB2 strain serving as the backbone. For the measles virus, the NC_001498.1 sequence (15,894 bp) is used as the backbone, while the vac2(GFP)H sequence (Length: 16,728 bp) served as the target. For the Ebola virus, the EBOV-May strain (18,959 bp) is used as the backbone, with the KY786027 strain serving as the ground truth for read simulation.

Evaluation on real data

Although there are many available long-read sequencing datasets of viruses, most of them lack ground-truth genomes for validation. Thus, we use Norovirus and HIV-1 to evaluate the tools' performance, as they have ground-truth genomes from both long-read and short-read sequencing data.

For the HIV-1 real datasets, we collect PacBio sequencing data from a mock HIV-1 community [22]. To create the datasets for viral genome reconstruction, we separate this dataset into read sets from five HIV-1 strains, by aligning them to the ground-truth genomes using the best hit. We test the 89.6, JR-CSF, and YU-2 subtypes, using HXB2 as the backbone to construct the alignment graph. Given that real data often contains gaps between reads, most De Novo assemblers fail to achieve consensus or produce only very short contigs. As shown in Table 6, as for the 89.6 Strain, Canu produces a contig of only 4,593 base pairs. Flye, on the other hand, encounters errors during real data processing, resulting in an unsuccessful assembly. Due to the lower quality of real reads, assemblers display higher mismatches, indels, and edit distances in the HIV-1 89.6 strain than observed in simulations. However, PVGA still outperforms all other assemblers. In the JR-CSF results, apart from Canu, which failed to assemble a complete genome, only PVGA and PBDAG-Con maintained single-digit mismatches, with PVGA showing lower indels, indel length, and edit distance.

Table 6. Results on real HIV-1 strain datasets(89.6, JR-CSF, YU-2)

Strain	Tool	Genome fraction	Genome length	Mismatch	Indels	Indel length	Edit distance
89.6 Strain (Length: 9713bp)	Canu	47.287	4593	6	0	0	5127
	Accuvir	99.990	9710	33	4	4	30
	Medaka	99.856	9733	25	6	22	74
	PBDAG-Con	100	9711	24	2	2	40
	PVGA (no_iter)	100	9709	25	2	2	36
	**PVGA**	**100**	**9710**	**24**	**1**	**1**	**28**
JR-CSF (Length: 9535bp)	Canu	88.512	8448	6	1	1	1320
	Accuvir	99.727	9736	36	3	3	241
	PBDAG-Con	99.99	9720	5	8	20	221
	Medaka	97.735	9535	33	8	42	316
	PVGA (no_iter)	99.99	9610	3	1	1	159
	**PVGA**	**99.99**	**9610**	**3**	**1**	**1**	**159**
YU-2 (Length: 9706bp)	Canu	86.750	8473	5	2	4	1343
	Accuvir	99.727	9713	5	5	9	16
	PBDAG-Con	100.000	9705	4	7	19	23
	Medaka	100.000	9698	28	9	52	80
	PVGA (no_iter)	100.000	9615	3	9	13	16
	**PVGA**	**100.000**	**9617**	**3**	**7**	**11**	**14**

We also test our method on real norovirus data (SRX10330013). As shown in Table 7, our method PVGA results in the fewest mismatches, indels, and the lowest edit distance among the five assemblers evaluated. This demonstrates that our PVGA method more effectively utilizes information from the alignment graph compared to PBDAG-Con, which focuses on assigning scores to nodes to maximize consensus, and Accuvir, which employs diverse beam search. While a diverse beam search approach increases the diversity of candidate paths, it often falls into local optima, failing to achieve the best results.

Table 7. Results on real Nanopore noroviruses (SRX10330013), with ground truth genome as Norovirus GII isolate BMH19-097 (Length: 7618 bp)

Tool	Genome fraction	Genome length	Mismatch	Indels	Indel length	Edit distance
Flye	92.964	7097	0	11	13	577
Canu	99.593	7632	0	12	14	104
Accuvir	99.396	7564	1	8	10	57
PBDAG-Con	99.383	7562	1	7	9	57
**PVGA**	**99.383**	**7569**	**0**	**4**	**4**	**51**

To assess the effectiveness of the PVGA method with extensive short-read datasets, we employ actual Illumina sequencing data of the norovirus. The Norovirus GII complete genome (NC_039477.1) serves as the reference framework for graph construction. We utilize SRR13951201, SRR13951221, and SRR13951199 as input reads, with corresponding ground truth data from Norovirus GII isolates BMH19-145, BMH13-039, and BMH14-056. The results are in Table 8. In all three Illumina norovirus datasets, PVGA achieves exceptional accuracy, with no mismatches, indels, or indel length errors except for some small misalignment at the two ends of the genomes. This verifies PVGA's excellent performance in assembling short-read datasets as well.

Table 8. Results on real Illumina norovirus

Reads	Genome fraction	Genome length	Mismatch	Indels	Indel length	Edit distance
SRR13951201	100	7572	0	0	0	2
SRR13951221	100	7567	0	0	0	17
SRR13951199	99.574	7485	0	0	0	20

Ground truth genome lengths: SRR13951201 (BMH19-145): 7,570 bp; SRR13951221 (BMH13-039): 7,550 bp; SRR13951199 (BMH14-056): 7,505 bp.

Benchmarking the capability of polishing

The results from low-coverage HIV-1 sequencing data indicate that, at extremely low depths, some De Novo assemblers, such as Flye and Canu, fail to obtain near-optimal solutions. Comparisons with the ground truth reveal that these assemblers do not achieve complete reconstruction in terms of both length and accuracy. For instance, at a coverage of 15x, Flye shows a high edit distance of 315, and Canu has a high edit distance of 551. These errors considerably compromise the accuracy of the assembly, adversely impacting subsequent tasks, such as protein structure prediction [34], etc.

To address these inaccuracies, the next essential step is to polish the assembled sequences to enhance their accuracy. A common approach is to use hybrid methods that integrate high-quality short reads, such as those from Illumina, with flawed assemblies. Polishing tools such as Pilon [18], which utilizes a mapping-based method, align these short reads to the assembly and apply a Bayesian model to determine the most accurate sequence by considering base quality scores and error frequencies. Another tool, NextPolish [19], similarly aligns short reads and uses an iterative process to correct errors in small regions of the assembly.

To evaluate the genome polishing performance of PVGA against NextPolish and Pilon, we conduct experiments to polish the assembly results generated by the PVGA and De Novo assembler Canu in previous experiments, respectively. The first involves a 9,706 bp HIV-1 genome assembled by PVGA from 15× coverage Nanopore reads (2 kb simulated read length, see Table 3), while the second consists of a 29,493 bp SARS-CoV-2 genome generated by Canu using 50× coverage long reads (2 kb average length, Figure 3).

We simulate Illumina short reads via Badread by setting parameter qscores as ideal (read length range 200-300 bp), with coverage parameters specifically calibrated: 15× for HIV-1 and 25× for SARS-CoV-2. The results in Table 9 demonstrate that PVGA outperforms NextPolish and Pilon in terms of polishing.

Table 9. Polished results of 15x coverage HIV-1 simulation data and 25x coverage SARS-CoV-2 simulation data

Virus	Tool	Genome fraction	Genome length	Mismatch	Indels	Indel length	Edit distance
HIV-1 (Length: 9713bp)	NextPolish	99.969	9722	2	12	12	17
	Pilon	99.969	9707	0	3	3	6
	**PVGA**	**100**	**9710**	**0**	**0**	**0**	**3**
SARS-CoV-2 (Length: 29646bp)	NextPolish	99.906	29636	2	26	28	58
	Pilon	99.906	29555	0	63	63	91
	**PVGA**	**99.906**	**29619**	**0**	**3**	**3**	**31**

Convergence performance across different backbones

In this subsection, we investigate how the choice of backbone influences the resulting genome assembly. We simulate the HIV-1 JRCSF strain nanopore reads with an average read length of 4 kb and coverage of 30x for the assembly. The error rate of simulation JRCSF reads is 5%. To evaluate the impact of different backbone sequences, we use four well-characterized HIV-1 reference genomes, including strains 89.6, HXB2, NL43, and YU2 as backbones. In addition, we also apply the De Novo assembly tool Flye [9] on our simulated reads and the obtained genome sequence is also used as the backbone. The Flye-derived backbone has an edit distance of 20 from the ground-truth genome. The similarities between the JRCSF ground truth genome and the various backbones are as follows: 89.6 (91.45%), HXB2 (92.78%), NL43 (92.55%), YU2 (92.83%) and Flye-derived assembly result (99.1%). Here the similarity between the two sequences is 1 - edit distance over the length of the ground truth genome. The similarities between the five different HIV-1 strain genome sequences are shown in Figure 5. The results are shown in Table 10.

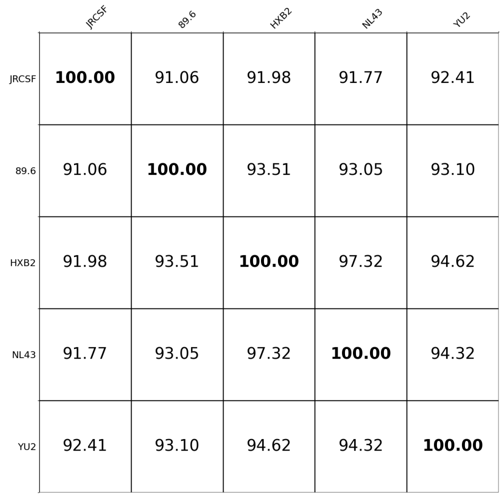

Figure 4. Pairwise similarity matrix of five HIV-1 Strains

Table 10. Results on simulated Nanopore HIV-1 JRCSF Strain reads with 5% error rate on different HIV-1 Strain backbones

Virus	Backbone Genome	Genome fraction	Genome length	Mismatch	Indels	Indel length	Edit distance
JRCSF (Length: 9540bp)	Flye-derived backbone	100	9540	0	0	0	0
	89.6	99.99	9539	0	0	0	1
	HXB2	99.99	9539	0	0	0	1
	NL43	99.99	9539	0	0	0	1
	YU2	99.99	9539	0	0	0	1

The similarities between the JRCSF ground truth genome and the four backbones are as follows: 89.6 (91.45%), HXB2 (92.78%), NL43 (92.55%), YU2 (92.83%), and the Flye assembly genome (99.1%).

From Table 10, we can see that for the backbones derived from 4 different HIV-1 strains, PVGA can obtain the same genome sequence with edit distance 1, and no indel or mismatch. In fact, there is just one missing base at the end of the obtained genome sequence. For the backbone generated by the De Novo assembler Flye, PVGA eliminates the 20-edit-distance discrepancy introduced by the De Novo approach, ultimately recovering the genome sequence identical to the ground truth genome.

To further investigate how the degree of divergence between the backbone and the ground truth impacts assembly results, we modify the HIV-1 JRCSF genome and randomly replacing 15%, 20%, 25%, and 30% of the bases in the HIV-1 JRCSF genome to create backbones with different divergence. For the simulation reads, we maintain a coverage of 30x and an average read length of 4 kb.

Table 11. Results on simulated Nanopore HIV-1 JRCSF Strain reads with different backbones

Virus	Backbone similarity	Genome fraction	Genome length	Mismatch	Indels	Indel length	Edit distance
JRCSF (Length: 9540bp)	85%	99.948	9535	0	0	0	5
	80%	99.937	9533	0	0	0	7
	75%	99.843	9525	0	0	0	15
	70%	99.644	9506	0	0	0	34

As shown in Table 11, with the increase of diversity between the backbone and the ground truth genome, our method can still obtain good results in terms of mismatch, indels, while the edit distance increases and the obtained genome length decreases. Basically, the obtained genome missed some bases at the two ends, but can still perfectly match the ground tree genome in the middle. The reason is that very few reads completely cover the ends, and with the increase of backbone diversity, the few reads covering the ends have a higher possibility of being aligned to the wrong places when constructing the alignment graph.

Evaluation of computing resource usage

We evaluate the CPU runtime and memory usage of PVGA in comparison with several widely used assemblers. The datasets used in the experiments included HIV-1, Measles, and SARS-CoV-2, with sequencing coverages of 50×, 100×, and 200×, and respective genome lengths of approximately 10 kb, 20 kb, and 30 kb. Notably, Accuvir [15] exhibited a runtime exceeding 30 minutes and was therefore excluded from subsequent performance figures. As illustrated in Figure 5, PVGA achieves runtime performance comparable to other mainstream assemblers, though it is not always the best in all scenarios.

Canu and Flye consistently demonstrate the longest runtimes. For Canu, this is primarily due to the computationally intensive processes of read error correction and graph simplification, while Flye's substantial runtime stems from constructing and optimizing the De Bruijn assembly graph. As sequencing coverage increases, the runtime for nearly all assemblers grows proportionally. Interestingly, even with the iterative mechanism, PVGA exhibits lower runtime compared to other assemblers like Canu and Flye. The efficiency gained through iteration primarily stems from the rapid convergence of the process. In the initial iteration, the generated result is already close to the ground truth, providing a more refined backbone for constructing the alignment graph in the next iteration. As the backbone becomes increasingly accurate, subsequent iterations achieve more precise alignments, and the dynamic programming algorithm further optimizes the assembly. This iterative refinement continues until the backbone and the assembled result become identical, ensuring that the assembly process reaches a stable and accurate configuration in a relatively short time.

In terms of memory consumption, we monitor the maximum memory usage during the assembly process across different assemblers. As shown in Figure 5, assemblers relying on alignment graph construction tend to require more memory such as PVGA and PBDAG-Con. This is because PVGA employs a more complex global graph processing approach, which necessitates storing and manipulating a large amount of graph data, leading to higher memory consumption. In contrast, Canu and Flye typically break the input data into smaller chunks, which reduces memory usage and facilitates multi-threaded optimization. However, given the relatively small size of viral genomes compared to those of other species, PVGA's memory consumption remains within a reasonable range for viral genome assembly. All the computing resource experiments are conducted on an Apple M2 chip for evaluation.

Conclusion

PVGA is a powerful virus-focused assembler that does both assembly and polishing. For virus genomes, small changes will lead to huge differences in terms of viral function and pathogenicity. Thus, for virus-focused assemblers, high-accuracy results are crucial. Our approach heavily depends on the input reads as evidence to produce the reported genome. It first adopts a reference genome to start with. We then align all the reads against the reference genome to get an alignment graph. After that, we use a dynamic programming algorithm to compute a path with the maximum weight of edges supported by reads. Most importantly, the obtained path is used as the new reference genome and the process is repeated until no further improvement is possible.

The proposed framework demonstrates robust compatibility with diverse sequencing platforms, achieving nucleotide-level accuracy for both long-read (Nanopore / PacBio) and short-read (Illumina) data modalities. Experiments show that PVGA always outperforms popular existing programs in various cases. In particular, simulated Nanopore datasets show that our method can correctly report the true genomes with 0 mismatch and 0 indels.

Availability of source code and requirements

- Project name: PVGA
- Project home page:
  github: https://github.com/SoSongzhi/PVGA
  workflow DOIs: 10.48546/workflowhub.workflow.1305.1
  biotoolsID: PVGA
  SciCrunch.org databases RRID: SCR_026410
- Operating system(s): Platform independent
- Programming language: Python
- Other requirements: numpy, pysam 0.22.0 or higher, pandas 1.5.2 or higher, Bio 1.7.1 or higher, biopython 1.83 or higher, consensus 1.0.5 or higher, networkx 3.1 or higher, pandas 1.5.2 or higher, python 3.10
- Biotools: QUAST 5.3.0, Badread 0.4.1
- License: MIT License

This needs to be under an Open Source Initiative approved license where practicable compiled running software is made available. If the code is not hosted in a repository the *GigaScience* GitHub repository is also available for this purpose.

Additional Files

Supplementary Table 1. Results on simulated PacBio HIV-1 datasets with a 5% error rate and an average read length of 2kb.
Supplementary Table 2. Results on simulated PacBio HIV-1 datasets with a 5% error rate and an average read length of 4kb.
Supplementary Table 3. Results on simulated PacBio HIV-1 datasets with a 5% error rate and an average read length of 6kb.
Supplementary Table 4. Results on simulated PacBio SARS-CoV-2 datasets with 5% error rate and an average read length of 2kb.
Supplementary Table 5. Results on simulated PacBio SARS-CoV-2 datasets with 5% error rate and an average read length of 4kb.
Supplementary Table 6. Results on simulated PacBio SARS-CoV-2 datasets with 5% error rate and an average read length of 8kb.

Data availability

HIV-1:

- 89.6 Strain complete genome is available in the NCBI database (GenBank: U39362.2)
- HXB2 Strain complete genome is available in the NCBI database (GenBank: K03455.1)
- haplotype benchmarking dataset which cantain mixed PacBio reads from five strains (HXB2, 89.6, JR-CSF, NL4-3, YU-2) [22] is availble in https://github.com/cbg-ethz/5-virus-mix

SARS-CoV-2:

- SARS-CoV-2 isolate Wuhan-Hu-1 is available in the NCBI database (NCBI Reference Sequence: NC_045512.2)

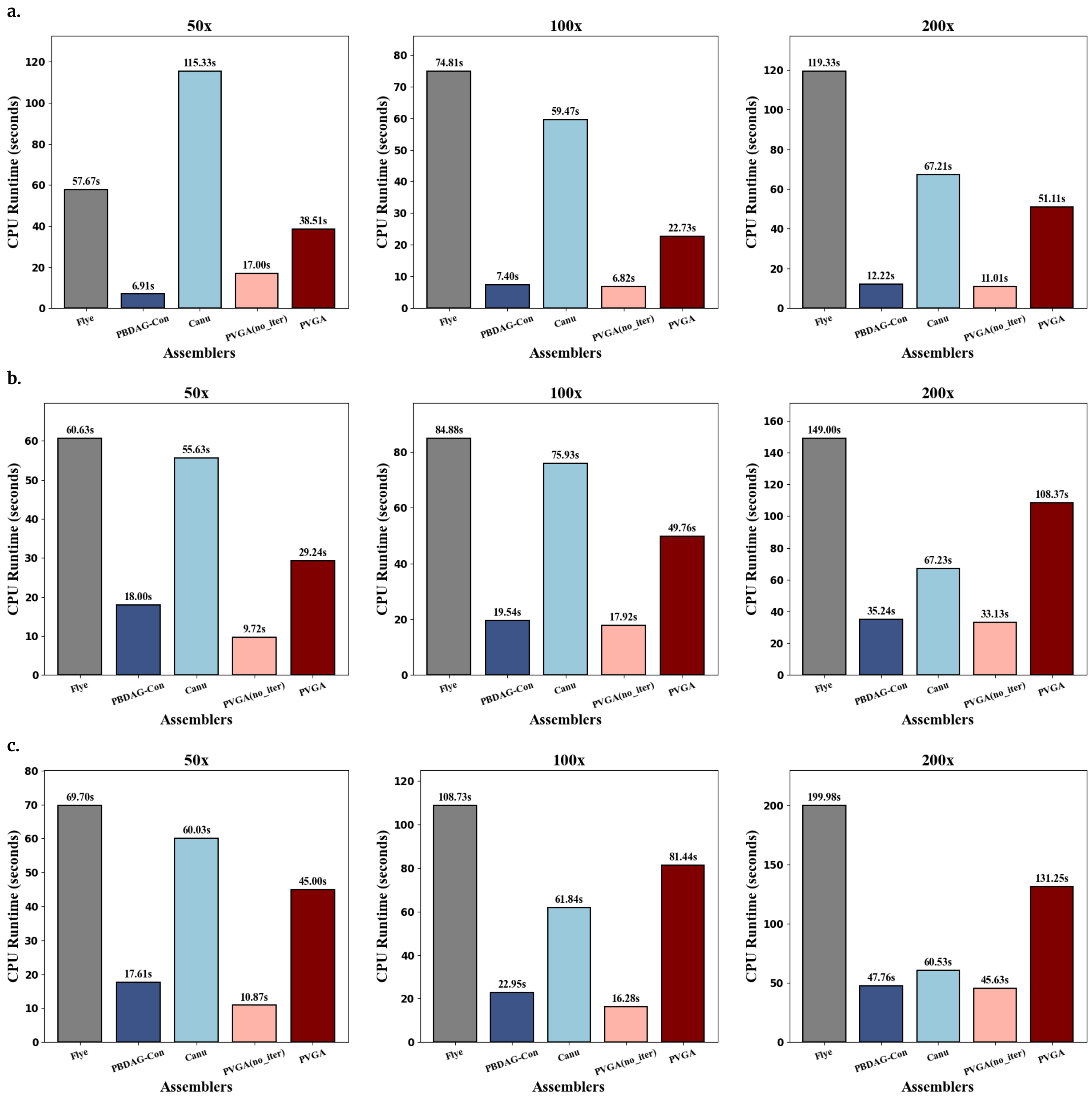

Figure 5. Comparison of CPU times for the five tools on the three datasets of 50x, 100x, 200x coverage respectively, **a.** HIV-1 virus 89.6 Strain, **b.** Measles Virus, **c.** SARS-CoV-2

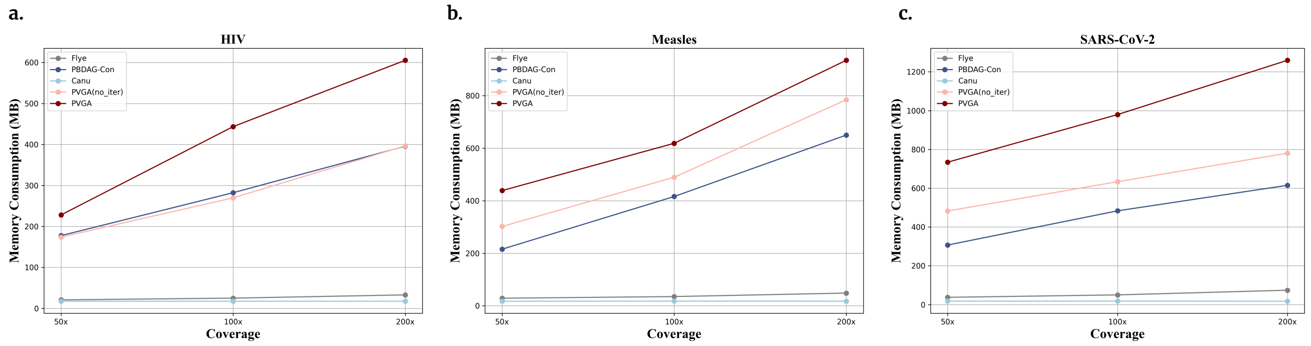

Figure 6. Comparison of maximum memory consumption during the runtime across three datasets with 50×, 100×, and 200× coverage: **(a)** HIV-1 89.6 strain, **(b)** Measles virus, **(c)** SARS-CoV-2.

- SARS-CoV-2 isolate INEI121916 is available int the NCBI database (GenBank: OZ072292.1)

Norovirus:

- Nanopore sequencing reads are available in the NCBI database (Run: SRX10330013)
- Illumina sequencing reads are available in the NCBI database (Run: RR13951201, Run: SRR13951221 and Run: SRR1395119)
- The complete genome of Norovirus GII is available in the NCBI database (GenBank: GenBank: MW661264.1, MW661284.1, GenBank: MW661248.1 and GenBank: MW661250.1)

Ebola virus:

- Ebola virus (EBOV-May) Mayinga strain complete genome is available in the NCBI database (GenBank: AF086833.2)
- Ebola virus/M.fascicularis-wt/GAB/2001/untreated-CCL053D9, complete genome is available in the NCBI database (GenBank: KY786027.1)

Measles virus:

- Measles complete genome is available in the NCBI database (NCBI Reference Sequence: NC_001498.1)
- Measles virus genotype A transgenic strain vac2(GFP)H genome is available in the NCBI database (GenBank: MH144178.1)

Declarations

List of abbreviations

- EBOV: Ebola virus
- GP: glycoprotein
- HGAP: Hierarchical Genome-assembly Process
- HIV: Human Immunodeficiency Virus
- NBCI: National Center for Biotechnology Information (NCBI).
- NP: Nucleoprotein
- NGS: Next-Generation Sequencing
- SARS-CoV-2: Severe Acute Respiratory Syndrome Coronavirus 2
- SMRT: Single Molecule Real-Time
- TGS: Third-Generation Sequencing

Consent for publication

Not applicable.

Competing Interests

No competing interests.

Funding

This work is fully supported by funds from the National Science Foundation (NSF: 61972329) and GRF grants for Hong Kong Special Administrative Region, P. R. China (CityU 11210119, and CityU 11206120).

Author's Contributions

L.W. conceived the original idea and supervised the entire work. Z.S. continuously expanded the work, conceptualized the iterative process, developed the entire PVGA algorithm, and was the primary contributor to the manuscript. D.C. provided suggestions during the experimental phase and assisted in locating real data for testing. Y.S. contributed valuable insights for the experimental design. All authors participated in reviewing and improving the manuscript.

Acknowledgements

This work is fully supported by funds from the National Science Foundation (NSF: 61972329) and GRF grants for Hong Kong Special Administrative Region, P. R. China (CityU 11210119, and CityU 11206120).

Authors' information

- **Zhi Song** is a Ph.D. candidate in the Department of Computer Science at City University of Hong Kong. His research interests include algorithms, machine learning, computational biology, and bioinformatics.
- **Dehan Cai** is a Ph.D. candidate in the Department of Electrical Engineering at City University of Hong Kong. His main research interests include sequence analysis in bioinformatics and computational biology, focusing on developing tools for microbial analysis using machine learning and statistical methods.
- **Yanni Sun** is a professor in the Department of Electrical Engineering at City University of Hong Kong. Before relocating to Hong Kong, she was an Associate Professor in the Department of Computer Science and Engineering at Michigan State University, USA. She received both her BS and MS degrees from Xi'an Jiao Tong University (China) in Computer Science and her PhD in Computer Science and Engineering from Washington University in Saint Louis, USA. Her research interests include bioinformatics and computational biology, particularly sequence analysis, machine learning, data mining for next-generation sequencing data, metagenomics, protein domain annotation, and noncoding RNA annotation. She received the NSF CAREER Award in 2010.
- **Lusheng Wang** obtained his Ph.D. in Computer Engineering from McMaster University in 1995. He is currently a professor in the Department of Computer Science at City University of Hong Kong. His research interests include algorithms, computational biology, and bioinformatics.

References

1. Hofacker IL, Stadler PF, Stocsits RR. Conserved RNA secondary structures in viral genomes: a survey. Bioinformatics 2004;20(10):1495–1499.
2. Harvey WT, Carabelli AM, Jackson B, Gupta RK, Thomson EC, Harrison EM, et al. SARS-CoV-2 variants, spike mutations and immune escape. Nature Reviews Microbiology 2021;19(7):409–424.
3. Jain M, Koren S, Miga KH, Quick J, Rand AC, Sasani TA, et al. Nanopore sequencing and assembly of a human genome with ultra-long reads. Nature biotechnology 2018;36(4):338–345.
4. Eid J, Fehr A, Gray J, Luong K, Lyle J, Otto G, et al. Real-time DNA sequencing from single polymerase molecules. Science 2009;323(5910):133–138.
5. Wenger AM, Peluso P, Rowell WJ, Chang PC, Hall RJ, Concepcion GT, et al. Accurate circular consensus long-read sequencing improves variant detection and assembly of a human genome. Nature biotechnology 2019;37(10):1155–1162.
6. Zerbino DR, Birney E. Velvet: algorithms for de novo short read assembly using de Bruijn graphs. Genome research 2008;18(5):821–829.
7. Simpson JT, Wong K, Jackman SD, Schein JE, Jones SJ, Birol I. ABySS: a parallel assembler for short read sequence data. Genome research 2009;19(6):1117–1123.

8. Bankevich A, Nurk S, Antipov D, Gurevich AA, Dvorkin M, Kulikov AS, et al. SPAdes: a new genome assembly algorithm and its applications to single-cell sequencing. Journal of computational biology 2012;19(5):455–477.
9. Kolmogorov M, Yuan J, Lin Y, Pevzner PA. Assembly of long, error-prone reads using repeat graphs. Nature biotechnology 2019;37(5):540–546.
10. Koren S, Walenz BP, Berlin K, Miller JR, Bergman NH, Phillippy AM. Canu: scalable and accurate long-read assembly via adaptive k-mer weighting and repeat separation. Genome research 2017;27(5):722–736.
11. Liu J, Yu T, Mu Z, Li G. TransLiG: a de novo transcriptome assembler that uses line graph iteration. Genome biology 2019;20:1–9.
12. Mu JC, Jiang H, Kiani A, Mohiyuddin M, Bani Asadi N, Wong WH. Fast and accurate read alignment for resequencing. Bioinformatics 2012;28(18):2366–2373.
13. Li H, Ruan J, Durbin R. Maq: Mapping and assembly with qualities. Version 06 2008;3:508.
14. Grubaugh ND, Gangavarapu K, Quick J, Matteson NL, De Jesus JG, Main BJ, et al. An amplicon-based sequencing framework for accurately measuring intrahost virus diversity using PrimalSeq and iVar. Genome biology 2019;20:1–19.
15. Yu R, Cai D, Sun Y. AccuVIR: an ACCUrate VIRal genome assembly tool for third-generation sequencing data. Bioinformatics 2023;39(1):btac827.
16. Li H. A statistical framework for SNP calling, mutation discovery, association mapping and population genetical parameter estimation from sequencing data. Bioinformatics 2011;27(21):2987–2993.
17. Hunt M, Gall A, Ong SH, Brener J, Ferns B, Goulder P, et al. IVA: accurate de novo assembly of RNA virus genomes. Bioinformatics 2015;31(14):2374–2376.
18. Walker BJ, Abeel T, Shea T, Priest M, Abouelliel A, Sakthikumar S, et al. Pilon: an integrated tool for comprehensive microbial variant detection and genome assembly improvement. PloS one 2014;9(11):e112963.
19. Hu J, Fan J, Sun Z, Liu S. NextPolish: a fast and efficient genome polishing tool for long-read assembly. Bioinformatics 2020;36(7):2253–2255.
20. Chin CS, Alexander DH, Marks P, Klammer AA, Drake J, Heiner C, et al. Nonhybrid, finished microbial genome assemblies from long-read SMRT sequencing data. Nature methods 2013;10(6):563–569.
21. Wick RR. Badread: simulation of error-prone long reads. Journal of Open Source Software 2019;4(36):1316.
22. Giallonardo FD, Töpfer A, Rey M, Prabhakaran S, Duport Y, Leemann C, et al. Full-length haplotype reconstruction to infer the structure of heterogeneous virus populations. Nucleic acids research 2014;42(14):e115–e115.
23. Li H. Minimap2: pairwise alignment for nucleotide sequences. Bioinformatics 2018;34(18):3094–3100.
24. Danecek P, Bonfield JK, Liddle J, Marshall J, Ohan V, Pollard MO, et al. Twelve years of SAMtools and BCFtools. Gigascience 2021;10(2):giab008.
25. Flint A, Reaume S, Harlow J, Hoover E, Weedmark K, Nasheri N. Genomic analysis of human noroviruses using combined Illumina–Nanopore data. Virus Evolution 2021;7(2):veab079.
26. Bukreyev A, Volchkov V, Blinov V, Netesov S. The VP35 and VP40 proteins of filoviruses: homology between Marburg and Ebola viruses. FEBS letters 1993;322(1):41–46.
27. Volchkov VE, Volchkova VA, Slenczka W, Klenk HD, Feldmann H. Release of viral glycoproteins during Ebola virus infection. Virology 1998;245(1):110–119.
28. Guedj J, Piorkowski G, Jacquot F, Madelain V, Nguyen THT, Rodallec A, et al. Antiviral efficacy of favipiravir against Ebola virus: A translational study in cynomolgus macaques. PLoS medicine 2018;15(3):e1002535.
29. Del Valle JR, Devaux P, Hodge G, Wegner NJ, McChesney MB, Cattaneo R. A vectored measles virus induces hepatitis B surface antigen antibodies while protecting macaques against measles virus challenge. Journal of virology 2007;81(19):10597–10605.
30. Pfaller CK, Mastorakos GM, Matchett WE, Ma X, Samuel CE, Cattaneo R. Measles virus defective interfering RNAs are generated frequently and early in the absence of C protein and can be destabilized by adenosine deaminase acting on RNA-1-like hypermutations. Journal of virology 2015;89(15):7735–7747.
31. Lee JY, Kong M, Oh J, Lim J, Chung SH, Kim JM, et al. Comparative evaluation of Nanopore polishing tools for microbial genome assembly and polishing strategies for downstream analysis. Scientific Reports 2021;11(1):20740.
32. Ip CL, Loose M, Tyson JR, de Cesare M, Brown BL, Jain M, et al. MinION Analysis and Reference Consortium: Phase 1 data release and analysis. F1000Research 2015;4.
33. Delahaye C, Nicolas J. Sequencing DNA with nanopores: Troubles and biases. PloS one 2021;16(10):e0257521.
34. Watson M, Warr A. Errors in long-read assemblies can critically affect protein prediction. Nature biotechnology 2019;37(2):124–126.

supplementary

Click here to access/download
Supplementary Material
supplementaryNew.pdf

Personal Cover Click here to access/download;Personal Cover;responseLetter.pdf

Lusheng Wang (the corresponding author)
Dept. of Computer Science
City University of Hong Kong
Hong Kong (SAR)
cswangl@cityu.edu.hk

Dear Editor and Reviewers,
We would like to thank you for your careful reading of our paper and the valuable comments. We have addressed the comments of all reviewers, with their comments in black and our responses in blue. The revised figures and descriptions were added to the main manuscript and the supplementary file.

Before we provide the detailed responses, we would like to summarize the **major improvements**.

1. The tool is registered in bio.tools (RRID:SCR_026410) and its computational workflows are permanently archived at WorkflowHub (DOI:10.48546/workflowhub.workflow.1305.1). To ensure broad accessibility, PVGA supports both PyPI installation (pip install pvga) and Bioconda deployment (conda install -c bioconda pvga).
2. We have added our detail description of our parameters in PVGA at our Github repository https://github.com/SoSongzhi/PVGA.
3. We have expanded our experiments to test how backbone choice affects assembly accuracy. We use real HIV strains (89.6, HXB2, NL43, YU2) as backbones for assembling the JRCSF genome, we also created computer-modified JRCSF backbones with increasing differences (15%, 20%, 25%, 30%) through controlled random mutations. Results in Tables 10-11 show how these backbone variations impact assembly quality. See subsection “Convergence performance across different backbones”

We hope that the following point-to-point responses will be clear enough to convey our ideas and address the comments of the reviewers.

Response to Reviewer 1’s comments

Major review points:
(1) Please add one proof of concept analysis for PVGA’s algorithmic convergence starting from different backbone genomes. A concern of mine is that the algorithm might converges towards certain alleles or strains when different backbone genomes are used. This can be tested with just three – or more – different strains of the same species. E.g. from three distinct strains {sample= X, backbones= {Y, Z}} you take reads from X and assemble them with Y and Z as backbones. Then check whether both approaches still assemble X accurately. Ideally and to be convincing, the genomes Y and Z in the set of backbones should be reasonably distant in sequence similarity and no closely related recombinants.

This is one of the major improvements, which we have responded as item 3 in major improvements. Basically, we add subsection “Convergence performance across different backbones” to address the issue. See Page. 8.

(2) I tried using the software and run it on some very simple SARS-CoV-2 sequence data. I noticed several issues that made it difficult to install and impractical to use the software yet. Please address the following issues and consider suggestions regarding the software:

a. [Issue]
The installation instructions do not work without further ado.
conda create pvga --name pvga python==3.10
This line has pvga as a positional argument. This does not work in conda. Further, if the user makes it until
pip install -r requirements.txt
then the pip dependency “python==3.10” collides and breaks because of the python instance previously installed via conda.

We sincerely appreciate this critical technical feedback. Users now can download PVGA with follow commands:

```
conda create --name pvga python=3.10
conda activate pvga
conda install -c bioconda pvga
```

b. [Issue]
Once I got through the installation section I couldn’t get PVGA to run. I provided a backbone genome (-b) and a set of ONT reads (-r). Now, the software informs me that a number if iterations (-n) is required as well as a ground truth sequence (-gt). The paper in contrast says that the iterative process simply runs until no further improvement is made in an iteration. Hence, I expect this parameter not to be required. If even specified, I’d understand this parameter as an upper bound. Also, in practical applications there is no known ground truth. Why is this parameter required?

We appreciate this critical technical feedback. The -n parameter was implemented to evaluate the influence of iterations on assembly results. For instance, setting -n 1 allows us to obtain results without iteration, as demonstrated in the benchmarking figure in the paper. Additionally, the -gt parameter introduced in the previous version was to input ground truth for alignment tool QUAST to evaluate the performance of the assembly. In the user-facing version, based on your recommendation, we have removed the -gt parameter and made the iteration number -n optional, with a default value as the upper bound. Users can still specify the iteration number as needed.

c. [Issue]
Hypothetically, if I could make PVGA run I wouldn’t know how to provide paired-end short-read sequencing data. Since there is only one read parameter (-r) do I need to provide short-read paired-end data as interleaved fastq file? Please improve documentation.

Thank you for this valuable advice. While PVGA takes single reads as input, it can also process paired-end reads. Users with paired-end data can merge their FASTQ files using BBMap while

preserving paired-end information. The merged FASTQ file, where overlapping read pairs are combined into longer single reads, can then be used as input for PVGA assembly. By combining these longer single reads with the unmerged reads from both FASTQ files, PVGA can effectively handle paired-end data. This approach maintains compatibility with PVGA's single-read input design while ensuring efficient processing of paired-end datasets. We have included this explanation along with the BBMap command lines in our README.

d. [Improvement suggestion]
For such a simple installation instruction you utilized two package management systems, conda and pip. Everything necessary to run PVGA can be installed via one concise conda environment. I suggest such an approach would be greatly appreciated by the users.
e. [Improvement suggestion]
Since every dependency can be installed via conda it would be of great help and simplicity for the user if PVGA itself could be installed via (bio)conda -https://bioconda.github.io/. This is a user-friendly way to distribute your bioinformatics software.

Thanks for the valuable suggestions regarding package management. PVGA is now officially available on both Bioconda (conda install -c bioconda pvga) and PyPI (pip install pvga), providing users with flexible installation options to suit their preferred workflows.

f.[Improvement suggestion]
It is good software development practice that the software complies with semantic versioning - https://semver.org/. Please display the version on the help page (--help). The shield on your Github README can then automatically use this implemented version.

Thanks for this valuable improvement suggestion. We have updated PVGA to comply with semantic versioning as per semver.org. The version number is now prominently displayed in the help page (via --help) and integrated into the GitHub README using dynamic shields from shields.io. We will maintain strict adherence to semantic versioning in all future releases.

g. [Improvement suggestion]
Short parameter of two letters is rather confusing and unnecessary for PVGA's short list of parameters. You might like to have a look at Heng Li's CLI best practice - https://lh3.github.io/2021/07/04/designing-command-line-interfaces.

Thanks for this valuable improvement suggestion. we have revised PVGA's command-line interface to use single-letter short parameters exclusively (e.g., -r for reads, -b for backbone, -o for output directory), accompanied by clear and detailed descriptions in the help page (--help).

(1) Please improve some minor language errors:
a. [Intro] (Article) "The virus genomes have relatively small size."

We totally agree with your suggestion. We have removed this sentense.

b. [Intro] (Article) "For example, several mutations in the spike protein gene of coronavirus can […]"

We totally agree with your suggestion. "*For example, several mutations in the spike protein gene of coronavirus can [...]*" has been modified to "*For example, genetic mutations in the coronavirus genome that alter the spike protein can affect its ability to interact with host cells, thereby affecting transmissibility and disease severity*." See the bottom of the left column on Page 1.

c. [Intro] (Wording) "[…] it is well-known that NGS struggles with assembling repetitive regions in the genome" → NGS itself is not performing assembly; better something like "difficult to assemble repetitive regions using NGS data"

We totally agree with your suggestion. *"[...] it is well-known that NGS struggles with assembling repetitive regions in the genome*" has been modified to "*However, due to the short read lengths and the presence of repetitive regions, it is often challenging to assemble genomes accurately using NGS data*." See the second paragraph of the Section "Introduction".

d. [Datasets] (Wording) "[…] as a target to generate the simulation reads." → "as a target and to generate simulated reads" or "as a target and to generate synthetic reads"

Thanks for your suggestion. "[…] as a target to generate the simulation reads." has been modified to "as a target and to generate simulation reads.". See the subsubsection "SAR-CoV-2" in the subsection "Datasets" See the bottom of the right column on Page 1.

e. [Datasets] (Typo) "We utilize two Measles virus strain." → plural "strains"

Thanks for your suggestion. "We utilize two Measles virus strain." has been modified to "We utilize two Measles virus strains." See the middle of the right column on Page 4.

f. [Evaluation on simulated data] (Wording) "[…] refinement process significantly enhances assembly accuracy." → I am against "significant" here since, in the scientific context, this usually means it's statistically tested which is rather difficult for these small numbers.

Thanks for your suggestion. The word "significant" has been removed.

g. I stop explicitly listing here but there are more errors, e.g. with the plural and relative clauses. Please spellcheck in general.

(2) [Intro]
When you introduce reference-based assembly you cite tools like BWA, Bowtie and GATK. To the best of my knowledge, these tools are primarily read mapper and variant caller. Both read mapping and variant calling serve as a prior for reference-guided assembly but it is technically not the assembly process they are performing. Assembly, both de novo and reference-guided, should yield a consensus sequence or contigs as an output. Software like bcftools (https://samtools.github.io/bcftools/bcftools.html), iVar (https://github.com/andersen-lab/ivar) or AccuVir perform reference-guided assembly. Please improve the wording here or correct accordingly.

Thanks for pointing out the error. We have removed the description of these tools from the section on reference-based assembly. Instead, we now focus on tools specifically designed for reference-guided assembly. The comparison before and after modification is as follows:

Origin: “Famous reference-guided assembly methods include BWA, Bowtie2, GATK, Novoalign, and Maq.”

Revised: “Famous reference-guided assembly methods include Novoalign, Maq, iVar, Accuvir and bcftools.” See the middle of the left column of Page 2.

(3) [Intro]
Please comment on the shortcoming of previous approaches that led to the motivation to develop another reference-guided assembler.

Thank you for your valuable suggestion. We have addressed the shortcoming of previous approaches in the introduction by stating: *“However, despite these advancements, current genome assembly tools still fail to achieve the requisite base-level accuracy for viral genome assembly.”* See the middle of the left column of Page 2.

(4) [Methods – Alignment graph construction]
The process is mostly well defined and nicely visualized with Figure 1. However, I have several detail questions here that need to be addressed in the manuscript:

a. What is u’? I assume from the context that it is the new node after a merging f: v_i, $s_i \rightarrow u$’ where v_i and s_i had the same label and parent.

Yes, you are correct. u’ is indeed the new node created after merging nodes vi and si, which share the same label and parent. To clarify this process, we have added an illustration showing how u’ is derived, along with its predecessor node v as follows:

Origin: “The weight of the remaining edge (v, u') is updated to be the number of supporting reads.”
Revised: “When merging nodes into a new node u' with its predecessor v, the weight of the resulting edge (v, u') is updated to reflect the total number of supporting reads.” See the subsection “Alignment graph construction”

b. A very crucial detail is hidden behind the wording “If r_i is matched with an identical letter s_j in the alignment, then r_i corresponds to the node in v_j in G_b.” This matching is a complex problem itself. From your Figure 1c I can only hypothesize that some sort of sequence-to-graph alignment is involved. Please describe the matching in more detail.

Thanks for your advise. Our alignment graph methodology is primarily based on the paper "Nonhybrid, finished microbial genome assemblies from long-read SMRT sequencing data", which provides a detailed methodology for constructing the alignment graph. Specifically, we first align the reads to the backbone genome using BLASR software, then construct the graph. We focus more explicitly on the algorithmic steps for building the alignment graph following read-to-backbone genome alignment, and we have supplemented these details in the manuscript as follows：

“In the alignment, if ri is aligned to an identical letter sj in the backbone sequence, then ri corresponds to the existing node vj in the graph Gb. The weight of the edge (vj–1, vj) will be incremented by one, where vj–1 is the predecessor node of vj. If ri is aligned with a space or a letter sj not identical to ri, we will create a new node ui labeled with ri and add an edge (ui–1, ui) with weight 1, where ui–1 is the node corresponding to the previous letter ri–1. See Fig. 1(b).” See the subsection “Alignment graph construction”

c. What happens in the backtracking if there is a numeric tie between the weights (alleles)? Can alleles accidentally swap in and out per iteration?

Thanks for your question. Based on our empirical observations, we have not encountered instances of numeric ties between allele weights. From an algorithmic perspective, if such a tie were to occur, our method would resolve it by selecting one of the alleles as the final result This selection process ensures deterministic output while maintaining assembly consistency.

(5) [Methods – Evaluation on simulated data]
Please state whether the competing methods (Canu, Flye, AccuVir, PBDAG-Con, Medaka) are de novo or reference-based methods.
Also, PEPPER-Margin is a quite popular pipeline for phased haplotype inference. To avoid adding more analyses here you can argue that you improve upon PEPPER-Margin via implication through AccuVir.

Thank you for your suggestion. Canu and Flye are De Novo methods, while Accuvir, PBDAG-Con, and Medaka are reference-based methods. We have added this description in the revised manuscript. In Accuvir, PEPPER and Margin were tested extensively on simulated data experiments, and the results showed that Accuvir and Medaka outperformed PEPPER-Margin in most cases. Since we used a similar approach to generate simulated data and given the superior performance of Accuvir and Medaka, we benchmarked PVGA only against Medaka and Accuvir, excluding the others from our analysis.

(6) [Methods – Evaluation on simulated data]
Have you left out an evaluation on simulated short-read data? If so, why?

Yes, we evaluated PVGA on real-world Illumina short-read datasets (Table 8), focusing on norovirus sequencing data (SRR13951201, SRR13951221, SRR1395119 with read lengths 301/201). This aligns with our methodology stated in the text: “To assess the effectiveness of the PVGA method with extensive short-read datasets, we employ actual Illumina sequencing data of the norovirus. […]” See the bottom of the right column of Page 2.

(7) [Methods – Evaluation on simulated data]
“Edit distance indicates a minimum number of operations required to transform the assembled genome into the reference sequence.” → “Edit distance defines a minimum number of substitution and indel operations required to transform the assembled genome into the reference sequence.”

Thank you for this insightful suggestion. We have revised the definition of edit distance in the Results section as recommended. See the middel part of the right column on the Page 4.

(8) [Methods – Evaluation on simulated data]
“To simulate real sequencing data, we used the Badread […]” → “To simulate sequencing data, we used the Badread […]”

Thank you for this insightful suggestion. We have deleted the word “real”.

(9) [Methods – Evaluation on simulated data]
You applied different error models to Badread to simulate reads of different sequencing technologies but used the same identity settings in both cases, i.e. for PacBio and ONT. Have you investigated whether this leads to a noticeable difference in sequence similarity between the simulated datasets? I.e. if the identity settings are the same for PacBio and ONT, what difference can you observe across the simulated datasets? Please add a brief comparison/number. One quick and easy solution would be to analyze the alignments of the simulated reads with the perl one-liners by Heng Li (https://lh3.github.io/2018/11/25/on-the-definition-of-sequence-identity).

Thanks for your question. We analyzed the simulated PacBio and Nanopore datasets using Heng Li's alignment-based identity metrics. While the overall read identities were comparable between the two datasets (both approximating the preset identity thresholds in Badread), we observed distinct error profile distributions as documented in Badread's error models (https://github.com/rrwick/Badread/wiki/Error-models). Specifically, PacBio-like simulated reads produced fewer completely correct reads compared to Nanopore-like simulated reads. (see " PacBio2016 model" section in documentation). This indicates that error-containing reads are more likely to occur, leading to increased alignment errors or misalignments during assembly. We have added relavite illustration in the supplemantary file.

(10) [Table 1,2,3]
“length: 9713bp” → “genome length: 9713bp” Maybe rephrase the description. The first half of the sentence suggests that HIV-1 would be a simulation.

Thank you for this insightful suggestion. We have revised the description as recommended.

(11) [Evaluation on poor sequencing conditions]
I believe this section provides an important and relevant analysis of a more error-prone sequencing experiment. However, please consider adding the following adjustments:
a. Please state clearly how you increased the error-rate from an average of 5%(?) to now 10%. The former is a little bit hidden in the “identity settings” of the simulation setup.

Thank you for the constructive feedback. We have explicitly clarified the error rate modification protocol as follows:

“To simulate poor sequencing conditions, we configured the following parameters for evaluating assembler performance under suboptimal data quality: We apply a truncated normal distribution of basecall identity (range: 85–95%, mean: 90%, SD: 5%), resulting in an average read error rate of 10\% and an upper accuracy bound of 95%. And we assign uniform sequencing depth (30×) and average read length (4 kb) across all viral genomes (HIV, Measles, Ebola) to standardize suboptimal quality conditions. The results are shown as Table5.” See the subsection “Evaluation on poor sequencing conditions”.

b. You use [32] to reason the high error-rate. While historically true, this paper and its data are a decade old and, in my humble experience, Oxford Nanopore Technologies has done a massive leap in sequencing quality since. Consider adding that the poor sequencing conditions setup reflects particularly poor sequencing runs, usage of outdated flowcells, or assembly of elder sequencing data. All those are common and perfectly realistic scenarios.

We sincerely thank the reviewer for their valuable insight. Based on your suggestion, we have revised the manuscript to add the rationale for evaluating the assembly of high error-rate reads as follows:
“With the advancement of sequencing technologies, there has been a significant leap in both the capabilities and quality of sequencing. For instance, PacBio sequencing technology can offer HiFi reads that provide an accuracy of 99.9%. However, some laboratories continue to rely on older sequencing equipment or encounter suboptimal results due to experimental limitations. In such cases, there is a need for an assembler capable of effectively handling data with relatively higher error rates.” See the subsection “Evaluation on poor sequencing conditions”.

(12) [Evaluation on real data]
The data situation is not entirely clear to me. As I understand, the mock community has sequencing data from five distinct HIV genomes. Then, you test the genome reconstruction with three different subtypes as target. Please address the following questions to better understand the experiment:

a. Why not five targets, one for each genome?

We selected three HIV strains (89.6, JR-CSF, and YU-2) for the assembly analysis because they met our quality criteria. All reads were first aligned to the HXB2 reference genome to standardize the analysis. These three strains have sufficient sequencing depth making them suitable for assembly. In contrast, the NL43 strain are low abundance reads because its reads were of very low quality and had insufficient coverage.

~~b. I can only assume from the paragraph that 89.6, JR-CSF and YU-2 are members of the five genomes from the mock community. Is that right?~~
~~c. Is the backbone genome HXB2 one member of the five genomes in the mock community?~~

These two questions were deleted by the reviewers.

d. Please add a reference of the mock community data here, or a hint to the Data availability section.

Thanks for your advise, We have added the citation for the mock HIV-1 community dataset in the subsection “Evaluation on real data”, which was sourced from the paper “Giallonardo,F.D. et al. (2014) Full-length haplotype reconstruction to infer the structure of heterogeneous virus populations. Nucleic Acids Res., 42, e115.” See the bottom of the left column on the page 7.

(13) [Evaluation on real data]
“As shown in Table 5, as for the 89.6 Strain, Canu produces a contig of only 4,593 base pairs” → I think that is supposed to be Table 6

Thank you for catching this inconsistency. We have corrected this reference in the revised manuscript.

(14) [Evaluation on real data]Do you have a reason/hypothesis what is causing the remaining mismatches and indels that remain after iterative PVGA? (e.g. in Table 6)

We attribute the residual mismatches and indels after iterative PVGA primarily to inherent limitations of real-world short-read data quality. This hypothesis aligns with the suboptimal performance of other assemblers (e.g., Accuvir, Medaka and PBDAG-Con) in Table 6), which exhibit higher mismatches compared with their performance under simulations reads. Additionally, the lack of validated ground truth for most real datasets complicates definitive error correction. In our manuscript, we illustrated “Due to the lower quality of real reads, assemblers dis-play higher mismatches” in the top of the left column on Page 7.

(15) [Evaluation on real data]
“The results are in Table 7. Our method PVGA exhibits exceptional accuracy […]” → I think that is supposed to be Table 8

Thank you for catching this inconsistency. We have corrected this reference in the revised manuscript.

(16) [Table 8]
Until here I assumed that if the number of mismatches and indels are both zero, an edit distance >0 can only be explained with missing sequence at the flanks of the genome. However, that’s not the case according to the first and third row of Table 8. How is the edit distance >0 while the genome fraction is 100 and the number of mismatches and indels is zero? Please comment on what’s the difference here between reconstructed strain and ground truth. I suppose that is what is meant with “[…] misalignment at the two ends of the genomes.” Is the reconstructed strain slightly longer than the ground truth?

It was a good question. The Genome fraction metric, as defined by QUAST, measures the proportion of the reference genome covered by aligned contigs, calculated by dividing the total aligned reference bases by the genome size.

Shown as the QUAST instructions: “Genome fraction (%): The total number of aligned bases in the reference, divided by the genome size. A base in the reference genome is counted as aligned

if at least one contig has at least one alignment to this base. Contigs from repeat regions may map to multiple places, and thus may be counted multiple times in this quantity."

This metric prioritizes coverage completeness over sequence accuracy. In Table 8, PVGA's contigs are slightly longer than the ground truth due to non-reference homologous sequences at the termini. While these terminal extensions do not affect the Genome fraction as the entire ground truth is still covered by the aligned regions. they should be counted in edit distance calculations. We have added the ground truth genome lengths in the Table8's notes.

(17) [Evaluation of computing resource usage]
"Notably, Accuvir[17] exhibited a runtime exceeding 30 minutes and was therefore excluded from subsequent performance figures." → I couldn't find running time analyses in the AccuVir publication but they ran analyses on 200x PacBio data. Can you double-check that your code/command was running successfully in general? The running time difference between 1-2 minutes of other tools and AccuVir requiring >30min is surprising.

Thank you for your careful review and valuable suggestion. We have reviewed our code for running Accuvir and confirmed that it takes longer on some datasets due to the numerous search operations. Accuvir employs a local search strategy (beam search) to generate multiple candidate paths and selects the highest-scoring one as the output. To ensure that the candidate paths include a high-quality option, Accuvir uses strategies involving multiple beam search operations, which increases the runtime. While we attempted to adjust parameters to reduce the search space, doing so resulted in faster execution but produced lower-quality output paths compared to the default settings. Therefore, we chose not to reduce the search space for Accuvir, accepting the longer running time.

(18) [Evaluation of computing resource usage]
"As shown in Figure 4, assemblers relying on alignment graph construction tend to require more memory" → Again, I think this is supposed to be Figure 5

Thank you for catching this inconsistency. We have corrected this reference in the revised manuscript.

Response to Reviewer 2's comments

The authors describe a new reference-guided assembly tool designed for viral genomes. The authors validate their assembly approach and compare with similar tools. The paper is generally well written, and the tool would be valuable to the community.

Thank you for your summary. We really appreciate your time in reviewing our manuscript. We have revised the manuscript accordingly. Our point-by-point responses are detailed below.

The tool is presented as an alternative to de novo assemblers. However, I am interested to know if PVGA was able to correct errors that were present in assemblies that were generated de novo e.g. from Flye and Canu. It seems like PVGA would serve equally well as either a reference-guided assembly tool or as an assembly polishing tool.

Thank you for your question. In the originally submitted manuscript, under the section "Benchmarking the capability of polishing" , We have already evaluated PVGA's polishing ability to correct errors introduced by De Novo assemblers like Canu. We first simulated the SARS-CoV-2 Illumina reads with lengths of 200-300 base pairs and coverages of 25x for polishing the Canu's assembly result. Also, we have expanded in the revised manuscript (Section "Convergence performance across different backbones") by assembling the HIV-1 JRCSF strain using **Flye-assembled** results as the backbone. When reapplying PVGA with the same reads used in Flye, PVGA perfectly eliminate the edit distance of 20 introduced by Flye, reconstructing the ground-truth genome.

My main concern is a lack of information on the minimum similarity requirements of the reference genome. The quality of the final assembly is entirely dependent on the quality of the original assembly. Incomplete coverage or low similarity will impact the final assembly and it would be useful for users to know at what point they should use an alternative approach (such as my suggestion above: de novo assembly followed by PVGA for polishing).

Thanks for your suggestion. We have conducted additional experiments to evaluate the impact of backbone selection on PVGA's algorithmic convergence. Specifically, we tested four distinct HIV-1 strains (89.6, HXB2, NL43, and YU2) as backbones for assembling reads derived from the JRCSF strain in both real and simulated data. We first compared the sequence similarity between these backbone genomes and the ground truth JRCSF genome. To further assess how backbone divergence affects assembly accuracy, we introduced manual random errors (15%, 20%, 25%, and 30%) into the JRCSF genome and used these modified sequences as backbones to assemble simulated reads with 5% error rates. The results (Table 11) demonstrate that even with a 30% error rate in the backbone, PVGA maintained robust assembly accuracy. This is attributed to the backbone contributing only a weight of 1 for base connectivity decisions, while read-derived evidence (proportional to coverage) dominated the alignment graph construction. However, higher divergence between the backbone and the ground truth led to shorter assembly lengths, specifically manifesting as fragment loss at the genome ends, while the central regions remained error-free as shown in Table 11. This occurs because regions at the ends of the backbone with higher divergence showed reduced alignment efficiency: reads spanning these divergent regions failed to align to the backbone and thus could not contribute to the graph construction.

I would strongly recommend adding the tool to bioconda. Can you use the bioconda version of blasr? I believe all the dependencies in requirements should be available on conda. You should also be to publish as a python package.

Thanks for your valuable suggestion, we have already contributed PVGA to bioconda. Now users can install and run the tool seamlessly without manually configuring their environments. The download command is provided below:

```
conda create --name pvga python=3.10
conda activate pvga
conda install -c bioconda pvga
```

Misc comments:
"The virus genomes have relatively small size."
All? Some?

Thanks for your suggestion. We have removed this sentence.

"As the basic structure of the virus genomes is highly conserved …"
Do you have a citation for this? As this is a reference-guided assembly tool, the quality of the final assembly is dependent on having adequate structural and sequence similarity to the reference used.

Thank you for your feedback. We have revised the statement to: "As the basic structure of many virus genomes is highly conserved…" The citation supporting this claim is derived from "Conserved RNA secondary structures in viral genomes: a survey" which illustrates that "The genomes of RNA viruses often carry conserved RNA structures that perform vital functions during the life cycle of the virus. See the begging part of Section "Introduction".

"However, these longer reads tend to have higher error rates."
I don't believe this is nearly as relevant as it would have been 5 years ago. PacBio HiFi reads are very accurate and nanopore is not far behind.

We have revised the statement to "In comparison with NGS， TGS tends to have higher error rates." In addition, we added discussion on this part in this paper to deepen the necessity of using assembler for high error rate reads' assembly in "Evaluation on poor sequencing conditions" section as follows：
"For instance, PacBio sequencing technology can offer HiFi reads that provide an accuracy of 99.9\%. However, some laboratories continue to rely on older sequencing equipment or encounter suboptimal results due to experimental limitations. In such cases, there is a need for an assembler capable of effectively handling data with relatively higher error rates."
See the begging part of the subsection "Evaluation on poor-sequencing conditions"

"Famous reference-guided assembly methods include BWA [11], Bowtie2 [12], GATK [13], Novoalign [14]..."
These are all read alignment programs if I'm not mistaken.

Thanks for your valuable suggestion. We have carefully reviewed your comments and agree that the tools mentioned (BWA, Bowtie, and GATK) are primarily designed for read mapping and variant calling rather than reference-guided assembly. While these tools are often used as part of the preprocessing steps for reference-guided assembly, they do not directly produce consensus sequences or contigs, which are the key outputs of an assembly process. We have removed the description of these tools from the section on reference-based assembly. Instead, we now focus on tools specifically designed for reference-guided assembly. The comparison before and after modification is as follows:

Origin: "Famous reference-guided assembly methods include BWA, Bowtie2, GATK, Novoalign, and Maq."

Revised: “Famous reference-guided assembly methods include Novoalign, Maq, iVar, Accuvir and bcftools.”

"For instance, IVA was developed as a De Novo assembler for RNA viruses, utilizing paired-end datasets to achieve more accurate assemblies [16]. Similarly, Accuvir introduced a reference-based long-read assembler for viruses, primarily employing diverse beam search algorithms on alignment graphs to improve accuracy [17]."
You could expand on how the PVGA approach compares with these other established tools.

Thanks for your comment, we have added the motivation of designing PVGA as follows: “However, despite these advancements, current viral genome assembly tools still fail to achieve the requisite base-level accuracy.” And we had a comprehensive comparison in Results section.

"PVGA starts with a reference genome and utilizes the sequencing reads directly to reduce noise."
Reduce what noise? How?

The term "noise" in this context refers to the sequence divergence between the backbone genome and the ground truth. PVGA mitigates such noise through an iterative graph refinement process as shown in the section “Results” and the section “Method”.

"The results demonstrate that PVGA consistently outperforms the best existing programs"
I would reword 'the best programs' to something like 'popular existing programs'

Thanks for your suggestion. We have revised it.

Figure 1: do the reads get remapped each iteration?

Yes, as described in the subsection "Updating the Reference Genome Iteratively", the alignment graph is dynamically reconstructed during each iteration by realigning all input reads to the updated backbone genome.

Is there a hard cap on the number of iterations?

The upper bound of iteration number is PVGA’s optional parameter. Users can specify an upper limit for the number of iterations via the -n parameter (default: 10).
