# Supplementary material for: PVGA: a precise viral genome assembler using an iterative alignment graph
Source: Gigascience. 2025 Jun 24;14:giaf063. doi: 10.1093/gigascience/giaf063 (PMC12206156; doi:10.1093/gigascience/giaf063)
Supplement: giaf063_GIGA-D-25-00004_Revision_2 [file giaf063_giga-d-25-00004_revision_2.pdf]

GigaScience

PVGA: A Precise Viral Genome Assembler Using Iterative Alignment Graph

--Manuscript Draft--

**Manuscript Number:**	GIGA-D-25-00004R2
**Full Title:**	PVGA: A Precise Viral Genome Assembler Using Iterative Alignment Graph
**Article Type:**	Technical Note
**Funding Information:**	
**Abstract:**	Abstract<br>Background: Viral genome analysis is crucial for understanding viruses evolution and mutation. Investigations into viral evolutionary dynamics and mutation patterns have garnered significant research attention since the outbreak of COVID-19. As the basic structure of many virus genomes is highly conserved [1]. RNA viruses have high mutation rates, and single-nucleotide variations may induce substantial phenotypic alterations in terms of viral function and pathogenicity. Thus, special assembly methods are required for viral genome analysis.<br>Result: PVGA starts with a reference genome and utilizes the sequencing reads directly to reduce noise. The first step in PVGA involves constructing an alignment graph based on a reference genome and the set of input sequencing reads. Then the optimal genomic path is determined through dynamic programming, maximizing the cumulative edge weights that reflect read support density across the alignment graph. The obtained path corresponds to a refined genome. Finally, we repeat the process by using the new reference genomes until no further improvement is possible. We evaluate PVGA's performance across both assembly and polishing tasks using simulated and real datasets including both long reads and short reads. The experiments demonstrate that PVGA always outperforms popular existing programs in terms of the quality of assembly results, while the running time of our method is compatible to others. In particular, simulated Nanopore datasets show that our method can correctly report the true genomes with 0 mismatch and 0 indels.<br>Conclusions: PVGA is a novel viral genome assembler that seamlessly integrates assembly and polishing into a unified workflow. Its design prioritizes high accuracy, enabling the detection of subtle genomic variations that can impact viral function and pathogenicity. By addressing the unique challenges of viral genome assembly, PVGA provides a reliable and precise solution for advancing our understanding of viral evolution and behavior.<br>Key words: Genome assembler; Virus genome; Alignment graph; Maximum total weight path; Iterative method.
**Corresponding Author:**	Lusheng Wang<br>City University of Hong Kong<br>Hong Kong, Kowloon HONG KONG
**Corresponding Author Secondary Information:**	
**Corresponding Author's Institution:**	City University of Hong Kong
**Corresponding Author's Secondary Institution:**	
**First Author:**	Zhi Song
**First Author Secondary Information:**	
**Order of Authors:**	Zhi Song
	Dehan Cai
	Yanni Sun
	LUSHENG WANG
**Order of Authors Secondary Information:**	
**Response to Reviewers:**	The responses are listed in the response letter file.

Powered by Editorial Manager® and ProduXion Manager® from Aries Systems Corporation

Additional Information:	
**Question**	**Response**
Are you submitting this manuscript to a special series or article collection?	No
**Experimental design and statistics**<br><br>Full details of the experimental design and statistical methods used should be given in the Methods section, as detailed in our Minimum Standards Reporting Checklist. Information essential to interpreting the data presented should be made available in the figure legends.<br><br>Have you included all the information requested in your manuscript?	Yes
**Resources**<br><br>A description of all resources used, including antibodies, cell lines, animals and software tools, with enough information to allow them to be uniquely identified, should be included in the Methods section. Authors are strongly encouraged to cite Research Resource Identifiers (RRIDs) for antibodies, model organisms and tools, where possible.<br><br>Have you included the information requested as detailed in our Minimum Standards Reporting Checklist?	Yes
**Availability of data and materials**<br><br>All datasets and code on which the conclusions of the paper rely must be either included in your submission or deposited in publicly available repositories (where available and ethically appropriate), referencing such data using a unique identifier in the references and in the “Availability of Data and Materials” section of your manuscript.	Yes

Have you have met the above requirement as detailed in our Minimum Standards Reporting Checklist?	
GigaScience has policies and guidelines in place for the use of generative AI-writing tools such as ChatGPT. If you have used such writing tools to assist with writing the manuscript this must be declared and cited in the text. Authors should not list AI-writing tools and other AI-assisted technologies as an author or co-author and should acknowledge that they are fully responsible for text generated or refined by AI-writing tools.<p><br><br>A summary of use (particularly in the introduction or among methods) needs to be included at the end of the paper, and the outputs should also be included as a supplementary file hosted in GigaDB or other open repositories. Please read our guidelines for more information. <p><br><br>By submitting to GigaScience, you are aware of the journal's AI-writing tools policy, and if you have declared use of such tools below, you have acknowledged this where appropriate in your manuscript and have made a summary of use and outputs available. <p><br><b>AI-assisted writing tools have been used in the preparation of this manuscript?	No

Click here to access/download;LaTeX - Main Document (TeX file);supplementary_documents.tex

SUPPLEMENTARY DOCUMENT

1 Benchmarking results on PacBio Datasets

Beyond testing on Nanopore data, we also test simulated PacBio data. Tables 1-3 present the results for HIV PacBio datasets, with average read lengths of 2kb, 4kb, and 6kb, respectively, while Tables 4-6 display the benchmarking results for SARS-CoV-2 at average read lengths of 2kb, 4kb, and 6kb, respectively. For both HIV and SARS-CoV-2 results, PVGA consistently demonstrates the lowest values across all conditions in terms of mismatches, indels, indel lengths, and edit distance, indicating that PVGA consistently outperforms other assemblers.

Although we set the same parameter ”identity” in badread to simulate reads at a 5% error rate. the assembly result quality from nearly all assemblers decline when processing simulated PacBio reads compared to those from simulated Nanopore reads. To evaluate the simulated PacBio reads' accuracy, we align reads to the reference genome using minimap2 and calculate their average identity. The tested reads maintained the expected 95% identity (5% error rate). According to Badread's documentation, PacBio-like simulations inherently produce fewer perfect reads (0 mismatches/indels) than Nanopore-like simulations, though this does not alter the global identity parameter. This indicates that error-containing reads are more likely to occur, leading to increased alignment errors or misalignments during assembly.

Nevertheless, PVGA continues to outperform all other assemblers, consistently exhibiting the lowest numbers of indels, mismatches, and edit distances. For instance, as shown in Table 6, at coverage of 50x with an 8kb read length from SARS-CoV-2 datasets, other assemblers report indels, mismatches, and edit distances in double or even triple digits, whereas PVGA maintains single-digit values for each of these metrics.

Table 1: Results on simulated PacBio HIV-1 datasets with a 5% error rate and an average read length of 2kb

Reads depth	Tool	Genome fraction	Contig length	Mismatch	Indels	Indel length	Edit distance
50x	Flye	89.9	8669	0	63	63	1044
	Canu	93.452	12459	0	157	163	7848
	Accuvir	99.959	9678	1	45	49	54
	PBDAG-Con	99.969	9635	0	70	79	82
	Medaka	99.969	9698	9	30	48	60
	PVGA(noiter)	99.969	9703	0	9	9	12
	**PVGA**	**99.969**	**9703**	**0**	**9**	**9**	**12**
100x	Flye	99.938	9642	0	65	65	79
	Canu	99.732	9619	0	66	68	89
	Accuvir	99.959	9672	1	39	43	48
	PBDAG-Con	99.969	9644	0	60	72	75
	Medaka	99.969	9672	8	37	58	69
	PVGA(noiter)	99.969	9710	0	4	4	7
	**PVGA**	**99.969**	**9711**	**0**	**3**	**3**	**6**
200x	Flye	99.99	9635	0	77	77	78
	Canu	93.452	12966	0	131	138	6157
	Accuvir	99.959	9669	0	46	48	52
	PBDAG-Con	99.969	9633	0	69	81	84
	Medaka	99.969	9737	10	17	41	54
	PVGA(noiter)	99.969	9698	4	33	48	55
	**PVGA**	**99.969**	**9707**	**0**	**3**	**3**	**6**

Table 2: Results on simulated PacBio HIV-1 datasets with a 5% error rate and an average read length of 4kb

Reads depth	Tool	Genome fraction	Contig length	Mismatch	Indels	Indel length	Edit distance
50x	Flye	98.312	9490	0	59	59	1066
	Canu	99.979	24809	0	181	194	15224
	Accuvir	99.959	9675	0	43	44	48
	PBDAG-Con	99.969	9648	0	56	62	65
	Medaka	99.969	9687	8	26	48	59
	PVGA(noiter)	99.969	9704	0	10	10	13
	**PVGA**	**99.969**	**9704**	**0**	**10**	**10**	**13**
100x	Flye	100	9653	0	60	60	63
	Canu	100	18670	0	143	143	9093
	Accuvir	99.959	9679	1	34	34	39
	PBDAG-Con	99.969	9650	0	53	60	63
	Medaka	99.969	9705	0	8	8	8
	PVGA(noiter)	99.969	9731	7	23	47	57
	**PVGA**	**99.969**	**9710**	**0**	**4**	**4**	**7**
200x	Flye	99.99	9637	0	75	75	76
	Canu	99.99	9689	0	69	69	116
	Accuvir	99.959	9694	1	27	29	34
	PBDAG-Con	99.969	9637	0	64	73	76
	Medaka	99.969	9684	10	32	58	71
	PVGA(noiter)	99.969	9709	0	1	1	4
	**PVGA**	**99.969**	**9710**	**0**	**1**	**1**	**4**

Table 3: Results on simulated PacBio HIV-1 datasets with a 5% error rate and an average read length of 6kb

Reads depth	Tool	Genome fraction	Contig length	Mismatch	Indels	Indel length	Edit distance
50x	Flye	85.689	8266	0	57	57	1471
	Accuvir	99.959	9680	1	40	43	48
	PBDAG-Con	99.969	9657	0	49	59	62
	Medaka	99.969	9694	14	21	52	69
	PVGA(noiter)	99.969	9709	0	7	7	10
	**PVGA**	**99.969**	**9709**	**0**	**7**	**7**	**10**
100x	Flye	99.969	9650	0	63	63	69
	Canu	99.99	9691	0	61	61	102
	Accuvir	99.959	9675	0	52	60	64
	PBDAG-Con	99.969	9640	0	58	70	73
	Medaka	99.969	9690	19	28	61	83
	PVGA(noiter)	99.969	9710	0	2	2	5
	**PVGA**	**99.969**	**9710**	**0**	**2**	**2**	**5**
200x	Flye	99.568	10304	1	97	98	819
	Accuvir	99.959	9682	1	34	35	40
	PBDAG-Con	99.969	9646	0	60	64	67
	Medaka	99.969	9706	0	7	7	7
	PVGA(noiter)	99.969	9737	10	17	41	54
	**PVGA**	**99.969**	**9710**	**0**	**1**	**1**	**4**

Table 4: Results on simulated PacBio SARS-CoV-2 datasets with 5% error rate and an average read length of 2kb

Reads depth	Tool	Genome fraction	Contig length	Mismatch	Indels	Indel length	Edit distance
25x	Flye	97.46	28759	0	139	140	899
	Canu	98.644	29078	0	164	166	2548
	Accuvir	100	29784	1	97	101	236
	PBDAG-Con	100	29554	0	92	93	94
	Medaka	100	29850	0	22	30	254
	PVGA(noiter)	100	29652	0	20	20	20
	**PVGA**	**100**	**29652**	**0**	**20**	**20**	**20**
50x	Flye	99.997	29531	0	118	119	125
	Canu	99.906	29493	0	124	125	153
	Accuvir	100	29792	1	83	85	309
	PBDAG-Con	100	29593	0	78	79	105
	Medaka	100	29855	0	22	25	249
	PVGA(noiter)	100	29647	0	14	14	15
	**PVGA**	**100**	**29647**	**0**	**14**	**14**	**15**

Table 5: Results on simulated PacBio SARS-CoV-2 datasets with 5% error rate and an average read length of 4kb

Reads depth	Tool	Genome fraction	Contig length	Mismatch	Indels	Indel length	Edit distance
25x	Flye	99.993	29501	0	140	143	145
	Canu	99.811	29420	0	166	170	226
	Accuvir	100	29744	1	125	129	353
	PBDAG-Con	100	29552	0	92	95	96
	Medaka	100	29813	0	64	67	291
	PVGA(noiter)	100	29651	0	20	20	21
	**PVGA**	**100**	**29651**	**0**	**20**	**20**	**21**
50x	Flye	99.997	29556	0	93	94	100
	Canu	91.716	27098	0	90	92	2548
	Accuvir	100	29747	1	108	127	337
	PBDAG-Con	100	29579	0	67	68	69
	Medaka	100	29813	0	24	28	252
	PVGA(noiter)	100	29648	0	5	5	6
	**PVGA**	**100**	**29648**	**0**	**5**	**5**	**6**

Table 6: Results on simulated PacBio SARS-CoV-2 datasets with 5% error rate and an average read length of 8kb("-" indicates that the assembler fails to produce a result.)

Reads depth	Tool	Genome fraction	Contig length	Mismatch	Indels	Indel length	Edit distance
25x	Flye	99.997	29531	0	118	119	125
	Canu	99.906	29493	0	124	125	153
	Accuvir	100	29792	1	83	85	309
	Medaka	100	29609	0	43	45	270
	PBDAG-Con	100	29593	0	78	79	105
	PVGA(noiter)	100	29647	0	14	14	15
	**PVGA**	**100**	**29647**	**0**	**14**	**14**	**15**
50x	Flye	99.983	29572	0	92	93	114
	Canu	-	-	-	-	-	-
	Accuvir	100	29807	0	66	68	291
	Medaka	100	29609	0	23	25	270
	PBDAG-Con	100	29577	2	69	70	73
	PVGA(noiter)	100	29649	0	8	8	9
	**PVGA**	**100**	**29650**	**0**	**7**	**7**	**8**

Click here to access/download;LaTeX - Main Document (TeX file);main.tex

```
This is pdfTeX, Version 3.141592653-2.6-1.40.26 (TeX Live 2024)
(preloaded format=pdflatex 2024.8.2)  15 MAY 2025 09:10
entering extended mode
 restricted \write18 enabled.
 %&-line parsing enabled.
**main.tex
(./main.tex
LaTeX2e <2024-06-01> patch level 2
L3 programming layer <2024-05-27>
(./oup-contemporary.cls
Document Class: oup-contemporary 2023/06/12, v1.2
(c:/texlive/2024/texmf-dist/tex/latex/base/article.cls
Document Class: article 2024/02/08 v1.4n Standard LaTeX document class
(c:/texlive/2024/texmf-dist/tex/latex/base/size10.clo
File: size10.clo 2024/02/08 v1.4n Standard LaTeX file (size option)
)
\c@part=\count194
\c@section=\count195
\c@subsection=\count196
\c@subsubsection=\count197
\c@paragraph=\count198
\c@subparagraph=\count199
\c@figure=\count266
\c@table=\count267
\abovecaptionskip=\skip49
\belowcaptionskip=\skip50
\bibindent=\dimen141
) (c:/texlive/2024/texmf-dist/tex/latex/base/inputenc.sty
Package: inputenc 2024/02/08 v1.3d Input encoding file
\inpenc@prehook=\toks17
\inpenc@posthook=\toks18
) (c:/texlive/2024/texmf-dist/tex/latex/base/fontenc.sty
Package: fontenc 2021/04/29 v2.0v Standard LaTeX package
) (c:/texlive/2024/texmf-dist/tex/generic/iftex/ifpdf.sty
Package: ifpdf 2019/10/25 v3.4 ifpdf legacy package. Use iftex instead.
(c:/texlive/2024/texmf-dist/tex/generic/iftex/iftex.sty
Package: iftex 2022/02/03 v1.0f TeX engine tests
)) (c:/texlive/2024/texmf-dist/tex/latex/microtype/microtype.sty
Package: microtype 2024/03/29 v3.1b Micro-typographical refinements (RS)
(c:/texlive/2024/texmf-dist/tex/latex/graphics/keyval.sty
Package: keyval 2022/05/29 v1.15 key=value parser (DPC)
\KV@toks@=\toks19
) (c:/texlive/2024/texmf-dist/tex/latex/etoolbox/etoolbox.sty
Package: etoolbox 2020/10/05 v2.5k e-TeX tools for LaTeX (JAW)
\etb@tempcnta=\count268
)
\MT@toks=\toks20
\MT@tempbox=\box52
\MT@count=\count269
LaTeX Info: Redefining \noprotrusionifhmode on input line 1061.
LaTeX Info: Redefining \leftprotrusion on input line 1062.
\MT@prot@toks=\toks21
LaTeX Info: Redefining \rightprotrusion on input line 1081.
LaTeX Info: Redefining \textls on input line 1392.
```

```
\MT@outer@kern=\dimen142
LaTeX Info: Redefining \textmicrotypecontext on input line 2013.
\MT@listname@count=\count270
(c:/texlive/2024/texmf-dist/tex/latex/microtype/microtype-pdftex.def
File: microtype-pdftex.def 2024/03/29 v3.1b Definitions specific to
pdftex (RS)

LaTeX Info: Redefining \lsstyle on input line 902.
LaTeX Info: Redefining \lslig on input line 902.
\MT@outer@space=\skip51
)
Package microtype Info: Loading configuration file microtype.cfg.
(c:/texlive/2024/texmf-dist/tex/latex/microtype/microtype.cfg
File: microtype.cfg 2024/03/29 v3.1b microtype main configuration file
(RS)
)) (c:/texlive/2024/texmf-dist/tex/latex/euler/euler.sty
Package: euler 1995/03/05 v2.5
Package: `euler' v2.5 <1995/03/05> (FJ and FMi)
LaTeX Font Info:    Redeclaring symbol font `letters' on input line 35.
LaTeX Font Info:    Encoding `OML' has changed to `U' for symbol font
(Font)              `letters' in the math version `normal' on input line
35.
LaTeX Font Info:    Overwriting symbol font `letters' in version `normal'
(Font)                  OML/cmm/m/it --> U/eur/m/n on input line 35.
LaTeX Font Info:    Encoding `OML' has changed to `U' for symbol font
(Font)              `letters' in the math version `bold' on input line
35.
LaTeX Font Info:    Overwriting symbol font `letters' in version `bold'
(Font)                  OML/cmm/b/it --> U/eur/m/n on input line 35.
LaTeX Font Info:    Overwriting symbol font `letters' in version `bold'
(Font)                  U/eur/m/n --> U/eur/b/n on input line 36.
LaTeX Font Info:    Redeclaring math symbol \Gamma on input line 47.
LaTeX Font Info:    Redeclaring math symbol \Delta on input line 48.
LaTeX Font Info:    Redeclaring math symbol \Theta on input line 49.
LaTeX Font Info:    Redeclaring math symbol \Lambda on input line 50.
LaTeX Font Info:    Redeclaring math symbol \Xi on input line 51.
LaTeX Font Info:    Redeclaring math symbol \Pi on input line 52.
LaTeX Font Info:    Redeclaring math symbol \Sigma on input line 53.
LaTeX Font Info:    Redeclaring math symbol \Upsilon on input line 54.
LaTeX Font Info:    Redeclaring math symbol \Phi on input line 55.
LaTeX Font Info:    Redeclaring math symbol \Psi on input line 56.
LaTeX Font Info:    Redeclaring math symbol \Omega on input line 57.
\symEulerFraktur=\mathgroup4
LaTeX Font Info:    Overwriting symbol font `EulerFraktur' in version
`bold'
(Font)                  U/euf/m/n --> U/euf/b/n on input line 63.
LaTeX Info: Redefining \oldstylenums on input line 85.
\symEulerScript=\mathgroup5
LaTeX Font Info:    Overwriting symbol font `EulerScript' in version
`bold'
(Font)                  U/eus/m/n --> U/eus/b/n on input line 93.
LaTeX Font Info:    Redeclaring math symbol \aleph on input line 97.
LaTeX Font Info:    Redeclaring math symbol \Re on input line 98.
LaTeX Font Info:    Redeclaring math symbol \Im on input line 99.
```

```
LaTeX Font Info:    Redeclaring math delimiter \vert on input line 101.
LaTeX Font Info:    Redeclaring math delimiter \backslash on input line
103.
LaTeX Font Info:    Redeclaring math symbol \neg on input line 106.
LaTeX Font Info:    Redeclaring math symbol \wedge on input line 108.
LaTeX Font Info:    Redeclaring math symbol \vee on input line 110.
LaTeX Font Info:    Redeclaring math symbol \setminus on input line 112.
LaTeX Font Info:    Redeclaring math symbol \sim on input line 113.
LaTeX Font Info:    Redeclaring math symbol \mid on input line 114.
LaTeX Font Info:    Redeclaring math delimiter \arrowvert on input line
116.
LaTeX Font Info:    Redeclaring math symbol \mathsection on input line
117.
\symEulerExtension=\mathgroup6
LaTeX Font Info:    Redeclaring math symbol \coprod on input line 125.
LaTeX Font Info:    Redeclaring math symbol \prod on input line 125.
LaTeX Font Info:    Redeclaring math symbol \sum on input line 125.
LaTeX Font Info:    Redeclaring math symbol \intop on input line 130.
LaTeX Font Info:    Redeclaring math symbol \ointop on input line 131.
LaTeX Font Info:    Redeclaring math symbol \braceld on input line 132.
LaTeX Font Info:    Redeclaring math symbol \bracerd on input line 133.
LaTeX Font Info:    Redeclaring math symbol \bracelu on input line 134.
LaTeX Font Info:    Redeclaring math symbol \braceru on input line 135.
LaTeX Font Info:    Redeclaring math symbol \infty on input line 136.
LaTeX Font Info:    Redeclaring math symbol \nearrow on input line 153.
LaTeX Font Info:    Redeclaring math symbol \searrow on input line 154.
LaTeX Font Info:    Redeclaring math symbol \nwarrow on input line 155.
LaTeX Font Info:    Redeclaring math symbol \swarrow on input line 156.
LaTeX Font Info:    Redeclaring math symbol \Leftrightarrow on input line
157.
LaTeX Font Info:    Redeclaring math symbol \Leftarrow on input line 158.
LaTeX Font Info:    Redeclaring math symbol \Rightarrow on input line
159.
LaTeX Font Info:    Redeclaring math symbol \leftrightarrow on input line
160.
LaTeX Font Info:    Redeclaring math symbol \leftarrow on input line 161.
LaTeX Font Info:    Redeclaring math symbol \rightarrow on input line
163.
LaTeX Font Info:    Redeclaring math delimiter \uparrow on input line
166.
LaTeX Font Info:    Redeclaring math delimiter \downarrow on input line
168.
LaTeX Font Info:    Redeclaring math delimiter \updownarrow on input line
170.
LaTeX Font Info:    Redeclaring math delimiter \Uparrow on input line
172.
LaTeX Font Info:    Redeclaring math delimiter \Downarrow on input line
174.
LaTeX Font Info:    Redeclaring math delimiter \Updownarrow on input line
176.
LaTeX Font Info:    Redeclaring math symbol \leftharpoonup on input line
177.
LaTeX Font Info:    Redeclaring math symbol \leftharpoondown on input
line 178.
```

```
LaTeX Font Info:    Redeclaring math symbol \rightharpoonup on input line
179.
LaTeX Font Info:    Redeclaring math symbol \rightharpoondown on input
line 180
.
LaTeX Font Info:    Redeclaring math delimiter \lbrace on input line 182.
LaTeX Font Info:    Redeclaring math delimiter \rbrace on input line 184.
\symcmmigroup=\mathgroup7
LaTeX Font Info:    Overwriting symbol font `cmmigroup' in version `bold'
(Font)                  OML/cmm/m/it --> OML/cmm/b/it on input line 200.
LaTeX Font Info:    Redeclaring math accent \vec on input line 201.
LaTeX Font Info:    Redeclaring math symbol \triangleleft on input line
202.
LaTeX Font Info:    Redeclaring math symbol \triangleright on input line
203.
LaTeX Font Info:    Redeclaring math symbol \star on input line 204.
LaTeX Font Info:    Redeclaring math symbol \lhook on input line 205.
LaTeX Font Info:    Redeclaring math symbol \rhook on input line 206.
LaTeX Font Info:    Redeclaring math symbol \flat on input line 207.
LaTeX Font Info:    Redeclaring math symbol \natural on input line 208.
LaTeX Font Info:    Redeclaring math symbol \sharp on input line 209.
LaTeX Font Info:    Redeclaring math symbol \smile on input line 210.
LaTeX Font Info:    Redeclaring math symbol \frown on input line 211.
LaTeX Font Info:    Redeclaring math accent \grave on input line 245.
LaTeX Font Info:    Redeclaring math accent \acute on input line 246.
LaTeX Font Info:    Redeclaring math accent \tilde on input line 247.
LaTeX Font Info:    Redeclaring math accent \ddot on input line 248.
LaTeX Font Info:    Redeclaring math accent \check on input line 249.
LaTeX Font Info:    Redeclaring math accent \breve on input line 250.
LaTeX Font Info:    Redeclaring math accent \bar on input line 251.
LaTeX Font Info:    Redeclaring math accent \dot on input line 252.
LaTeX Font Info:    Redeclaring math accent \hat on input line 254.
) (c:/texlive/2024/texmf-dist/tex/latex/merriweather/merriweather.sty
Package: merriweather 2022/09/20 (Bob Tennent) Supports
Merriweather(Sans) font
s for all LaTeX engines.
(c:/texlive/2024/texmf-dist/tex/generic/iftex/ifxetex.sty
Package: ifxetex 2019/10/25 v0.7 ifxetex legacy package. Use iftex
instead.
) (c:/texlive/2024/texmf-dist/tex/generic/iftex/ifluatex.sty
Package: ifluatex 2019/10/25 v1.5 ifluatex legacy package. Use iftex
instead.
) (c:/texlive/2024/texmf-dist/tex/latex/base/textcomp.sty
Package: textcomp 2024/04/24 v2.1b Standard LaTeX package
) (c:/texlive/2024/texmf-dist/tex/latex/xkeyval/xkeyval.sty
Package: xkeyval 2022/06/16 v2.9 package option processing (HA)
(c:/texlive/2024/texmf-dist/tex/generic/xkeyval/xkeyval.tex
(c:/texlive/2024/te
xmf-dist/tex/generic/xkeyval/xkvutils.tex
\XKV@toks=\toks22
\XKV@tempa@toks=\toks23
)
\XKV@depth=\count271
```

```
File: xkeyval.tex 2014/12/03 v2.7a key=value parser (HA)
)) (c:/texlive/2024/texmf-dist/tex/latex/base/fontenc.sty
Package: fontenc 2021/04/29 v2.0v Standard LaTeX package
) (c:/texlive/2024/texmf-dist/tex/latex/fontaxes/fontaxes.sty
Package: fontaxes 2020/07/21 v1.0e Font selection axes
LaTeX Info: Redefining \upshape on input line 29.
LaTeX Info: Redefining \itshape on input line 31.
LaTeX Info: Redefining \slshape on input line 33.
LaTeX Info: Redefining \swshape on input line 35.
LaTeX Info: Redefining \scshape on input line 37.
LaTeX Info: Redefining \sscshape on input line 39.
LaTeX Info: Redefining \ulcshape on input line 41.
LaTeX Info: Redefining \textsw on input line 47.
LaTeX Info: Redefining \textssc on input line 48.
LaTeX Info: Redefining \textulc on input line 49.
)) (c:/texlive/2024/texmf-dist/tex/latex/mathastext/mathastext.sty
Package: mathastext 2024/07/27 v1.4b Use the text font in math mode (JFB)

Package mathastext Info: Starting the math mode configuration.
\mst@exists@muskip=\muskip17
\mst@forall@muskip=\muskip18
\mst@prime@muskip=\muskip19
\mst@do@nonletters=\toks24
\mst@undo@nonletters=\toks25
\mst@do@easynonletters=\toks26
\mst@undo@easynonletters=\toks27
\symmtoperatorfont=\mathgroup8
\symmtletterfont=\mathgroup9
(   mathastext:   ) ! and ?
(   mathastext:   ) punctuation: , . : ; and \colon
LaTeX Info: Redefining \relbar on input line 1201.
LaTeX Info: Redefining \rightarrowfill on input line 1202.
LaTeX Info: Redefining \leftarrowfill on input line 1205.
(   mathastext:   ) + and =
LaTeX Info: Redefining \Relbar on input line 1298.
(   mathastext:   ) adding = ; and + to \nfss@catcodes
(   mathastext:   ) parentheses ( ) [ ] and slash /
(   mathastext:   ) alldelims: < > \backslash \setminus | \vert \mid \{
\}
LaTeX Font Info:    Redeclaring math symbol \setminus on input line 1364.
LaTeX Info: Redefining \models on input line 1383.
(   mathastext:   ) \# \mathdollar \% \&
(   mathastext:   ) \imath and \jmath
LaTeX Font Info:    Overwriting math alphabet `\Mathnormalbold' in
version `nor
mal'
(Font)                  T1/Merriwthr-OsF/b/it --> T1/Merriwthr-OsF/b/it
on inpu
t line 2863.
LaTeX Font Info:    Overwriting math alphabet `\Mathnormalbold' in
version `bol
d'
(Font)                  T1/Merriwthr-OsF/b/it --> T1/Merriwthr-OsF/b/it
on inpu
```

```
t line 2863.
LaTeX Font Info:    Overwriting symbol font `mtletterfont' in version
`normal'
(Font)                  T1/Merriwthr-OsF/m/it --> T1/Merriwthr-OsF/m/it
on inpu
t line 2863.
LaTeX Font Info:    Overwriting symbol font `mtletterfont' in version
`bold'
(Font)                  T1/Merriwthr-OsF/m/it --> T1/Merriwthr-OsF/b/it
on inpu
t line 2863.
LaTeX Font Info:    Overwriting symbol font `mtoperatorfont' in version
`normal
'
(Font)                  T1/Merriwthr-OsF/m/n --> T1/Merriwthr-OsF/m/n on
input
line 2863.
LaTeX Font Info:    Overwriting symbol font `mtoperatorfont' in version
`bold'
(Font)                  T1/Merriwthr-OsF/m/n --> T1/Merriwthr-OsF/b/n on
input
line 2863.
LaTeX Font Info:    Overwriting math alphabet `\Mathbf' in version
`normal'
(Font)                  T1/Merriwthr-OsF/b/n --> T1/Merriwthr-OsF/b/n on
input
line 2863.
LaTeX Font Info:    Overwriting math alphabet `\Mathbf' in version `bold'
(Font)                  T1/Merriwthr-OsF/b/n --> T1/Merriwthr-OsF/b/n on
input
line 2863.
LaTeX Font Info:    Overwriting math alphabet `\Mathit' in version
`normal'
(Font)                  T1/Merriwthr-OsF/m/it --> T1/Merriwthr-OsF/m/it
on inpu
t line 2863.
LaTeX Font Info:    Overwriting math alphabet `\Mathit' in version `bold'
(Font)                  T1/Merriwthr-OsF/m/it --> T1/Merriwthr-OsF/b/it
on inpu
t line 2863.
LaTeX Font Info:    Overwriting math alphabet `\Mathsf' in version
`normal'
(Font)                  T1/MerriwthrSans-OsF/m/n --> T1/MerriwthrSans-
OsF/m/n o
n input line 2863.
LaTeX Font Info:    Overwriting math alphabet `\Mathsf' in version `bold'
(Font)                  T1/MerriwthrSans-OsF/m/n --> T1/MerriwthrSans-
OsF/b/n o
n input line 2863.
LaTeX Font Info:    Overwriting math alphabet `\Mathtt' in version
`normal'
(Font)                  T1/lmtt/m/n --> T1/lmtt/m/n on input line 2863.
LaTeX Font Info:    Overwriting math alphabet `\Mathtt' in version `bold'
(Font)                  T1/lmtt/m/n --> T1/lmtt/b/n on input line 2863.
```

```
(   mathastext:   ) Latin letters in the `normal', resp. `bold',
(   mathastext:   ) math versions are now set up to use the fonts
(   mathastext:   ) T1/Merriwthr-OsF/m/it, resp. T1/Merriwthr-OsF/b/it.
(   mathastext:   ) Other characters (digits, ...) and \log-like names
will be
(   mathastext:   ) typeset with the n shape.
(   mathastext:   ) \hbar
(   mathastext:   ) minus as endash
(   mathastext:   ) The italic option is in effect.
(   mathastext:   ) \HUGE has been (re)-defined.
(   mathastext:   ) mathastext has declared larger sizes for subscripts.
(   mathastext:   ) To keep LaTeX defaults, use option
`defaultmathsizes'.

Package mathastext Info: Loading is complete.  You can now use
\Mathastext to
(mathastext)             modify the normal and bold math versions.  Use
it
(mathastext)             with optional argument or use \MTDeclareVersion
to
(mathastext)             declare additional math versions.
) (c:/texlive/2024/texmf-dist/tex/latex/relsize/relsize.sty
Package: relsize 2013/03/29 ver 4.1
) (c:/texlive/2024/texmf-dist/tex/latex/ragged2e/ragged2e.sty
Package: ragged2e 2023/06/22 v3.6 ragged2e Package
\CenteringLeftskip=\skip52
\RaggedLeftLeftskip=\skip53
\RaggedRightLeftskip=\skip54
\CenteringRightskip=\skip55
\RaggedLeftRightskip=\skip56
\RaggedRightRightskip=\skip57
\CenteringParfillskip=\skip58
\RaggedLeftParfillskip=\skip59
\RaggedRightParfillskip=\skip60
\JustifyingParfillskip=\skip61
\CenteringParindent=\skip62
\RaggedLeftParindent=\skip63
\RaggedRightParindent=\skip64
\JustifyingParindent=\skip65
) (c:/texlive/2024/texmf-dist/tex/latex/xcolor/xcolor.sty
Package: xcolor 2023/11/15 v3.01 LaTeX color extensions (UK)
(c:/texlive/2024/texmf-dist/tex/latex/graphics-cfg/color.cfg
File: color.cfg 2016/01/02 v1.6 sample color configuration
)
Package xcolor Info: Driver file: pdftex.def on input line 274.
(c:/texlive/2024/texmf-dist/tex/latex/graphics-def/pdftex.def
File: pdftex.def 2024/04/13 v1.2c Graphics/color driver for pdftex
) (c:/texlive/2024/texmf-dist/tex/latex/graphics/mathcolor.ltx)
Package xcolor Info: Model `cmy' substituted by `cmy0' on input line
1350.
Package xcolor Info: Model `hsb' substituted by `rgb' on input line 1354.
Package xcolor Info: Model `RGB' extended on input line 1366.
Package xcolor Info: Model `HTML' substituted by `rgb' on input line
1368.
```

```
Package xcolor Info: Model `Hsb' substituted by `hsb' on input line 1369.
Package xcolor Info: Model `tHsb' substituted by `hsb' on input line
1370.
Package xcolor Info: Model `HSB' substituted by `hsb' on input line 1371.
Package xcolor Info: Model `Gray' substituted by `gray' on input line
1372.
Package xcolor Info: Model `wave' substituted by `hsb' on input line
1373.
) (c:/texlive/2024/texmf-dist/tex/latex/colortbl/colortbl.sty
Package: colortbl 2024/07/06 v1.0i Color table columns (DPC)
(c:/texlive/2024/texmf-dist/tex/latex/tools/array.sty
Package: array 2024/06/14 v2.6d Tabular extension package (FMi)
\col@sep=\dimen143
\ar@mcellbox=\box53
\extrarowheight=\dimen144
\NC@list=\toks28
\extratabsurround=\skip66
\backup@length=\skip67
\ar@cellbox=\box54
)
\everycr=\toks29
\minrowclearance=\skip68
\rownum=\count272
) (c:/texlive/2024/texmf-dist/tex/latex/graphics/graphicx.sty
Package: graphicx 2021/09/16 v1.2d Enhanced LaTeX Graphics (DPC,SPQR)
(c:/texlive/2024/texmf-dist/tex/latex/graphics/graphics.sty
Package: graphics 2024/05/23 v1.4g Standard LaTeX Graphics (DPC,SPQR)
(c:/texlive/2024/texmf-dist/tex/latex/graphics/trig.sty
Package: trig 2023/12/02 v1.11 sin cos tan (DPC)
) (c:/texlive/2024/texmf-dist/tex/latex/graphics-cfg/graphics.cfg
File: graphics.cfg 2016/06/04 v1.11 sample graphics configuration
)
Package graphics Info: Driver file: pdftex.def on input line 106.
)
\Gin@req@height=\dimen145
\Gin@req@width=\dimen146
) (c:/texlive/2024/texmf-dist/tex/latex/xpatch/xpatch.sty
(c:/texlive/2024/texm
f-dist/tex/latex/l3kernel/expl3.sty
Package: expl3 2024-05-27 L3 programming layer (loader)
(c:/texlive/2024/texmf-dist/tex/latex/l3backend/l3backend-pdftex.def
File: l3backend-pdftex.def 2024-05-08 L3 backend support: PDF output
(pdfTeX)
\l__color_backend_stack_int=\count273
\l__pdf_internal_box=\box55
))
Package: xpatch 2020/03/25 v0.3a Extending etoolbox patching commands
(c:/texlive/2024/texmf-dist/tex/latex/l3packages/xparse/xparse.sty
Package: xparse 2024-05-08 L3 Experimental document command parser
)) (c:/texlive/2024/texmf-dist/tex/latex/environ/environ.sty
Package: environ 2014/05/04 v0.3 A new way to define environments
(c:/texlive/2024/texmf-dist/tex/latex/trimspaces/trimspaces.sty
Package: trimspaces 2009/09/17 v1.1 Trim spaces around a token list
)
```

```
\@envbody=\toks30
) (c:/texlive/2024/texmf-dist/tex/latex/lastpage/lastpage.sty
Package: lastpage 2024/07/07 v2.1c lastpage: 2.09 or 2e? (HMM)
(c:/texlive/2024/texmf-dist/tex/latex/lastpage/lastpage2e.sty
Package: lastpage2e 2024/07/07 v2.1c Decide which 2e lastpage version to
use (H
MM)
(c:/texlive/2024/texmf-dist/tex/latex/lastpage/lastpagemodern.sty
Package: lastpagemodern 2024-07-07 v2.1c Refers to last page's name (HMM;
JPG)
\c@lastpagecount=\count274
)
)) (c:/texlive/2024/texmf-dist/tex/latex/graphics/rotating.sty
Package: rotating 2016/08/11 v2.16d rotated objects in LaTeX
(c:/texlive/2024/texmf-dist/tex/latex/base/ifthen.sty
Package: ifthen 2024/03/16 v1.1e Standard LaTeX ifthen package (DPC)
)
\c@r@tfl@t=\count275
\rotFPtop=\skip69
\rotFPbot=\skip70
\rot@float@box=\box56
\rot@mess@toks=\toks31
) (c:/texlive/2024/texmf-dist/tex/latex/graphics/lscape.sty
Package: lscape 2020/05/28 v3.02 Landscape Pages (DPC)
) (c:/texlive/2024/texmf-dist/tex/latex/tools/afterpage.sty
Package: afterpage 2023/07/04 v1.08 After-Page Package (DPC)
\AP@output=\toks32
\AP@partial=\box57
\AP@footins=\box58
) (c:/texlive/2024/texmf-dist/tex/latex/textpos/textpos.sty
Package: textpos 2022/07/23 v1.10.1
Package textpos Info: choosing support for LaTeX3 on input line 60.
\TP@textbox=\box59
\TP@holdbox=\box60
\TPHorizModule=\dimen147
\TPVertModule=\dimen148
\TP@margin=\dimen149
\TP@absmargin=\dimen150
Grid set 16 x 16 = 37.34424pt x 52.81541pt
\TPboxrulesize=\dimen151
\TP@ox=\dimen152
\TP@oy=\dimen153
\TP@tbargs=\toks33
TextBlockOrigin set to 0pt x 0pt
) (c:/texlive/2024/texmf-dist/tex/latex/url/url.sty
\Urlmuskip=\muskip20
Package: url 2013/09/16  ver 3.4  Verb mode for urls, etc.
) (c:/texlive/2024/texmf-dist/tex/latex/newfloat/newfloat.sty
Package: newfloat 2023/10/01 v1.2 Defining new floating environments (AR)
Package newfloat Info: `rotating' package detected.
) (c:/texlive/2024/texmf-dist/tex/latex/mdframed/mdframed.sty
Package: mdframed 2013/07/01 1.9b: mdframed
(c:/texlive/2024/texmf-dist/tex/latex/kvoptions/kvoptions.sty
```

```
Package: kvoptions 2022-06-15 v3.15 Key value format for package options
(HO)
(c:/texlive/2024/texmf-dist/tex/generic/ltxcmds/ltxcmds.sty
Package: ltxcmds 2023-12-04 v1.26 LaTeX kernel commands for general use
(HO)
) (c:/texlive/2024/texmf-dist/tex/latex/kvsetkeys/kvsetkeys.sty
Package: kvsetkeys 2022-10-05 v1.19 Key value parser (HO)
)) (c:/texlive/2024/texmf-dist/tex/latex/zref/zref-abspage.sty
Package: zref-abspage 2023-09-14 v2.35 Module abspage for zref (HO)
(c:/texlive/2024/texmf-dist/tex/latex/zref/zref-base.sty
Package: zref-base 2023-09-14 v2.35 Module base for zref (HO)
(c:/texlive/2024/texmf-dist/tex/generic/infwarerr/infwarerr.sty
Package: infwarerr 2019/12/03 v1.5 Providing info/warning/error messages
(HO)
) (c:/texlive/2024/texmf-dist/tex/generic/kvdefinekeys/kvdefinekeys.sty
Package: kvdefinekeys 2019-12-19 v1.6 Define keys (HO)
) (c:/texlive/2024/texmf-dist/tex/generic/pdftexcmds/pdftexcmds.sty
Package: pdftexcmds 2020-06-27 v0.33 Utility functions of pdfTeX for
LuaTeX (HO
)
Package pdftexcmds Info: \pdf@primitive is available.
Package pdftexcmds Info: \pdf@ifprimitive is available.
Package pdftexcmds Info: \pdfdraftmode found.
) (c:/texlive/2024/texmf-dist/tex/generic/etexcmds/etexcmds.sty
Package: etexcmds 2019/12/15 v1.7 Avoid name clashes with e-TeX commands
(HO)
) (c:/texlive/2024/texmf-dist/tex/latex/auxhook/auxhook.sty
Package: auxhook 2019-12-17 v1.6 Hooks for auxiliary files (HO)
)
Package zref Info: New property list: main on input line 767.
Package zref Info: New property: default on input line 768.
Package zref Info: New property: page on input line 769.
)
\c@abspage=\count276
Package zref Info: New property: abspage on input line 67.
) (c:/texlive/2024/texmf-dist/tex/latex/needspace/needspace.sty
Package: needspace 2010/09/12 v1.3d reserve vertical space
)
\mdf@templength=\skip71
\c@mdf@globalstyle@cnt=\count277
\mdf@skipabove@length=\skip72
\mdf@skipbelow@length=\skip73
\mdf@leftmargin@length=\skip74
\mdf@rightmargin@length=\skip75
\mdf@innerleftmargin@length=\skip76
\mdf@innerrightmargin@length=\skip77
\mdf@innertopmargin@length=\skip78
\mdf@innerbottommargin@length=\skip79
\mdf@splittopskip@length=\skip80
\mdf@splitbottomskip@length=\skip81
\mdf@outermargin@length=\skip82
\mdf@innermargin@length=\skip83
\mdf@linewidth@length=\skip84
\mdf@innerlinewidth@length=\skip85
```

```
\mdf@middlelinewidth@length=\skip86
\mdf@outerlinewidth@length=\skip87
\mdf@roundcorner@length=\skip88
\mdf@footenotedistance@length=\skip89
\mdf@userdefinedwidth@length=\skip90
\mdf@needspace@length=\skip91
\mdf@frametitleaboveskip@length=\skip92
\mdf@frametitlebelowskip@length=\skip93
\mdf@frametitlerulewidth@length=\skip94
\mdf@frametitleleftmargin@length=\skip95
\mdf@frametitlerightmargin@length=\skip96
\mdf@shadowsize@length=\skip97
\mdf@extratopheight@length=\skip98
\mdf@subtitleabovelinewidth@length=\skip99
\mdf@subtitlebelowlinewidth@length=\skip100
\mdf@subtitleaboveskip@length=\skip101
\mdf@subtitlebelowskip@length=\skip102
\mdf@subtitleinneraboveskip@length=\skip103
\mdf@subtitleinnerbelowskip@length=\skip104
\mdf@subsubtitleabovelinewidth@length=\skip105
\mdf@subsubtitlebelowlinewidth@length=\skip106
\mdf@subsubtitleaboveskip@length=\skip107
\mdf@subsubtitlebelowskip@length=\skip108
\mdf@subsubtitleinneraboveskip@length=\skip109
\mdf@subsubtitleinnerbelowskip@length=\skip110
(c:/texlive/2024/texmf-dist/tex/latex/mdframed/md-frame-0.mdf
File: md-frame-0.mdf 2013/07/01\ 1.9b: md-frame-0
)
\mdf@frametitlebox=\box61
\mdf@footnotebox=\box62
\mdf@splitbox@one=\box63
\mdf@splitbox@two=\box64
\mdf@splitbox@save=\box65
\mdfsplitboxwidth=\skip111
\mdfsplitboxtotalwidth=\skip112
\mdfsplitboxheight=\skip113
\mdfsplitboxdepth=\skip114
\mdfsplitboxtotalheight=\skip115
\mdfframetitleboxwidth=\skip116
\mdfframetitleboxtotalwidth=\skip117
\mdfframetitleboxheight=\skip118
\mdfframetitleboxdepth=\skip119
\mdfframetitleboxtotalheight=\skip120
\mdffootnoteboxwidth=\skip121
\mdffootnoteboxtotalwidth=\skip122
\mdffootnoteboxheight=\skip123
\mdffootnoteboxdepth=\skip124
\mdffootnoteboxtotalheight=\skip125
\mdftotallinewidth=\skip126
\mdfboundingboxwidth=\skip127
\mdfboundingboxtotalwidth=\skip128
\mdfboundingboxheight=\skip129
\mdfboundingboxdepth=\skip130
\mdfboundingboxtotalheight=\skip131
```

```
\mdf@freevspace@length=\skip132
\mdf@horizontalwidthofbox@length=\skip133
\mdf@verticalmarginwhole@length=\skip134
\mdf@horizontalspaceofbox=\skip135
\mdfsubtitleheight=\skip136
\mdfsubsubtitleheight=\skip137
\c@mdfcountframes=\count278

****** mdframed patching \endmdf@trivlist

****** -- success******

\mdf@envdepth=\count279
\c@mdf@env@i=\count280
\c@mdf@env@ii=\count281
\c@mdf@zref@counter=\count282
Package zref Info: New property: mdf@pagevalue on input line 895.
) (c:/texlive/2024/texmf-dist/tex/latex/titlesec/titlesec.sty
Package: titlesec 2023/10/27 v2.16 Sectioning titles
\ttl@box=\box66
\beforetitleunit=\skip138
\aftertitleunit=\skip139
\ttl@plus=\dimen154
\ttl@minus=\dimen155
\ttl@toksa=\toks34
\titlewidth=\dimen156
\titlewidthlast=\dimen157
\titlewidthfirst=\dimen158
) (c:/texlive/2024/texmf-dist/tex/latex/koma-script/scrextend.sty
Package: scrextend 2023/07/07 v3.41 KOMA-Script package (extend other
classes w
ith features of KOMA-Script classes)
(c:/texlive/2024/texmf-dist/tex/latex/koma-script/scrkbase.sty
Package: scrkbase 2023/07/07 v3.41 KOMA-Script package (KOMA-Script-
dependent b
asics and keyval usage)
(c:/texlive/2024/texmf-dist/tex/latex/koma-script/scrbase.sty
Package: scrbase 2023/07/07 v3.41 KOMA-Script package (KOMA-Script-
independent
basics and keyval usage)
(c:/texlive/2024/texmf-dist/tex/latex/koma-script/scrlfile.sty
Package: scrlfile 2023/07/07 v3.41 KOMA-Script package (file load hooks)
(c:/texlive/2024/texmf-dist/tex/latex/koma-script/scrlfile-hook.sty
Package: scrlfile-hook 2023/07/07 v3.41 KOMA-Script package (using LaTeX
hooks)

(c:/texlive/2024/texmf-dist/tex/latex/koma-script/scrlogo.sty
Package: scrlogo 2023/07/07 v3.41 KOMA-Script package (logo)
)))
Applying: [2021/05/01] Usage of raw or classic option list on input line
252.
Already applied: [0000/00/00] Usage of raw or classic option list on
input line
 368.
```

```
))
Package scrextend Info: unexpected definition of `\@makefnmark'.
(scrextend)             Trying to patch it on input line 1762.
Package scrextend Info: patch seems to be successfull on input line 1762.
)

LaTeX Font Warning: Font shape `T1/cmr/m/n' in size <7.5> not available
(Font)              size <7> substituted on input line 69.

(c:/texlive/2024/texmf-dist/tex/latex/tools/calc.sty
Package: calc 2023/07/08 v4.3 Infix arithmetic (KKT,FJ)
\calc@Acount=\count283
\calc@Bcount=\count284
\calc@Adimen=\dimen159
\calc@Bdimen=\dimen160
\calc@Askip=\skip140
\calc@Bskip=\skip141
LaTeX Info: Redefining \setlength on input line 80.
LaTeX Info: Redefining \addtolength on input line 81.
\calc@Ccount=\count285
\calc@Cskip=\skip142
) (c:/texlive/2024/texmf-dist/tex/latex/geometry/geometry.sty
Package: geometry 2020/01/02 v5.9 Page Geometry
(c:/texlive/2024/texmf-dist/tex/generic/iftex/ifvtex.sty
Package: ifvtex 2019/10/25 v1.7 ifvtex legacy package. Use iftex instead.
)
\Gm@cnth=\count286
\Gm@cntv=\count287
\c@Gm@tempcnt=\count288
\Gm@bindingoffset=\dimen161
\Gm@wd@mp=\dimen162
\Gm@odd@mp=\dimen163
\Gm@even@mp=\dimen164
\Gm@layoutwidth=\dimen165
\Gm@layoutheight=\dimen166
\Gm@layouthoffset=\dimen167
\Gm@layoutvoffset=\dimen168
\Gm@dimlist=\toks35
) (c:/texlive/2024/texmf-dist/tex/latex/preprint/authblk.sty
Package: authblk 2001/02/27 1.3 (PWD)
\affilsep=\skip143
\@affilsep=\skip144
\c@Maxaffil=\count289
\c@authors=\count290
\c@affil=\count291
) (c:/texlive/2024/texmf-dist/tex/latex/footmisc/footmisc.sty
Package: footmisc 2023/07/05 v6.0f a miscellany of footnote facilities
\FN@temptoken=\toks36
\footnotemargin=\dimen169
\@outputbox@depth=\dimen170
Package footmisc Info: Declaring symbol style bringhurst on input line
696.
Package footmisc Info: Declaring symbol style chicago on input line 704.
Package footmisc Info: Declaring symbol style wiley on input line 713.
```

```
Package footmisc Info: Declaring symbol style lamport-robust on input
line 724.

Package footmisc Info: Declaring symbol style lamport* on input line 744.
Package footmisc Info: Declaring symbol style lamport*-robust on input
line 765
.
) (c:/texlive/2024/texmf-dist/tex/latex/fancyhdr/fancyhdr.sty
Package: fancyhdr 2024/07/23 v4.3.1 Extensive control of page headers and
foote
rs
\f@nch@headwidth=\skip145
\f@nch@O@elh=\skip146
\f@nch@O@erh=\skip147
\f@nch@O@olh=\skip148
\f@nch@O@orh=\skip149
\f@nch@O@elf=\skip150
\f@nch@O@erf=\skip151
\f@nch@O@olf=\skip152
\f@nch@O@orf=\skip153
) (c:/texlive/2024/texmf-dist/tex/generic/alphalph/alphalph.sty
Package: alphalph 2019/12/09 v2.6 Convert numbers to letters (HO)
(c:/texlive/2024/texmf-dist/tex/generic/intcalc/intcalc.sty
Package: intcalc 2019/12/15 v1.3 Expandable calculations with integers
(HO)
))
\c@authorfn=\count292
(c:/texlive/2024/texmf-dist/tex/latex/abstract/abstract.sty
Package: abstract 2009/06/08 v1.2a configurable abstracts
\abstitleskip=\skip154
\absleftindent=\skip155
\absrightindent=\skip156
\absparindent=\skip157
\absparsep=\skip158
)
Package newfloat Info: New float `keypoints' with options
`placement=t!,name=kp
t' on input line 291.
\c@keypoints=\count293
\newfloat@ftype=\count294
Package newfloat Info: float type `keypoints'=8 on input line 291.
(c:/texlive/2024/texmf-dist/tex/latex/enumitem/enumitem.sty
Package: enumitem 2019/06/20 v3.9 Customized lists
\labelindent=\skip159
\enit@outerparindent=\dimen171
\enit@toks=\toks37
\enit@inbox=\box67
\enit@count@id=\count295
\enitdp@description=\count296
) (c:/texlive/2024/texmf-dist/tex/latex/quoting/quoting.sty
Package: quoting 2014/01/28 v0.1c Consolidated environment for displayed
text
\quo@toppartop=\skip160
) (c:/texlive/2024/texmf-dist/tex/latex/sttools/stfloats.sty
```

```
Package: stfloats 2017/03/27 v3.3 Improve float mechanism and
baselineskip sett
ings
\@dblbotnum=\count297
\c@dblbotnumber=\count298
) (c:/texlive/2024/texmf-dist/tex/latex/booktabs/booktabs.sty
Package: booktabs 2020/01/12 v1.61803398 Publication quality tables
\heavyrulewidth=\dimen172
\lightrulewidth=\dimen173
\cmidrulewidth=\dimen174
\belowrulesep=\dimen175
\belowbottomsep=\dimen176
\aboverulesep=\dimen177
\abovetopsep=\dimen178
\cmidrulesep=\dimen179
\cmidrulekern=\dimen180
\defaultaddspace=\dimen181
\@cmidla=\count299
\@cmidlb=\count300
\@aboverulesep=\dimen182
\@belowrulesep=\dimen183
\@thisruleclass=\count301
\@lastruleclass=\count302
\@thisrulewidth=\dimen184
) (c:/texlive/2024/texmf-dist/tex/latex/tools/tabularx.sty
Package: tabularx 2023/12/11 v2.12a `tabularx' package (DPC)
\TX@col@width=\dimen185
\TX@old@table=\dimen186
\TX@old@col=\dimen187
\TX@target=\dimen188
\TX@delta=\dimen189
\TX@cols=\count303
\TX@ftn=\toks38
)
\enitdp@tablenotes=\count304
(c:/texlive/2024/texmf-dist/tex/latex/caption/caption.sty
Package: caption 2023/08/05 v3.6o Customizing captions (AR)
(c:/texlive/2024/texmf-dist/tex/latex/caption/caption3.sty
Package: caption3 2023/07/31 v2.4d caption3 kernel (AR)
\caption@tempdima=\dimen190
\captionmargin=\dimen191
\caption@leftmargin=\dimen192
\caption@rightmargin=\dimen193
\caption@width=\dimen194
\caption@indent=\dimen195
\caption@parindent=\dimen196
\caption@hangindent=\dimen197
Package caption Info: Standard document class detected.
)
\c@caption@flags=\count305
\c@continuedfloat=\count306
Package caption Info: rotating package is loaded.
Package caption Info: scrextend package is loaded.
\caption@addmargin@hsize=\dimen198
```

```
\caption@addmargin@linewidth=\dimen199
) (c:/texlive/2024/texmf-dist/tex/latex/natbib/natbib.sty
Package: natbib 2010/09/13 8.31b (PWD, AO)
\bibhang=\skip161
\bibsep=\skip162
LaTeX Info: Redefining \cite on input line 694.
\c@NAT@ctr=\count307
)) (c:/texlive/2024/texmf-dist/tex/latex/siunitx/siunitx.sty
Package: siunitx 2024-06-24 v3.3.19 A comprehensive (SI) units package
\l__siunitx_number_uncert_offset_int=\count308
\l__siunitx_number_exponent_fixed_int=\count309
\l__siunitx_number_min_decimal_int=\count310
\l__siunitx_number_min_integer_int=\count311
\l__siunitx_number_round_precision_int=\count312
\l__siunitx_number_lower_threshold_int=\count313
\l__siunitx_number_upper_threshold_int=\count314
\l__siunitx_number_group_first_int=\count315
\l__siunitx_number_group_size_int=\count316
\l__siunitx_number_group_minimum_int=\count317
\l__siunitx_angle_tmp_dim=\dimen256
\l__siunitx_angle_marker_box=\box68
\l__siunitx_angle_unit_box=\box69
\l__siunitx_compound_count_int=\count318
(c:/texlive/2024/texmf-dist/tex/latex/translations/translations.sty
Package: translations 2022/02/05 v1.12 internationalization of LaTeX2e
packages
 (CN)
) (c:/texlive/2024/texmf-dist/tex/latex/amsmath/amstext.sty
Package: amstext 2021/08/26 v2.01 AMS text
(c:/texlive/2024/texmf-dist/tex/latex/amsmath/amsgen.sty
File: amsgen.sty 1999/11/30 v2.0 generic functions
\@emptytoks=\toks39
\ex@=\dimen257
))
\l__siunitx_table_tmp_box=\box70
\l__siunitx_table_tmp_dim=\dimen258
\l__siunitx_table_column_width_dim=\dimen259
\l__siunitx_table_integer_box=\box71
\l__siunitx_table_decimal_box=\box72
\l__siunitx_table_uncert_box=\box73
\l__siunitx_table_before_box=\box74
\l__siunitx_table_after_box=\box75
\l__siunitx_table_before_dim=\dimen260
\l__siunitx_table_carry_dim=\dimen261
\l__siunitx_unit_tmp_int=\count319
\l__siunitx_unit_position_int=\count320
\l__siunitx_unit_total_int=\count321
) (c:/texlive/2024/texmf-dist/tex/latex/pgf/frontendlayer/tikz.sty
(c:/texlive/
2024/texmf-dist/tex/latex/pgf/basiclayer/pgf.sty (c:/texlive/2024/texmf-
dist/te
x/latex/pgf/utilities/pgfrcs.sty (c:/texlive/2024/texmf-
dist/tex/generic/pgf/ut
ilities/pgfutil-common.tex
```

```
\pgfutil@everybye=\toks40
\pgfutil@tempdima=\dimen262
\pgfutil@tempdimb=\dimen263
) (c:/texlive/2024/texmf-dist/tex/generic/pgf/utilities/pgfutil-latex.def
\pgfutil@abb=\box76
) (c:/texlive/2024/texmf-dist/tex/generic/pgf/utilities/pgfrcs.code.tex
(c:/tex
live/2024/texmf-dist/tex/generic/pgf/pgf.revision.tex)
Package: pgfrcs 2023-01-15 v3.1.10 (3.1.10)
))
Package: pgf 2023-01-15 v3.1.10 (3.1.10)
(c:/texlive/2024/texmf-dist/tex/latex/pgf/basiclayer/pgfcore.sty
(c:/texlive/20
24/texmf-dist/tex/latex/pgf/systemlayer/pgfsys.sty
(c:/texlive/2024/texmf-dist/
tex/generic/pgf/systemlayer/pgfsys.code.tex
Package: pgfsys 2023-01-15 v3.1.10 (3.1.10)
(c:/texlive/2024/texmf-dist/tex/generic/pgf/utilities/pgfkeys.code.tex
\pgfkeys@pathtoks=\toks41
\pgfkeys@temptoks=\toks42

(c:/texlive/2024/texmf-
dist/tex/generic/pgf/utilities/pgfkeyslibraryfiltered.co
de.tex
\pgfkeys@tmptoks=\toks43
))
\pgf@x=\dimen264
\pgf@y=\dimen265
\pgf@xa=\dimen266
\pgf@ya=\dimen267
\pgf@xb=\dimen268
\pgf@yb=\dimen269
\pgf@xc=\dimen270
\pgf@yc=\dimen271
\pgf@xd=\dimen272
\pgf@yd=\dimen273
\w@pgf@writea=\write3
\r@pgf@reada=\read2
\c@pgf@counta=\count322
\c@pgf@countb=\count323
\c@pgf@countc=\count324
\c@pgf@countd=\count325
\t@pgf@toka=\toks44
\t@pgf@tokb=\toks45
\t@pgf@tokc=\toks46
\pgf@sys@id@count=\count326
(c:/texlive/2024/texmf-dist/tex/generic/pgf/systemlayer/pgf.cfg
File: pgf.cfg 2023-01-15 v3.1.10 (3.1.10)
)
Driver file for pgf: pgfsys-pdftex.def
(c:/texlive/2024/texmf-dist/tex/generic/pgf/systemlayer/pgfsys-pdftex.def
File: pgfsys-pdftex.def 2023-01-15 v3.1.10 (3.1.10)
(c:/texlive/2024/texmf-dist/tex/generic/pgf/systemlayer/pgfsys-common-
pdf.def
```

```
File: pgfsys-common-pdf.def 2023-01-15 v3.1.10 (3.1.10)
)))
(c:/texlive/2024/texmf-
dist/tex/generic/pgf/systemlayer/pgfsyssoftpath.code.tex
File: pgfsyssoftpath.code.tex 2023-01-15 v3.1.10 (3.1.10)
\pgfsyssoftpath@smallbuffer@items=\count327
\pgfsyssoftpath@bigbuffer@items=\count328
)
(c:/texlive/2024/texmf-
dist/tex/generic/pgf/systemlayer/pgfsysprotocol.code.tex
File: pgfsysprotocol.code.tex 2023-01-15 v3.1.10 (3.1.10)
)) (c:/texlive/2024/texmf-
dist/tex/generic/pgf/basiclayer/pgfcore.code.tex
Package: pgfcore 2023-01-15 v3.1.10 (3.1.10)
(c:/texlive/2024/texmf-dist/tex/generic/pgf/math/pgfmath.code.tex
(c:/texlive/2
024/texmf-dist/tex/generic/pgf/math/pgfmathutil.code.tex)
(c:/texlive/2024/texm
f-dist/tex/generic/pgf/math/pgfmathparser.code.tex
\pgfmath@dimen=\dimen274
\pgfmath@count=\count329
\pgfmath@box=\box77
\pgfmath@toks=\toks47
\pgfmath@stack@operand=\toks48
\pgfmath@stack@operation=\toks49
) (c:/texlive/2024/texmf-
dist/tex/generic/pgf/math/pgfmathfunctions.code.tex)
(c:/texlive/2024/texmf-
dist/tex/generic/pgf/math/pgfmathfunctions.basic.code.te
x)
(c:/texlive/2024/texmf-
dist/tex/generic/pgf/math/pgfmathfunctions.trigonometric
.code.tex)
(c:/texlive/2024/texmf-
dist/tex/generic/pgf/math/pgfmathfunctions.random.code.t
ex)
(c:/texlive/2024/texmf-
dist/tex/generic/pgf/math/pgfmathfunctions.comparison.co
de.tex)
(c:/texlive/2024/texmf-
dist/tex/generic/pgf/math/pgfmathfunctions.base.code.tex
)
(c:/texlive/2024/texmf-
dist/tex/generic/pgf/math/pgfmathfunctions.round.code.te
x)
(c:/texlive/2024/texmf-
dist/tex/generic/pgf/math/pgfmathfunctions.misc.code.tex
)
(c:/texlive/2024/texmf-
dist/tex/generic/pgf/math/pgfmathfunctions.integerarithm
etics.code.tex) (c:/texlive/2024/texmf-
dist/tex/generic/pgf/math/pgfmathcalc.co
de.tex) (c:/texlive/2024/texmf-
dist/tex/generic/pgf/math/pgfmathfloat.code.tex
```

```
\c@pgfmathroundto@lastzeros=\count330
)) (c:/texlive/2024/texmf-dist/tex/generic/pgf/math/pgfint.code.tex)
(c:/texliv
e/2024/texmf-dist/tex/generic/pgf/basiclayer/pgfcorepoints.code.tex
File: pgfcorepoints.code.tex 2023-01-15 v3.1.10 (3.1.10)
\pgf@picminx=\dimen275
\pgf@picmaxx=\dimen276
\pgf@picminy=\dimen277
\pgf@picmaxy=\dimen278
\pgf@pathminx=\dimen279
\pgf@pathmaxx=\dimen280
\pgf@pathminy=\dimen281
\pgf@pathmaxy=\dimen282
\pgf@xx=\dimen283
\pgf@xy=\dimen284
\pgf@yx=\dimen285
\pgf@yy=\dimen286
\pgf@zx=\dimen287
\pgf@zy=\dimen288
)
(c:/texlive/2024/texmf-
dist/tex/generic/pgf/basiclayer/pgfcorepathconstruct.cod
e.tex
File: pgfcorepathconstruct.code.tex 2023-01-15 v3.1.10 (3.1.10)
\pgf@path@lastx=\dimen289
\pgf@path@lasty=\dimen290
)
(c:/texlive/2024/texmf-
dist/tex/generic/pgf/basiclayer/pgfcorepathusage.code.te
x
File: pgfcorepathusage.code.tex 2023-01-15 v3.1.10 (3.1.10)
\pgf@shorten@end@additional=\dimen291
\pgf@shorten@start@additional=\dimen292
) (c:/texlive/2024/texmf-
dist/tex/generic/pgf/basiclayer/pgfcorescopes.code.tex
File: pgfcorescopes.code.tex 2023-01-15 v3.1.10 (3.1.10)
\pgfpic=\box78
\pgf@hbox=\box79
\pgf@layerbox@main=\box80
\pgf@picture@serial@count=\count331
)
(c:/texlive/2024/texmf-
dist/tex/generic/pgf/basiclayer/pgfcoregraphicstate.code
.tex
File: pgfcoregraphicstate.code.tex 2023-01-15 v3.1.10 (3.1.10)
\pgflinewidth=\dimen293
)
(c:/texlive/2024/texmf-
dist/tex/generic/pgf/basiclayer/pgfcoretransformations.c
ode.tex
File: pgfcoretransformations.code.tex 2023-01-15 v3.1.10 (3.1.10)
\pgf@pt@x=\dimen294
\pgf@pt@y=\dimen295
\pgf@pt@temp=\dimen296
```

```
) (c:/texlive/2024/texmf-
dist/tex/generic/pgf/basiclayer/pgfcorequick.code.tex
File: pgfcorequick.code.tex 2023-01-15 v3.1.10 (3.1.10)
) (c:/texlive/2024/texmf-
dist/tex/generic/pgf/basiclayer/pgfcoreobjects.code.te
x
File: pgfcoreobjects.code.tex 2023-01-15 v3.1.10 (3.1.10)
)
(c:/texlive/2024/texmf-
dist/tex/generic/pgf/basiclayer/pgfcorepathprocessing.co
de.tex
File: pgfcorepathprocessing.code.tex 2023-01-15 v3.1.10 (3.1.10)
) (c:/texlive/2024/texmf-
dist/tex/generic/pgf/basiclayer/pgfcorearrows.code.tex
File: pgfcorearrows.code.tex 2023-01-15 v3.1.10 (3.1.10)
\pgfarrowsep=\dimen297
) (c:/texlive/2024/texmf-
dist/tex/generic/pgf/basiclayer/pgfcoreshade.code.tex
File: pgfcoreshade.code.tex 2023-01-15 v3.1.10 (3.1.10)
\pgf@max=\dimen298
\pgf@sys@shading@range@num=\count332
\pgf@shadingcount=\count333
) (c:/texlive/2024/texmf-
dist/tex/generic/pgf/basiclayer/pgfcoreimage.code.tex
File: pgfcoreimage.code.tex 2023-01-15 v3.1.10 (3.1.10)
)
(c:/texlive/2024/texmf-
dist/tex/generic/pgf/basiclayer/pgfcoreexternal.code.tex
File: pgfcoreexternal.code.tex 2023-01-15 v3.1.10 (3.1.10)
\pgfexternal@startupbox=\box81
) (c:/texlive/2024/texmf-
dist/tex/generic/pgf/basiclayer/pgfcorelayers.code.tex
File: pgfcorelayers.code.tex 2023-01-15 v3.1.10 (3.1.10)
)
(c:/texlive/2024/texmf-
dist/tex/generic/pgf/basiclayer/pgfcoretransparency.code
.tex
File: pgfcoretransparency.code.tex 2023-01-15 v3.1.10 (3.1.10)
)
(c:/texlive/2024/texmf-
dist/tex/generic/pgf/basiclayer/pgfcorepatterns.code.tex
File: pgfcorepatterns.code.tex 2023-01-15 v3.1.10 (3.1.10)
) (c:/texlive/2024/texmf-
dist/tex/generic/pgf/basiclayer/pgfcorerdf.code.tex
File: pgfcorerdf.code.tex 2023-01-15 v3.1.10 (3.1.10)
))) (c:/texlive/2024/texmf-
dist/tex/generic/pgf/modules/pgfmoduleshapes.code.te
x
File: pgfmoduleshapes.code.tex 2023-01-15 v3.1.10 (3.1.10)
\pgfnodeparttextbox=\box82
) (c:/texlive/2024/texmf-
dist/tex/generic/pgf/modules/pgfmoduleplot.code.tex
File: pgfmoduleplot.code.tex 2023-01-15 v3.1.10 (3.1.10)
)
```

```
(c:/texlive/2024/texmf-dist/tex/latex/pgf/compatibility/pgfcomp-version-
0-65.st
y
Package: pgfcomp-version-0-65 2023-01-15 v3.1.10 (3.1.10)
\pgf@nodesepstart=\dimen299
\pgf@nodesepend=\dimen300
)
(c:/texlive/2024/texmf-dist/tex/latex/pgf/compatibility/pgfcomp-version-
1-18.st
y
Package: pgfcomp-version-1-18 2023-01-15 v3.1.10 (3.1.10)
)) (c:/texlive/2024/texmf-dist/tex/latex/pgf/utilities/pgffor.sty
(c:/texlive/2
024/texmf-dist/tex/latex/pgf/utilities/pgfkeys.sty
(c:/texlive/2024/texmf-dist/
tex/generic/pgf/utilities/pgfkeys.code.tex)) (c:/texlive/2024/texmf-
dist/tex/la
tex/pgf/math/pgfmath.sty (c:/texlive/2024/texmf-
dist/tex/generic/pgf/math/pgfma
th.code.tex)) (c:/texlive/2024/texmf-
dist/tex/generic/pgf/utilities/pgffor.code
.tex
Package: pgffor 2023-01-15 v3.1.10 (3.1.10)
\pgffor@iter=\dimen301
\pgffor@skip=\dimen302
\pgffor@stack=\toks50
\pgffor@toks=\toks51
)) (c:/texlive/2024/texmf-
dist/tex/generic/pgf/frontendlayer/tikz/tikz.code.tex
Package: tikz 2023-01-15 v3.1.10 (3.1.10)

(c:/texlive/2024/texmf-
dist/tex/generic/pgf/libraries/pgflibraryplothandlers.co
de.tex
File: pgflibraryplothandlers.code.tex 2023-01-15 v3.1.10 (3.1.10)
\pgf@plot@mark@count=\count334
\pgfplotmarksize=\dimen303
)
\tikz@lastx=\dimen304
\tikz@lasty=\dimen305
\tikz@lastxsaved=\dimen306
\tikz@lastysaved=\dimen307
\tikz@lastmovetox=\dimen308
\tikz@lastmovetoy=\dimen309
\tikzleveldistance=\dimen310
\tikzsiblingdistance=\dimen311
\tikz@figbox=\box83
\tikz@figbox@bg=\box84
\tikz@tempbox=\box85
\tikz@tempbox@bg=\box86
\tikztreelevel=\count335
\tikznumberofchildren=\count336
\tikznumberofcurrentchild=\count337
\tikz@fig@count=\count338
```

```
(c:/texlive/2024/texmf-
dist/tex/generic/pgf/modules/pgfmodulematrix.code.tex
File: pgfmodulematrix.code.tex 2023-01-15 v3.1.10 (3.1.10)
\pgfmatrixcurrentrow=\count339
\pgfmatrixcurrentcolumn=\count340
\pgf@matrix@numberofcolumns=\count341
)
\tikz@expandcount=\count342

(c:/texlive/2024/texmf-
dist/tex/generic/pgf/frontendlayer/tikz/libraries/tikzli
brarytopaths.code.tex
File: tikzlibrarytopaths.code.tex 2023-01-15 v3.1.10 (3.1.10)
))) (c:/texlive/2024/texmf-dist/tex/latex/placeins/placeins.sty
Package: placeins 2005/04/18  v 2.2
) (c:/texlive/2024/texmf-dist/tex/latex/float/float.sty
Package: float 2001/11/08 v1.3d Float enhancements (AL)
\c@float@type=\count343
\float@exts=\toks52
\float@box=\box87
\@float@everytoks=\toks53
\@floatcapt=\box88
) (c:/texlive/2024/texmf-dist/tex/latex/tools/multicol.sty
Package: multicol 2024/05/23 v1.9h multicolumn formatting (FMi)
\c@tracingmulticols=\count344
\mult@box=\box89
\multicol@leftmargin=\dimen312
\c@unbalance=\count345
\c@collectmore=\count346
\doublecol@number=\count347
\multicoltolerance=\count348
\multicolpretolerance=\count349
\full@width=\dimen313
\page@free=\dimen314
\premulticols=\dimen315
\postmulticols=\dimen316
\multicolsep=\skip163
\multicolbaselineskip=\skip164
\partial@page=\box90
\last@line=\box91
\mc@boxedresult=\box92
\maxbalancingoverflow=\dimen317
\mult@rightbox=\box93
\mult@grightbox=\box94
\mult@firstbox=\box95
\mult@gfirstbox=\box96
\@tempa=\box97
\@tempa=\box98
\@tempa=\box99
\@tempa=\box100
\@tempa=\box101
\@tempa=\box102
\@tempa=\box103
\@tempa=\box104
```

```
\@tempa=\box105
\@tempa=\box106
\@tempa=\box107
\@tempa=\box108
\@tempa=\box109
\@tempa=\box110
\@tempa=\box111
\@tempa=\box112
\@tempa=\box113
\@tempa=\box114
\@tempa=\box115
\@tempa=\box116
\@tempa=\box117
\@tempa=\box118
\@tempa=\box119
\@tempa=\box120
\@tempa=\box121
\@tempa=\box122
\@tempa=\box123
\@tempa=\box124
\@tempa=\box125
\@tempa=\box126
\@tempa=\box127
\@tempa=\box128
\@tempa=\box129
\@tempa=\box130
\@tempa=\box131
\@tempa=\box132
\c@minrows=\count350
\c@columnbadness=\count351
\c@finalcolumnbadness=\count352
\last@try=\dimen318
\multicolovershoot=\dimen319
\multicolundershoot=\dimen320
\mult@nat@firstbox=\box133
\colbreak@box=\box134
\mc@col@check@num=\count353
) (c:/texlive/2024/texmf-dist/tex/latex/tcolorbox/tcolorbox.sty
Package: tcolorbox 2024/07/10 version 6.3.0 text color boxes
(c:/texlive/2024/texmf-dist/tex/latex/tools/verbatim.sty
Package: verbatim 2024-01-22 v1.5x LaTeX2e package for verbatim
enhancements
\every@verbatim=\toks54
\verbatim@line=\toks55
\verbatim@in@stream=\read3
)
\tcb@titlebox=\box135
\tcb@upperbox=\box136
\tcb@lowerbox=\box137
\tcb@phantombox=\box138
\c@tcbbreakpart=\count354
\c@tcblayer=\count355
\c@tcolorbox@number=\count356
\l__tcobox_tmpa_box=\box139
```

```
\l__tcobox_tmpa_dim=\dimen321
\tcb@temp=\box140
\tcb@temp=\box141
\tcb@temp=\box142
\tcb@temp=\box143
) (c:/texlive/2024/texmf-dist/tex/latex/hyperref/hyperref.sty
Package: hyperref 2024-07-10 v7.01j Hypertext links for LaTeX
(c:/texlive/2024/texmf-dist/tex/generic/pdfescape/pdfescape.sty
Package: pdfescape 2019/12/09 v1.15 Implements pdfTeX's escape features
(HO)
) (c:/texlive/2024/texmf-dist/tex/latex/hycolor/hycolor.sty
Package: hycolor 2020-01-27 v1.10 Color options for hyperref/bookmark
(HO)
) (c:/texlive/2024/texmf-dist/tex/latex/hyperref/nameref.sty
Package: nameref 2023-11-26 v2.56 Cross-referencing by name of section
(c:/texlive/2024/texmf-dist/tex/latex/refcount/refcount.sty
Package: refcount 2019/12/15 v3.6 Data extraction from label references
(HO)
) (c:/texlive/2024/texmf-
dist/tex/generic/gettitlestring/gettitlestring.sty
Package: gettitlestring 2019/12/15 v1.6 Cleanup title references (HO)
)
\c@section@level=\count357
) (c:/texlive/2024/texmf-dist/tex/generic/stringenc/stringenc.sty
Package: stringenc 2019/11/29 v1.12 Convert strings between diff.
encodings (HO
)
)
\@linkdim=\dimen322
\Hy@linkcounter=\count358
\Hy@pagecounter=\count359
(c:/texlive/2024/texmf-dist/tex/latex/hyperref/pd1enc.def
File: pd1enc.def 2024-07-10 v7.01j Hyperref: PDFDocEncoding definition
(HO)
Now handling font encoding PD1 ...
... no UTF-8 mapping file for font encoding PD1
)
\Hy@SavedSpaceFactor=\count360
(c:/texlive/2024/texmf-dist/tex/latex/hyperref/puenc.def
File: puenc.def 2024-07-10 v7.01j Hyperref: PDF Unicode definition (HO)
Now handling font encoding PU ...
... no UTF-8 mapping file for font encoding PU
)
Package hyperref Info: Option `colorlinks' set `true' on input line 4040.
Package hyperref Info: Hyper figures OFF on input line 4157.
Package hyperref Info: Link nesting OFF on input line 4162.
Package hyperref Info: Hyper index ON on input line 4165.
Package hyperref Info: Plain pages OFF on input line 4172.
Package hyperref Info: Backreferencing OFF on input line 4177.
Package hyperref Info: Implicit mode ON; LaTeX internals redefined.
Package hyperref Info: Bookmarks ON on input line 4424.
\c@Hy@tempcnt=\count361
LaTeX Info: Redefining \url on input line 4763.
\XeTeXLinkMargin=\dimen323
```

```
(c:/texlive/2024/texmf-dist/tex/generic/bitset/bitset.sty
Package: bitset 2019/12/09 v1.3 Handle bit-vector datatype (HO)
(c:/texlive/2024/texmf-dist/tex/generic/bigintcalc/bigintcalc.sty
Package: bigintcalc 2019/12/15 v1.5 Expandable calculations on big
integers (HO
)
))
\Fld@menulength=\count362
\Field@Width=\dimen324
\Fld@charsize=\dimen325
Package hyperref Info: Hyper figures OFF on input line 6042.
Package hyperref Info: Link nesting OFF on input line 6047.
Package hyperref Info: Hyper index ON on input line 6050.
Package hyperref Info: backreferencing OFF on input line 6057.
Package hyperref Info: Link coloring ON on input line 6060.
Package hyperref Info: Link coloring with OCG OFF on input line 6067.
Package hyperref Info: PDF/A mode OFF on input line 6072.
(c:/texlive/2024/texmf-dist/tex/latex/base/atbegshi-ltx.sty
Package: atbegshi-ltx 2021/01/10 v1.0c Emulation of the original atbegshi
package with kernel methods
)
\Hy@abspage=\count363
\c@Item=\count364
\c@Hfootnote=\count365
)
Package hyperref Info: Driver (autodetected): hpdftex.
(c:/texlive/2024/texmf-dist/tex/latex/hyperref/hpdftex.def
File: hpdftex.def 2024-07-10 v7.01j Hyperref driver for pdfTeX
(c:/texlive/2024/texmf-dist/tex/latex/base/atveryend-ltx.sty
Package: atveryend-ltx 2020/08/19 v1.0a Emulation of the original
atveryend pac
kage
with kernel methods
)
\HyAnn@Count=\count366
\Fld@listcount=\count367
\c@bookmark@seq@number=\count368
(c:/texlive/2024/texmf-dist/tex/latex/rerunfilecheck/rerunfilecheck.sty
Package: rerunfilecheck 2022-07-10 v1.10 Rerun checks for auxiliary files
(HO)
(c:/texlive/2024/texmf-dist/tex/generic/uniquecounter/uniquecounter.sty
Package: uniquecounter 2019/12/15 v1.4 Provide unlimited unique counter
(HO)
)
Package uniquecounter Info: New unique counter `rerunfilecheck' on input
line 2
85.
)
\Hy@SectionHShift=\skip165
)
Package translations Info: No language package found. I am going to use
`englis
h' as default language. on input line 59.
```

```
LaTeX Font Info:    Trying to load font information for T1+Merriwthr-OsF
on inp
ut line 59.
(c:/texlive/2024/texmf-dist/tex/latex/merriweather/T1Merriwthr-OsF.fd
File: T1Merriwthr-OsF.fd 2020/08/30 (autoinst) Font definitions for
T1/Merriwth
r-OsF.
)
LaTeX Font Info:    Font shape `T1/Merriwthr-OsF/m/n' will be
(Font)              scaled to size 7.5pt on input line 59.
(./main.aux

LaTeX Warning: Label `sec2' multiply defined.

)
\openout1 = `main.aux'.

LaTeX Font Info:    Checking defaults for OML/cmm/m/it on input line 59.
LaTeX Font Info:    ... okay on input line 59.
LaTeX Font Info:    Checking defaults for OMS/cmsy/m/n on input line 59.
LaTeX Font Info:    ... okay on input line 59.
LaTeX Font Info:    Checking defaults for OT1/cmr/m/n on input line 59.
LaTeX Font Info:    ... okay on input line 59.
LaTeX Font Info:    Checking defaults for T1/cmr/m/n on input line 59.
LaTeX Font Info:    ... okay on input line 59.
LaTeX Font Info:    Checking defaults for TS1/cmr/m/n on input line 59.
LaTeX Font Info:    ... okay on input line 59.
LaTeX Font Info:    Checking defaults for OMX/cmex/m/n on input line 59.
LaTeX Font Info:    ... okay on input line 59.
LaTeX Font Info:    Checking defaults for U/cmr/m/n on input line 59.
LaTeX Font Info:    ... okay on input line 59.
LaTeX Font Info:    Checking defaults for PD1/pdf/m/n on input line 59.
LaTeX Font Info:    ... okay on input line 59.
LaTeX Font Info:    Checking defaults for PU/pdf/m/n on input line 59.
LaTeX Font Info:    ... okay on input line 59.
LaTeX Info: Redefining \microtypecontext on input line 59.
Package microtype Info: Applying patch `item' on input line 59.
Package microtype Info: Applying patch `toc' on input line 59.
Package microtype Info: Applying patch `eqnum' on input line 59.
Package microtype Info: Applying patch `footnote' on input line 59.
Package microtype Info: Applying patch `verbatim' on input line 59.
Package microtype Info: Generating PDF output.
Package microtype Info: Character protrusion enabled (level 2).
Package microtype Info: Using default protrusion set `alltext'.
Package microtype Info: Automatic font expansion enabled (level 2),
(microtype)             stretch: 20, shrink: 20, step: 1, non-selected.
Package microtype Info: Using default expansion set `alltext-nott'.
LaTeX Info: Redefining \showhyphens on input line 59.
Package microtype Info: No adjustment of tracking.
Package microtype Info: No adjustment of interword spacing.
Package microtype Info: No adjustment of character kerning.
Package microtype Info: Loading generic protrusion settings for font
family
(microtype)             `Merriwthr-OsF' (encoding: T1).
```

```
(microtype)             For optimal results, create family-specific
settings.
(microtype)             See the microtype manual for details.
LaTeX Font Info:    Redeclaring symbol font `operators' on input line 59.
LaTeX Font Info:    Encoding `OT1' has changed to `T1' for symbol font
(Font)              `operators' in the math version `normal' on input
line 59.
LaTeX Font Info:    Overwriting symbol font `operators' in version
`normal'
(Font)                  OT1/cmr/m/n --> T1/Merriwthr-OsF/m/up on input
line 59.

LaTeX Font Info:    Encoding `OT1' has changed to `T1' for symbol font
(Font)              `operators' in the math version `bold' on input line
59.
LaTeX Font Info:    Overwriting symbol font `operators' in version `bold'
(Font)                  OT1/cmr/bx/n --> T1/Merriwthr-OsF/m/up on input
line 59
.
LaTeX Font Info:    Overwriting symbol font `operators' in version `bold'
(Font)                  T1/Merriwthr-OsF/m/up --> T1/Merriwthr-OsF/b/up
on inpu
t line 59.
LaTeX Font Info:    Redeclaring math alphabet \mathbf on input line 59.
LaTeX Font Info:    Overwriting math alphabet `\mathbf' in version
`normal'
(Font)                  OT1/cmr/bx/n --> T1/Merriwthr-OsF/b/up on input
line 59
.
LaTeX Font Info:    Overwriting math alphabet `\mathbf' in version `bold'
(Font)                  OT1/cmr/bx/n --> T1/Merriwthr-OsF/b/up on input
line 59
.
LaTeX Font Info:    Redeclaring math alphabet \mathsf on input line 59.
LaTeX Font Info:    Overwriting math alphabet `\mathsf' in version
`normal'
(Font)                  OT1/cmss/m/n --> T1/MerriwthrSans-OsF/m/up on
input lin
e 59.
LaTeX Font Info:    Overwriting math alphabet `\mathsf' in version `bold'
(Font)                  OT1/cmss/bx/n --> T1/MerriwthrSans-OsF/m/up on
input li
ne 59.
LaTeX Font Info:    Redeclaring math alphabet \mathit on input line 59.
LaTeX Font Info:    Overwriting math alphabet `\mathit' in version
`normal'
(Font)                  OT1/cmr/m/it --> T1/Merriwthr-OsF/m/it on input
line 59
.
LaTeX Font Info:    Overwriting math alphabet `\mathit' in version `bold'
(Font)                  OT1/cmr/bx/it --> T1/Merriwthr-OsF/m/it on input
line 5
9.
LaTeX Font Info:    Redeclaring math alphabet \mathtt on input line 59.
```

```
LaTeX Font Info:    Overwriting math alphabet `\mathtt' in version
`normal'
(Font)                  OT1/cmtt/m/n --> T1/lmtt/m/up on input line 59.
LaTeX Font Info:    Overwriting math alphabet `\mathtt' in version `bold'
(Font)                  OT1/cmtt/m/n --> T1/lmtt/m/up on input line 59.
LaTeX Font Info:    Overwriting math alphabet `\mathsf' in version `bold'
(Font)                  T1/MerriwthrSans-OsF/m/up --> T1/MerriwthrSans-
OsF/b/up
 on input line 59.
LaTeX Font Info:    Overwriting math alphabet `\mathit' in version `bold'
(Font)                  T1/Merriwthr-OsF/m/it --> T1/Merriwthr-OsF/b/it
on inpu
t line 59.
\c@mv@tabular=\count369
\c@mv@boldtabular=\count370
(c:/texlive/2024/texmf-dist/tex/context/base/mkii/supp-pdf.mkii
[Loading MPS to PDF converter (version 2006.09.02).]
\scratchcounter=\count371
\scratchdimen=\dimen326
\scratchbox=\box144
\nofMPsegments=\count372
\nofMParguments=\count373
\everyMPshowfont=\toks56
\MPscratchCnt=\count374
\MPscratchDim=\dimen327
\MPnumerator=\count375
\makeMPintoPDFobject=\count376
\everyMPtoPDFconversion=\toks57
) (c:/texlive/2024/texmf-dist/tex/latex/epstopdf-pkg/epstopdf-base.sty
Package: epstopdf-base 2020-01-24 v2.11 Base part for package epstopdf
Package epstopdf-base Info: Redefining graphics rule for `.eps' on input
line 4
85.
(c:/texlive/2024/texmf-dist/tex/latex/latexconfig/epstopdf-sys.cfg
File: epstopdf-sys.cfg 2010/07/13 v1.3 Configuration of (r)epstopdf for
TeX Liv
e
))
Package newfloat Info: `float' package detected.
*geometry* driver: auto-detecting
*geometry* detected driver: pdftex
*geometry* verbose mode - [ preamble ] result:
* driver: pdftex
* paper: a4paper
* layout: <same size as paper>
* layoutoffset:(h,v)=(0.0pt,0.0pt)
* modes: includefoot twoside
* h-part:(L,W,R)=(54.64pt, 488.22787pt, 54.64pt)
* v-part:(T,H,B)=(66.0pt, 745.04684pt, 34.0pt)
* \paperwidth=597.50787pt
* \paperheight=845.04684pt
* \textwidth=488.22787pt
* \textheight=715.04684pt
* \oddsidemargin=-17.62999pt
```

```
* \evensidemargin=-17.62999pt
* \topmargin=-47.76999pt
* \headheight=17.5pt
* \headsep=24.0pt
* \topskip=10.0pt
* \footskip=30.0pt
* \marginparwidth=48.0pt
* \marginparsep=10.0pt
* \columnsep=18.0pt
* \skip\footins=22.0pt plus 2.0pt
* \hoffset=0.0pt
* \voffset=0.0pt
* \mag=1000
* \@twocolumntrue
* \@twosidetrue
* \@mparswitchtrue
* \@reversemarginfalse
* (1in=72.27pt=25.4mm, 1cm=28.453pt)

Package caption Info: Begin \AtBeginDocument code.
Package caption Info: float package is loaded.
Package caption Info: hyperref package is loaded.
Package caption Info: End \AtBeginDocument code.

(c:/texlive/2024/texmf-dist/tex/latex/translations/translations-basic-
dictionar
y-english.trsl
File: translations-basic-dictionary-english.trsl (english translation
file `tra
nslations-basic-dictionary')
)
Package translations Info: loading dictionary `translations-basic-
dictionary' f
or `english'. on input line 59.
Package hyperref Info: Link coloring ON on input line 59.
(./main.out) (./main.out)
\@outlinefile=\write4
\openout4 = `main.out'.

\@gscitedetails=\box145
\@gscitedetailsheight=\skip166
\@gsheadbox=\box146
\@gsheadboxheight=\skip167
LaTeX Font Info:    Font shape `T1/Merriwthr-OsF/b/n' will be
(Font)              scaled to size 6.5pt on input line 59.
LaTeX Font Info:    Calculating math sizes for size <7.5> on input line
59.

LaTeX Font Warning: Font shape `T1/Merriwthr-OsF/m/up' undefined
(Font)              using `T1/Merriwthr-OsF/m/n' instead on input line
59.

LaTeX Font Info:    Font shape `T1/Merriwthr-OsF/m/up' will be
(Font)              scaled to size 6.24973pt on input line 59.
```

```
LaTeX Font Info:    Font shape `T1/Merriwthr-OsF/m/up' will be
(Font)              scaled to size 5.24997pt on input line 59.
LaTeX Font Info:    Trying to load font information for U+eur on input
line 59.

(c:/texlive/2024/texmf-dist/tex/latex/amsfonts/ueur.fd
File: ueur.fd 2013/01/14 v3.01 Euler Roman
) (c:/texlive/2024/texmf-dist/tex/latex/microtype/mt-eur.cfg
File: mt-eur.cfg 2006/07/31 v1.1 microtype config. file: AMS Euler Roman
(RS)
)

LaTeX Font Warning: Font shape `OMS/cmsy/m/n' in size <7.5> not available
(Font)              size <7> substituted on input line 59.

LaTeX Font Info:    External font `cmex10' loaded for size
(Font)              <7.5> on input line 59.
LaTeX Font Info:    External font `cmex10' loaded for size
(Font)              <6.24973> on input line 59.
LaTeX Font Info:    External font `cmex10' loaded for size
(Font)              <5.24997> on input line 59.
LaTeX Font Info:    Trying to load font information for U+euf on input
line 59.

(c:/texlive/2024/texmf-dist/tex/latex/amsfonts/ueuf.fd
File: ueuf.fd 2013/01/14 v3.01 Euler Fraktur
) (c:/texlive/2024/texmf-dist/tex/latex/microtype/mt-euf.cfg
File: mt-euf.cfg 2006/07/03 v1.1 microtype config. file: AMS Euler
Fraktur (RS)

)
LaTeX Font Info:    Trying to load font information for U+eus on input
line 59.

(c:/texlive/2024/texmf-dist/tex/latex/amsfonts/ueus.fd
File: ueus.fd 2013/01/14 v3.01 Euler Script
) (c:/texlive/2024/texmf-dist/tex/latex/microtype/mt-eus.cfg
File: mt-eus.cfg 2006/07/28 v1.2 microtype config. file: AMS Euler Script
(RS)
)
LaTeX Font Info:    Trying to load font information for U+euex on input
line 59
.
(c:/texlive/2024/texmf-dist/tex/latex/amsfonts/ueuex.fd
File: ueuex.fd 2013/01/14 v3.01 Euler extra symbols
)

LaTeX Font Warning: Font shape `OML/cmm/m/it' in size <7.5> not available
(Font)              size <7> substituted on input line 59.

LaTeX Font Info:    Font shape `T1/Merriwthr-OsF/m/n' will be
(Font)              scaled to size 6.24973pt on input line 59.
LaTeX Font Info:    Font shape `T1/Merriwthr-OsF/m/n' will be
(Font)              scaled to size 5.24997pt on input line 59.
```

```
LaTeX Font Info:    Font shape `T1/Merriwthr-OsF/m/it' will be
(Font)              scaled to size 7.5pt on input line 59.
LaTeX Font Info:    Font shape `T1/Merriwthr-OsF/m/it' will be
(Font)              scaled to size 6.24973pt on input line 59.
LaTeX Font Info:    Font shape `T1/Merriwthr-OsF/m/it' will be
(Font)              scaled to size 5.24997pt on input line 59.
LaTeX Font Info:    Font shape `T1/Merriwthr-OsF/m/n' will be
(Font)              scaled to size 8.0pt on input line 59.
LaTeX Font Info:    Font shape `T1/Merriwthr-OsF/m/it' will be
(Font)              scaled to size 8.0pt on input line 59.
LaTeX Font Info:    Font shape `T1/Merriwthr-OsF/b/it' will be
(Font)              scaled to size 8.0pt on input line 59.
TextBlockOrigin set to 4pc+6.64pt x 4pc+6pt

Overfull \hbox (54.64pt too wide) in paragraph at lines 85--85
[][]
 []

LaTeX Font Info:    Font shape `T1/Merriwthr-OsF/m/n' will be
(Font)              scaled to size 14.0pt on input line 85.
LaTeX Font Info:    Font shape `T1/Merriwthr-OsF/m/n' will be
(Font)              scaled to size 8.99997pt on input line 85.
LaTeX Font Info:    Calculating math sizes for size <14> on input line
85.
LaTeX Font Info:    Font shape `T1/Merriwthr-OsF/m/up' will be
(Font)              scaled to size 14.0pt on input line 85.
LaTeX Font Info:    Font shape `T1/Merriwthr-OsF/m/up' will be
(Font)              scaled to size 11.66617pt on input line 85.
LaTeX Font Info:    Font shape `T1/Merriwthr-OsF/m/up' will be
(Font)              scaled to size 9.79996pt on input line 85.
LaTeX Font Info:    External font `cmex10' loaded for size
(Font)              <14> on input line 85.
LaTeX Font Info:    External font `cmex10' loaded for size
(Font)              <11.66617> on input line 85.
LaTeX Font Info:    External font `cmex10' loaded for size
(Font)              <9.79996> on input line 85.
LaTeX Font Info:    Font shape `T1/Merriwthr-OsF/m/n' will be
(Font)              scaled to size 11.66617pt on input line 85.
LaTeX Font Info:    Font shape `T1/Merriwthr-OsF/m/n' will be
(Font)              scaled to size 9.79996pt on input line 85.
LaTeX Font Info:    Font shape `T1/Merriwthr-OsF/m/it' will be
(Font)              scaled to size 14.0pt on input line 85.
LaTeX Font Info:    Font shape `T1/Merriwthr-OsF/m/it' will be
(Font)              scaled to size 11.66617pt on input line 85.
LaTeX Font Info:    Font shape `T1/Merriwthr-OsF/m/it' will be
(Font)              scaled to size 9.79996pt on input line 85.
LaTeX Font Info:    Font shape `T1/Merriwthr-OsF/b/n' will be
(Font)              scaled to size 18.0pt on input line 85.
LaTeX Font Info:    Font shape `T1/Merriwthr-OsF/m/n' will be
(Font)              scaled to size 13.0pt on input line 85.
LaTeX Font Info:    Calculating math sizes for size <13> on input line
85.
LaTeX Font Info:    Font shape `T1/Merriwthr-OsF/m/up' will be
(Font)              scaled to size 13.0pt on input line 85.
```

```
LaTeX Font Info:    Font shape `T1/Merriwthr-OsF/m/up' will be
(Font)              scaled to size 10.83287pt on input line 85.
LaTeX Font Info:    Font shape `T1/Merriwthr-OsF/m/up' will be
(Font)              scaled to size 9.09996pt on input line 85.

LaTeX Font Warning: Font shape `OMS/cmsy/m/n' in size <13> not available
(Font)              size <12> substituted on input line 85.

LaTeX Font Info:    External font `cmex10' loaded for size
(Font)              <13> on input line 85.
LaTeX Font Info:    External font `cmex10' loaded for size
(Font)              <10.83287> on input line 85.
LaTeX Font Info:    External font `cmex10' loaded for size
(Font)              <9.09996> on input line 85.

LaTeX Font Warning: Font shape `OML/cmm/m/it' in size <13> not available
(Font)              size <12> substituted on input line 85.

LaTeX Font Info:    Font shape `T1/Merriwthr-OsF/m/n' will be
(Font)              scaled to size 10.83287pt on input line 85.
LaTeX Font Info:    Font shape `T1/Merriwthr-OsF/m/n' will be
(Font)              scaled to size 9.09996pt on input line 85.
LaTeX Font Info:    Font shape `T1/Merriwthr-OsF/m/it' will be
(Font)              scaled to size 13.0pt on input line 85.
LaTeX Font Info:    Font shape `T1/Merriwthr-OsF/m/it' will be
(Font)              scaled to size 10.83287pt on input line 85.
LaTeX Font Info:    Font shape `T1/Merriwthr-OsF/m/it' will be
(Font)              scaled to size 9.09996pt on input line 85.
LaTeX Font Info:    Calculating math sizes for size <10.83287> on input
line 85
.
LaTeX Font Info:    Font shape `T1/Merriwthr-OsF/m/up' will be
(Font)              scaled to size 9.027pt on input line 85.
LaTeX Font Info:    Font shape `T1/Merriwthr-OsF/m/up' will be
(Font)              scaled to size 7.58296pt on input line 85.

LaTeX Font Warning: Font shape `OMS/cmsy/m/n' in size <7.58296> not
available
(Font)              size <8> substituted on input line 85.

LaTeX Font Info:    External font `cmex10' loaded for size
(Font)              <9.027> on input line 85.
LaTeX Font Info:    External font `cmex10' loaded for size
(Font)              <7.58296> on input line 85.

LaTeX Font Warning: Font shape `OML/cmm/m/it' in size <7.58296> not
available
(Font)              size <8> substituted on input line 85.

LaTeX Font Info:    Font shape `T1/Merriwthr-OsF/m/n' will be
(Font)              scaled to size 9.027pt on input line 85.
LaTeX Font Info:    Font shape `T1/Merriwthr-OsF/m/n' will be
(Font)              scaled to size 7.58296pt on input line 85.
LaTeX Font Info:    Font shape `T1/Merriwthr-OsF/m/it' will be
```

```
(Font)              scaled to size 9.027pt on input line 85.
LaTeX Font Info:    Font shape `T1/Merriwthr-OsF/m/it' will be
(Font)              scaled to size 7.58296pt on input line 85.
LaTeX Font Info:    Font shape `T1/Merriwthr-OsF/m/n' will be
(Font)              scaled to size 9.0pt on input line 85.
LaTeX Font Info:    Font shape `T1/Merriwthr-OsF/m/up' will be
(Font)              scaled to size 9.0pt on input line 85.
LaTeX Font Info:    Font shape `T1/Merriwthr-OsF/m/up' will be
(Font)              scaled to size 7.0pt on input line 85.
LaTeX Font Info:    Font shape `T1/Merriwthr-OsF/m/up' will be
(Font)              scaled to size 5.0pt on input line 85.
LaTeX Font Info:    External font `cmex10' loaded for size
(Font)              <9> on input line 85.
LaTeX Font Info:    External font `cmex10' loaded for size
(Font)              <7> on input line 85.
LaTeX Font Info:    External font `cmex10' loaded for size
(Font)              <5> on input line 85.
LaTeX Font Info:    Font shape `T1/Merriwthr-OsF/m/n' will be
(Font)              scaled to size 7.0pt on input line 85.
LaTeX Font Info:    Font shape `T1/Merriwthr-OsF/m/n' will be
(Font)              scaled to size 5.0pt on input line 85.
LaTeX Font Info:    Font shape `T1/Merriwthr-OsF/m/it' will be
(Font)              scaled to size 9.0pt on input line 85.
LaTeX Font Info:    Font shape `T1/Merriwthr-OsF/m/it' will be
(Font)              scaled to size 7.0pt on input line 85.
LaTeX Font Info:    Font shape `T1/Merriwthr-OsF/m/it' will be
(Font)              scaled to size 5.0pt on input line 85.
LaTeX Font Info:    Font shape `T1/Merriwthr-OsF/m/n' will be
(Font)              scaled to size 6.5pt on input line 85.
LaTeX Font Info:    Calculating math sizes for size <6.5> on input line
85.
LaTeX Font Info:    Font shape `T1/Merriwthr-OsF/m/up' will be
(Font)              scaled to size 6.5pt on input line 85.
LaTeX Font Info:    Font shape `T1/Merriwthr-OsF/m/up' will be
(Font)              scaled to size 5.41643pt on input line 85.
LaTeX Font Info:    Font shape `T1/Merriwthr-OsF/m/up' will be
(Font)              scaled to size 4.54997pt on input line 85.

LaTeX Font Warning: Font shape `OMS/cmsy/m/n' in size <6.5> not available
(Font)              size <6> substituted on input line 85.


LaTeX Font Warning: Font shape `OMS/cmsy/m/n' in size <5.41643> not
available
(Font)              size <5> substituted on input line 85.


LaTeX Font Warning: Font shape `OMS/cmsy/m/n' in size <4.54997> not
available
(Font)              size <5> substituted on input line 85.

LaTeX Font Info:    External font `cmex10' loaded for size
(Font)              <6.5> on input line 85.
LaTeX Font Info:    External font `cmex10' loaded for size
```

```
(Font)              <5.41643> on input line 85.
LaTeX Font Info:    External font `cmex10' loaded for size
(Font)              <4.54997> on input line 85.

LaTeX Font Warning: Font shape `OML/cmm/m/it' in size <6.5> not available
(Font)              size <6> substituted on input line 85.


LaTeX Font Warning: Font shape `OML/cmm/m/it' in size <5.41643> not
available
(Font)              size <5> substituted on input line 85.


LaTeX Font Warning: Font shape `OML/cmm/m/it' in size <4.54997> not
available
(Font)              size <5> substituted on input line 85.

LaTeX Font Info:    Font shape `T1/Merriwthr-OsF/m/n' will be
(Font)              scaled to size 5.41643pt on input line 85.
LaTeX Font Info:    Font shape `T1/Merriwthr-OsF/m/n' will be
(Font)              scaled to size 4.54997pt on input line 85.
LaTeX Font Info:    Font shape `T1/Merriwthr-OsF/m/it' will be
(Font)              scaled to size 6.5pt on input line 85.
LaTeX Font Info:    Font shape `T1/Merriwthr-OsF/m/it' will be
(Font)              scaled to size 5.41643pt on input line 85.
LaTeX Font Info:    Font shape `T1/Merriwthr-OsF/m/it' will be
(Font)              scaled to size 4.54997pt on input line 85.
LaTeX Font Info:    Font shape `T1/Merriwthr-OsF/m/n' will be
(Font)              scaled to size 3.75pt on input line 85.

Overfull \hbox (54.64pt too wide) in paragraph at lines 85--85
[][][]
 []

LaTeX Font Info:    Font shape `T1/Merriwthr-OsF/b/n' will be
(Font)              scaled to size 10.0pt on input line 85.
LaTeX Font Info:    Font shape `T1/Merriwthr-OsF/b/n' will be
(Font)              scaled to size 8.0pt on input line 85.

Package natbib Warning: Citation `hofacker2004conserved' on page 1
undefined on
 input line 85.


Overfull \hbox (54.64pt too wide) in paragraph at lines 85--85
[][][]
 []

LaTeX Font Info:    Font shape `T1/Merriwthr-OsF/b/n' will be
(Font)              scaled to size 7.5pt on input line 90.

Package natbib Warning: Citation `hofacker2004conserved' on page 1
undefined on
 input line 90.
```

```
Package natbib Warning: Citation `harvey2021sars' on page 1 undefined on
input
line 90.



Package natbib Warning: Citation `jain2018nanopore' on page 1 undefined
on inpu
t line 92.


Package natbib Warning: Citation `eid2009real' on page 1 undefined on
input lin
e 93.


Package natbib Warning: Citation `wenger2019accurate' on page 1 undefined
on in
put line 93.


Underfull \hbox (badness 10000) in paragraph at lines 92--94
[]\T1/Merriwthr-OsF/m/n/7.5 (+20) Sequencing of vi-ral genomes pri-mar-
ily re-l
ies on Next-
 []


Underfull \hbox (badness 1609) in paragraph at lines 92--94
\T1/Merriwthr-OsF/m/n/7.5 (+20) NGS data. Third-generation se-quenc-ing
(TGS) t
ech-nolo-gies,
 []


Underfull \vbox (badness 10000) has occurred while \output is active []



LaTeX Font Info:    Font shape `T1/Merriwthr-OsF/m/n' will be
(Font)              scaled to size 7.8pt on input line 94.
LaTeX Font Info:    Font shape `T1/Merriwthr-OsF/b/n' will be
(Font)              scaled to size 7.8pt on input line 94.
[1{c:/texlive/2024/texmf-
var/fonts/map/pdftex/updmap/pdftex.map}{c:/texlive/202
4/texmf-dist/fonts/enc/dvips/merriweather/merriwthr_posqbl.enc}


]
```

Package natbib Warning: Citation `velvet' on page 2 undefined on input
line 95.

Package natbib Warning: Citation `abyss' on page 2 undefined on input
line 95.

Package natbib Warning: Citation `spades' on page 2 undefined on input
line 95.

Package natbib Warning: Citation `flye' on page 2 undefined on input line
95.

Package natbib Warning: Citation `canu' on page 2 undefined on input line
95.

Package natbib Warning: Citation `translig' on page 2 undefined on input
line 9
5.

Package natbib Warning: Citation `novoalign' on page 2 undefined on input
line
96.

Package natbib Warning: Citation `maq' on page 2 undefined on input line
96.

Package natbib Warning: Citation `ivar' on page 2 undefined on input line
96.

Package natbib Warning: Citation `accuvir' on page 2 undefined on input
line 96
.

Package natbib Warning: Citation `bcftools' on page 2 undefined on input
line 9
6.

Package natbib Warning: Citation `hunt2015iva' on page 2 undefined on
input lin
e 98.

```
Package natbib Warning: Citation `accuvir' on page 2 undefined on input
line 98
.


Package natbib Warning: Citation `pilon' on page 2 undefined on input
line 98.


Package natbib Warning: Citation `NextPolish' on page 2 undefined on
input line
 98.



LaTeX Font Info:    Font shape `T1/Merriwthr-OsF/b/n' will be
(Font)              scaled to size 8.5pt on input line 113.

Package natbib Warning: Citation `pbd' on page 2 undefined on input line
115.

LaTeX Font Info:    Font shape `T1/Merriwthr-OsF/m/up' will be
(Font)              scaled to size 7.5pt on input line 115.

LaTeX Warning: File `images/alignmentgraph.pdf' not found on input line
162.


! Package pdftex.def Error: File `images/alignmentgraph.pdf' not found:
using d
raft setting.

See the pdftex.def package documentation for explanation.
Type  H <return>  for immediate help.
 ...

l.162 }

Try typing  <return>  to proceed.
If that doesn't work, type  X <return>  to quit.

LaTeX Font Info:    Trying to load font information for T1+lmtt on input
line 1
62.
(c:/texlive/2024/texmf-dist/tex/latex/lm/t1lmtt.fd
File: t1lmtt.fd 2015/05/01 v1.6.1 Font defs for Latin Modern
)
Package microtype Info: Loading generic protrusion settings for font
family
(microtype)             `lmtt' (encoding: T1).
(microtype)             For optimal results, create family-specific
settings.
(microtype)             See the microtype manual for details.
```

```
LaTeX Warning: File `images/itergraph.pdf' not found on input line 162.


! Package pdftex.def Error: File `images/itergraph.pdf' not found: using
draft
setting.

See the pdftex.def package documentation for explanation.
Type  H <return>  for immediate help.
 ...

l.162 }

Try typing  <return>  to proceed.
If that doesn't work, type  X <return>  to quit.

LaTeX Font Info:    Font shape `T1/Merriwthr-OsF/m/n' will be
(Font)              scaled to size 6.25008pt on input line 162.
LaTeX Font Info:    Font shape `T1/Merriwthr-OsF/m/n' will be
(Font)              scaled to size 6.0pt on input line 168.
LaTeX Font Info:    Font shape `T1/Merriwthr-OsF/b/n' will be
(Font)              scaled to size 6.0pt on input line 168.
LaTeX Font Info:    Font shape `T1/Merriwthr-OsF/m/up' will be
(Font)              scaled to size 6.0pt on input line 168.
LaTeX Font Info:    External font `cmex10' loaded for size
(Font)              <6> on input line 168.
LaTeX Font Info:    Font shape `T1/Merriwthr-OsF/m/it' will be
(Font)              scaled to size 6.0pt on input line 168.

LaTeX Warning: `!h' float specifier changed to `!ht'.



LaTeX Font Info:    Font shape `T1/Merriwthr-OsF/m/it' will be
(Font)              scaled to size 7.8pt on input line 173.
[2]

Package natbib Warning: Citation `wick2019badread' on page 3 undefined on
input
 line 195.

LaTeX Font Info:    Font shape `T1/Merriwthr-OsF/b/sl' in size <7.5> not
availa
ble
(Font)              Font shape `T1/Merriwthr-OsF/b/it' tried instead on
input l
ine 198.
LaTeX Font Info:    Font shape `T1/Merriwthr-OsF/b/it' will be
(Font)              scaled to size 7.5pt on input line 198.
```

```
Package natbib Warning: Citation `swh_benchmark' on page 3 undefined on
input l
ine 201.


Package natbib Warning: Citation `li2018minimap2' on page 3 undefined on
input
line 201.


Package natbib Warning: Citation `danecek2021twelve' on page 3 undefined
on inp
ut line 201.


Package natbib Warning: Citation `noro' on page 3 undefined on input line
208.


Package natbib Warning: Citation `bukreyev1993vp35' on page 3 undefined
on inpu
t line 212.


Package natbib Warning: Citation `volchkov1998release' on page 3
undefined on i
nput line 212.


Package natbib Warning: Citation `guedj2018antiviral' on page 3 undefined
on in
put line 212.


Package natbib Warning: Citation `del2007vectored' on page 3 undefined on
input
 line 215.


Package natbib Warning: Citation `pfaller2015measles' on page 3 undefined
on in
put line 215.



[3{c:/texlive/2024/texmf-dist/fonts/enc/dvips/lm/lm-ec.enc}]

Package natbib Warning: Citation `flye' on page 4 undefined on input line
222.


Package natbib Warning: Citation `canu' on page 4 undefined on input line
222.
```

```
Package natbib Warning: Citation `accuvir' on page 4 undefined on input
line 22
2.


Package natbib Warning: Citation `pbd' on page 4 undefined on input line
222.


Package natbib Warning: Citation `medaka' on page 4 undefined on input
line 222
.

LaTeX Font Info:    Font shape `T1/Merriwthr-OsF/b/n' will be
(Font)              scaled to size 7.0pt on input line 239.

LaTeX Warning: `h' float specifier changed to `ht'.


LaTeX Warning: File `images/hiv.png' not found on input line 283.


! Package pdftex.def Error: File `images/hiv.png' not found: using draft
settin
g.

See the pdftex.def package documentation for explanation.
Type  H <return>  for immediate help.
 ...

l.283        {images/hiv.png}

Try typing  <return>  to proceed.
If that doesn't work, type  X <return>  to quit.


LaTeX Warning: File `images/covidbar.png' not found on input line 286.


! Package pdftex.def Error: File `images/covidbar.png' not found: using
draft s
etting.

See the pdftex.def package documentation for explanation.
Type  H <return>  for immediate help.
 ...

l.286 ...s[width=1\textwidth]{images/covidbar.png}

Try typing  <return>  to proceed.
If that doesn't work, type  X <return>  to quit.
```

```
Underfull \vbox (badness 2865) has occurred while \output is active []



LaTeX Warning: `h' float specifier changed to `ht'.



[4] [5]

Package natbib Warning: Citation `ip2015minion' on page 6 undefined on
input li
ne 448.


Package natbib Warning: Citation `delahaye2021sequencing' on page 6
undefined o
n input line 448.


Underfull \vbox (badness 10000) has occurred while \output is active []



LaTeX Font Info:    Trying to load font information for TS1+Merriwthr-OsF
on in
put line 454.
(c:/texlive/2024/texmf-dist/tex/latex/merriweather/TS1Merriwthr-OsF.fd
File: TS1Merriwthr-OsF.fd 2020/08/30 (autoinst) Font definitions for
TS1/Merriw
thr-OsF.
)
LaTeX Font Info:    Font shape `TS1/Merriwthr-OsF/m/n' will be
(Font)              scaled to size 7.5pt on input line 454.
Package microtype Info: Loading generic protrusion settings for font
family
(microtype)             `Merriwthr-OsF' (encoding: TS1).
(microtype)             For optimal results, create family-specific
settings.
(microtype)             See the microtype manual for details.
LaTeX Font Info:    Font shape `T1/Merriwthr-OsF/m/n' will be
(Font)              scaled to size 6.8438pt on input line 489.

Package natbib Warning: Citation `swh_benchmark' on page 6 undefined on
input l
ine 499.



[6{c:/texlive/2024/texmf-
dist/fonts/enc/dvips/merriweather/merriwthr_owzwzj.enc
}]
Underfull \vbox (badness 10000) has occurred while \output is active []
```

```
Package natbib Warning: Citation `watson2019errors' on page 7 undefined
on inpu
t line 598.


Package natbib Warning: Citation `pilon' on page 7 undefined on input
line 600.



Package natbib Warning: Citation `NextPolish' on page 7 undefined on
input line
 600.


Package natbib Warning: Citation `flye' on page 7 undefined on input line
652.


LaTeX Warning: File `images/5VM_diversity.png' not found on input line
665.


! Package pdftex.def Error: File `images/5VM_diversity.png' not found:
using dr
aft setting.

See the pdftex.def package documentation for explanation.
Type  H <return>  for immediate help.
 ...

l.665 ...extwidth, clip]{images/5VM_diversity.png}

Try typing  <return>  to proceed.
If that doesn't work, type  X <return>  to quit.



[7]
Underfull \vbox (badness 10000) has occurred while \output is active []




Package natbib Warning: Citation `accuvir' on page 8 undefined on input
line 72
7.
```

```
LaTeX Warning: File
`images/hiv_50x_execution_times_runtime_comparison.png' not
 found on input line 741.


! Package pdftex.def Error: File
`images/hiv_50x_execution_times_runtime_compar
ison.png' not found: using draft setting.

See the pdftex.def package documentation for explanation.
Type  H <return>  for immediate help.
 ...

l.741 ...x_execution_times_runtime_comparison.png}

Try typing  <return>  to proceed.
If that doesn't work, type  X <return>  to quit.


LaTeX Warning: File
`images/hiv_100x_execution_times_runtime_comparison.png' no
t found on input line 744.


! Package pdftex.def Error: File
`images/hiv_100x_execution_times_runtime_compa
rison.png' not found: using draft setting.

See the pdftex.def package documentation for explanation.
Type  H <return>  for immediate help.
 ...

l.744 ...x_execution_times_runtime_comparison.png}

Try typing  <return>  to proceed.
If that doesn't work, type  X <return>  to quit.


LaTeX Warning: File
`images/hiv_200x_execution_times_runtime_comparison.png' no
t found on input line 747.


! Package pdftex.def Error: File
`images/hiv_200x_execution_times_runtime_compa
rison.png' not found: using draft setting.

See the pdftex.def package documentation for explanation.
Type  H <return>  for immediate help.
 ...

l.747 ...x_execution_times_runtime_comparison.png}

Try typing  <return>  to proceed.
```

```
If that doesn't work, type  X <return>  to quit.


Overfull \hbox (4.71582pt too wide) in paragraph at lines 741--748
[][]   []   []
 []


LaTeX Warning: File
`images/measles_50x_execution_times_runtime_comparison.png'
 not found on input line 753.


! Package pdftex.def Error: File
`images/measles_50x_execution_times_runtime_co
mparison.png' not found: using draft setting.

See the pdftex.def package documentation for explanation.
Type  H <return>  for immediate help.
 ...

l.753 ...x_execution_times_runtime_comparison.png}

Try typing  <return>  to proceed.
If that doesn't work, type  X <return>  to quit.


LaTeX Warning: File
`images/measles_100x_execution_times_runtime_comparison.png
' not found on input line 756.


! Package pdftex.def Error: File
`images/measles_100x_execution_times_runtime_c
omparison.png' not found: using draft setting.

See the pdftex.def package documentation for explanation.
Type  H <return>  for immediate help.
 ...

l.756 ...x_execution_times_runtime_comparison.png}

Try typing  <return>  to proceed.
If that doesn't work, type  X <return>  to quit.


LaTeX Warning: File
`images/measles_200x_execution_times_runtime_comparison.png
' not found on input line 759.


! Package pdftex.def Error: File
`images/measles_200x_execution_times_runtime_c
omparison.png' not found: using draft setting.
```

```
See the pdftex.def package documentation for explanation.
Type  H <return>  for immediate help.
 ...

l.759 ...x_execution_times_runtime_comparison.png}

Try typing  <return>  to proceed.
If that doesn't work, type  X <return>  to quit.


Overfull \hbox (4.71582pt too wide) in paragraph at lines 753--760
[][]   []   []
 []


LaTeX Warning: File
`images/covid_50x_execution_times_runtime_comparison.png' n
ot found on input line 765.


! Package pdftex.def Error: File
`images/covid_50x_execution_times_runtime_comp
arison.png' not found: using draft setting.

See the pdftex.def package documentation for explanation.
Type  H <return>  for immediate help.
 ...

l.765 ...x_execution_times_runtime_comparison.png}

Try typing  <return>  to proceed.
If that doesn't work, type  X <return>  to quit.


LaTeX Warning: File
`images/covid_100x_execution_times_runtime_comparison.png'
not found on input line 768.


! Package pdftex.def Error: File
`images/covid_100x_execution_times_runtime_com
parison.png' not found: using draft setting.

See the pdftex.def package documentation for explanation.
Type  H <return>  for immediate help.
 ...

l.768 ...x_execution_times_runtime_comparison.png}

Try typing  <return>  to proceed.
If that doesn't work, type  X <return>  to quit.
```

```
LaTeX Warning: File
`images/covid_200x_execution_times_runtime_comparison.png'
not found on input line 771.


! Package pdftex.def Error: File
`images/covid_200x_execution_times_runtime_com
parison.png' not found: using draft setting.

See the pdftex.def package documentation for explanation.
Type  H <return>  for immediate help.
 ...

l.771 ...x_execution_times_runtime_comparison.png}

Try typing  <return>  to proceed.
If that doesn't work, type  X <return>  to quit.


Overfull \hbox (4.71582pt too wide) in paragraph at lines 765--772
[][]   []   []
 []

LaTeX Font Info:    Font shape `T1/Merriwthr-OsF/b/n' will be
(Font)              scaled to size 6.8438pt on input line 778.

LaTeX Warning: File `images/hiv_memory_consumption_plot.png' not found on
input
 line 787.


! Package pdftex.def Error: File `images/hiv_memory_consumption_plot.png'
not f
ound: using draft setting.

See the pdftex.def package documentation for explanation.
Type  H <return>  for immediate help.
 ...

l.787     {images/hiv_memory_consumption_plot.png}

Try typing  <return>  to proceed.
If that doesn't work, type  X <return>  to quit.


LaTeX Warning: File `images/measles_memory_consumption_plot.png' not
found on i
nput line 789.


! Package pdftex.def Error: File
`images/measles_memory_consumption_plot.png' n
ot found: using draft setting.
```

```
See the pdftex.def package documentation for explanation.
Type  H <return>  for immediate help.
 ...

l.789 ...ages/measles_memory_consumption_plot.png}

Try typing  <return>  to proceed.
If that doesn't work, type  X <return>  to quit.


LaTeX Warning: File `images/covid_memory_consumption_plot.png' not found
on inp
ut line 791.


! Package pdftex.def Error: File
`images/covid_memory_consumption_plot.png' not
 found: using draft setting.

See the pdftex.def package documentation for explanation.
Type  H <return>  for immediate help.
 ...

l.791 ...images/covid_memory_consumption_plot.png}

Try typing  <return>  to proceed.
If that doesn't work, type  X <return>  to quit.

LaTeX Font Info:    Font shape `TS1/Merriwthr-OsF/m/n' will be
(Font)              scaled to size 6.0pt on input line 794.

LaTeX Warning: Float too large for page by 7.9475pt on input line 796.



[8] [9]

Package natbib Warning: Citation `song_pvga_2025' on page 10 undefined on
input
 line 818.


Underfull \hbox (badness 1237) in paragraph at lines 834--836
\T1/Merriwthr-OsF/b/n/7.5 (+20) Supplementary Ta-ble 2. \T1/Merriwthr-
OsF/m/up/
7.5 (+20) Re-sults on sim-u-lated PacBio HIV-1
 []


Package natbib Warning: Citation `swh_benchmark' on page 10 undefined on
input
line 851.
```

```
Underfull \hbox (badness 1226) in paragraph at lines 857--858
[]\T1/Merriwthr-OsF/m/up/7.5 (+20) SARS-CoV-2 iso-late Wuhan-Hu-1 is
avail-able
 in the NCBI
 []


Underfull \hbox (badness 3525) in paragraph at lines 858--859
[]\T1/Merriwthr-OsF/m/up/7.5 (+20) SARS-CoV-2 iso-late INEI121916 is
avail-able
 int the NCBI
 []





[10]
No file main.bbl.

Package natbib Warning: There were undefined citations.





[11

]
enddocument/afterlastpage: lastpage setting LastPage.
(./main.aux)
 ***********
LaTeX2e <2024-06-01> patch level 2
L3 programming layer <2020/03/25>
 ***********

LaTeX Font Warning: Size substitutions with differences
(Font)              up to 1.0pt have occurred.


LaTeX Font Warning: Some font shapes were not available, defaults
substituted.


LaTeX Warning: There were multiply-defined labels.

Package rerunfilecheck Info: File `main.out' has not changed.
(rerunfilecheck)             Checksum:
AE4CC2F69179BA763E55EF14DA252DE6;6137.
 )
Here is how much of TeX's memory you used:
 36951 strings out of 473583
 775317 string characters out of 5732343
 2027908 words of memory out of 5000000
```

```
 58503 multiletter control sequences out of 15000+600000
 2029268 words of font info for 612 fonts, out of 8000000 for 9000
 1141 hyphenation exceptions out of 8191
 123i,12n,131p,1231b,1073s stack positions out of
10000i,1000n,20000p,200000b,200000s
<c:/texlive/2024/texmf-dist/fonts/type1/sorkin/merriweather/Merriwthr-
Bold.pf
b><c:/texlive/2024/texmf-dist/fonts/type1/sorkin/merriweather/Merriwthr-
BoldIta
lic.pfb><c:/texlive/2024/texmf-
dist/fonts/type1/sorkin/merriweather/Merriwthr-I
talic.pfb><c:/texlive/2024/texmf-
dist/fonts/type1/sorkin/merriweather/Merriwthr
-Regular.pfb><c:/texlive/2024/texmf-
dist/fonts/type1/public/amsfonts/cm/cmsy10.
pfb><c:/texlive/2024/texmf-
dist/fonts/type1/public/amsfonts/cm/cmsy6.pfb><c:/te
xlive/2024/texmf-
dist/fonts/type1/public/amsfonts/euler/eurm7.pfb><c:/texlive/2
024/texmf-dist/fonts/type1/public/lm/lmtt8.pfb>
Output written on main.pdf (11 pages, 346832 bytes).
PDF statistics:
 297 PDF objects out of 1000 (max. 8388607)
 264 compressed objects within 3 object streams
 62 named destinations out of 1000 (max. 500000)
 226069 words of extra memory for PDF output out of 266212 (max.
10000000)
```

Manuscript

Click here to access/download;Manuscript;PVGA_gigascience.pdf

Placeholder for OUP logo oup.pdf

GigaScience, 2023, 1–12

doi: xx.xxxx/xxxx
Manuscript in Preparation
Paper

PAPER

PVGA: A Precise Viral Genome Assembler Using Iterative Alignment Graph

Zhi Song[1], Dehan Cai[3], Yanni Sun[3,*] and Lusheng Wang[1,2 *]

[1]Department of Computer Science, City University of Hong Kong and [2]City University of Hong Kong Shenzhen Research Institution and [3]Department of Electrical Engineering, City University of Hong Kong

*Correspondence address. Yanni Sun, Electrical Engineering, City University of Hong Kong, Kowloon, China. Email: yannisun@cityu.edu.hk; Lusheng Wang, Department of Computer Science, City University of Hong Kong, Kowloon, China. Email: cswangl@cityu.edu.hk

ORCID iDs: Zhi Song [0000-0001-9720-1084]; Dehan Cai [0000-0002-8148-4574]; Yanni Sun [0000-0003-1373-8023]; Lusheng WANG [0000-0002-4344-8791].

Abstract

Background: Viral genome analysis is crucial for understanding viruses evolution and mutation. Investigations into viral evolutionary dynamics and mutation patterns have garnered significant research attention since the outbreak of COVID-19. The basic structure of many virus genomes is highly conserved [1]. RNA viruses have high mutation rates, and single-nucleotide variations may induce substantial phenotypic alterations in terms of viral function and pathogenicity. Thus, special assembly methods are required for viral genome analysis.
Result: PVGA starts with a reference genome and the sequencing reads. The first step in PVGA involves constructing an alignment graph based on a reference genome and the set of input sequencing reads. Then the optimal genomic path is determined through dynamic programming, maximizing the cumulative edge weights that reflect read support density across the alignment graph. The obtained path corresponds to a refined genome. Finally, we repeat the process by using the new reference genomes until no further improvement is possible. We evaluate PVGA's performance across both assembly and polishing tasks using simulated and real datasets including both long reads and short reads. The experiments demonstrate that PVGA always outperforms popular existing programs in terms of the quality of assembly results, while the running time of our method is compatible to others. In particular, simulated Nanopore datasets show that our method can correctly report the true genomes with 0 mismatch and 0 indels.
Conclusions: PVGA is a novel viral genome assembler that seamlessly integrates assembly and polishing into a unified workflow. Its design prioritizes high accuracy, enabling the detection of subtle genomic variations that can impact viral function and pathogenicity. By addressing the unique challenges of viral genome assembly, PVGA provides a reliable and precise solution for advancing our understanding of viral evolution and behavior.

Key words: Genome assembler; Virus genome; Alignment graph; Maximum total weight path; Iterative method

Introduction

Viral genome analysis is crucial for understanding viruses evolution and mutation. Investigations into viral evolutionary dynamics and mutation patterns have garnered significant research attention since the outbreak of COVID-19. The basic structure of many virus genomes is highly conserved [1]. RNA viruses have high mutation rates, and single-nucleotide variations may induce substantial phenotypic alterations in terms of viral function and pathogenicity. For example, genetic mutations in the coronavirus genome that alter the spike protein can affect its ability to interact with host cells, thereby affecting transmissibility and disease severity [2]. Thus, special assembly methods are required for viral genome analysis.

Sequencing of viral genomes primarily relies on Next-Generation Sequencing (NGS) and Third-Generation Sequencing (TGS) technologies. NGS is recognized for its short read lengths and high accuracy, with platforms such as Illumina. However, due to the short read lengths and the presence of repetitive regions, it is often challenging to assemble genomes accurately using NGS data. Third-generation sequencing (TGS) technologies, such as Nanopore and PacBio, are effective for resolving complex

Compiled on: May 15, 2025.
Draft manuscript prepared by the author.

genome structures and repetitive regions due to their longer read lengths. In comparison with NGS, TGS tends to have higher error rates. For instance, Nanopore sequencing relies on measuring electrical current changes as DNA passes through a nanopore, and factors such as pore condition, molecule speed, and signal noise can interfere with base-calling accuracy, making it difficult to distinguish adjacent bases, thereby reducing the quality of the reads [3]. PacBio sequencing, using its Single Molecule Real-Time (SMRT) technology [4], is also widely applied for TGS. SMRT sequencing is well-suited for detecting structural variations and resolving repetitive regions, but it frequently introduces insertion and deletion (indel) errors. To improve accuracy, PacBio introduced HiFi(high-fidelity) sequencing, which produces highly accurate long reads by repeatedly sequencing the same molecule [5]. However, this increased accuracy comes at a higher cost. Achieving high-quality viral genome assembly requires balancing sequencing accuracy with cost. Both the choice of sequencing technology and the assembly algorithms play critical roles in producing reliable genome assemblies.

Genome assembly techniques are broadly categorized into two types: De Novo assembly and reference-guided assembly. De Novo assembly tools include Velvet [6], ABySS [7], SPAdes [8], Flye [9], Canu [10] and Translig [11], which reconstructs the genome without relying on a reference genome. However, De Novo methods often encounter challenges in highly repetitive regions, which may lead to misassemblies, redundant contigs, or gaps. Moreover, in regions of low coverage, De Novo assembly may produce incomplete or missing sequences, and further introduce gaps and errors. With the help of the reference genome sequence, one can obtain the locations of reads in the genome. Thus, reference-guided assembly methods can possibly fill gaps between reads and improve prediction accuracy in low-coverage regions. Famous reference-guided assembly methods include Novoalign [12], Maq [13], iVar [14], Accuvir [15] and bcftools [16].

There are some assemblers that are optimized for viruses. For instance, IVA was developed as a De Novo assembler for RNA viruses, utilizing paired-end datasets to achieve more accurate assemblies [17]. Similarly, Accuvir [15] introduced a reference-based long-read assembler for viruses, primarily employing diverse beam search algorithms on alignment graphs to improve accuracy. In addition to assembly tools, genome polishing methods have become increasingly important for enhancing assembly accuracy by correcting errors using high-accuracy reads. Pilon [18] improves genome assemblies by analyzing read alignments, constructing a pileup structure to evaluate base-level evidence, and iteratively adjusting the assembly based on read quality and consistency. NextPolish [19] employs a combination of alignment-based error correction and iterative consensus polishing, utilizing short-read data to precisely correct mismatches and indels, further refining the final genome assembly. However, despite these advancements, current genome assembly tools still fail to achieve the requisite base-level accuracy for viral genome assembly.

In this paper, we present PVGA, a novel viral genome assembler that can perform both assembly and polishing, effectively handling both long-read and short-read sequencing data. PVGA starts with a reference genome and utilizes the sequencing reads directly to reduce noise. The first step in PVGA involves constructing an alignment graph based on a reference genome and the set of input sequencing reads. Then the optimal genomic path is determined through dynamic programming, maximizing the cumulative edge weights that reflect read support density across the alignment graph. Finally, we repeat the process by using the new reference genomes until no further improvement is possible.

We evaluated PVGA's performance across both assembly and polishing tasks using simulated and real datasets including both long reads and short reads. The results demonstrate that PVGA consistently outperforms popular existing programs. In particular, simulated Nanopore datasets show that our method can correctly report the true genomes with 0 mismatch and 0 indels, except for some small errors at the two ends of the genomes.

Methods

Our new method contains three steps. Step 1: We construct an alignment graph based on the set of input reads using an initial reference genome as the backbone. The initial reference genome should be from the same species. One can also generate an initial reference genome using an existing De Novo assembler. Step 2: After constructing the alignment graph, we apply a dynamic programming algorithm to select a path supported by the largest number of read coverage and construct a new reference genome based on the path. Step 3: We then use the latest reference genome as the backbone to repeat Steps 1-2. The process stops when the new reference genome is identical to the old one.

Alignment graph construction

The graph construction method is inspired by the hierarchical genome-assembly process (HGAP) proposed by Chin [20]. The input contains two parts: read sequences and a backbone sequence. First, we construct the initial graph G_b with n nodes $v_1, v_2, \ldots v_n$ and $n-1$ edges based on the backbone sequence $S = s_1 s_2 \ldots s_n$, where each node v_i is labeled with the letter s_i and there is an edge (v_i, v_{i+1}) connecting the two consecutive nodes. We then align each read $R = r_1 r_2 \ldots r_k$ with the reference sequence G_b.

In the alignment, if r_i is aligned to an identical letter s_j in the backbone sequence, then r_i corresponds to the existing node v_j in the graph G_b. The weight of the edge (v_{j-1}, v_j) will be incremented by one, where v_{j-1} is the predecessor node of v_j. If r_i is aligned with a space or a letter s_j not identical to r_i, we will create a new node u_i labeled with r_i and add an edge (u_{i-1}, u_i) with weight 1, where u_{i-1} is the node corresponding to the previous letter r_{i-1}. See Fig. 1(b).

We will repeat the above process until all reads have been applied. The obtained intermediate graph is denoted as $G_I = (V, E)$. In the intermediate graph G_I, each edge is assigned a weight of 1, representing the number of supporting reads. To reduce the complexity of G_I, we merge nodes with the same label and the same parent repeatedly. When merging nodes into a new node u' with its predecessor v, the weight of the resulting edge (v, u') is updated to reflect the total number of supporting reads. Finally, if there are multiple edges between any two nodes u and v in G_I, we combine them into a single edge and update the weight accordingly. This process results in a simplified final alignment graph G.

Finding a directed path in the alignment graph with maximum total weight

The weight on each edge is the number of supporting reads. In order to find a new reference sequence, we will try to find a path in G containing the maximum total weight. Such a path is the path supported by the largest number of reads.

We do a topological sorting on the set of nodes in G and obtain a linear order among the set of nodes in G. Let DP[v] denote the maximum total weight of paths ending at node v. The value of DP[v] can be computed as follows.

$$DP[u] = \max_{v \in Pred(u)} \{DP[v] + w(v, u)\}, \tag{1}$$

where $w(v, u)$ is the weight of the edge from node v to node u. We can compute all the DP[v]s according to the topological order. After that, we will find a node v with the largest DP[v] value and use a standard backtracking process to get a path with maximum total weight on G ending at v.

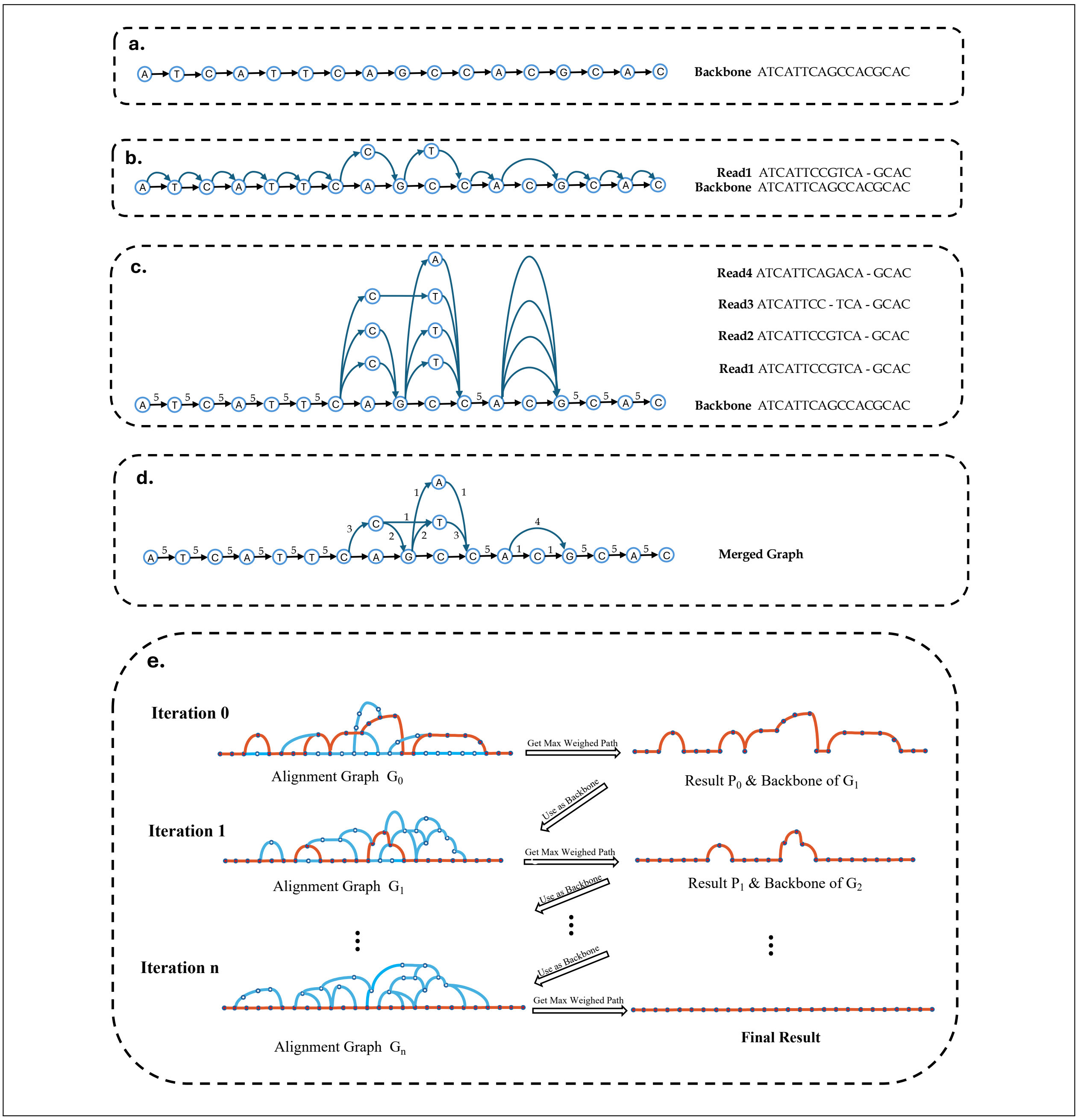

Figure 1. Flowchart of construction of alignment graph and iteration process. **a** PVGA takes a reference Genome as backbone graph G_B.
b PVGA aligns the first read Read1 to the backbone.
c Four reads are aligned with the backbone, awaiting the subsequent merging process.
d PVGA merges edges that point to the same node; the new edge's weight is equal to the sum of the weights of the merged edges. This process can be performed either after aligning all reads or during the alignment process, with a final merge conducted after all reads have been aligned.
e Iteratively construct the alignment graph using the result from the previous iteration as the backbone.

Running time: To compute each $DP[u]$ in equation (1), we need $O(Deg(u))$ time, where $Deg(u)$ is the in-degree of u. Therefore, the total running time complexity of this dynamic programming algorithm is $O(|E|)$, where $|E|$ represents the total number of edges in the graph.

Updating the reference genome iteratively

The quality of the obtained maximum weight path heavily depends on the initial reference genome, as errors or biases in the initial reference can impact the alignment and subsequent path computation. To address this, we use the obtained maximum weight path as a new reference genome. A new alignment graph is then re-constructed using all the input reads and the updated reference genome. The maximum weight path is computed again based on this updated graph. This process is repeated iteratively to refine the assembly. With each iteration, the alignment graph and the resulting path become more accurate and consistent. Iteration stops when the genome obtained from the current iteration is identical to the genome from the previous iteration. This indicates that the backbone and the assembly result have reached a consistent state and no further adjustment is possible using this method. This condition for algorithm termination seems to be very strong and one may worry about the running time of this condition.

Recall that PVGA is for virus genome assembly and the genome size is relatively small. Experimental results indicate that our

method exhibits rapid convergence, often requiring no more than 3 or 4 iterations to reach a stable solution in practical applications. The running time comparison is in Figure 5. Moreover, this iterative method can reach high-quality results and experiments show that our iterative method always outperforms the state-of-art methods.

Results

Datasets

We evaluate PVGA using both simulated and real viral sequencing data. To simulate a broad spectrum of sequencing conditions, we use Badread [21], which is designed for generating various kinds of simulated genomes. All real genome data and real sequencing reads used in this study are sourced from the National Center for Biotechnology Information (NCBI). These datasets include the following viral strains.

HIV-1:
HIV-specific data provides a critical benchmark for evaluating assembly methods, given its widespread availability in public databases. We use the 89.6 strain (GenBank: U39362.2) as the target genome, and generate simulated reads in bulk, with sizes detailed in the next subsection, to serve as the ground truth. The HXB2 strain (GenBank: K03455.1) is selected as the reference genome for constructing the initial alignment graph, which shares 93.58% similarity with the ground truth 89.6 strain.

The haplotype benchmarking dataset [22] contains mixed PacBio reads from five strains (HXB2, 89.6, JR-CSF, NL4-3, YU-2). To obtain reads from a single strain, we first align the reads to the genomes of these strains using minimap2 [23]. We then extract the reads based on their closest aligned genome (identified by samtools [24]).

SARS-CoV-2:
SARS-CoV-2 sequencing data is characterized by extensive genomic length and high sequence homology. We select SARS-Cov-2 isolate Wuhan-Hu-1 (NCBI Reference Sequence: NC_045512.2) as the reference genome and SARS-Cov-2 isolate (GenBank: OZ072292.1) as a target and to generate simulation reads.

Norovirus:
Noroviruses are common pathogens that can cause acute gastroenteritis. We obtain third-generation nanopore sequencing data of noroviruses(SRX10330013) from the National Food Virology Reference Centre at Health Canada [25]. This dataset consists of 5,741 spots, totaling 2.9 million bases, with the norovirus GII strain BMH19-097 serving as the ground truth genome. We use the complete genome of Norovirus GII (NC_039477.1) as the reference for graph construction. We also employ actual Illumina sequencing data of the norovirus. We utilize SRR13951201 (35.8M bases), SRR13951221 (60M bases), and SRR13951199(12.9M bases) as input reads, with corresponding ground truth data from Norovirus GII isolates BMH19-145, BMH13-039 and BMH14-056.

Ebola:
We utilize two Ebola virus (EBOV) genomes. The first genome corresponds to the Ebola virus (EBOV-May) Mayinga strain, isolated in Zarie in 1976. This genome consists of 18,959 base pairs and is publicly available under the NCBI accession number AF086833.2. EBOV-May is a negative-sense, single-stranded RNA virus of the genus Orthoebolavirus, encoding seven structural proteins such as nucleoprotein (NP), glycoprotein (GP), and RNA-dependent RNA polymerase (L) [26, 27]. The second Ebola virus strain is isolated from Macaca fascicularis and sequenced using IonTorrent technology. The genome, consisting of 18,871 base pairs, is publicly accessible under the NCBI accession number KY786027.1. It was assembled using the CLC Genomics Workbench v9.5.4 and serves as the ground truth genome for generating synthetic reads in this study. The metadata associated with this genome are part of BioProject PRJNA379115 and BioSample SAMN06603499, with additional information submitted by Guedj et al. [28]

Measles:
Measles virus (MV), a member of the genus Morbillivirus in the family Paramyxoviridae, is a highly contagious, negative-sense, single-stranded RNA virus. We utilize two Measles virus strains. The first genome, a complete reference genome of Measles morbillivirus, was sourced from the NCBI RefSeq database (accession number NC_001498.1). This genome consists of 15,894 base pairs. The second genome is the Measles virus genotype A transgenic strain vac2(GFP)H and serves as the ground truth genome for generating synthetic reads. This genome, available under the GenBank accession number MH144178.1, spans 16,728 base pairs and includes a transgenic insertion of the green fluorescent protein (GFP) gene. It was sequenced using Sanger dideoxy sequencing and has been used in experimental studies for functional and structural analyses [29, 30].

Evalution on simulation data

We evaluate the performance of several assemblers using simulated Nanopore and PacBio data. Tests are conducted on two viral strains, HIV-1, and SARS-CoV-2, under both standard and low coverage conditions to assess their robustness.

We compare our method PVGA with the state-of-the-art methods, including De Novo methods: Flye [9], Canu [10] and reference-based methods: Accuvir [15], PBDAG-Con [20] and Medaka [31]. In addition, to illustrate the improvement achieved by the iterative step of PVGA, we also present the results of our method without the iterative step (referred to as "PVGA (no_iter)")

We evaluate the assembly quality using several metrics, including genome fraction, mismatches, indels, indel length, and edit distance. Genome fraction represents the percentage of reference genome bases accurately matched by the assembled genome. Mismatches refer to the number of positions where the nucleotide in the assembly differs from the reference sequence. Indels refer to the number of insertions and deletions in the assembly relative to the reference. Indel length refers to the total length of the indels. Edit distance defines a minimum number of substitution and indel operations required to transform the assembled genome into the reference sequence. These metrics collectively measure the assembly's accuracy and its deviation from the ground truth genome.

Benchmarking on Standard-Length and Depth HIV-1 Simulated Data
As the genome length increases, all methods tend to show higher error rates. However, PVGA consistently outperforms the compared assemblers. For our simulations, the average lengths of reads for Nanopore and PacBio were set at 2k, 4k, and 6k, with sequencing depths of 50x, 100x, and 200x.

To simulate sequencing data, we used the Badread tool with the error model parameter set to nanopore2023, which was trained on ONT R10.4.1 reads. The identity was set to (95, 99, 2.5), indicating a normal distribution with a mean of 95 and a standard deviation of 2.5. For PacBio sequencing, we set the error model in Badread to pacbio2021, trained on PacBio Sequel II HiFi reads. We use the same identity settings as (95, 99, 2.5). The results for Nanopore are presented in Figure 2 and Table 1, while the results for PacBio are shown in the supplementary materials.

From Table 1, we can see that PVGA achieves 0 mismatches and 0 indels with an edit distance of 3 across all Nanopore test cases. Figure 2, illustrates similar cases, where the average lengths of reads are 2kb and 4kb, respectively.

The PacBio results are illustrated in the supplementary docu-

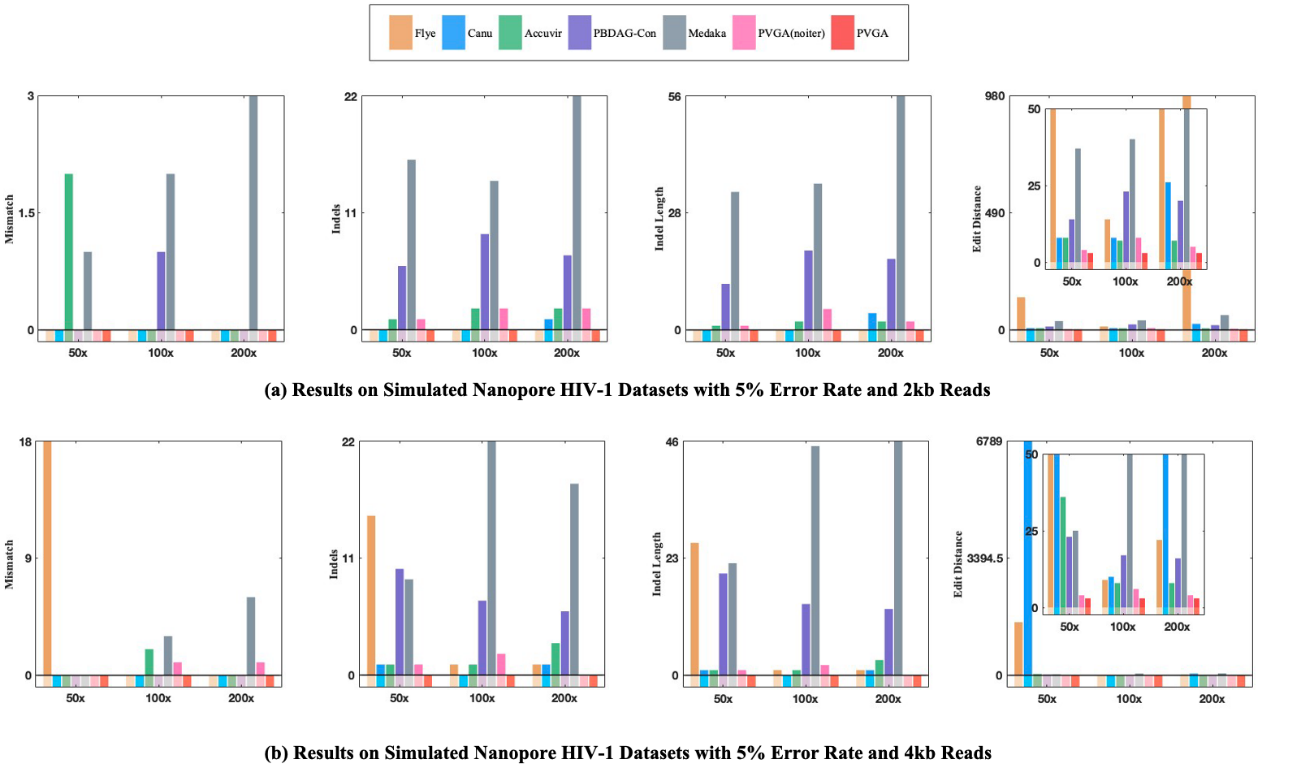

Figure 2. Results on simulated Nanopore HIV-1 datasets with an average read length of 2kb and 4kb, respectively. The 4 sub-figures in each row represent mismatch, indels, indel length, and edit distance from left to right, respectively.

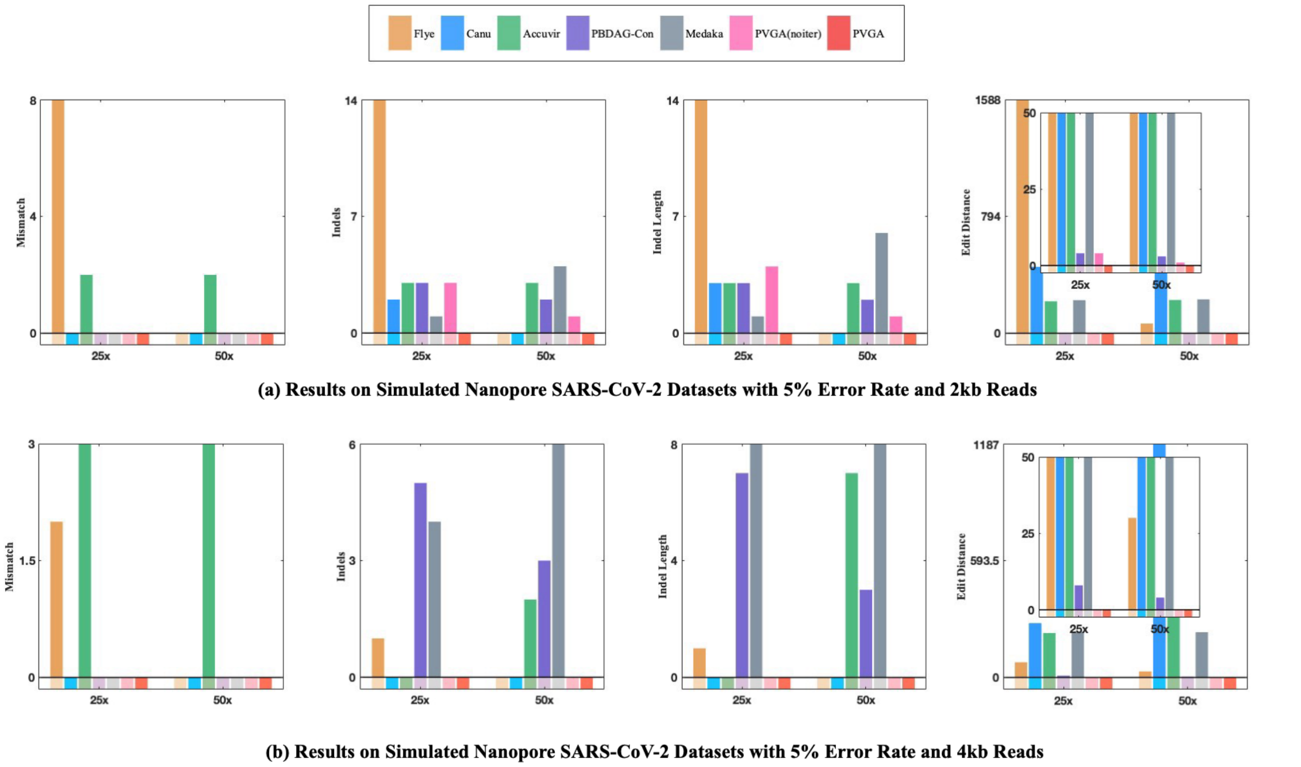

Figure 3. Results on simulated Nanopore SARS-CoV-2 datasets with an average read length of 2kb and 4kb, respectively. The 4 sub-figures in each row represent mismatch, indels, indel length, and edit distance from left to right, respectively.

Table 1. Results on Simulated Nanopore HIV-1 (genome length: 9713bp) Datasets with 5% Error Rate and an average read length of 6kb ("-" indicates that the assembler fails to produce a result.)

Reads depth	Tool	Genome fraction	Genome length	Mismatch	Indels	Indel length	Edit distance
50x	Flye	93.143	9067	0	0	0	686
	Canu	100	9748	0	0	0	35
	Accuvir	99.949	9709	0	2	3	4
	PBDAG-Con	99.969	9693	1	7	17	20
	medaka	99.969	9724	2	12	30	35
	PVGA (no_iter)	99.969	9706	0	2	4	7
	**PVGA**	**99.969**	**9710**	**0**	**0**	**0**	**3**
100x	Flye	96.757	9405	0	0	0	322
	Canu	-	-	-	-	-	-
	Accuvir	99.949	9706	0	2	2	7
	PBDAG-Con	99.969	9693	0	8	17	20
	medaka	99.969	9728	1	22	46	50
	PVGA (no_iter)	99.969	9709	2	1	1	6
	**PVGA**	**99.969**	**9710**	**0**	**0**	**0**	**3**
200x	Flye	100	9734	0	1	1	23
	Canu	-	-	-	-	-	-
	Accuvir	99.959	9710	0	1	1	5
	PBDAG-Con	99.969	9696	0	6	14	17
	medaka	99.969	9732	7	26	68	77
	PVGA (no_iter)	99.969	9705	1	5	5	9
	**PVGA**	**99.969**	**9710**	**0**	**0**	**0**	**3**

Canu could not construct the assembly graph required for genome assembly, when the reads dataset lacks sufficient independent reads and effective overlaps.

ment Section. As shown in Table 1 as well as Figure 2, the iterative refinement process enhances assembly accuracy. For instance, with 6k read lengths and a depth of 200x, the process reduces indels from 5 to 0, mismatches from 1 to 0, and the edit distance from 9 to 3. The experiments show that increasing coverage improves performance for some assemblers. For PVGA, a coverage of 50x is sufficient to achieve 0 mismatches and 0 indels.

Benchmarking on Low Coverage HIV-1 Simulated Data

Sequencing costs increase with coverage. An efficient assembler should perform well not only for high coverage but also under lower coverage conditions to support cost-effective sequencing. Thus, we further test the cases, where the coverage are 30x, 25x, and 20x, respectively, with an average read length of 2kb. See Table 2.

Table 2. Results on simulated Nanopore HIV-1 (genome length: 9713bp) datasets with low coverage: 30x, 25x and 20x

Reads depth	Tool	Genome fraction	Genome length	Mismatch	Indels	Indel length	Edit distance
30x	Flye	70.751	6917	0	1	1	2888
	Canu	93.452	11602	0	1	1	4147
	Accuvir	99.938	9707	1	3	4	11
	PBDAG-Con	99.969	9695	0	7	15	18
	Medaka	99.969	9714	1	9	18	22
	PVGA (no_iter)	99.969	9706	0	1	4	7
	**PVGA**	**99.969**	**9710**	**0**	**0**	**0**	**3**
25x	Flye	77.906	7576	0	0	0	2155
	Canu	93.452	11156	0	1	1	7325
	Accuvir	99.959	9713	3	3	4	11
	PBDAG-Con	99.969	9688	0	8	22	25
	Medaka	99.969	9711	2	8	17	22
	PVGA (no_iter)	99.969	9709	0	1	1	4
	**PVGA**	**99.969**	**9710**	**0**	**0**	**0**	**3**
20x	Flye	96.788	9440	0	1	1	353
	Canu	93.452	11549	0	4	5	5918
	Accuvir	99.949	9708	2	2	2	9
	PBDAG-Con	99.969	9688	0	13	24	27
	Medaka	99.969	9714	0	10	20	23
	PVGA (no_iter)	99.969	9710	0	0	0	3
	**PVGA**	**99.969**	**9710**	**0**	**0**	**0**	**3**

At these lower coverage levels, PVGA continues to show robust performance. As shown in Tables 2, at coverage levels of 30x, 25x, and 20x, while some assemblers experience a noticeable drop in accuracy, PVGA consistently achieves the best performance with 0 mismatches and 0 indels. To figure out the threshold at which the accuracy of PVGA begins to decline, we test the case, where the coverage ranges from 19x down to 15x with an average read length of 2kb. The results are shown in Table 3.

Table 3. Results on simulated Nanopore HIV-1 (genome length: 9713bp) datasets with low coverage (<20x)

Reads depth	Tool	Genome fraction	Genome length	Mismatch	Indels	Indel length	Edit distance
19x	Flye	88.963	8673	0	0	0	1104
	Canu	93.452	11853	0	5	6	5044
	Accuvir	99.866	9709	0	1	1	8
	PBDAG-Con	99.969	9682	1	11	28	32
	Medaka	99.969	9707	3	11	17	23
	PVGA (no_iter)	99.969	9707	0	1	3	6
	**PVGA**	**99.969**	**9710**	**0**	**0**	**0**	**3**
18x	Flye	93.926	9140	0	3	3	613
	Canu	99.969	9708	0	2	2	5
	Accuvir	99.959	9711	4	5	6	14
	PBDAG-Con	99.969	9686	0	11	24	27
	Medaka	99.969	9707	1	8	14	18
	PVGA (no_iter)	99.969	9709	0	1	1	4
	**PVGA**	**99.969**	**9710**	**0**	**0**	**0**	**3**
17x	Flye	72.614	7085	0	2	2	2694
	Canu	99.866	10907	0	5	5	1226
	Accuvir	99.753	9713	1	1	2	12
	PBDAG-Con	99.969	9685	0	8	25	28
	Medaka	99.969	9711	0	7	23	26
	PVGA (no_iter)	99.969	9710	0	0	0	3
	**PVGA**	**99.969**	**9710**	**0**	**0**	**0**	**3**
16x	Flye	93.092	9047	0	3	3	682
	Canu	93.452	11805	0	3	3	4126
	Accuvir	99.959	9719	0	7	10	14
	PBDAG-Con	99.969	9696	1	6	14	18
	Medaka	99.969	9707	0	1	3	7
	PVGA (no_iter)	99.969	9710	0	2	2	5
	**PVGA**	**99.969**	**9711**	**0**	**1**	**1**	**4**
15x	Flye	96.87	9402	0	7	7	315
	Canu	94.399	9163	1	6	6	551
	Accuvir	99.856	9693	0	7	8	22
	PBDAG-Con	99.969	9687	2	12	27	32
	Medaka	99.969	9703	3	6	13	19
	PVGA (no_iter)	99.969	9706	0	4	4	7
	**PVGA**	**99.969**	**9706**	**0**	**4**	**4**	**7**

As shown in Table 3, at coverages of 19x, 18x, and 17x, PVGA maintains zero indels. When the coverage drops to 16x, PVGA has its first indel with a length of 1, increasing to 4 at 15x. Despite this, PVGA continues to outperform all other assemblers, with the lowest indel count, indel length, mismatch rate, and edit distance. These results demonstrate PVGA's robustness under low coverage conditions, highlighting its potential to reduce sequencing costs while maintaining accuracy.

Benchmarking on Simulated SARS-CoV-2 Data

The SARS-CoV-2 virus is one of the RNA viruses with a long genome, approximately 29.9 kb. Although SARS-CoV-2 variants are highly similar, a few differences can lead to distinct biological properties such as pathogenicity, transmissibility, and immune response. Therefore, assembling an accurate SARS-CoV-2 genome is essential. In this section, we conduct experiments on simulated SARS-CoV-2 data at different depths (25x and 50x) and read lengths (2kb, 4kb, and 8kb) for both Nanopore and PacBio datasets to evaluate the performance of tools on a highly similar virus with a long genome. Results for Nanopore are presented in Table 4 and Figure 3, while PacBio results are included in the supplementary document.

Table 4 shows that both PVGA and Flye achieve zero indels and mismatches. However, PVGA demonstrates superior performance, reconstructing the genome flawlessly, with no errors even at the two ends of the genome.

Evaluation on poor sequencing conditions

With the advancement of sequencing technologies, there has been a significant leap in both the capabilities and quality of sequencing. For instance, PacBio sequencing technology can offer HiFi reads that provide an accuracy of 99.9%. However, some laboratories continue to rely on older sequencing equipment or encounter suboptimal results due to experimental limitations. In such cases, there is a need for an assembler capable of effectively handling data with relatively higher error rates. A study by the MinION Analysis and

Table 4. Results on simulated Nanopore SARS-CoV-2 (length: 29646bp) datasets with 5% error rate and an average read length of 8kb

Reads depth	Tool	Genome fraction	Genome length	Mismatch	Indels	Indel length	Edit distance
25x	Flye	100	29656	0	0	0	10
	Canu	96.711	28669	0	6	11	977
	Accuvir	100	29872	0	3	3	226
	PBDAG-Con	100	29646	0	14	1	2
	Medaka	100	29878	0	2	8	232
	PVGA (no_iter)	100	29644	0	4	4	4
	**PVGA**	**100**	**29646**	**0**	**0**	**0**	**0**
50x	Flye	99.98	29645	0	0	0	11
	Canu	97.834	28999	0	2	5	647
	Accuvir	100	29869	0	3	3	223
	PBDAG-Con	100	29672	0	0	0	26
	Medaka	100	29646	0	5	11	235
	PVGA (no_iter)	100	29643	0	0	0	3
	**PVGA**	**100**	**29646**	**0**	**0**	**0**	**0**

Reference Consortium reported that the median total error of all 2D reads was 12%, with 2D pass reads showing a slightly lower error rate of 10.5% [32]. Additionally, after basecalling, the global error rate of raw reads is typically around 10% [33].

To simulate poor sequencing conditions, we configured the following parameters for evaluating assembler performance under suboptimal data quality: We apply a truncated normal distribution of basecall identity (range: 85–95%, mean: 90%, SD: 5%), resulting in an average read error rate of 10% and an upper accuracy bound of 95%. We assign uniform sequencing depth (30×) and average read length (4 kb) across all viral genomes (HIV-1, Measles, Ebola) to standardize suboptimal quality conditions. The results are shown in Table 5.

Table 5. Results on simulated Nanopore HIV-1, Measles, Ebola virus datasets with 10% error rate and an average read length of 4kb with an average depth of 30x

Virus	Tool	Genome fraction (%)	Genome length	Mismatch	Indels	Indel length	Edit distance
HIV-1 (Length: 9713bp)	Flye	100	9732	0	16	17	53
	Canu	99.053	16468	0	36	43	6993
	Accuvir	99.835	9683	0	14	14	30
	PBDAG-Con	99.969	9645	1	26	67	71
	PVGA (no_iter)	99.969	9720	1	26	38	42
	**PVGA**	**99.969**	**9717**	**0**	**7**	**11**	**14**
Measles (Length: 16728bp)	Flye	99.988	13899	0	17	20	2829
	Canu	99.815	16674	2	21	23	56
	Accuvir	99.994	16725	4	9	12	17
	PBDAG-Con	94.996	15879	1	10	12	850
	PVGA (no_iter)	100	16733	2	12	17	19
	**PVGA**	**100**	**16734**	**1**	**5**	**8**	**9**
Ebola (Length: 18871bp)	Flye	99.989	18851	1	18	18	21
	Canu	99.862	18824	1	16	21	48
	Accuvir	100	18979	9	26	28	123
	PBDAG-Con	100	18858	0	13	13	13
	PVGA (no_iter)	100	18874	0	12	13	13
	**PVGA**	**100**	**18879**	**0**	**7**	**8**	**8**

For HIV-1, the 89.6 strain is used as the target to simulate reads, with the HXB2 strain serving as the backbone. For the measles virus, the NC_001498.1 sequence (15,894 bp) is used as the backbone, while the vac2(GFP)H sequence (Length: 16,728 bp) served as the target. For the Ebola virus, the EBOV-May strain (18,959 bp) is used as the backbone, with the KY786027 strain serving as the ground truth for read simulation.

Evaluation on real data

Although there are many available long-read sequencing datasets of viruses, most of them lack ground-truth genomes for validation. Thus, we use Norovirus and HIV-1 to evaluate the tools' performance, as they have ground-truth genomes from both long-read and short-read sequencing data.

For the HIV-1 real datasets, we collect PacBio sequencing data from a mock HIV-1 community [22]. To create the datasets for viral genome reconstruction, we separate this dataset into read sets from five HIV-1 strains, by aligning them to the ground-truth genomes using the best hit. We test the 89.6, JR-CSF, and YU-2 subtypes, using HXB2 as the backbone to construct the alignment graph. Given that real data often contains gaps between reads, most De Novo assemblers fail to achieve consensus or produce only very short contigs. As shown in Table 6, as for the 89.6 Strain, Canu produces a contig of only 4,593 base pairs. Flye, on the other hand, encounters errors during real data processing, resulting in an unsuccessful assembly. Due to the lower quality of real reads, assemblers display higher mismatches, indels, and edit distances in the HIV-1 89.6 strain than observed in simulations. However, PVGA still outperforms all other assemblers. In the JR-CSF results, apart from Canu, which failed to assemble a complete genome, only PVGA and PBDAG-Con maintained single-digit mismatches, with PVGA showing lower indels, indel length, and edit distance.

Table 6. Results on real HIV-1 strain datasets(89.6, JR-CSF, YU-2)

Strain	Tool	Genome fraction	Genome length	Mismatch	Indels	Indel length	Edit distance
89.6 Strain (Length: 9713bp)	Canu	47.287	4593	6	0	0	5127
	Accuvir	99.990	9710	33	4	4	30
	Medaka	99.856	9733	25	6	22	74
	PBDAG-Con	100	9711	24	2	2	40
	PVGA (no_iter)	100	9709	25	2	2	36
	**PVGA**	**100**	**9710**	**24**	**1**	**1**	**28**
JR-CSF (Length: 9535bp)	Canu	88.512	8448	6	1	1	1320
	Accuvir	99.727	9736	36	3	3	241
	PBDAG-Con	99.99	9720	5	8	20	221
	Medaka	97.735	9535	33	8	42	316
	PVGA (no_iter)	99.99	9610	3	1	1	159
	**PVGA**	**99.99**	**9610**	**3**	**1**	**1**	**159**
YU-2 (Length: 9706bp)	Canu	86.750	8473	5	2	4	1343
	Accuvir	99.727	9713	5	5	9	16
	PBDAG-Con	100.000	9705	4	7	19	23
	Medaka	100.000	9698	28	9	52	80
	PVGA (no_iter)	100.000	9615	3	9	13	16
	**PVGA**	**100.000**	**9617**	**3**	**7**	**11**	**14**

We also test our method on real norovirus data (SRX10330013). As shown in Table 7, our method PVGA results in the fewest mismatches, indels, and the lowest edit distance among the five assemblers evaluated. This demonstrates that our PVGA method more effectively utilizes information from the alignment graph compared to PBDAG-Con, which focuses on assigning scores to nodes to maximize consensus, and Accuvir, which employs diverse beam search. While a diverse beam search approach increases the diversity of candidate paths, it often falls into local optima, failing to achieve the best results.

Table 7. Results on real Nanopore noroviruses (SRX10330013), with ground truth genome as Norovirus GII isolate BMH19-097 (Length: 7618 bp)

Tool	Genome fraction	Genome length	Mismatch	Indels	Indel length	Edit distance
Flye	92.964	7097	0	11	13	577
Canu	99.593	7632	0	12	14	104
Accuvir	99.396	7564	1	8	10	57
PBDAG-Con	99.383	7562	1	7	9	57
**PVGA**	**99.383**	**7569**	**0**	**4**	**4**	**51**

To assess the effectiveness of the PVGA method with extensive short-read datasets, we employ actual Illumina sequencing data of the norovirus. The Norovirus GII complete genome (NC_039477.1) serves as the reference framework for graph construction. We utilize SRR13951201, SRR13951221, and SRR13951199 as input reads, with corresponding ground truth data from Norovirus GII isolates BMH19-145, BMH13-039, and BMH14-056. The results are in Table 8. In all three Illumina norovirus datasets, PVGA achieves exceptional accuracy, with no mismatches, indels, or indel length errors except for some small misalignment at the two ends of the genomes. This verifies PVGA's excellent performance in assembling short-read datasets as well.

Table 8. Results on real Illumina norovirus

Reads	Genome fraction	Genome length	Mismatch	Indels	Indel length	Edit distance
SRR13951201	100	7572	0	0	0	2
SRR13951221	100	7567	0	0	0	17
SRR13951199	99.574	7485	0	0	0	20

Ground truth genome lengths: SRR13951201 (BMH19-145): 7,570 bp; SRR13951221 (BMH13-039): 7,550 bp; SRR13951199 (BMH14-056): 7,505 bp.

Benchmarking the capability of polishing

The results from low-coverage HIV-1 sequencing data indicate that, at extremely low depths, some De Novo assemblers, such as Flye and Canu, fail to obtain near-optimal solutions. Comparisons with the ground truth reveal that these assemblers do not achieve complete reconstruction in terms of both length and accuracy. For instance, at a coverage of 15x, Flye shows a high edit distance of 315, and Canu has a high edit distance of 551. These errors considerably compromise the accuracy of the assembly, adversely impacting subsequent tasks, such as protein structure prediction [34], etc.

To address these inaccuracies, the next essential step is to polish the assembled sequences to enhance their accuracy. A common approach is to use hybrid methods that integrate high-quality short reads, such as those from Illumina, with flawed assemblies. Polishing tools such as Pilon [18], which utilizes a mapping-based method, align these short reads to the assembly and apply a Bayesian model to determine the most accurate sequence by considering base quality scores and error frequencies. Another tool, NextPolish [19], similarly aligns short reads and uses an iterative process to correct errors in small regions of the assembly.

To evaluate the genome polishing performance of PVGA against NextPolish and Pilon, we conduct experiments to polish the assembly results generated by the PVGA and De Novo assembler Canu in previous experiments, respectively. The first involves a 9,706 bp HIV-1 genome assembled by PVGA from 15× coverage Nanopore reads (2 kb simulated read length, see Table 3), while the second consists of a 29,493 bp SARS-CoV-2 genome generated by Canu using 50× coverage long reads (2 kb average length, Figure 3).

We simulate Illumina short reads via Badread by setting parameter qscores as ideal (read length range 200-300 bp), with coverage parameters specifically calibrated: 15× for HIV-1 and 25× for SARS-CoV-2. The results in Table 9 demonstrate that PVGA outperforms NextPolish and Pilon in terms of polishing.

Table 9. Polished results of 15x coverage HIV-1 simulation data and 25x coverage SARS-CoV-2 simulation data

Virus	Tool	Genome fraction	Genome length	Mismatch	Indels	Indel length	Edit distance
HIV-1 (Length: 9713bp)	NextPolish	99.969	9722	2	12	12	17
	Pilon	99.969	9707	0	3	3	6
	**PVGA**	**100**	**9710**	**0**	**0**	**0**	**3**
SARS-CoV-2 (Length: 29646bp)	NextPolish	99.906	29636	2	26	28	58
	Pilon	99.906	29555	0	63	63	91
	**PVGA**	**99.906**	**29619**	**0**	**3**	**3**	**31**

Convergence performance across different backbones

In this subsection, we investigate how the choice of backbone influences the resulting genome assembly. We simulate the HIV-1 JRCSF strain nanopore reads with an average read length of 4 kb and coverage of 30x for the assembly. The error rate of simulation JRCSF reads is 5%. To evaluate the impact of different backbone sequences, we use four well-characterized HIV-1 reference genomes, including strains 89.6, HXB2, NL43, and YU2 as backbones. In addition, we also apply the De Novo assembly tool Flye [9] on our simulated reads and the obtained genome sequence is also used as the backbone. The Flye-derived backbone has an edit distance of 20 from the ground-truth genome. The similarities between the JRCSF ground truth genome and the various backbones are as follows: 89.6 (91.45%), HXB2 (92.78%), NL43 (92.55%), YU2 (92.83%) and Flye-derived assembly result (99.1%). Here the similarity between the two sequences is 1 - edit distance over the length of the ground truth genome. The similarities between the five different HIV-1 strain genome sequences are shown in Figure 4. The results are shown in Table 10.

	JRCSF	89.6	HXB2	NL43	YU2
JRCSF	**100.00**	91.06	91.98	91.77	92.41
89.6	91.06	**100.00**	93.51	93.05	93.10
HXB2	91.98	93.51	**100.00**	97.32	94.62
NL43	91.77	93.05	97.32	**100.00**	94.32
YU2	92.41	93.10	94.62	94.32	**100.00**

Figure 4. Pairwise similarity matrix of five HIV-1 Strains

Table 10. Results on simulated Nanopore HIV-1 JRCSF Strain reads with 5% error rate on different HIV-1 Strain backbones

Virus	Backbone Genome	Genome fraction	Genome length	Mismatch	Indels	Indel length	Edit distance
JRCSF (Length: 9540bp)	Flye-derived backbone	100	9540	0	0	0	0
	89.6	99.99	9539	0	0	0	1
	HXB2	99.99	9539	0	0	0	1
	NL43	99.99	9539	0	0	0	1
	YU2	99.99	9539	0	0	0	1

The similarities between the JRCSF ground truth genome and the four backbones are as follows: 89.6 (91.45%), HXB2 (92.78%), NL43 (92.55%), YU2 (92.83%), and the Flye assembly genome (99.1%).

From Table 10, we can see that for the backbones derived from 4 different HIV-1 strains, PVGA can obtain the same genome sequence with edit distance 1, and no indel or mismatch. In fact, there is just one missing base at the end of the obtained genome sequence. For the backbone generated by the De Novo assembler Flye, PVGA eliminates the 20-edit-distance discrepancy introduced by the De Novo approach, ultimately recovering the genome sequence identical to the ground truth genome.

To further investigate how the degree of divergence between the backbone and the ground truth impacts assembly results, we modify the HIV-1 JRCSF genome and randomly replace 15%, 20%, 25%, and 30% of the bases in the HIV-1 JRCSF genome to create backbones with different divergence. For the simulation reads, we maintain a coverage of 30x and an average read length of 4 kb.

Table 11. Results on simulated Nanopore HIV-1 JRCSF Strain reads with different backbones

Virus	Backbone similarity	Genome fraction	Genome length	Mismatch	Indels	Indel length	Edit distance
JRCSF (Length: 9540bp)	85%	99.948	9535	0	0	0	5
	80%	99.937	9533	0	0	0	7
	75%	99.843	9525	0	0	0	15
	70%	99.644	9506	0	0	0	34

As shown in Table 11, with the increase of diversity between the backbone and the ground truth genome, our method can still obtain good results in terms of mismatch, indels, while the edit distance increases and the obtained genome length decreases. Basically, the obtained genome missed some bases at the two ends, but can still perfectly match the ground truth genome in the middle. The reason is that very few reads completely cover the ends, and with the increase of backbone diversity, the few reads covering the ends have a higher possibility of being aligned to the wrong places when constructing the alignment graph.

Evaluation of computing resource usage

We evaluate the CPU runtime and memory usage of PVGA in comparison with several widely used assemblers. The datasets used in the experiments included HIV-1, Measles, and SARS-CoV-2, with sequencing coverages of 50×, 100×, and 200×, and respective genome lengths of approximately 10 kb, 20 kb, and 30 kb. Notably, Accuvir [15] exhibited a runtime exceeding 30 minutes and was therefore excluded from subsequent performance figures. As illustrated in Figure 5, PVGA achieves runtime performance comparable to other mainstream assemblers, though it is not always the best in all scenarios.

Canu and Flye consistently demonstrate the longest runtimes. For Canu, this is primarily due to the computationally intensive processes of read error correction and graph simplification, while Flye's substantial runtime stems from constructing and optimizing the De Bruijn assembly graph. As sequencing coverage increases, the runtime for nearly all assemblers grows proportionally. Interestingly, even with the iterative mechanism, PVGA exhibits lower runtime compared to other assemblers like Canu and Flye. The efficiency gained through iteration primarily stems from the rapid convergence of the process. In the initial iteration, the generated result is already close to the ground truth, providing a more refined backbone for constructing the alignment graph in the next iteration. As the backbone becomes increasingly accurate, subsequent iterations achieve more precise alignments, and the dynamic programming algorithm further optimizes the assembly. This iterative refinement continues until the backbone and the assembled result become identical, ensuring that the assembly process reaches a stable and accurate configuration in a relatively short time.

In terms of memory consumption, we monitor the maximum memory usage during the assembly process across different assemblers. As shown in Figure 6, assemblers relying on alignment graph construction tend to require more memory such as PVGA and PBDAG-Con. This is because PVGA employs a more complex global graph processing approach, which necessitates storing and manipulating a large amount of graph data, leading to higher memory consumption. In contrast, Canu and Flye typically break the input data into smaller chunks, which reduces memory usage and facilitates multi-threaded optimization. However, given the relatively small size of viral genomes compared to those of other species, PVGA's memory consumption remains within a reasonable range for viral genome assembly. All the computing resource experiments are conducted on an Apple M2 chip for evaluation.

Conclusion

PVGA is a powerful virus-focused assembler that does both assembly and polishing. For virus genomes, small changes will lead to huge differences in terms of viral function and pathogenicity. Thus, for virus-focused assemblers, high-accuracy results are crucial. Our approach heavily depends on the input reads as evidence to produce the reported genome. It first adopts a reference genome to start with. We then align all the reads against the reference genome to get an alignment graph. After that, we use a dynamic programming algorithm to compute a path with the maximum weight of edges supported by reads. Most importantly, the obtained path is used as the new reference genome and the process is repeated until no further improvement is possible.

The proposed framework demonstrates robust compatibility with diverse sequencing platforms, achieving nucleotide-level accuracy for both long-read (Nanopore / PacBio) and short-read (Illumina) data modalities. Experiments show that PVGA always outperforms popular existing programs in various cases. In particular, simulated Nanopore datasets show that our method can correctly report the true genomes with 0 mismatch and 0 indels.

Availability of source code and requirements

- Project name: PVGA
- Project home page:
  - github: `https://github.com/SoSongzhi/PVGA`
  - workflow DOIs: 10.48546/workflowhub.workflow.1305.1[35]
  - biotoolsID: PVGA
  - SciCrunch.org databases RRID: SCR_026410
- Operating system(s): Platform independent
- Programming language: Python
- Other requirements: numpy, pysam 0.22.0 or higher, pandas 1.5.2 or higher, Bio 1.7.1 or higher, biopython 1.83 or higher, consensus 1.0.5 or higher, networkx 3.1 or higher, pandas 1.5.2 or higher, python 3.10
- Biotools: QUAST 5.3.0, Badread 0.4.1
- License: MIT License

Additional Files

Supplementary Table 1. Results on simulated PacBio HIV-1 datasets with a 5% error rate and an average read length of 2kb.
Supplementary Table 2. Results on simulated PacBio HIV-1 datasets with a 5% error rate and an average read length of 4kb.
Supplementary Table 3. Results on simulated PacBio HIV-1 datasets with a 5% error rate and an average read length of 6kb.
Supplementary Table 4. Results on simulated PacBio SARS-CoV-2 datasets with 5% error rate and an average read length of 2kb.
Supplementary Table 5. Results on simulated PacBio SARS-CoV-2 datasets with 5% error rate and an average read length of 4kb.
Supplementary Table 6. Results on simulated PacBio SARS-CoV-2 datasets with 5% error rate and an average read length of 8kb.

Data availability

HIV-1:

- 89.6 Strain complete genome is available in the NCBI database (GenBank: U39362.2)
- HXB2 Strain complete genome is available in the NCBI database (GenBank: K03455.1)
- haplotype benchmarking dataset which cantain mixed PacBio reads from five strains (HXB2, 89.6, JR-CSF, NL4-3, YU-2) [22]

SARS-CoV-2:

- SARS-CoV-2 isolate Wuhan-Hu-1 is available in the NCBI database (NCBI Reference Sequence: NC_045512.2)
- SARS-CoV-2 isolate INEI121916 is available int the NCBI database (GenBank: OZ072292.1)

Norovirus:

- Nanopore sequencing reads are available in the NCBI database (Run: SRX10330013)

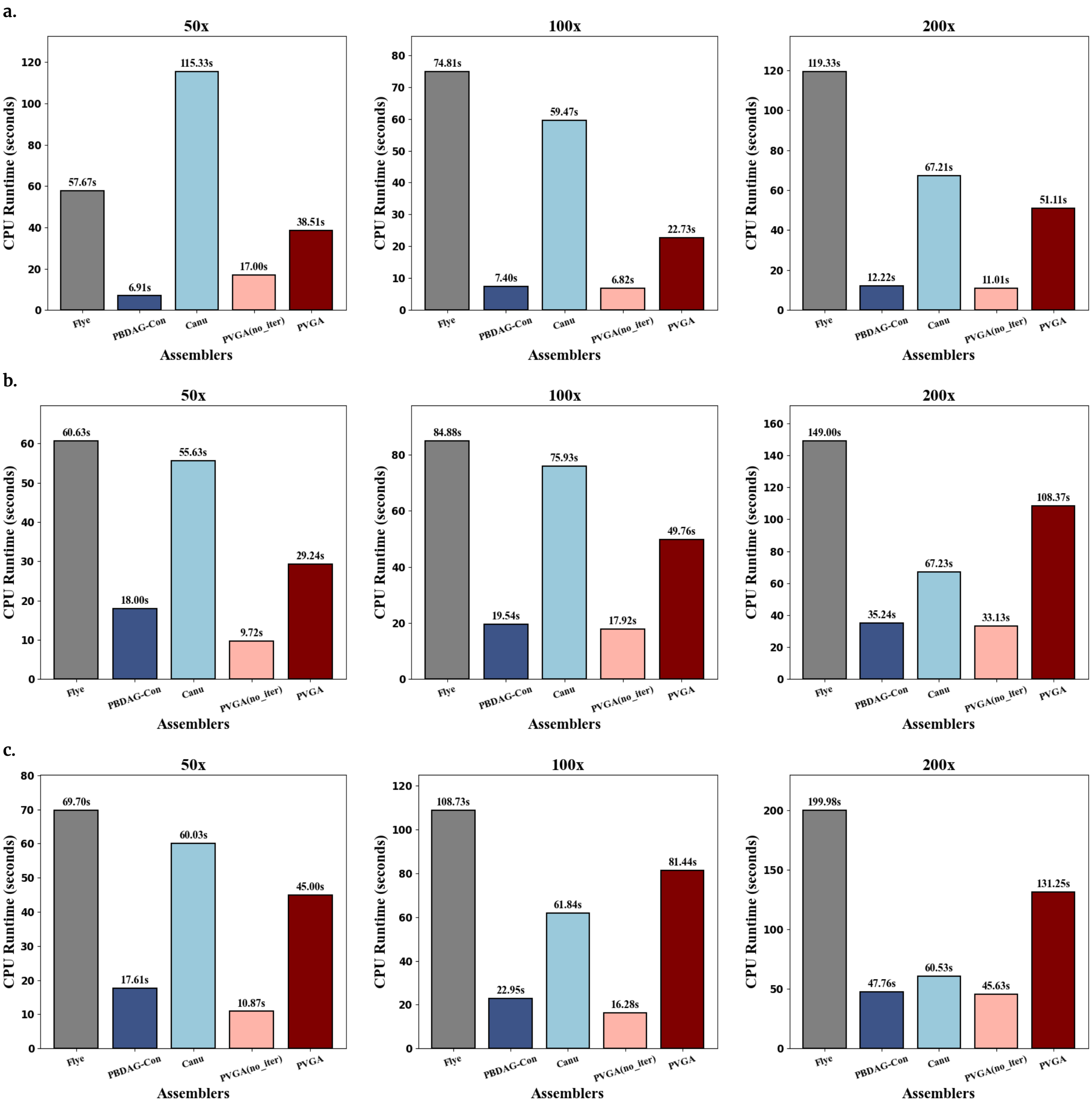

Figure 5. Comparison of CPU times for the five tools on the three datasets of 50x, 100x, 200x coverage respectively, **a.** HIV-1 virus 89.6 Strain, **b.** Measles Virus, **c.** SARS-CoV-2

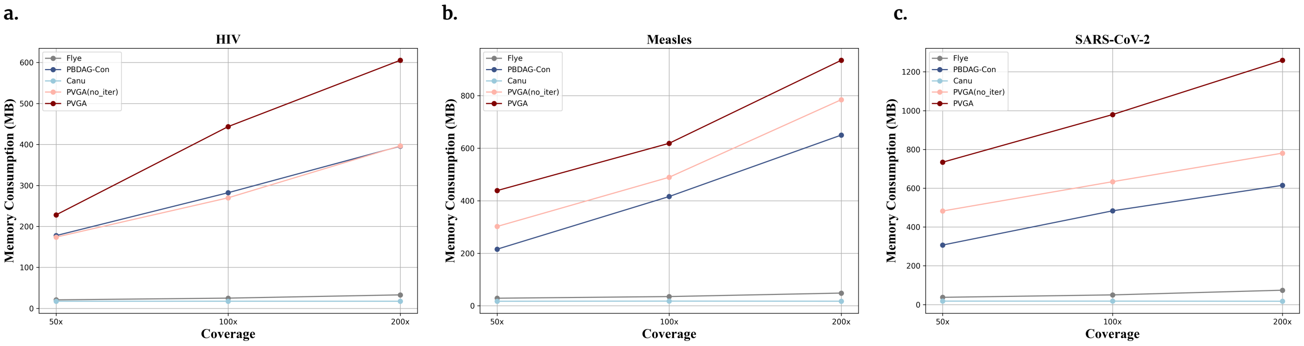

Figure 6. Comparison of maximum memory consumption during the runtime across three datasets with 50×, 100×, and 200× coverage: **(a)** HIV-1 89.6 strain, **(b)** Measles virus, **(c)** SARS-CoV-2.

- Illumina sequencing reads are available in the NCBI database (Run: RR13951201, Run: SRR13951221 and Run: SRR1395119)
- The complete genome of Norovirus GII is available in the NCBI database (GenBank: GenBank: MW661264.1, MW661284.1, GenBank: MW661248.1 and GenBank: MW661250.1)

Ebola virus:

- Ebola virus (EBOV-May) Mayinga strain complete genome is available in the NCBI database (GenBank: AF086833.2)
- Ebola virus/M.fascicularis-wt/GAB/2001/untreated-CCL053D9, complete genome is available in the NCBI database (GenBank: KY786027.1)

Measles virus:

- Measles complete genome is available in the NCBI database (NCBI Reference Sequence: NC_001498.1)
- Measles virus genotype A transgenic strain vac2(GFP)H genome is available in the NCBI database (GenBank: MH144178.1)

Declarations

List of abbreviations

- EBOV: Ebola virus
- GP: glycoprotein
- HGAP: Hierarchical Genome-assembly Process
- HIV: Human Immunodeficiency Virus
- NBCI: National Center for Biotechnology Information (NCBI).
- NP: Nucleoprotein
- NGS: Next-Generation Sequencing
- SARS-CoV-2: Severe Acute Respiratory Syndrome Coronavirus 2
- SMRT: Single Molecule Real-Time
- TGS: Third-Generation Sequencing

Consent for publication

Not applicable.

Competing Interests

No competing interests.

Funding

This work is fully supported by funds from the National Science Foundation (NSF: 61972329) and GRF grants for Hong Kong Special Administrative Region, P. R. China (CityU 11218821).

Author's Contributions

L.W. conceived the original idea and supervised the entire work. Z.S. continuously expanded the work, conceptualized the iterative process, developed the entire PVGA algorithm, and was the primary contributor to the manuscript. D.C. provided suggestions during the experimental phase and assisted in locating real data for testing. Y.S. contributed valuable insights for the experimental design. All authors participated in reviewing and improving the manuscript.

Acknowledgements

This work is fully supported by funds from the National Science Foundation (NSF: 61972329) and GRF grants for Hong Kong Special Administrative Region, P. R. China (CityU 11218821).

Authors' information

- **Zhi Song** is a Ph.D. candidate in the Department of Computer Science at City University of Hong Kong. His research interests include algorithms, machine learning, computational biology, and bioinformatics.
- **Dehan Cai** is a Ph.D. candidate in the Department of Electrical Engineering at City University of Hong Kong. His main research interests include sequence analysis in bioinformatics and computational biology, focusing on developing tools for microbial analysis using machine learning and statistical methods.
- **Yanni Sun** is a professor in the Department of Electrical Engineering at City University of Hong Kong. Before relocating to Hong Kong, she was an Associate Professor in the Department of Computer Science and Engineering at Michigan State University, USA. She received both her BS and MS degrees from Xi'an Jiao Tong University (China) in Computer Science and her PhD in Computer Science and Engineering from Washington University in Saint Louis, USA. Her research interests include bioinformatics and computational biology, particularly sequence analysis, machine learning, data mining for next-generation sequencing data, metagenomics, protein domain annotation, and noncoding RNA annotation. She received the NSF CAREER Award in 2010.
- **Lusheng Wang** obtained his Ph.D. in Computer Engineering from McMaster University in 1995. He is currently a professor in the Department of Computer Science at City University of Hong Kong. His research interests include algorithms, computational biology, and bioinformatics.

References

1. Hofacker IL, Stadler PF, Stocsits RR. Conserved RNA secondary structures in viral genomes: a survey. Bioinformatics 2004;20(10):1495–1499.
2. Harvey WT, Carabelli AM, Jackson B, Gupta RK, Thomson EC, Harrison EM, et al. SARS-CoV-2 variants, spike mutations and immune escape. Nature Reviews Microbiology 2021;19(7):409–424.
3. Jain M, Koren S, Miga KH, Quick J, Rand AC, Sasani TA, et al. Nanopore sequencing and assembly of a human genome with ultra-long reads. Nature biotechnology 2018;36(4):338–345.
4. Eid J, Fehr A, Gray J, Luong K, Lyle J, Otto G, et al. Real-time DNA sequencing from single polymerase molecules. Science 2009;323(5910):133–138.
5. Wenger AM, Peluso P, Rowell WJ, Chang PC, Hall RJ, Concepcion GT, et al. Accurate circular consensus long-read sequencing improves variant detection and assembly of a human genome. Nature biotechnology 2019;37(10):1155–1162.
6. Zerbino DR, Birney E. Velvet: algorithms for de novo short read assembly using de Bruijn graphs. Genome research 2008;18(5):821–829.
7. Simpson JT, Wong K, Jackman SD, Schein JE, Jones SJ, Birol I. ABySS: a parallel assembler for short read sequence data. Genome research 2009;19(6):1117–1123.
8. Bankevich A, Nurk S, Antipov D, Gurevich AA, Dvorkin M, Kulikov AS, et al. SPAdes: a new genome assembly algorithm and its applications to single-cell sequencing. Journal of computational biology 2012;19(5):455–477.
9. Kolmogorov M, Yuan J, Lin Y, Pevzner PA. Assembly of long, error-prone reads using repeat graphs. Nature biotechnology 2019;37(5):540–546.
10. Koren S, Walenz BP, Berlin K, Miller JR, Bergman NH, Phillippy AM. Canu: scalable and accurate long-read assembly via adap-

tive k-mer weighting and repeat separation. Genome research 2017;27(5):722–736.
11. Liu J, Yu T, Mu Z, Li G. TransLiG: a de novo transcriptome assembler that uses line graph iteration. Genome biology 2019;20:1–9.
12. Mu JC, Jiang H, Kiani A, Mohiyuddin M, Bani Asadi N, Wong WH. Fast and accurate read alignment for resequencing. Bioinformatics 2012;28(18):2366–2373.
13. Li H, Ruan J, Durbin R. Maq: Mapping and assembly with qualities. Version 06 2008;3:508.
14. Grubaugh ND, Gangavarapu K, Quick J, Matteson NL, De Jesus JG, Main BJ, et al. An amplicon-based sequencing framework for accurately measuring intrahost virus diversity using PrimalSeq and iVar. Genome biology 2019;20:1–19.
15. Yu R, Cai D, Sun Y. AccuVIR: an ACCUrate VIRal genome assembly tool for third-generation sequencing data. Bioinformatics 2023;39(1):btac827.
16. Li H. A statistical framework for SNP calling, mutation discovery, association mapping and population genetical parameter estimation from sequencing data. Bioinformatics 2011;27(21):2987–2993.
17. Hunt M, Gall A, Ong SH, Brener J, Ferns B, Goulder P, et al. IVA: accurate de novo assembly of RNA virus genomes. Bioinformatics 2015;31(14):2374–2376.
18. Walker BJ, Abeel T, Shea T, Priest M, Abouelliel A, Sakthikumar S, et al. Pilon: an integrated tool for comprehensive microbial variant detection and genome assembly improvement. PloS one 2014;9(11):e112963.
19. Hu J, Fan J, Sun Z, Liu S. NextPolish: a fast and efficient genome polishing tool for long-read assembly. Bioinformatics 2020;36(7):2253–2255.
20. Chin CS, Alexander DH, Marks P, Klammer AA, Drake J, Heiner C, et al. Nonhybrid, finished microbial genome assemblies from long-read SMRT sequencing data. Nature methods 2013;10(6):563–569.
21. Wick RR. Badread: simulation of error-prone long reads. Journal of Open Source Software 2019;4(36):1316.
22. Di Giallonardo F, Töpfer A, Rey M, Prabhakaran S, et al., 5-virus-mix: Benchmarking data sets for haplotype reconstruction methods, sequenced with Illumina MiSeq, 454/Roche GSJunior, and Pacific Biosciences. Software Heritage; 2014. https://archive.softwareheritage.org/swh:1:snp:7396004d2b720de6dc88e14164955aea819766aa.
23. Li H. Minimap2: pairwise alignment for nucleotide sequences. Bioinformatics 2018;34(18):3094–3100.
24. Danecek P, Bonfield JK, Liddle J, Marshall J, Ohan V, Pollard MO, et al. Twelve years of SAMtools and BCFtools. Gigascience 2021;10(2):giab008.
25. Flint A, Reaume S, Harlow J, Hoover E, Weedmark K, Nasheri N. Genomic analysis of human noroviruses using combined Illumina–Nanopore data. Virus Evolution 2021;7(2):veab079.
26. Bukreyev A, Volchkov V, Blinov V, Netesov S. The VP35 and VP40 proteins of filoviruses: homology between Marburg and Ebola viruses. FEBS letters 1993;322(1):41–46.
27. Volchkov VE, Volchkova VA, Slenczka W, Klenk HD, Feldmann H. Release of viral glycoproteins during Ebola virus infection. Virology 1998;245(1):110–119.
28. Guedj J, Piorkowski G, Jacquot F, Madelain V, Nguyen THT, Rodallec A, et al. Antiviral efficacy of favipiravir against Ebola virus: A translational study in cynomolgus macaques. PLoS medicine 2018;15(3):e1002535.
29. Del Valle JR, Devaux P, Hodge G, Wegner NJ, McChesney MB, Cattaneo R. A vectored measles virus induces hepatitis B surface antigen antibodies while protecting macaques against measles virus challenge. Journal of virology 2007;81(19):10597–10605.
30. Pfaller CK, Mastorakos GM, Matchett WE, Ma X, Samuel CE, Cattaneo R. Measles virus defective interfering RNAs are generated frequently and early in the absence of C protein and can be destabilized by adenosine deaminase acting on RNA-1-like hypermutations. Journal of virology 2015;89(15):7735–7747.
31. Lee JY, Kong M, Oh J, Lim J, Chung SH, Kim JM, et al. Comparative evaluation of Nanopore polishing tools for microbial genome assembly and polishing strategies for downstream analysis. Scientific Reports 2021;11(1):20740.
32. Ip CL, Loose M, Tyson JR, de Cesare M, Brown BL, Jain M, et al. MinION Analysis and Reference Consortium: Phase 1 data release and analysis. F1000Research 2015;4.
33. Delahaye C, Nicolas J. Sequencing DNA with nanopores: Troubles and biases. PloS one 2021;16(10):e0257521.
34. Watson M, Warr A. Errors in long-read assemblies can critically affect protein prediction. Nature biotechnology 2019;37(2):124–126.
35. Song Z, PVGA. WorkflowHub; 2025. https://doi.org/10.48546/WORKFLOWHUB.WORKFLOW.1305.1.

Supplementary

Click here to access/download
Supplementary Material
supplementary.pdf

 Click here to access/download;Personal Cover;pvga_response.pdf

Lusheng Wang (the corresponding author)
Dept. of Computer Science
City University of Hong Kong
Hong Kong (SAR)
cswangl@cityu.edu.hk

Dear Editor and Reviewers,

We would like to thank you for your careful reading of our paper and the valuable comments. We have addressed the comments of all reviewers, with our responses in blue.

Response to Reviewer 1's comments

Major review points:
(1) The installation instructions
sudo apt install blasr
conda create -n pvga python=3.10
pip install pvga
still do not work on my side without further ado and I cannot verify the tool's functionality. To the best of my knowledge, not every operating system's Aptitude package manager contains blasr. Further, users without sudo privileges (such as HPC users) cannot use sudo commands. blasr can easily by installed via bioconda. I strongly recommend
conda create -n pvga -c bioconda python=3.10 blasr
as an installation instruction to circumvent installing blasr from outside the conda environment. Next, a simple
conda activate pvga
is missing in the instructions. Finally, I found myself in a recursion of installing dependencies when precisely following installation the GitHub instructions.
$ pvga
Traceback (most recent call last):
File "/<foo>/envs/pvga/bin/pvga", line 5, in <module>
from pvga import main
File "/<foo>/envs/pvga/lib/python3.10/site-packages/pvga/__init__.py", line 1, in <module>
from .pvga import main
File "/<foo>/envs/pvga/lib/python3.10/site-packages/pvga/pvga.py", line 1, in <module>
import networkx as nx
ModuleNotFoundError: No module named 'networkx'
I started installing networkx but then numpy etc followed so I guess it is a general issue. Please make sure you have an up-to-date installation instruction that works out-of-the-box without sudo privileges.

Thank you for your advice. In the initial response letter, we mentioned that there is a conda-based installation method. For pip installations, users need to install the dependencies listed in the requirements.txt file using the command *pip install -r requirements.txt*. We have now incorporated this documentation into the README file. Additionally, we have also added instructions to the README regarding how users can download blasr through bioconda.

(2) The input preparation “For paired-end reads” on GitHub is not clear to me. You recommend bbmerge.sh to merge forward (R1) and reverse (R2) reads and then to concatenate the result with unmerged reads. Hence, the all.fastq will result in three consecutive sets of sequences: contigs, unpaired R1, and unpaired R2. This is not preserving paired-end information in the fastq file. And according to the methods section of PVGA there is no need to even do so. The contigs (merged.fastq) might result in longer alignments but there is no preservation of the read pairing. Technically, to just have a fitting input format you could simply concatenate the R1 and R2 files.

Thank you for your feedback. Since PVGA accepts single-end reads as input, paired-end reads must be converted into a single-read format. The simplest way to do this is to concatenate the two paired-end read files (R1 and R2) into one file. However, this method does not use the pairing information during alignment. In many cases, paired-end reads have overlapping regions. By merging each read pair into a single, longer read, it is possible to keep this pairing information in the downstream alignment. This is exactly what BBMerge does. Note that BBMerge does not generate contigs like traditional assembly tools. Instead, it merges two overlapping paired-end reads into a longer single-end read. This allows tools that only support single-end input to still benefit from the paired-end data. Although the difference in PVGA performance between merged and unmerged reads is usually small, we recommend merging as a good practice when working with paired-end data. Longer reads often lead to better alignment. We emphasize that this is an optional optimization step, as noted on our GitHub page. For users who prefer not to merge reads, simply concatenating the R1 and R2 files is still a valid option. Our GitHub documentation now includes detailed instructions for both methods.

(3) Internal cross-referencing is still wrong. On page 8, referenced Figure 5 should likely be Figure 4. On page 9, referenced Figure 5 should likely be Figure 6.

Thanks for your advice. We have already corrected the reference.

Minor review points:

(4) From what can tell from the code there is still no software versioning. This would be very helpful for packaging/containerizing PVGA, reproducibility, and workflows around PVGA.

Thanks for your advice. Users can check the package version through bioconda. Also we have added version information in GitHub.

(5) There are still language errors. E.g.:
"As the basic structure of many virus genomes is highly conserved." – in the Abstract.
This is a clause. The main clause is missing I guess.
"Basically, the obtained genome missed some bases at the two ends, but can still perfectly match the ground tree genome in the middle." – in the paragraph Convergence performance across different backbones. I guess “tree” should be
“truth”.

Thanks for your advice. We have fixed these typos.

(6) Supplementary Material 1 claims assemblies are better from ONT data than PacBio. Is this true? This is quite surprising since PacBio data should be of higher quality.

Thanks for your advice. We have already explained the reason why badread simulated PacBio reads's quality is not good as the ONT reads in the original response letter as follows:

"We analyzed the simulated PacBio and Nanopore datasets using Heng Li's alignment-based identity metrics. While the overall read identities were comparable between the two datasets (both approximating the preset identity thresholds in Badread), we observed distinct error profile distributions as documented in Badread's error models (https://github.com/rrwick/Badread/wiki/Error-models). Specifically, PacBio-like simulated reads produced fewer completely correct reads compared to Nanopore-like simulated reads. (see " PacBio2016 model" section in documentation). This indicates that error-containing reads are more likely to occur, leading to increased alignment errors or misalignments during assembly. We have added relavite illustration in the supplemantary file."

(7) Are the edge weights really correct in Figure 1.c? I think the edge weights at the beginning of the backbone do not match the reads.

Thanks for your kindly advise. We have already corrected the figures.
