# Supplementary material for: PVGA: a precise viral genome assembler using an iterative alignment graph
Source: Gigascience. 2025 Jun 24;14:giaf063. doi: 10.1093/gigascience/giaf063 (PMC12206156; doi:10.1093/gigascience/giaf063)
Supplement: giaf063_Authors_Response_To_Reviewer_Comments_Original_Submission [file giaf063_authors_response_to_reviewer_comments_original_submission.pdf]

Author's Response To Reviewer Comments

Dear Editor and Reviewers,
We would like to thank you for your careful reading of our paper and the valuable comments. We have addressed the comments of all reviewers, with their comments in black and our responses in blue. The revised figures and descriptions were added to the main manuscript and the supplementary file.

Before we provide the detailed responses, we would like to summarize the major improvements.

1. The tool is registered in bio.tools (RRID:SCR_026410) and its computational workflows are permanently archived at WorkflowHub (DOI:10.48546/workflowhub.workflow.1305.1). To ensure broad accessibility, PVGA supports both PyPI installation (pip install pvga) and Bioconda deployment (conda install -c bioconda pvga).
2. We have added our detail description of our parameters in PVGA at our Github repository https://github.com/SoSongzhi/PVGA.
3. We have expanded our experiments to test how backbone choice affects assembly accuracy. We use real HIV strains (89.6, HXB2, NL43, YU2) as backbones for assembling the JRCSF genome, we also created computer-modified JRCSF backbones with increasing differences (15%, 20%, 25%, 30%) through controlled random mutations. Results in Tables 10-11 show how these backbone variations impact assembly quality.
See subsection “Convergence performance across different backbones”

We hope that the following point-to-point responses will be clear enough to convey our ideas and address the comments of the reviewers.

Response to Reviewer 1’s comments

Major review points:
(1) Please add one proof of concept analysis for PVGA’s algorithmic convergence starting from different backbone genomes. A concern of mine is that the algorithm might converges towards certain alleles or strains when different backbone genomes are used. This can be tested with just three – or more – different strains of the same species. E.g. from three distinct strains {sample= X, backbones= {Y, Z}} you take reads from X and assemble them with Y and Z as backbones. Then check whether both approaches still assemble X accurately. Ideally and to be convincing, the genomes Y and Z in the set of backbones should be reasonably distant in sequence similarity and no closely related recombinants.

This is one of the major improvements, which we have responded as item 3 in major improvements. Basically, we add subsection “Convergence performance across different backbones” to address the issue. See Page. 8.

(2) I tried using the software and run it on some very simple SARS-CoV-2 sequence data. I noticed several issues that made it difficult to install and impractical to use the software yet. Please address the following issues and consider suggestions regarding the software:

a. [Issue]
The installation instructions do not work without further ado.
conda create pvga --name pvga python==3.10
This line has pvga as a positional argument. This does not work in conda. Further, if the user makes it until
pip install -r requirements.txt
then the pip dependency “python==3.10” collides and breaks because of the python instance previously installed via conda.

We sincerely appreciate this critical technical feedback. Users now can download PVGA with follow commands:

```
conda create --name pvga python=3.10
conda activate pvga
conda install -c bioconda pvga
```

b. [Issue]
Once I got through the installation section I couldn't get PVGA to run. I provided a backbone genome (-b) and a set of ONT reads (-r). Now, the software informs me that a number if iterations (-n) is required as well as a ground truth sequence (-gt). The paper in contrast says that the iterative process simply runs until no further improvement is made in an iteration. Hence, I expect this parameter not to be required. If even specified, I'd understand this parameter as an upper bound. Also, in practical applications there is no known ground truth. Why is this parameter required?

We appreciate this critical technical feedback. The -n parameter was implemented to evaluate the influence of iterations on assembly results. For instance, setting -n 1 allows us to obtain results without iteration, as demonstrated in the benchmarking figure in the paper. Additionally, the -gt parameter introduced in the previous version was to input ground truth for alignment tool QUAST to evaluate the performance of the assembly. In the user-facing version, based on your recommendation, we have removed the -gt parameter and made the iteration number -n optional, with a default value as the upper bound. Users can still specify the iteration number as needed.

c. [Issue]
Hypothetically, if I could make PVGA run I wouldn't know how to provide paired-end short-read sequencing data. Since there is only one read parameter (-r) do I need to provide short-read paired-end data as interleaved fastq file? Please improve documentation.

Thank you for this valuable advice. While PVGA takes single reads as input, it can also process paired-end reads. Users with paired-end data can merge their FASTQ files using BBMap while preserving paired-end information. The merged FASTQ file, where overlapping read pairs are combined into longer single reads, can then be used as input for PVGA assembly. By combining these longer single reads with the unmerged reads from both FASTQ files, PVGA can effectively handle paired-end data. This approach maintains compatibility with PVGA's single-read input design while ensuring efficient processing of paired-end datasets. We have included this explanation along with the BBMap command lines in our README.

d. [Improvement suggestion]
For such a simple installation instruction you utilized two package management systems, conda and pip. Everything necessary to run PVGA can be installed via one concise conda environment. I suggest such an approach would be greatly appreciated by the users.
e. [Improvement suggestion]
Since every dependency can be installed via conda it would be of great help and simplicity for the user if PVGA itself could be installed via (bio)conda -https://bioconda.github.io/. This is a user-friendly way to distribute your bioinformatics software.

Thanks for the valuable suggestions regarding package management. PVGA is now officially available on both Bioconda (conda install -c bioconda pvga) and PyPI (pip install pvga), providing users with flexible installation options to suit their preferred workflows.

f.[Improvement suggestion]
It is good software development practice that the software complies with semantic versioning - https://semver.org/. Please display the version on the help page (--help).

The shield on your Github README can then automatically use this implemented
version.

Thanks for this valuable improvement suggestion. We have updated PVGA to comply with semantic versioning as per semver.org. The version number is now prominently displayed in the help page (via --help) and integrated into the GitHub README using dynamic shields from shields.io. We will maintain strict adherence to semantic versioning in all future releases.
g. [Improvement suggestion]
Short parameter of two letters is rather confusing and unnecessary for PVGA's short list of parameters. You might like to have a look at Heng Li's CLI best practice - https://lh3.github.io/2021/07/04/designing-command-line-interfaces.

Thanks for this valuable improvement suggestion. we have revised PVGA's command-line interface to use single-letter short parameters exclusively (e.g., -r for reads, -b for backbone, -o for output directory), accompanied by clear and detailed descriptions in the help page (--help).

(1) Please improve some minor language errors:
a. [Intro] (Article) "The virus genomes have relatively small size."

We totally agree with your suggestion. We have removed this sentense.

b. [Intro] (Article) "For example, several mutations in the spike protein gene of coronavirus can […]"

We totally agree with your suggestion. "For example, several mutations in the spike protein gene of coronavirus can […]" has been modified to "For example, genetic mutations in the coronavirus genome that alter the spike protein can affect its ability to interact with host cells, thereby affecting transmissibility and disease severity." See the bottom of the left column on Page 1.

c. [Intro] (Wording) "[…] it is well-known that NGS struggles with assembling repetitive regions in the genome" → NGS itself is not performing assembly; better something like "difficult to assemble repetitive regions using NGS data"

We totally agree with your suggestion. "[…] it is well-known that NGS struggles with assembling repetitive regions in the genome" has been modified to "However, due to the short read lengths and the presence of repetitive regions, it is often challenging to assemble genomes accurately using NGS data." See the second paragraph of the Section "Introduction".

d. [Datasets] (Wording) "[…] as a target to generate the simulation reads." → "as a target and to generate simulated reads" or "as a target and to generate synthetic reads"

Thanks for your suggestion. "[…] as a target to generate the simulation reads." has been modified to "as a target and to generate simulation reads.". See the subsubsection "SAR-CoV-2" in the subsection "Datasets" See the bottom of the right column on Page 1.

e. [Datasets] (Typo) "We utilize two Measles virus strain." → plural "strains"

Thanks for your suggestion. "We utilize two Measles virus strain." has been modified to "We utilize two Measles virus strains." See the middle of the right column on Page 4.

f. [Evaluation on simulated data] (Wording) "[…] refinement process significantly enhances assembly accuracy." → I am against "significant" here since, in the scientific context, this usually means it's statistically tested which is rather difficult for these small numbers.

Thanks for your suggestion. The word "significant" has been removed.

g. I stop explicitly listing here but there are more errors, e.g. with the plural and relative clauses. Please

spellcheck in general.

(2) [Intro]
When you introduce reference-based assembly you cite tools like BWA, Bowtie and GATK. To the best of my knowledge, these tools are primarily read mapper and variant caller. Both read mapping and variant calling serve as a prior for reference-guided assembly but it is technically not the assembly process they are performing. Assembly, both de novo and reference-guided, should yield a consensus sequence or contigs as an output. Software like bcftools (https://samtools.github.io/bcftools/bcftools.html), iVar (https://github.com/andersen-lab/ivar) or AccuVir perform reference-guided assembly. Please improve the wording here or correct accordingly.

Thanks for pointing out the error. We have removed the description of these tools from the section on reference-based assembly. Instead, we now focus on tools specifically designed for reference-guided assembly. The comparison before and after modification is as follows:

Origin: “Famous reference-guided assembly methods include BWA, Bowtie2, GATK, Novoalign, and Maq.”

Revised: “Famous reference-guided assembly methods include Novoalign, Maq, iVar, Accuvir and bcftools.” See the middle of the left column of Page 2.

(3) [Intro]
Please comment on the shortcoming of previous approaches that led to the motivation to develop another reference-guided assembler.

Thank you for your valuable suggestion. We have addressed the shortcoming of previous approaches in the introduction by stating: “However, despite these advancements, current genome assembly tools still fail to achieve the requisite base-level accuracy for viral genome assembly.” See the middle of the left column of Page 2.

(4) [Methods – Alignment graph construction]
The process is mostly well defined and nicely visualized with Figure 1. However, I have several detail questions here that need to be addressed in the manuscript:
a. What is u'? I assume from the context that it is the new node after a merging f: vi, $si \to u'$ where vi and si had the same label and parent.

Yes, you are correct. u' is indeed the new node created after merging nodes vi and si, which share the same label and parent. To clarify this process, we have added an illustration showing how u' is derived, along with its predecessor node v as follows:

Origin: “The weight of the remaining edge (v, u') is updated to be the number of supporting reads.”
Revised: “When merging nodes into a new node u' with its predecessor v, the weight of the resulting edge (v, u') is updated to reflect the total number of supporting reads.” See the subsection “Alignment graph construction”

b. A very crucial detail is hidden behind the wording “If ri is matched with an identical letter sj in the alignment, then ri corresponds to the node in vj in Gb.” This matching is a complex problem itself. From your Figure 1c I can only hypothesize that some sort of sequence-to-graph alignment is involved. Please describe the matching in more detail.

Thanks for your advise. Our alignment graph methodology is primarily based on the paper "Nonhybrid, finished microbial genome assemblies from long-read SMRT sequencing data", which provides a detailed methodology for constructing the alignment graph. Specifically, we first align the reads to the backbone genome using BLASR software, then construct the graph. We focus more explicitly on the algorithmic steps for building the alignment graph following read-to-backbone genome alignment, and we have supplemented these details in the manuscript as follows：

"In the alignment, if ri is aligned to an identical letter sj in the backbone sequence, then ri corresponds to the existing node vj in the graph Gb. The weight of the edge (vj−1, vj) will be incremented by one, where vj−1 is the predecessor node of vj. If ri is aligned with a space or a letter sj not identical to ri, we will create a new node ui labeled with ri and add an edge (ui−1, ui) with weight 1, where ui−1 is the node corresponding to the previous letter ri−1. See Fig. 1(b)."
See the subsection "Alignment graph construction"

c. What happens in the backtracking if there is a numeric tie between the weights (alleles)? Can alleles accidentally swap in and out per iteration?

Thanks for your question. Based on our empirical observations, we have not encountered instances of numeric ties between allele weights. From an algorithmic perspective, if such a tie were to occur, our method would resolve it by selecting one of the alleles as the final result This selection process ensures deterministic output while maintaining assembly consistency.

(5) [Methods – Evaluation on simulated data]
Please state whether the competing methods (Canu, Flye, AccuVir, PBDAG-Con, Medaka) are de novo or reference-based methods.
Also, PEPPER-Margin is a quite popular pipeline for phased haplotype inference. To avoid adding more analyses here you can argue that you improve upon PEPPER-Margin via implication through AccuVir.

Thank you for your suggestion. Canu and Flye are De Novo methods, while Accuvir, PBDAG-Con, and Medaka are reference-based methods. We have added this description in the revised manuscript. In Accuvir, PEPPER and Margin were tested extensively on simulated data experiments, and the results showed that Accuvir and Medaka outperformed PEPPER-Margin in most cases. Since we used a similar approach to generate simulated data and given the superior performance of Accuvir and Medaka, we benchmarked PVGA only against Medaka and Accuvir, excluding the others from our analysis.

(6) [Methods – Evaluation on simulated data]
Have you left out an evaluation on simulated short-read data? If so, why?

Yes, we evaluated PVGA on real-world Illumina short-read datasets (Table 8), focusing on norovirus sequencing data (SRR13951201, SRR13951221, SRR1395119 with read lengths 301/201). This aligns with our methodology stated in the text: "To assess the effectiveness of the PVGA method with extensive short-read datasets, we employ actual Illumina sequencing data of the norovirus. […]" See the bottom of the right column of Page 2.

(7) [Methods – Evaluation on simulated data]
"Edit distance indicates a minimum number of operations required to transform the assembled genome into the reference sequence." → "Edit distance defines a minimum number of substitution and indel operations required to transform the assembled genome into the reference sequence."

Thank you for this insightful suggestion. We have revised the definition of edit distance in the Results section as recommended. See the middel part of the right column on the Page 4.

(8) [Methods – Evaluation on simulated data]
"To simulate real sequencing data, we used the Badread […]" → "To simulate sequencing data, we used the Badread […]"

Thank you for this insightful suggestion. We have deleted the word "real".

(9) [Methods – Evaluation on simulated data]
You applied different error models to Badread to simulate reads of different sequencing technologies but

used the same identity settings in both cases, i.e. for PacBio and ONT. Have
you investigated whether this leads to a noticeable difference in sequence similarity between
the simulated datasets? I.e. if the identity settings are the same for PacBio and ONT, what
difference can you observe across the simulated datasets? Please add a brief comparison/number. One
quick and easy solution would be to analyze the alignments of the
simulated reads with the perl one-liners by Heng Li (https://lh3.github.io/2018/11/25/on-the-definition-of-sequence-identity).

Thanks for your question. We analyzed the simulated PacBio and Nanopore datasets using Heng Li's alignment-based identity metrics. While the overall read identities were comparable between the two datasets (both approximating the preset identity thresholds in Badread), we observed distinct error profile distributions as documented in Badread's error models (https://github.com/rrwick/Badread/wiki/Error-models). Specifically, PacBio-like simulated reads produced fewer completely correct reads compared to Nanopore-like simulated reads. (see " PacBio2016 model" section in documentation). This indicates that error-containing reads are more likely to occur, leading to increased alignment errors or misalignments during assembly. We have added relavite illustration in the supplemantary file.

(10) [Table 1,2,3]
“length: 9713bp” → “genome length: 9713bp” Maybe rephrase the description. The first half
of the sentence suggests that HIV-1 would be a simulation.

Thank you for this insightful suggestion. We have revised the description as recommended.

(11) [Evaluation on poor sequencing conditions]
I believe this section provides an important and relevant analysis of a more error-prone
sequencing experiment. However, please consider adding the following adjustments:
a. Please state clearly how you increased the error-rate from an average of 5%(?) to now
10%. The former is a little bit hidden in the “identity settings” of the simulation setup.

Thank you for the constructive feedback. We have explicitly clarified the error rate modification protocol as follows:

“To simulate poor sequencing conditions, we configured the following parameters for evaluating assembler performance under suboptimal data quality: We apply a truncated normal distribution of basecall identity (range: 85–95%, mean: 90%, SD: 5%), resulting in an average read error rate of 10\% and an upper accuracy bound of 95%. And we assign uniform sequencing depth (30×) and average read length (4 kb) across all viral genomes (HIV, Measles, Ebola) to standardize suboptimal quality conditions. The results are shown as Table5.” See the subsection “Evaluation on poor sequencing conditions”.

b. You use [32] to reason the high error-rate. While historically true, this paper and its
data are a decade old and, in my humble experience, Oxford Nanopore Technologies
has done a massive leap in sequencing quality since. Consider adding that the poor
sequencing conditions setup reflects particularly poor sequencing runs, usage of
outdated flowcells, or assembly of elder sequencing data. All those are common and
perfectly realistic scenarios.

We sincerely thank the reviewer for their valuable insight. Based on your suggestion, we have revised the manuscript to add the rationale for evaluating the assembly of high error-rate reads as follows:
“With the advancement of sequencing technologies, there has been a significant leap in both the capabilities and quality of sequencing. For instance, PacBio sequencing technology can offer HiFi reads that provide an accuracy of 99.9%. However, some laboratories continue to rely on older sequencing equipment or encounter suboptimal results due to experimental limitations. In such cases, there is a need for an assembler capable of effectively handling data with relatively higher error rates.” See the subsection “Evaluation on poor sequencing conditions”.

(12) [Evaluation on real data]
The data situation is not entirely clear to me. As I understand, the mock community has sequencing data from five distinct HIV genomes. Then, you test the genome reconstruction with three different subtypes as target. Please address the following questions to better understand the experiment:
a. Why not five targets, one for each genome?

We selected three HIV strains (89.6, JR-CSF, and YU-2) for the assembly analysis because they met our quality criteria. All reads were first aligned to the HXB2 reference genome to standardize the analysis. These three strains have sufficient sequencing depth making them suitable for assembly. In contrast, the NL43 strain are low abundance reads because its reads were of very low quality and had insufficient coverage.

b. I can only assume from the paragraph that 89.6, JR-CSF and YU-2 are members of the five genomes from the mock community. Is that right?
c. Is the backbone genome HXB2 one member of the five genomes in the mock community?

These two questions were deleted by the reviewers.

d. Please add a reference of the mock community data here, or a hint to the Data availability section.

Thanks for your advise, We have added the citation for the mock HIV-1 community dataset in the subsection "Evaluation on real data", which was sourced from the paper "Giallonardo,F.D. et al. (2014) Full-length haplotype reconstruction to infer the structure of heterogeneous virus populations. Nucleic Acids Res., 42, e115." See the bottom of the left column on the page 7.

(13) [Evaluation on real data]
"As shown in Table 5, as for the 89.6 Strain, Canu produces a contig of only 4,593 base pairs"
→ I think that is supposed to be Table 6

Thank you for catching this inconsistency. We have corrected this reference in the revised manuscript.

(14) [Evaluation on real data]Do you have a reason/hypothesis what is causing the remaining mismatches and indels that remain after iterative PVGA? (e.g. in Table 6)

We attribute the residual mismatches and indels after iterative PVGA primarily to inherent limitations of real-world short-read data quality. This hypothesis aligns with the suboptimal performance of other assemblers (e.g., Accuvir, Medaka and PBDAG-Con) in Table 6), which exhibit higher mismatches compared with their performance under simulations reads. Additionally, the lack of validated ground truth for most real datasets complicates definitive error correction. In our manuscript, we illustrated "Due to the lower quality of real reads, assemblers dis-play higher mismatches" in the top of the left column on Page 7.

(15) [Evaluation on real data]
"The results are in Table 7. Our method PVGA exhibits exceptional accuracy […]" → I think that is supposed to be Table 8

Thank you for catching this inconsistency. We have corrected this reference in the revised manuscript.

(16) [Table 8]
Until here I assumed that if the number of mismatches and indels are both zero, an edit distance >0 can only be explained with missing sequence at the flanks of the genome.
However, that's not the case according to the first and third row of Table 8. How is the edit distance >0 while the genome fraction is 100 and the number of mismatches and indels is zero?
Please comment on what's the difference here between reconstructed strain and ground truth. I suppose that is what is meant with "[…] misalignment at the two ends of the genomes."
Is the reconstructed strain slightly longer than the ground truth?

It was a good question. The Genome fraction metric, as defined by QUAST, measures the proportion of the reference genome covered by aligned contigs, calculated by dividing the total aligned reference bases by the genome size.

Shown as the QUAST instructions: “Genome fraction (%): The total number of aligned bases in the reference, divided by the genome size. A base in the reference genome is counted as aligned if at least one contig has at least one alignment to this base. Contigs from repeat regions may map to multiple places, and thus may be counted multiple times in this quantity.”

This metric prioritizes coverage completeness over sequence accuracy. In Table 8, PVGA’s contigs are slightly longer than the ground truth due to non-reference homologous sequences at the termini. While these terminal extensions do not affect the Genome fraction as the entire ground truth is still covered by the aligned regions. they should be counted in edit distance calculations. We have added the ground truth genome lengths in the Table8’s notes.

(17) [Evaluation of computing resource usage]
“Notably, Accuvir[17] exhibited a runtime exceeding 30 minutes and was therefore excluded from subsequent performance figures.” → I couldn’t find running time analyses in the AccuVir publication but they ran analyses on 200x PacBio data. Can you double-check that your code/command was running successfully in general? The running time difference between 1-2 minutes of other tools and AccuVir requiring >30min is surprising.

Thank you for your careful review and valuable suggestion. We have reviewed our code for running Accuvir and confirmed that it takes longer on some datasets due to the numerous search operations. Accuvir employs a local search strategy (beam search) to generate multiple candidate paths and selects the highest-scoring one as the output. To ensure that the candidate paths include a high-quality option, Accuvir uses strategies involving multiple beam search operations, which increases the runtime. While we attempted to adjust parameters to reduce the search space, doing so resulted in faster execution but produced lower-quality output paths compared to the default settings. Therefore, we chose not to reduce the search space for Accuvir, accepting the longer running time.

(18) [Evaluation of computing resource usage]
“As shown in Figure 4, assemblers relying on alignment graph construction tend to require more memory” → Again, I think this is supposed to be Figure 5

Thank you for catching this inconsistency. We have corrected this reference in the revised manuscript.

Response to Reviewer 2’s comments

The authors describe a new reference-guided assembly tool designed for viral genomes. The authors validate their assembly approach and compare with similar tools. The paper is generally well written, and the tool would be valuable to the community.

Thank you for your summary. We really appreciate your time in reviewing our manuscript. We have revised the manuscript accordingly. Our point-by-point responses are detailed below.

The tool is presented as an alternative to de novo assemblers. However, I am interested to know if PVGA was able to correct errors that were present in assemblies that were generated de novo e.g. from Flye and Canu. It seems like PVGA would serve equally well as either a reference-guided assembly tool or as an assembly polishing tool.

Thank you for your question. In the originally submitted manuscript, under the section "Benchmarking the capability of polishing" , We have already evaluated PVGA's polishing ability to correct errors introduced by De Novo assemblers like Canu. We first simulated the SARS-CoV-2 Illumina reads with lengths of 200-300 base pairs and coverages of 25x for polishing the Canu's assembly result. Also, we have expanded in the revised manuscript (Section "Convergence performance across different backbones") by assembling the HIV-1 JRCSF strain using Flye-assembled results as the backbone. When reapplying PVGA with the same reads used in Flye, PVGA perfectly eliminate the edit distance of 20 introduced by Flye, reconstructing the ground-truth genome.

My main concern is a lack of information on the minimum similarity requirements of the reference genome. The quality of the final assembly is entirely dependent on the quality of the original assembly. Incomplete coverage or low similarity will impact the final assembly and it would be useful for users to know at what point they should use an alternative approach (such as my suggestion above: de novo assembly followed by PVGA for polishing).

Thanks for your suggestion. We have conducted additional experiments to evaluate the impact of backbone selection on PVGA's algorithmic convergence. Specifically, we tested four distinct HIV-1 strains (89.6, HXB2, NL43, and YU2) as backbones for assembling reads derived from the JRCSF strain in both real and simulated data. We first compared the sequence similarity between these backbone genomes and the ground truth JRCSF genome. To further assess how backbone divergence affects assembly accuracy, we introduced manual random errors (15%, 20%, 25%, and 30%) into the JRCSF genome and used these modified sequences as backbones to assemble simulated reads with 5% error rates. The results (Table 11) demonstrate that even with a 30% error rate in the backbone, PVGA maintained robust assembly accuracy. This is attributed to the backbone contributing only a weight of 1 for base connectivity decisions, while read-derived evidence (proportional to coverage) dominated the alignment graph construction. However, higher divergence between the backbone and the ground truth led to shorter assembly lengths, specifically manifesting as fragment loss at the genome ends, while the central regions remained error-free as shown in Table 11. This occurs because regions at the ends of the backbone with higher divergence showed reduced alignment efficiency: reads spanning these divergent regions failed to align to the backbone and thus could not contribute to the graph construction.

I would strongly recommend adding the tool to bioconda. Can you use the bioconda version of blasr? I believe all the dependencies in requirements should be available on conda. You should also be to publish as a python package.

Thanks for your valuable suggestion, we have already contributed PVGA to bioconda. Now users can install and run the tool seamlessly without manually configuring their environments. The download command is provided below:
conda create --name pvga python=3.10
conda activate pvga
conda install -c bioconda pvga

Misc comments:
"The virus genomes have relatively small size."
All? Some?

Thanks for your suggestion. We have removed this sentence.

"As the basic structure of the virus genomes is highly conserved …"
Do you have a citation for this? As this is a reference-guided assembly tool, the quality of the final assembly is dependent on having adequate structural and sequence similarity to the reference used.

Thank you for your feedback. We have revised the statement to: "As the basic structure of many virus genomes is highly conserved…" The citation supporting this claim is derived from "Conserved RNA secondary structures in viral genomes: a survey" which illustrates that "The genomes of RNA viruses often carry conserved RNA structures that perform vital functions during the life cycle of the virus. See the

begging part of Section “Introduction”.

"However, these longer reads tend to have higher error rates."
I don't believe this is nearly as relevant as it would have been 5 years ago. PacBio HiFi reads are very accurate and nanopore is not far behind.

We have revised the statement to “In comparison with NGS, TGS tends to have higher error rates.” In addition, we added discussion on this part in this paper to deepen the necessity of using assembler for high error rate reads’ assembly in “Evaluation on poor sequencing conditions” section as follows：
“For instance, PacBio sequencing technology can offer HiFi reads that provide an accuracy of 99.9\%. However, some laboratories continue to rely on older sequencing equipment or encounter suboptimal results due to experimental limitations. In such cases, there is a need for an assembler capable of effectively handling data with relatively higher error rates.”
See the begging part of the subsection “Evaluation on poor-sequencing conditions”

"Famous reference-guided assembly methods include BWA [11], Bowtie2 [12], GATK [13], Novoalign [14]..."
These are all read alignment programs if I'm not mistaken.

Thanks for your valuable suggestion. We have carefully reviewed your comments and agree that the tools mentioned (BWA, Bowtie, and GATK) are primarily designed for read mapping and variant calling rather than reference-guided assembly. While these tools are often used as part of the preprocessing steps for reference-guided assembly, they do not directly produce consensus sequences or contigs, which are the key outputs of an assembly process. We have removed the description of these tools from the section on reference-based assembly. Instead, we now focus on tools specifically designed for reference-guided assembly. The comparison before and after modification is as follows:

Origin: “Famous reference-guided assembly methods include BWA, Bowtie2, GATK, Novoalign, and Maq.”

Revised: “Famous reference-guided assembly methods include Novoalign, Maq, iVar, Accuvir and bcftools.”

"For instance, IVA was developed as a De Novo assembler for RNA viruses, utilizing paired-end datasets to achieve more accurate assemblies [16]. Similarly, Accuvir introduced a reference-based long-read assembler for viruses, primarily employing diverse beam search algorithms on alignment graphs to improve accuracy [17]."
You could expand on how the PVGA approach compares with these other established tools.

Thanks for your comment, we have added the motivation of designing PVGA as follows:
“However, despite these advancements, current viral genome assembly tools still fail to achieve the requisite base-level accuracy.” And we had a comprehensive comparison in Results section.

"PVGA starts with a reference genome and utilizes the sequencing reads directly to reduce noise."
Reduce what noise? How?

The term "noise" in this context refers to the sequence divergence between the backbone genome and the ground truth. PVGA mitigates such noise through an iterative graph refinement process as shown in the section “Results” and the section “Method”.

"The results demonstrate that PVGA consistently outperforms the best existing programs"
I would reword 'the best programs' to something like 'popular existing programs'

Thanks for your suggestion. We have revised it.

Figure 1: do the reads get remapped each iteration?

Yes, as described in the subsection "Updating the Reference Genome Iteratively", the alignment graph is

dynamically reconstructed during each iteration by realigning all input reads to the updated backbone genome.

Is there a hard cap on the number of iterations?

The upper bound of iteration number is PVGA’s optional parameter. Users can specify an upper limit for the number of iterations via the -n parameter (default: 10).

Editorial Manager®

Cookie Preference Center

We use cookies which are necessary to make our site work. We may also use additional cookies to analyze, improve and personalize our content and your digital experience. For more information, see our Cookie Policy.

You may choose not to allow some types of cookies. However, blocking some types may impact your experience of our site and the services we are able to offer. See the different category headings below to find out more or change your settings.

Allow all

Manage Consent Preferences

Strictly Necessary Cookies

Always active

These cookies are necessary for the website to function and cannot be switched off in our systems. They are usually only set in response to actions made by you which amount to a request for services, such as setting your privacy preferences, logging in or filling in forms. You can set your browser to block or alert you about these cookies, but some parts of the site will not then work. These cookies do not store any personally identifiable information.

Functional Cookies

These cookies enable the website to provide enhanced functionality and personalisation. They may be set by us or by third party providers whose services we have added to our pages. If you do not allow these cookies then some or all of these services may not function properly.

Performance Cookies

These cookies allow us to count visits and traffic sources so we can measure and improve the performance of our site. They help us to know which pages are the most and least popular and see how visitors move around the site.

Targeting Cookies

These cookies may be set through our site by our advertising partners. They may be used by those companies to build a profile of your interests and show you relevant adverts on other sites. If you do not allow these cookies, you will experience less targeted advertising.

Cookie List

Clear

Apply Cancel
Consent Leg.Interest

Confirm my choices

Powered by onetrus
