# Supplementary material for: PVGA: a precise viral genome assembler using an iterative alignment graph
Source: Gigascience. 2025 Jun 24;14:giaf063. doi: 10.1093/gigascience/giaf063 (PMC12206156; doi:10.1093/gigascience/giaf063)
Supplement: giaf063_Reviewer_2_Report_Original_Submission — Michael Roach -- 2/4/2025 [file giaf063_reviewer_2_report_original_submission.pdf]

Reviewer Report

Title: PVGA: A Precise Viral Genome Assembler Using Iterative Alignment Graph

Version: Original Submission **Date:** 2/4/2025

Reviewer name: Michael Roach

Reviewer Comments to Author:

The authors describe a new reference-guided assembly tool designed for viral genomes. The authors validate their assembly approach and compare with similar tools. The paper is generally well written, and the tool would be valuable to the community.The tool is presented as an alternative to de novo assemblers. However, I am interested to know if PVGA was able to correct errors that were present in assemblies that were generated de novo e.g. from Flye and Canu. It seems like PVGA would serve equally well as either a reference-guided assembly tool or as an assembly polishing tool.My main concern is a lack of information on the minimum similarity requirements of the reference genome. The quality of the final assembly is entirely dependent on the quality of the original assembly. Incomplete coverage or low similarity will impact the final assembly and it would be useful for users to know at what point they should use an alternative approach (such as my suggestion above: de novo assembly followed by PVGA for polishing).I would strongly recommend adding the tool to bioconda. Can you use the bioconda version of blasr? I believe all the dependencies in requirements should be available on conda. You should also be to publish as a python package.Misc comments:"The virus genomes have relatively small size."All? Some?"As the basic structure of the virus genomes is highly conserved …"Do you have a citation for this? As this is a reference-guided assembly tool, the quality of the final assembly is dependent on having adequate structural and sequence similarity to the reference used."However, these longer reads tend to have higher error rates."I don't believe this is nearly as relevant as it would have been 5 years ago. PacBio HiFi reads are very accurate and nanopore is not far behind."Famous reference-guided assembly methods include BWA [11], Bowtie2 [12], GATK [13], Novoalign [14]..."These are all read alignment programs if I'm not mistaken."For instance, IVA was developed as a De Novo assembler for RNA viruses, utilizing paired-end datasets to achieve more accurate assemblies [16]. Similarly, Accuvir introduced a reference-based long-read assembler for viruses, primarily employing diverse beam search algorithms on alignment graphs to improve accuracy [17]."You could expand on how the PVGA approach compares with these other established tools."PVGA starts with a reference genome and utilizes the sequencing reads directly to reduce noise."Reduce what noise? How?"The results demonstrate that PVGA consistently outperforms the best existing programs"I would reword 'the best programs' to something like 'popular existing programs'Figure 1: do the reads get remapped each iteration? Is there a hard cap on the number of iterations?

Level of Interest

Please indicate how interesting you found the manuscript: Choose an item.

Quality of Written English

Please indicate the quality of language in the manuscript: Choose an item.

Declaration of Competing Interests

Please complete a declaration of competing interests, considering the following questions:

- Have you in the past five years received reimbursements, fees, funding, or salary from an organisation that may in any way gain or lose financially from the publication of this manuscript, either now or in the future?
- Do you hold any stocks or shares in an organisation that may in any way gain or lose financially from the publication of this manuscript, either now or in the future?
- Do you hold or are you currently applying for any patents relating to the content of the manuscript?
- Have you received reimbursements, fees, funding, or salary from an organization that holds or has applied for patents relating to the content of the manuscript?
- Do you have any other financial competing interests?
- Do you have any non-financial competing interests in relation to this paper?

If you can answer no to all of the above, write 'I declare that I have no competing interests' below. If your reply is yes to any, please give details below.

I declare that I have no competing interests

I agree to the open peer review policy of the journal. I understand that my name will be included on my report to the authors and, if the manuscript is accepted for publication, my named report including any attachments I upload will be posted on the website along with the authors' responses. I agree for my report to be made available under an Open Access Creative Commons CC-BY license (http://creativecommons.org/licenses/by/4.0/). I understand that any comments which I do not wish to be included in my named report can be included as confidential comments to the editors, which will not be published.

Choose an item.

To further support our reviewers, we have joined with Publons, where you can gain additional credit to further highlight your hard work (see: https://publons.com/journal/530/gigascience). On publication of this paper, your review will be automatically added to Publons, you can then choose whether or not to claim your Publons credit. I understand this statement.

Yes Choose an item.
